# Enantioselective Synthesis of Spiroindenes by Enol-Directed Rhodium(III)-Catalyzed C–H Functionalization and Spiroannulation

**DOI:** 10.1002/anie.201507029

**Published:** 2015-09-25

**Authors:** Suresh Reddy Chidipudi, David J Burns, Imtiaz Khan, Hon Wai Lam

**Affiliations:** School of Chemistry, University of Nottingham University Park, Nottingham, NG7 2RD (UK) E-mail: hon.lam@nottingham.ac.uk Homepage: http://www.nottingham.ac.uk/~pczhl

**Keywords:** alkynes, catalysis, C–H activation, enantioselective, rhodium

## Abstract

Chiral cyclopentadienyl rhodium complexes promote highly enantioselective enol-directed C(sp^2^)-H functionalization and oxidative annulation with alkynes to give spiroindenes containing all-carbon quaternary stereocenters. High selectivity between two possible directing groups, as well as control of the direction of rotation in the isomerization of an *O*-bound rhodium enolate into the *C*-bound isomer, appear to be critical for high enantiomeric excesses.

Cyclopentadienyl rhodium(III) complexes are well-established as highly active and versatile precatalysts in a diverse array of C–H functionalization reactions.[1] However, enantioselective variants of these reactions only became possible with the development of chiral *C*_2_-symmetric cyclopentadienyl ligands by Ye and Cramer,[2] and an artificial Rh^III^-containing metalloenzyme by Ward, Rovis, and co-workers.[3] To date, a handful of catalytic enantioselective Rh^III^-catalyzed C–H functionalizations have been described,[2–5] but there is a compelling need to develop new processes to access novel classes of enantioenriched products.[6]

We recently reported Ru- and Pd-catalyzed oxidative annulations of α-aryl cyclic 1,3-dicarbonyl compounds (or their enol tautomers) with alkynes that provide achiral or racemic spiroindenes.[7] Given that indenes appear in several biologically active compounds,[8, 9] the ability to prepare chiral spiro-fused indenes **4** by asymmetric C–H functionalization would be valuable.[4d, 10] Because we also found that [{Cp*RhCl_2_}_2_] is an effective precatalyst,[7a, 11] chiral cyclopentadienyl rhodium complexes **3** appeared to be highly promising for investigation. However, in contrast to existing enantioselective Rh^III^-catalyzed C–H functionalizations, which all rely upon aryl C(sp^2^)–H activation of substrates containing a single directing group (Scheme 1 a),[2–5] the substrates **1** required for our proposed study contain two potential directing groups (Scheme 1 b). Within the accepted model for enantioinduction using complexes **3**,[2b, 5] cyclorhodation can generate up to four species, which differ in which directing group participates in cyclometallation, and/or the orientation of the rhodacycle within the chiral pocket (Scheme 2). This situation contrasts with existing processes,[2–5] including the dearomatizing oxidative spiroannulations of You and co-workers,[4d] in which only two conformations of one rhodacycle need to be considered. Given the possibility of other reaction pathways with potentially different stereochemical outcomes, the development of a highly enantioselective process was far from certain. Herein, we report the successful realization of asymmetric [3+2] spiroannulations to give a diverse range of spiroindenes in up to 97 % *ee.*

**Scheme 1:**
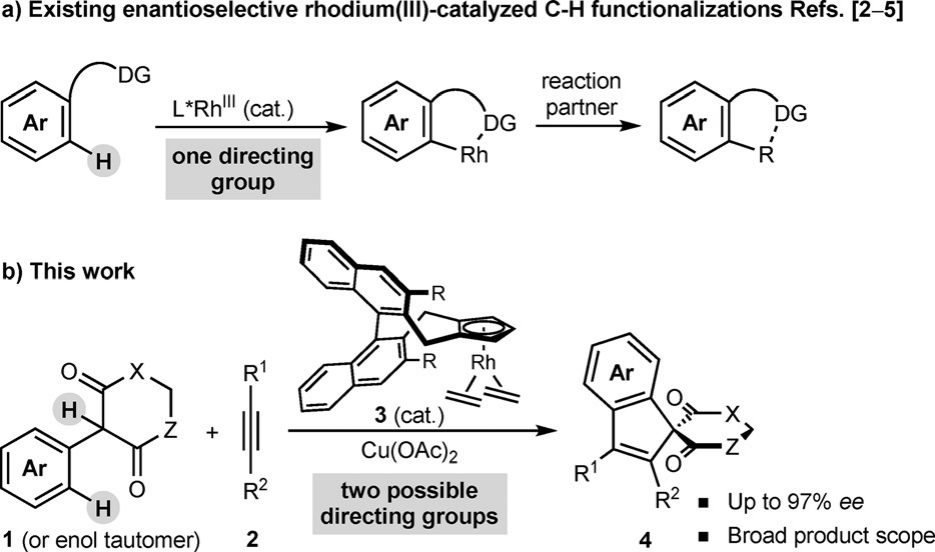
Enantioselective Rh^III^-catalyzed C–H functionalizations.

**Scheme 2:**
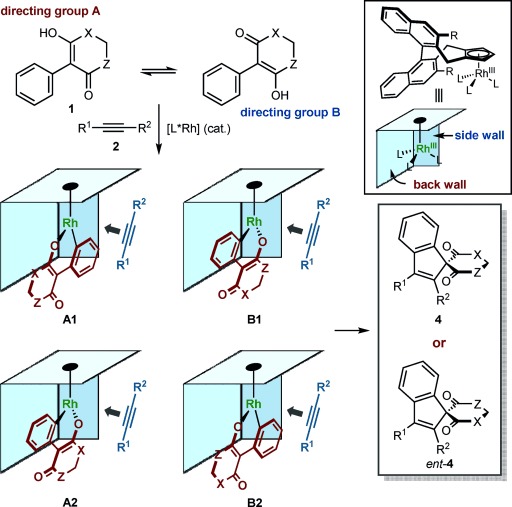
Possible species to consider upon cyclorhodation.

Our investigations began with an evaluation of chiral cyclopentadienyl rhodium complexes **3 a**–**3 f**[2b] in the reaction of 4-hydroxy-6-methyl-3-phenyl-2*H*-pyran-2-one (**1 a**) with 1-phenylpropyne (**2 a**, 1.5 equiv), using Cu(OAc)_2_ (2.1 equiv) in DMF[12] at 50 °C for 24 h (Table 1). Benzoyl peroxide, which was employed as an additive in previous enantioselective Rh-catalyzed C–H functionalizations,[2, 4] was unnecessary,[13] and in all cases, only one regioisomer of spiroindene **4 a** was detected. The parent complex **3 a** (R=H) gave **4 a** in 93 % NMR yield, but the enantioselectivity was moderate (entry 1).[14] Higher selectivities were obtained with complexes **3b–3f** containing larger groups at the 3,3′-positions (entries 2–6). The OTBDPS- containing complex **3 f** was optimal, and provided **4 a** in high NMR yield and 95 % *ee* (entry 6).

**Table 1:** Catalyst evaluation in the reaction of 1 a with 2 a^[a]^ 
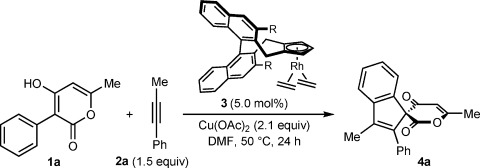

Entry	Rh complex3	NMR Yield [%]^[b]^	*ee* [%]^[c]^
1	**3 a** R=H	93	58
2	**3 b** R=OMe	97	90
3	**3 c** R=O*i*Pr	33	78
4	**3 d** R=Ph	41	88
5	**3 e** R=OTIPS	84	92
6	**3 f** R=OTBDPS	98	95

[a] Reactions were conducted with 0.05 mmol of **1 a**. [b] Determined by ^1^H NMR spectroscopy using 1,3,5-trimethoxybenzene as an internal standard. [c] Determined by HPLC analysis on a chiral stationary phase. TIPS=triisopropylsilyl, TBDPS=*tert*-butyldiphenylsilyl.

With an effective chiral complex identified, the enantioselective spiroannulation of **1 a** with various alkynes was explored (Scheme 3). With unsymmetrical alkynes, the regioselectivities of these reactions were excellent, and with the exception of spiroindene **4 d**, which was formed as a 19:1 regioisomeric mixture, only single regioisomers were detected. With 1-phenylpropyne (**2 a**), spiroindene **4 a** was isolated in 84 % yield and 95 % *ee*. The same reaction run at room temperature provided **4 a** in 78 % yield and 97 % *ee*. Diphenylacetylene reacted to give spiroindene **4 b** in 67 % yield and 93 % *ee*, whereas a symmetrical dialkyl alkyne gave spiroindene **4 c** in moderate yield and enantioselectivity. However, other alkyl/(hetero)aryl alkynes were excellent reaction partners. For example, alkynes containing 5-indolyl, 3-indolyl, or 2-thienyl substituents provided spiroindenes **4 d**–**4 f** in 74–93 % yield and 89–97 % *ee*.

**Scheme 3:**
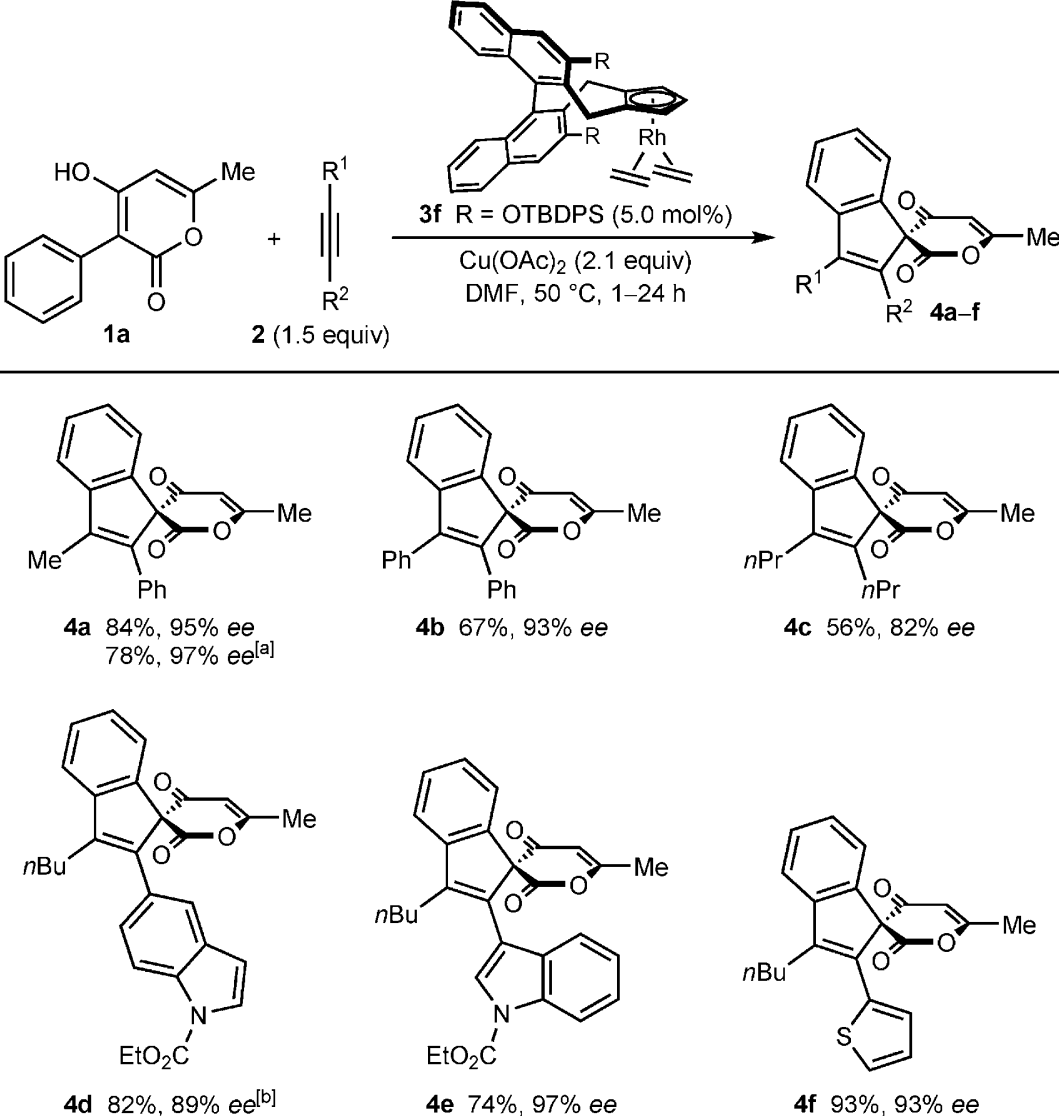
Enantioselective oxidative annulations of 1 a with various alkynes. Reactions were conducted with 0.30 mmol of 1 a. Yields are of isolated products. Enantiomeric excesses were determined by HPLC analysis on a chiral stationary phase. [a] Conducted with 0.20 mmol of 1 a at room temperature for 24 h. [b] Formed as a 19:1 mixture of regioisomers as determined by ^1^H NMR of the unpurified reaction mixture. The isolated product was also a 19:1 mixture of regioisomers.

Various other substrates also underwent the spiroannulation with a range of alkynes to give spiroindenes containing ketoesters (**4 g**–**4 l**, **4 q**, and **4 r**), ketolactams (**4 m**–**4 p** and **4 t**–**4 v**), a diketone (**4 s**), or a barbiturate (**4 w**) with generally high enantioselectivities (Scheme 4). Although complex **3 f** was generally effective, in some cases the less sterically hindered complex **3 b** gave superior yields and enantioselectivities (**4 t**–**4 w**). The reason for the superiority of complex **3 b** in these cases is not currently known. Substitution at the *meta*- or *para*-position of the α-phenyl group was tolerated (**4 g**–**4 i**). With a *meta*-CF_3_ group, C–H functionalization occurred at the more sterically accessible site (**4 g**).[14] In our previous oxidative annulation work,[7] only six-membered cyclic 1,3-dicarbonyl compounds were employed. Therefore, it is notable that, for the first time, five- and seven-membered substrates could be employed (**4 l**, **4 m**, and **4 o**). The low yield of **4 l** is attributed to its instability under the reaction conditions. Products containing the 1,3-dicarbonyl component within various polycyclic ring systems were also prepared (**4 p**–**4 r** and **4 v**), although the enantioselectivities of **4 p** and **4 q** were more modest. A substrate in which the two possible directing groups are almost identical electronically, but sterically well-differentiated, gave spiroindene **4 s** in 77 % yield and a reasonable 78 % *ee*. 1-Methyl-5-phenylbarbituric acid, in which the two carbonyl groups adjacent to the phenyl group are electronically and sterically similar, gave spiroindene **4 w** with low enantioselectivity. Finally, several of the reactions could be carried out in dimethyl carbonate, a significantly more environmentally friendly solvent than DMF (**4 i**, **4 t**, and **4 u**).[15]

**Scheme 4:**
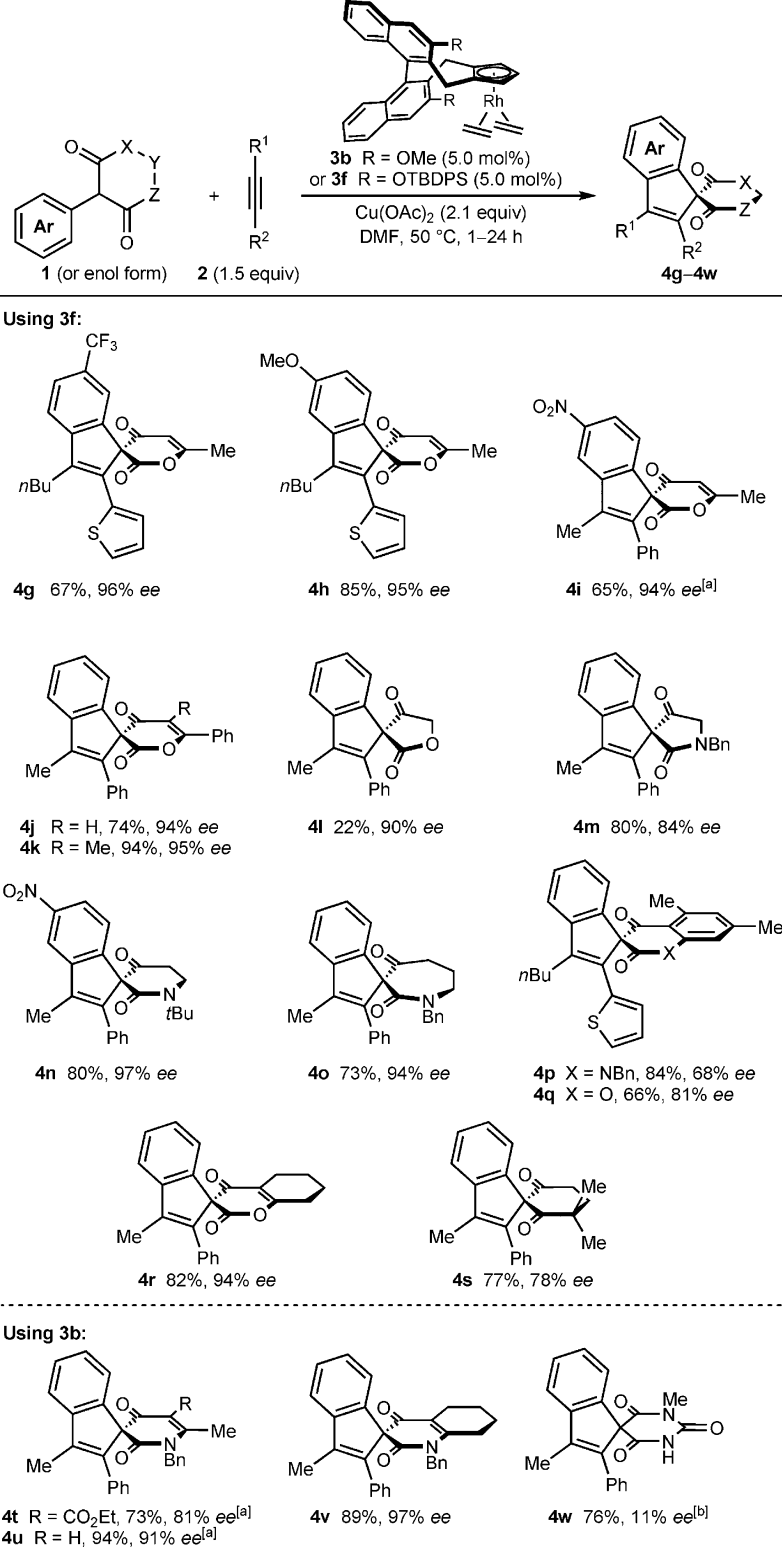
Reactions were conducted with 0.20 or 0.30 mmol of 1 (see Supporting Information for details). Yields are of isolated products. Enantiomeric excesses were determined by HPLC analysis on a chiral stationary phase. [a] Dimethyl carbonate was used as the solvent. [b] The absolute stereochemistry of the major enantiomer of 4 w is not known.

To gain further insight into these annulations, deuteration reactions were conducted. Treatment of **1 c** under the standard conditions in the absence of an alkyne but with the addition of D_2_O for 4 h led to recovery of [D]_*n*_-**1 c** with 5 % deuteration at the *ortho*-positions of the arene only (Scheme 5 a). Furthermore, reaction of **1 c** with alkyne **2 f** under the same conditions led to recovered **1 c** with no observable deuteration, and spiroindene [D]_*n*_-**4 h** that was partially deuterated at the pyran-2,4-dione ring[16] but not at the arene (Scheme 5 b). Interestingly, the presence of D_2_O decreased the regioselectivity of this reaction compared to the one conducted in DMF only (Scheme 4), and [D]_*n*_-**4 h** was isolated as an 8:1 mixture of inseparable regioisomers. The experiments shown in Scheme 5 suggest that cyclorhodation is largely irreversible under these conditions.

**Scheme 5:**
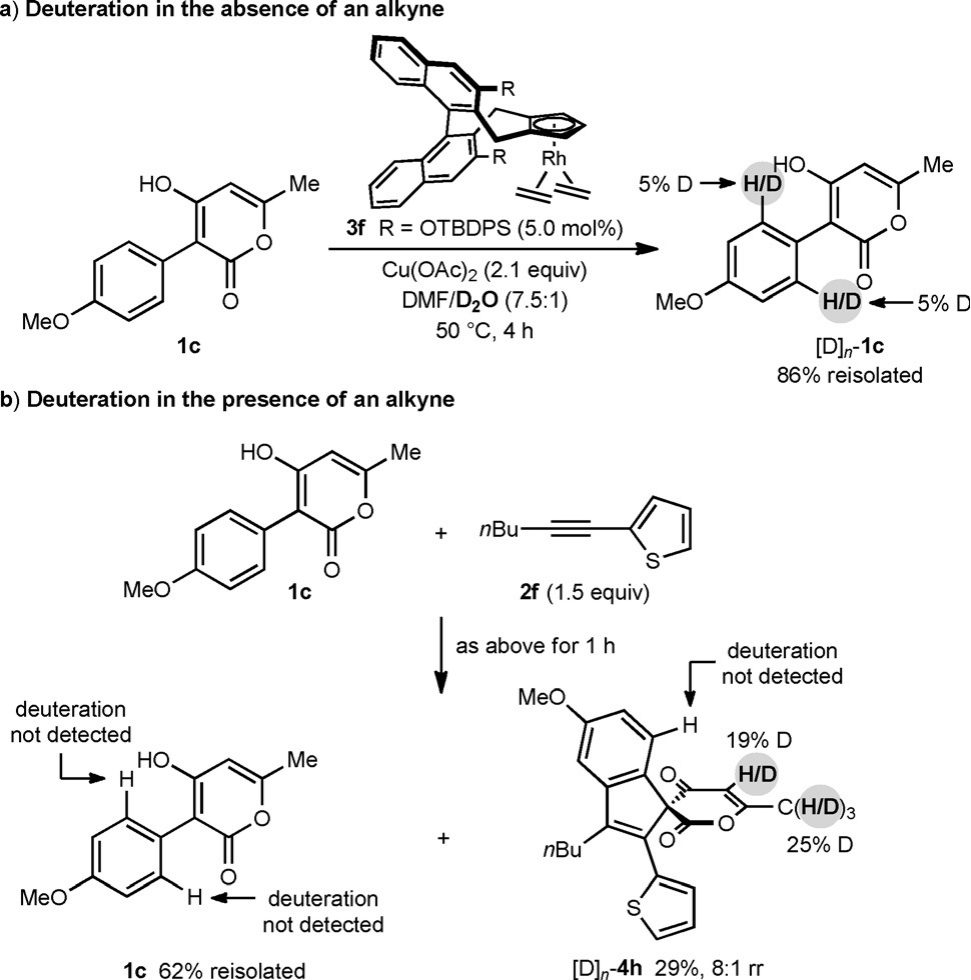
Deuteration experiments.

A proposed catalytic cycle and stereochemical model[14] for these reactions is shown in Scheme 6 a, using **1 a** and **2 a** as representative substrates. After formation of rhodium diacetate complex **5**, we assume that cyclorhodation of **1 a** is promoted by the most enolizable of the two possible directing groups, which is the enol derived from the ketone rather than the ester, to give rhodacycle **6 a**. Coordination and migratory insertion of the alkyne would then give rhodacycle **7 a**. The alternative conformations **6 b** and **7 b** appear to be disfavored because of steric interactions between the side wall of the cyclopentadienyl ligand with the metallated arene of **6 b** or the phenyl substituent of **7 b** (Scheme 6 b). The next step is the isomerization of the *O*-bound rhodium enolate **7 a** into the *C*-bound isomer **8 a**, presumably through an oxa-π-allylrhodium species, which requires a rotation of the 4-alkoxypyran-2-one moiety. Because the rhodium alkoxide of this moiety is in closest proximity to the chiral ligand, it experiences the greatest steric interactions, and we propose there is a preference for this group to rotate away from the ligand to give **8 a**. Reductive elimination of **8 a** gives spiroindene **4 a** and Rh^I^ species **9**, which is oxidized by Cu(OAc)_2_ to regenerate **5**. The formation of the minor enantiomer from **7 a** requires an unfavorable rotation of the rhodium alkoxide towards the back wall of the chiral ligand (Scheme 6 c).

**Scheme 6:**
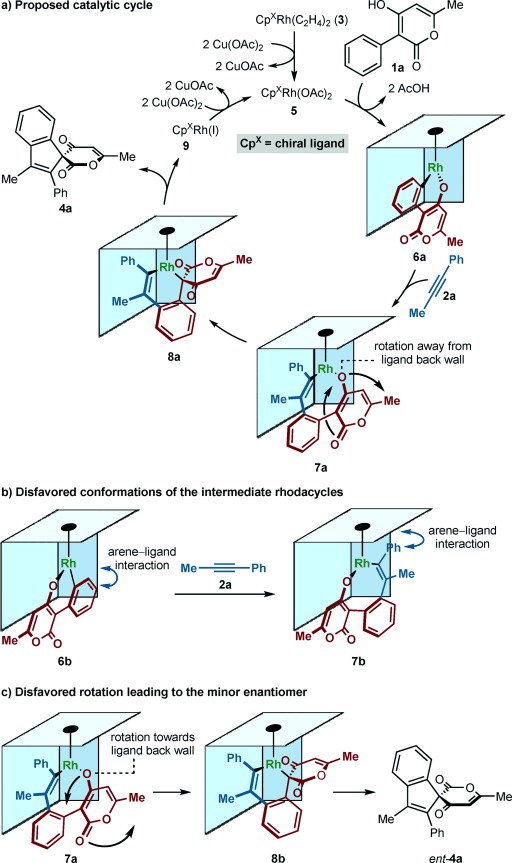
Proposed catalytic cycle and stereochemical model.

An alternative explanation that cannot be excluded is that migratory insertion of **6 a** with the alkyne directly produces a rhodacycle with a conformation closely related to that of **7 a**, but with the rhodium alkoxide already partially rotated away from the chiral ligand. Continued rotation of the 4-alkoxypyran-2-one moiety in the same direction, according to the principle of least motion,[17] would then give **8 a**.

In conclusion, we have developed an enantioselective synthesis of spiroindenes from the oxidative annulation of α-aryl cyclic 1,3-dicarbonyl compounds (or their enol tautomers) with alkynes, using chiral cyclopentadienyl rhodium catalysts. The process tolerates a wide range of substrates to give diverse products containing all carbon-quaternary stereocenters with high enantioselectivities. Application of these chiral complexes in other classes of C–H functionalization/oxidative annulation is underway, and these results will be reported in due course.

## Supporting Information

As a service to our authors and readers, this journal provides supporting information supplied by the authors. Such materials are peer reviewed and may be re-organized for online delivery, but are not copy-edited or typeset. Technical support issues arising from supporting information (other than missing files) should be addressed to the authors.

miscellaneous_information
