# Supplementary material for: Enantioselective Synthesis of Spiroindenes by Enol-Directed Rhodium(III)-Catalyzed C–H Functionalization and Spiroannulation
Source: Angew Chem Int Ed Engl. 2015 Sep 25;54(47):13975–9. doi: 10.1002/anie.201507029 (PMC4648053; doi:10.1002/anie.201507029)
Supplement: Supplementary file 1 — miscellaneous_information [file anie0054-13975-sd1.pdf]

Angewandte Chemie
Eine Zeitschrift der Gesellschaft Deutscher Chemiker

Supporting Information

Enantioselective Synthesis of Spiroindenes by Enol-Directed Rhodium(III)-Catalyzed C–H Functionalization and Spiroannulation

*Suresh Reddy Chidipudi, David J. Burns, Imtiaz Khan, and Hon Wai Lam**

anie_201507029_sm_miscellaneous_information.pdf

Supporting Information

Contents **Page**

General Information….. 2
Synthesis of Substrates... 3
Preparation of Chiral Cyclopentadienyl Rh(I) Metal Complexes... 20
Enantioselective Rhodium-Catalyzed Spiroannulation Reactions.. 24
Stereochemical Determinations... 49
Deuteration Experiments.. 51
Tests for the Self-Disproportionation of Enantiomers (SDE) Phenomenon............................ 54
NMR Spectra.. 56

General Information

Unless specified otherwise, all reactions were carried out under an atmosphere of nitrogen using oven-dried glassware. Unless specified otherwise, all commercially available reagents and solvents were used as received. THF was dried and purified by passage through activated alumina columns using a solvent purification system. 'Petrol' refers to that fraction of light petroleum ether boiling in the range 40–60 °C. Thin layer chromatography (TLC) was performed on Merck DF-Alufoilien $60F_{254}$ 0.2 mm precoated plates. Compounds were visualized by exposure to UV light or by dipping the plates into solutions of potassium permanganate or vanillin followed by heating. Flash column chromatography was carried out using silica gel (Fisher Scientific 60 Å particle size 35–70 micron). Melting points were recorded on a Gallenkamp melting point apparatus and are uncorrected. The solvent of recrystallization is reported in parentheses. Infra-red spectra were recorded on a Nicolet Avatar 360 FT instrument on the neat compound using the attenuated total refraction technique. NMR spectra were acquired on Bruker DPX300, AV400, AV(III)400, DPX400, or AV(III)500 spectrometers at room temperature unless otherwise stated. ^{1}H and ^{13}C NMR spectra were referenced to external tetramethylsilane *via* the residual protonated solvent (^{1}H) or the solvent itself (^{13}C). All chemical shifts are reported in parts per million (ppm). For $CDCl_3$, the shifts are referenced to 7.27 ppm for ^{1}H NMR spectroscopy and 77.0 ppm for ^{13}C NMR spectroscopy. For $(CD_3)_2SO$, the shifts are referenced to 2.50 ppm for ^{1}H NMR spectroscopy and 39.52 ppm for ^{13}C NMR spectroscopy. Proton-decoupled ^{19}F NMR spectra were recorded on a Bruker AVA400 (376 MHz) or DPX300 (282.2 MHz) spectrometers. Chemical shifts (δ) are quoted in parts per million (ppm) downfield of $CFCl_3$ (δ = 0 ppm), using fluorobenzene (C_6H_5F at –113.5 ppm) or trifluoroacetic acid (CF_3CO_2H at −76.55 ppm), as internal standard. High-resolution mass spectra were recorded using electrospray ionization (ESI) techniques at the School of Chemistry, University of Nottingham. Optical rotations were performed on a Bellingham and Stanley ADP 400 polarimeter. Chiral HPLC analysis was performed on an Agilent 1290 series instrument using 4.6 x 250 mm columns. Authentic racemic samples of products were obtained using $[Cp^{*}RhCl_2]_2$.

Synthesis of Substrates

Preparation of Substrate 1a[1]

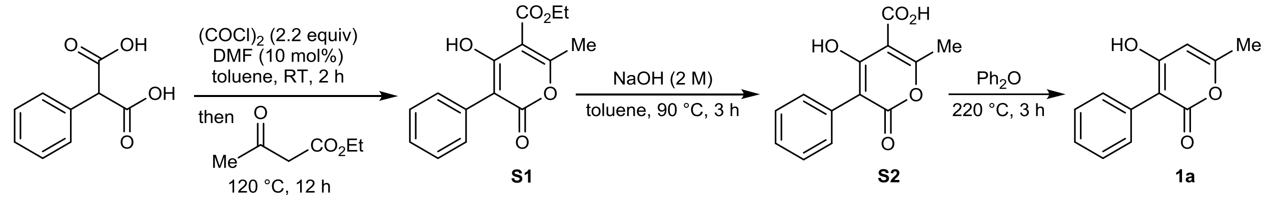

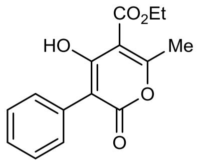

Ethyl 4-hydroxy-6-methyl-2-oxo-3-phenyl-2*H*-pyran-5-carboxylate (S1). To a stirred suspension of phenylmalonic acid (5.00 g, 27.7 mmol) and DMF (0.21 mL, 2.81 mmol) in toluene (80 mL) was added oxalyl chloride (5.2 mL, 61.0 mmol) dropwise, and the resulting mixture was stirred at room temperature until effervescence had ceased. Ethyl acetoacetate (3.5 mL, 27.7 mmol) was added and the resulting mixture was heated at 120 °C for 12 h. The mixture was concentrated *in vacuo* and the residue was purified by flash column chromatography (15% acetone/petrol) to give the *pyranone* **S1** as a pale brown solid (3.37 g, 44%). R_f 0.30 (20% acetone/petrol); m.p. 68–69 °C (EtOAc/petrol); IR 1732 (C=O), 1717 (C=O), 1683, 1675, 1558, 1541, 1417, 1289, 1099, 998, 900, 824, 734 cm^{-1}; ^{1}H NMR (300 MHz, $CDCl_3$) δ 12.04 (1H, s, O**H**), 7.55–7.48 (2H, m, Ar**H**), 7.41 (2H, t, *J* = 7.4 Hz, Ar**H**), 7.36–7.26 (1H, m, Ar**H**), 4.44 (2H, q, *J* = 7.1 Hz, OC**H**$_2$), 2.68 (3H, s, C**H**$_3$), 1.42 (3H, t, *J* = 7.1 Hz, OCH_2C**H**$_3$); ^{13}C NMR (100.6 MHz, $CDCl_3$) δ 171.9 (C), 169.2 (C), 164.3 (C), 161.3 (C), 130.5 (C), 130.2 (2 × CH), 127.8 (2 × CH), 127.5 (CH), 103.1 (C), 101.7 (C), 63.0 (CH_2), 22.5 (CH_3), 13.9 (CH_3); HRMS (ESI +ve) Exact mass calculated for $C_{15}H_{15}O_5$ $[M+H]^+$: 275.0914, found: 275.0929.

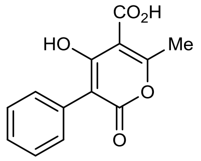

4-Hydroxy-6-methyl-2-oxo-3-phenyl-2*H*-pyran-5-carboxylic acid (S2). To a stirred solution of ester **S1** (3.37 g, 12.3 mmol) in toluene (24 mL) was added aqueous 2 M NaOH solution (18.4 mL, 36.8 mmol) slowly, and the reaction mixture was stirred at 90 °C for 3 h. After cooling to room temperature, the aqueous layer was separated and washed with toluene (50 mL). The aqueous layer was acidified to pH 2–3 using aqueous 10% HCl solution and extracted with EtOAc (2 × 100 mL). The combined organic extracts were washed with water (100 mL), brine (100 mL), dried (Na_2SO_4), and concentrated *in vacuo* to leave a pale brown solid. Purification by trituration (20% acetone/petrol) gave the *carboxylic acid* **S2** as a pale brown solid (2.56 g, 85%). m.p. 208–209 °C (EtOAc/petrol); IR 1675 (C=O), 1652, 1646, 1558, 1521, 1456, 1420, 1253, 1230, 1109, 1004, 833, 726 cm^{-1}; ^{1}H

1. Heidenbluth, B. *Chem. Ber.* **1958**, *91*, 2849–2853.

NMR (500 MHz, $(CD_3)_2SO$) δ 12.15 (2H, br s, O**H** and COO**H**), 7.44–7.38 (2H, m, Ar**H**), 7.37–7.30 (2H, m, Ar**H**), 7.27–7.21 (1H, m, Ar**H**), 2.62 (3H, s, C**H**$_3$); ^{13}C NMR (125.8 MHz, $(CD_3)_2SO$) δ 170.5 (C), 170.4 (C), 167.0 (C), 161.2 (C), 132.2 (C), 130.5 (2 × CH), 127.5 (2 × CH), 126.6 (CH), 103.1 (C), 100.9 (C), 21.2 (CH_3); HRMS (ESI +ve) Exact mass calculated for $C_{13}H_{11}O_5$ $[M+H]^+$: 247.0601, found: 247.0602.

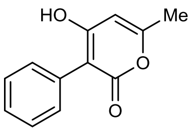

4-Hydroxy-6-methyl-3-phenyl-2*H*-pyran-2-one (1a). A stirred suspension of acid **S2** (2.56 g, 10.4 mmol) in Ph_2O (20 mL) was heated at 220 °C for 3 h. The mixture was cooled to room temperature, diluted with petrol, and stirred for 1 h to give a clear suspension, which upon filtration afforded a light brown solid which was purified by trituration (20% acetone/petrol) to give the *pyranone* **1a** as a pale brown solid (1.74 g, 83%). R_f 0.20 (20% acetone/petrol); m.p. 226–227 °C (EtOH); IR 1663 (C=O), 1636, 1558, 1540, 1436, 1403, 1358, 1337, 1313, 1181, 1001, 841, 774, 748 cm^{-1}; ^{1}H NMR (300 MHz, $(CD_3)_2SO$) δ 11.18 (1H, br s, O**H**), 7.40–7.28 (4H, m, Ar**H**), 7.26–7.18 (1H, m, Ar**H**), 6.14 (1H, s, C**H**=CCH$_3$), 2.21 (3H, s, C**H**$_3$); ^{13}C NMR (100.6 MHz, $(CD_3)_2SO$) δ 165.5 (C), 163.6 (C), 161.3 (C), 132.4 (C), 130.5 (2 × CH), 127.4 (2 × CH), 126.5 (CH), 101.5 (C), 100.2 (CH), 19.4 (CH_3); HRMS (ESI +ve) Exact mass calculated for $C_{12}H_{10}NaO_3$ $[M+Na]^+$: 225.0522, found: 225.0530.

Preparation of Substrate 1b

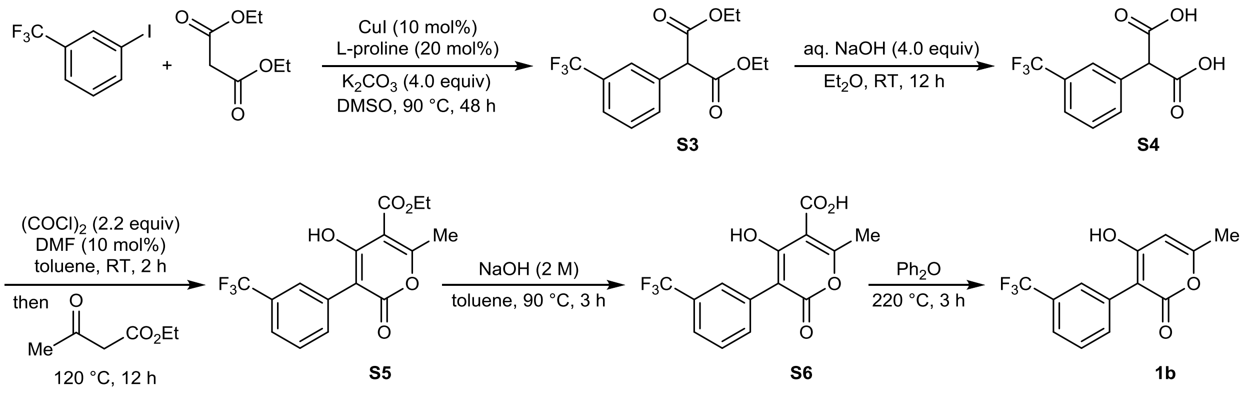

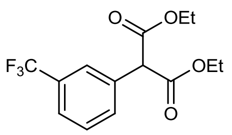

1,3-Diethyl 2-[3-(trifluoromethyl)phenyl]propanedioate (S3). 1-Iodo-3-(trifluoromethyl)benzene (25.0 g, 91.9 mmol) was added to a stirred solution of CuI (1.75 g, 9.19 mmol), L-proline (2.12 g, 18.4 mmol), K_2CO_3 (50.7 g, 368 mmol), and diethyl malonate (42.1 mL, 276 mmol) in anhydrous DMSO (300 mL), and the reaction mixture was then stirred at 90 °C for 48 h. The reaction was cooled to 0 °C, quenched with saturated aqueous NH_4Cl solution (200 mL) and extracted with EtOAc (2 × 300 mL). The

combined organic layers were washed with brine (150 mL), dried ($MgSO_4$), filtered, and concentrated in *vacuo*. Purification of the residue by flash column chromatography (5% EtOAc/petrol) gave the 2-aryl malonate **S3** as a colorless oil (11.7 g, 42%) that displayed spectroscopic data consistent with those reported previously.[2] R_f 0.65 (20% acetone/petrol).

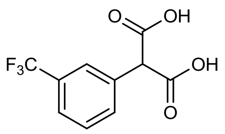

2-[3-(Trifluoromethyl)phenyl]propanedioic acid (S4). A solution of 1,3-diethyl 2-[3-(trifluoromethyl)phenyl]propanedioate **S3** (11.7 g, 38.6 mmol) in Et_2O (40 mL) was added to a stirred solution of 2 M aqueous NaOH (77 mL, 154 mmol) at 0 °C and the resulting mixture was stirred at room temperature for 12 h. The aqueous layer was separated and washed with EtOAc (2 × 100 mL), acidified to pH 2 with 6 M HCl, and extracted with EtOAc (2 × 200 mL). The combined organic extracts were washed with brine (100 mL), dried ($MgSO_4$), filtered, and concentrated in *vacuo* to leave the *malonic acid* **S4** as an off-white solid (8.14 g, 85%) that was used in the next step without further purification.

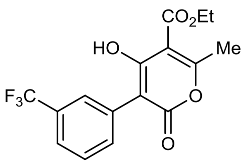

Ethyl 4-hydroxy-6-methyl-2-oxo-3-[3-(trifluoromethyl)phenyl]-2*H*-pyran-5-carboxylate (S5). To a stirred suspension of 2-[3-(trifluoromethyl)phenyl]propanedioic acid **S4** (4.50 g, 18.1 mmol) and DMF (0.14 mL, 1.81 mmol) in toluene (60 mL) was added oxalyl chloride(3.4 mL, 39.9 mmol) dropwise, and the resulting mixture was stirred at room temperature until effervescence had ceased. Ethyl acetoacetate (2.3 mL, 18.1 mmol) was added and the resulting mixture was heated at 120 °C for 12 h. The mixture was concentrated *in vacuo* and the residue was purified by flash column chromatography (14% acetone/petrol) to give the *pyranone* **S5** as a pale brown solid (2.23 g, 36%). R_f 0.35 (20% acetone/petrol); m.p. 80–81 °C (EtOAc/petrol); IR 1716 (C=O), 1670 (C=O), 1558, 1398, 1308, 1288, 1163, 1124, 1102, 1076, 827, 812, 708 cm^{-1}; ^1H NMR (300 MHz, $CDCl_3$) δ 12.24 (1H, s, O**H**), 7.81 (1H, s, Ar**H**), 7.75–7.69 (1H, m, Ar**H**), 7.59–7.47 (2H, m, Ar**H**), 4.46 (2H, q, *J* = 7.1 Hz, OC**H**$_2$), 2.70 (3H, s, C**H**$_3$), 1.42 (3H, t, *J* = 7.1 Hz, OCH_2C**H**$_3$); ^{13}C NMR (100.6 MHz, $CDCl_3$) δ 172.5 (C), 169.2 (C), 165.0 (C), 161.0 (C), 133.8 (CH), 131.5 (C), 130.1 (C, q, *J* = 32.1 Hz), 128.2 (CH), 127.3 (CH, q, *J* = 3.8 Hz), 124.14 (CH, q, *J* = 3.7 Hz), 124.11 (C, q, *J* = 272.4 Hz), 101.8 (C), 101.6 (C), 63.3 (CH_2), 22.6 (CH_3), 13.9 (CH_3); ^{19}F NMR (282.2 MHz, $(CD_3)_2SO$) δ –62.6 (3F, s, CF_3); HRMS (ESI +ve) Exact mass calculated for $C_{16}H_{14}F_3O_5$ $[M+H]^+$: 343.0788, found: 343.0800.

2. Xiaoan, X.; Guorong, C.; Dawei, M. *Org. Lett.* **2005**, *7*, 4693–4695.

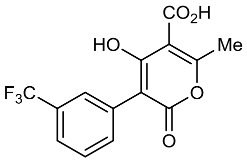

4-Hydroxy-6-methyl-2-oxo-3-[3-(trifluoromethyl)phenyl]-2*H*-pyran-5-carboxylic acid (S6). To a stirred solution of ester **S5** (5.00 g, 14.6 mmol) in toluene (30 mL) was added aqueous 2 M NaOH solution (22 mL, 43.8 mmol) slowly, and the reaction mixture was stirred at 90 °C for 3 h. After cooling to room temperature, the aqueous layer was separated and washed with toluene (50 mL). The aqueous layer was acidified to pH (2–3) using aqueous 10% HCl solution and extracted with EtOAc (2 × 150 mL). The combined organic extracts were washed with water (100 mL), brine (100 mL), dried (Na_2SO_4), and concentrated *in vacuo* to leave a pale brown solid. Purification by trituration (20% acetone/petrol) gave the *carboxylic acid* **S6** as a pale brown solid (3.76 g, 82%). m.p. 194–195 °C (EtOAc/petrol); IR 1696 (C=O), 1558, 1540, 1436, 1307, 1242, 1114, 808, 721 cm^{-1}; ^{1}H NMR (300 MHz, $(CD_3)_2SO$) δ 13.95 (2H, br s, O**H** and COO**H**), 7.84–7.73 (2H, m, Ar**H**), 7.62–7.52 (2H, m, Ar**H**), 2.63 (3H, s, C**H**$_3$); ^{13}C NMR (100.6 MHz, $(CD_3)_2SO$) δ 171.1 (C), 170.2 (C), 168.2 (C), 161.1 (C), 134.5 (CH), 133.6 (C), 128.5 (CH), 128.4 (C, q, *J* = 31.6 Hz), 126.8 (CH, q, *J* = 3.8 Hz), 124.4 (C, q, *J* = 272.4 Hz), 123.1 (CH, q, *J* = 3.5 Hz), 103.2 (C), 99.2 (C), 21.3 (CH_3); ^{19}F NMR (282.2 MHz, $(CD_3)_2SO$) δ –61.1 (3F, s, CF_3); HRMS (ESI +ve) Exact mass calculated for $C_{14}H_{10}F_3O_5$ $[M+H]^+$: 315.0475, found: 315.0469.

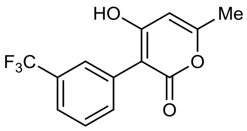

4-Hydroxy-6-methyl-3-[3-(trifluoromethyl)phenyl]-2*H*-pyran-2-one (1b). A stirred suspension of acid **S6** (4.30 g, 13.7 mmol) in Ph_2O (20 mL) was heated at 220 °C for 3 h. The mixture was cooled to room temperature, diluted with petrol, and stirred for 1 h to give a clear suspension, which upon filtration afforded a light brown solid which was purified by trituration (20% acetone/petrol) to give the *pyranone* **1b** as a pale brown solid (3.07 g, 83%). R_f 0.20 (20% acetone/petrol); m.p. 240–241 °C (EtOH); IR 1655 (C=O), 1617, 1558, 1453, 1380, 1316, 1326, 1186, 1163, 1113, 1078, 1001, 803 cm^{-1}; ^{1}H NMR (300 MHz, $(CD_3)_2SO$) δ 11.81 (1H, br s, O**H**), 7.90–7.67 (2H, m, Ar**H**), 7.65–7.46 (2H, m, Ar**H**), 6.18 (1H, s, C**H**=CCH_3), 2.23 (3H, s, C**H**$_3$); ^{13}C NMR (100.6 MHz, $(CD_3)_2SO$) δ 166.2 (C), 163.5 (C), 162.2 (C), 134.6 (CH), 133.7 (C), 128.5 (CH), 128.4 (C, q, *J* = 31.5 Hz), 126.8 (CH, q, *J* = 3.7 Hz), 124.4 (C, q, *J* = 272.4 Hz), 123.2 (CH, q, *J* = 3.7 Hz), 100.2 (CH), 100.1 (C), 19.5 (CH_3); ^{19}F NMR (282.2 MHz, $(CD_3)_2SO$) δ –61.1 (3F, s, CF_3); HRMS (ESI +ve) Exact mass calculated for $C_{13}F_3H_9NaO_3$ $[M+Na]^+$: 293.0396, found: 293.412.

Preparation of Substrate 1c

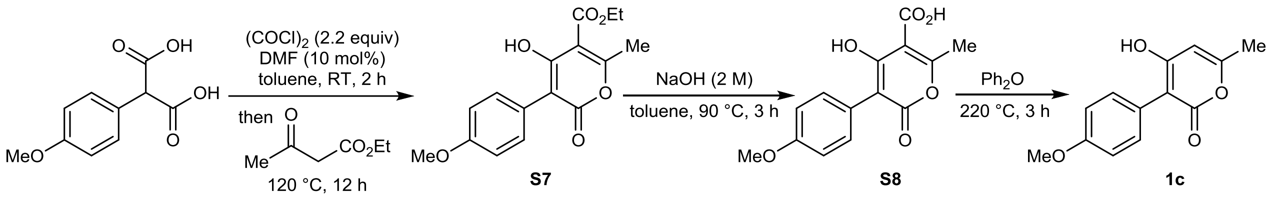

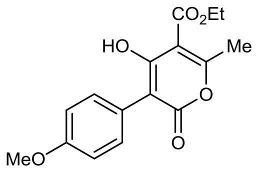

Ethyl 4-hydroxy-3-(4-methoxyphenyl)-6-methyl-2-oxo-2*H*-pyran-5-carboxylate (S7). To a stirred suspension of 2-(4-methoxyphenyl)propanedioic acid[3] (3.00 g, 14.3 mmol) and DMF (0.11 mL, 1.43 mmol) in toluene (40 mL) was added oxalyl chloride (2.7 mL, 31.4 mmol) dropwise, and the resulting mixture was stirred at room temperature until effervescence had ceased. Ethyl acetoacetate (1.82 mL, 14.3 mmol) was added and the resulting mixture was heated at 120 °C for 12 h. The mixture was concentrated *in vacuo* and the residue was purified by flash column chromatography (14% acetone/petrol) to give the *pyranone* **S7** as a pale brown solid (1.83 g, 42%). R_f 0.30 (20% acetone/petrol); m.p. 74–75 °C (EtOAc/petrol); IR 1717 (C=O), 1683 (C=O), 1675, 1558, 1540, 1406, 1289, 1102, 1006, 831, 801 cm^{-1}; ^1H NMR (300 MHz, $CDCl_3$) δ 11.94 (1H, s, O**H**), 7.49–7.42 (2H, m, Ar**H**), 6.99–6.93 (2H, m, Ar**H**), 4.48 (2H, q, *J* = 7.1 Hz, OC**H**$_2$), 3.83 (3H, s, OC**H**$_3$), 2.71 (3H, s, C**H**$_3$), 1.46 (3H, t, *J* = 7.1 Hz, OCH_2C**H**$_3$); ^{13}C NMR (100.6 MHz, $CDCl_3$) δ 171.4 (C), 169.3 (C), 163.9 (C), 161.6 (C), 158.9 (C), 131.6 (2 × CH), 122.7 (C), 113.5 (2 × CH), 103.0 (C), 101.8 (C), 63.0 (CH_2), 55.2 (CH_3), 22.6 (CH_3), 14.0 (CH_3); HRMS (ESI +ve) Exact mass calculated for $C_{16}H_{17}O_6$ $[M+H]^+$: 305.1020, found: 305.1034.

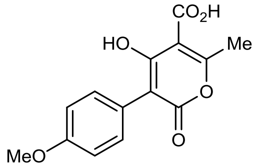

4-Hydroxy-3-(4-methoxyphenyl)-6-methyl-2-oxo-2*H*-pyran-5-carboxylic acid (S8). To a stirred solution of ester **S7** (1.83 g, 6.01 mmol) in toluene (12 mL) was added aqueous 2 M NaOH solution (9.0 mL, 18.0 mmol) slowly, and the reaction mixture was stirred at 90 °C for 3 h. After cooling to room temperature, the aqueous layer was separated and washed with toluene (30 mL). The aqueous layer was acidified to pH (2–3) using aqueous 10% HCl solution and extracted with EtOAc (2 × 50 mL). The combined organic extracts were washed with water (100 mL), brine (100 mL), dried (Na_2SO_4), and concentrated *in vacuo* to leave a pale brown solid. Purification by trituration (20% acetone/petrol) gave the *carboxylic acid* **S8** as a pale brown solid (1.41 g, 85%). m.p. 193–194 °C (EtOAc/petrol); IR 1683 (C=O), 1670, 1652, 1558, 1507, 1436, 1403, 1244, 1183, 1108, 842, 786 cm^{-1}; ^1H NMR (500 MHz, $(CD_3)_2SO$) δ 10.76 (2H, br s, O**H** and COO**H**), 7.36–7.27 (2H, m, Ar**H**),

3. Reddy Chidipudi, S.; Khan, I.; Lam, H. W. *Angew. Chem., Int. Ed.* **2012**, *51*, 12115–12119.

6.92–6.89 (2H, m, Ar**H**), 3.76 (3H, s, OC**H**$_3$), 2.60 (3H, s, C**H**$_3$); ^{13}C NMR (125.8 MHz, $(CD_3)_2SO$) δ 170.4 (C), 170.0 (C), 166.5 (C), 161.4 (C), 157.9 (C), 131.6 (2 × CH), 124.1 (C), 113.0 (2 × CH), 103.1 (C), 100.6 (C), 55.0 (CH_3), 21.0 (CH_3); HRMS (ESI +ve) Exact mass calculated for $C_{14}H_{13}O_6$ $[M+H]^+$: 277.0707, found: 277.0701.

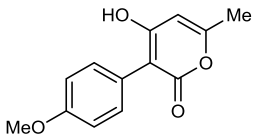

4-Hydroxy-3-(4-methoxyphenyl)-6-methyl-2*H*-pyran-2-one (1c). A stirred suspension of acid **S8** (1.41 g, 5.10 mmol) in Ph_2O (10 mL) was heated at 220 °C for 3 h. The mixture was cooled to room temperature, diluted with petrol, and stirred for 1 h to give a clear suspension, which upon filtration afforded a light brown solid which was purified by trituration (20% acetone/petrol) to give the *pyranone* **1c** as a pale brown solid (971 mg, 83%). R_f 0.20 (20% acetone/petrol); m.p. 205–206 °C (EtOH); IR 1683 (C=O), 1653, 1635, 1617, 1558, 1541, 1516, 1443, 1386, 1377, 1287, 1252, 1179, 1035, 1002, 834, 799 cm^{-1}; ^{1}H NMR (300 MHz, $(CD_3)_2SO$) δ 11.40 (1H, br s, O**H**), 7.33–7.28 (2H, m, Ar**H**), 6.92–6.86 (2H, m, Ar**H**), 6.11 (1H, d, *J* = 0.6 Hz, C**H**=CCH_3), 3.75 (3H, s, OC**H**$_3$), 2.19 (3H, s, C**H**$_3$); ^{13}C NMR (100.6 MHz, $(CD_3)_2SO$) δ 165.2 (C), 163.8 (C), 160.7 (C), 157.8 (C), 131.6 (2 × CH), 124.5 (C), 112.9 (2 × CH), 101.2 (C), 100.3 (CH), 55.0 (CH_3), 19.4 (CH_3); HRMS (ESI +ve) Exact mass calculated for $C_{13}H_{13}O_4$ $[M+H]^+$: 233.0808, found: 233.0807.

4-Hydroxy-6-methyl-3-(4-nitrophenyl)-2*H*-pyran-2-one (1d)

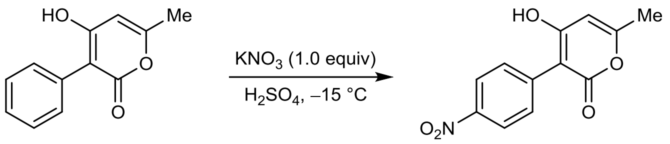

To a stirred suspension of **1a** (2.07 g, 10.2 mmol) in *conc*. H_2SO_4 (40 mL) was added KNO_3 (1.13 g, 11.2 mmol) portionwise at –15 °C over 10 min, and the reaction mixture was stirred for 2 h at the same temperature. The reaction mixture was poured into cold water (200 mL) and extracted with EtOAc (2 × 200 mL). The combined organic layers were washed with water (2 × 100 mL), brine (100 mL), dried (Na_2SO_4), and *concentrated in vacuo* to leave a yellow solid (2.34 g, 93%) which was recrystallized from EtOH to give the *nitroarene* **1d** as a pale yellow solid (1.83 g, 73%). R_f 0.10 (25% acetone/petrol); m.p. 283–284 °C (EtOH); IR 1636 (C=O), 1576, 1518, 1396, 1347, 1289, 1184, 1001, 939, 833, 757 cm^{-1}; ^{1}H NMR (500 MHz, $(CD_3)_2SO$) δ 12.10 (1H, br s, O**H**), 8.22–8.17 (2H, m, Ar**H**), 7.77–7.71 (2H, m, Ar**H**), 6.20 (1H, q, *J* = 0.8 Hz, C**H**=CCH_3), 2.25 (3H, d, *J* = 0.8 Hz, C**H**$_3$); ^{13}C NMR (125.8 MHz, $(CD_3)_2SO$) δ 166.8 (C), 163.0 (C), 162.8 (C), 145.5 (C), 140.1 (C), 131.5 (2 × CH), 122.5 (2 × CH), 100.2 (CH), 99.6 (C), 19.5 (CH_3); HRMS (ESI +ve) Exact mass calculated for $C_{12}H_9NNaO_5$ $[M+Na]^+$: 270.0373, found: 270.0370.

4-Hydroxy-3,6-diphenyl-2*H*-pyran-2-one (1e)

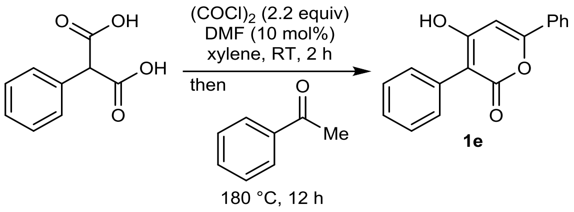

To a stirred suspension of phenylmalonic acid (3.00 g, 16.6 mmol) and DMF (0.13 mL, 1.66 mmol) in xylene (50 mL) was added oxalyl chloride (3.2 mL, 36.6 mmol) dropwise, and the resulting mixture was stirred at room temperature until effervescence had ceased. Acetophenone (1.94 mL, 16.6 mmol) was added and the resulting mixture was heated at 180 °C for 12 h. The mixture was concentrated *in vacuo* and the residue was purified by flash column chromatography (20% acetone/petrol) to give *pyranone* **1e** an off-white solid (1.32 g, 30%). R_f 0.30 (30% acetone/petrol); m.p. 286–287 °C (EtOH); IR 1622 (C=O), 1616, 1558, 1540, 1386, 1332, 1313, 1198, 835, 782, 767 cm^{-1}; ^{1}H NMR (300 MHz, $(CD_3)_2SO$) δ 11.70 (1H, br s, O**H**), 7.85–7.75 (2H, m, Ar**H**), 7.60–7.50 (3H, m, Ar**H**), 7.49–7.43 (2H, m, Ar**H**), 7.37 (1H, t, *J* = 7.5 Hz, Ar**H**), 7.31–7.23 (1H, m, Ar**H**), 6.85 (1H, s, C**H**=CPh); ^{13}C NMR (100.6 MHz, $(CD_3)_2SO$) δ 165.2 (C), 162.9 (C), 158.0 (C), 132.2 (C), 131.0 (C), 130.9 (CH), 130.5 (2 × CH), 129.3 (2 × CH), 127.5 (2 × CH), 126.8 (CH), 125.3 (2 × CH), 103.2 (C), 98.2 (CH); HRMS (ESI +ve) Exact mass calculated for $C_{17}H_{12}NaO_3$ $[M+Na]^+$: 287.0679, found: 287.0684.

4-Hydroxy-5-methyl-3,6-diphenyl-2*H*-pyran-2-one (1f)

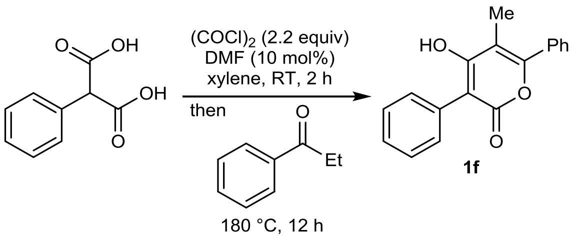

To a stirred suspension of phenylmalonic acid (3.00 g, 16.6 mmol) and DMF (0.13 mL, 1.66 mmol) in xylene (50 mL) was added oxalyl chloride (3.2 mL, 36.6 mmol) dropwise, and the resulting mixture was stirred at room temperature until effervescence had ceased. Propiophenone (2.20 mL, 16.6 mmol) was added and the resulting mixture was heated at 180 °C for 12 h. The mixture was concentrated *in vacuo* and the residue was purified by trituration (15% acetone/petrol) to give the *pyranone* **1f** as an off-white solid (3.17 g, 68%). R_f 0.25 (20% acetone/petrol); m.p. 239–240 °C (EtOH); IR 1662 (C=O), 1559, 1406, 1385, 1184, 1117, 1016, 774 cm^{-1}; ^{1}H NMR (400 MHz, $(CD_3)_2SO$) δ 10.75 (1H, br s, O**H**), 7.65–7.58 (2H, m, Ar**H**), 7.57–7.50 (3H, m, Ar**H**), 7.45–7.37 (4H, m, Ar**H**), 7.36–7.30 (1H, m, Ar**H**), 2.05 (3H, s, C**H**$_3$); ^{13}C NMR (100.6 MHz, $(CD_3)_2SO$) δ

165.0 (C), 162.7 (C), 155.8 (C), 132.5 (C), 132.1 (C), 130.9 (2 × CH), 129.9 (CH), 128.8 (2 × CH), 128.5 (2 × CH), 128.0 (2 × CH), 127.3 (CH), 108.4 (C), 104.6 (C), 11.8 (CH_3); HRMS (ESI +ve) Exact mass calculated for $C_{18}H_{15}O_3$ $[M+H]^+$: 279.1016, found: 279.1025.

4-Hydroxy-2(5*H*)-furanone (1g)

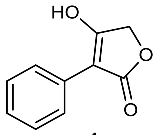

1g

Substrate **1g** was synthesized according to a literature procedure.[4]

Preparation of Substrate 1h

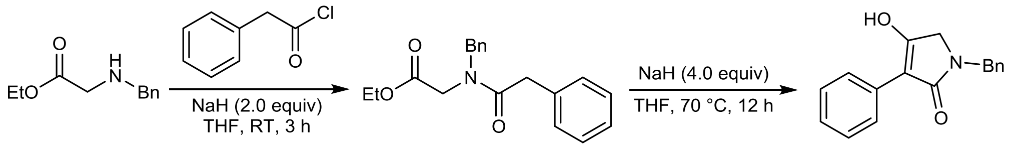

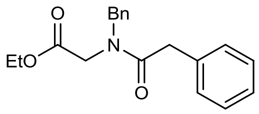

Ethyl 2-(*N*-benzyl-2-phenylacetamido)acetate (S10). To a suspension of NaH (60% in mineral oil, 1.99 g, 52.0 mmol) in THF (50 mL) at 0 °C was added a solution of **S9**[5] (5.02 g, 26.0 mmol) in THF (25 mL), and the mixture was stirred for 30 min. Phenylacetyl chloride (3.4 mL, 26.0 mmol) was added slowly and the mixture was stirred at room temperature for 3 h. The reaction was quenched carefully with 10% aqueous HCl solution and the aqueous layer was separated and extracted with CH_2Cl_2 (2 × 50 mL). The combined organic layers were dried ($MgSO_4$), filtered, and concentrated *in vacuo*. Purification of the residue by flash column chromatography (10% acetone/petrol) gave the *amide* **S10** as a 1:0.41 mixture of rotamers (by ^{1}H NMR analysis) as a colorless viscous oil (5.50 g, 68%). R_f 0.53 (30% acetone/petrol); IR 1744 (C=O), 1651 (C=O), 1602, 1496, 1374, 1196, 1028, 732 cm^{-1}; HRMS (ESI +ve) Exact mass calculated for $C_{19}H_{21}NNaO_3$ $[M+Na]^+$: 334.1414, found: 334.1414.

Major rotamer: ^{1}H NMR (400 MHz, $CDCl_3$) δ 7.39–7.20 (8H, m, Ar**H**), 7.11 (2H, d, *J* = 6.6 Hz, Ar**H**), 4.62 (2H, s, NC**H**$_2$Ph), 4.19 (2H, q, *J* = 7.2 Hz, OC**H**$_2$), 4.07 (2H, s, C**H**$_2$Ph), 3.85 (2H, s, NC**H**$_2$C=O), 1.29–1.19 (3H, m, OCH$_2$C**H**$_3$); ^{13}C NMR (100.6 MHz, $CDCl_3$) δ 171.8 (C), 169.1 (C), 135.8 (C), 134.5 (C), 128.9 (2 × CH), 128.7 (2 × CH), 128.6 (2 × CH), 127.8 (CH), 126.8 (CH),

4. Weber, V.; Rubat, C.; Duroux, E.; Lartigue, C.; Madesclairea, M.; Coudert, P. *Bioorg. Med. Chem.* **2005**, *13*, 4552–4564.
5. Prepared according to: Kruijtzer, J. A. W.; Hofmeyer, L. J. F.; Heerma, W.; Versluis, C.; Liskamp, R. M. J. *Chem. Eur. J.* **1998**, *4*, 1570–1580.

126.7 (2 × CH), 61.1 (CH_2), 52.4 (CH_2), 47.1 (CH_2), 40.6 (CH_2), 14.1 (CH_3).

Minor rotamer: ^{1}H NMR (400 MHz, $CDCl_3$) δ 7.39–7.20 (10H, m, Ar**H**), 4.70 (2H, s, NC**H**$_2$Ph), 4.12 (2H, q, *J* = 7.2 Hz, OC**H**$_2$), 3.93 (2H, s, C**H**$_2$Ph), 3.75 (2H, s, NC**H**$_2$C=O), 1.29–1.19 (3H, m, OCH_2C**H**$_3$); ^{13}C NMR (100.6 MHz, $CDCl_3$) δ 171.4 (C), 168.9 (C), 136.6 (C), 134.4 (C), 128.8 (2 × CH), 128.5 (2 × CH), 128.4 (2 × CH), 127.6 (CH), 126.9 (CH), 109.9 (CH), 61.5 (CH_2), 49.7 (CH_2), 48.6 (CH_2), 41.0 (CH_2), 14.0 (CH_3).

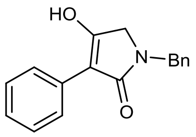

1-Benzyl-3-phenylpyrrolidine-2,4-dione (1h). To a suspension of NaH (60% in mineral oil, 1.38 g, 36.0 mmol) in THF (20 mL) at 0 °C was added a solution of **S10** (2.80 g, 9.00 mmol) in THF (10 mL). The mixture was stirred for 10 min and then heated at 70 °C for 12 h. The reaction was quenched carefully with 10% aqueous HCl solution and the aqueous layer was separated and extracted with CH_2Cl_2 (2 × 25 mL). The combined organic layers were dried ($MgSO_4$), filtered, and concentrated *in vacuo*. Purification of the residue by flash column chromatography (10% acetone/petrol) gave the *lactam* **1h** as a white amorphous solid (1.93 g, 81%). R_f 0.23 (30% acetone/petrol); m.p. 205–206 °C (20% acetone/petrol); IR 1665 (C=O), 1579 (C=O), 1450, 1385, 1313, 1218, 974, 782 cm^{-1}; ^{1}H NMR (400 MHz, $(CD_3)_2SO$) δ 11.67 (1H, s, O**H**), 8.02 (2H, d, *J* = 7.4 Hz, Ar**H**), 7.40–7.30 (4H, m, Ar**H**), 7.29–7.20 (3H, m, Ar**H**), 7.17 (1H, t, *J* = 7.4 Hz, Ar**H**), 4.56 (2H, s, NC**H**$_2$Ph), 3.85 (2H, s, $PhCH_2NC$**H**$_2$); ^{13}C NMR (100.6 MHz, $(CD_3)_2SO$) δ 171.1 (C), 167.0 (C), 138.1 (C), 132.3 (C), 128.6 (2 × CH), 127.7 (2 × CH), 127.5 (2 × CH), 127.1 (CH), 126.7 (2 × CH), 125.6 (C), 102.9 (CH), 48.7 (CH_2), 44.5 (CH_2); HRMS (ESI +ve) Exact mass calculated for $C_{17}H_{15}NNaO_2$ $[M+Na]^+$: 288.0995, found: 288.0995.

Preparation of Substrate 1i

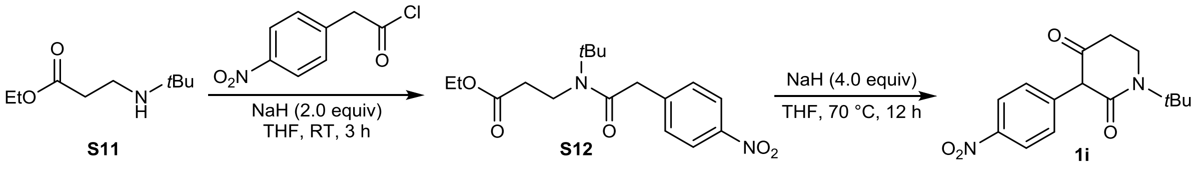

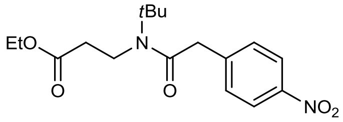

Ethyl 3-[*N*-*tert*-butyl-2-(4-nitrophenyl)acetamido]propanoate (S12). To a suspension of NaH (60% in mineral oil, 2.30 g, 60.0 mmol) in THF (50 mL) at 0 °C was added a solution of **S11**[6] (5.19 g, 30.0 mmol) in THF (25 mL), and the mixture was stirred for 30 min. 4-Nitrophenylacetyl chloride[7] (5.97

6. Prepared according to: Kachkovskyi, G.; Faderl, C.; Reisera, O. *Adv. Synth. Catal.* **2013**, *355*, 2240–2248.
7. Prepared according to: Player, M. R.; Layer, M. R.; Parsons, W. H.; Huang, H.; Hutta, D. A.; Hu, H.; Rinker, J. *US Patent*, 2008/114007 A1, **2008**.

g, 30.0 mmol) was added slowly and the mixture was stirred at room temperature for 3 h. The reaction was quenched carefully with 10% aqueous HCl solution (10%) and the aqueous layer was separated and washed with CH_2Cl_2 (2 × 50 mL). The combined organic layers were dried ($MgSO_4$), filtered, and concentrated *in vacuo*. Purification of the residue by flash column chromatography (10% acetone/petrol) gave **S12** as a yellow viscous oil (6.25 g, 62%). R_f 0.44 (30% acetone/petrol); IR 1730 (C=O), 1643 (C=O), 1606, 1510, 1397, 1343, 1211, 1225, 1053, 858 cm^{-1}; ^{1}H NMR (400 MHz, $CDCl_3$) δ 8.21–8.16 (2H, m, Ar**H**), 7.44–7.42 (2H, m, Ar**H**), 4.17 (2H, q, *J* = 7.1 Hz, OC**H**$_2$), 3.83 (2H, s, ArC**H**$_2$C=O), 3.68–3.61 (2H, m, NC**H**$_2$CH$_2$), 2.60–2.54 (2H, m, CH$_2$C**H**$_2$C=O), 1.45 (9H, s, C(C**H**$_3$)$_3$), 1.28 (3H, t, *J* = 7.1 Hz, OCH$_2$C**H**$_3$); ^{13}C NMR (100.6 MHz, $CDCl_3$) δ 170.7 (C), 170.1 (C), 146.9 (C), 143.3 (C), 130.0 (2 × CH), 123.7 (2 × CH), 61.0 (CH$_2$), 57.7 (C), 43.1 (CH$_2$), 41.2 (CH$_2$), 36.7 (CH$_2$), 28.8 (3 × CH$_3$), 14.1 (CH$_3$); HRMS (ESI +ve) Exact mass calculated for $C_{17}H_{24}N_2NaO_5$ [M+Na]$^+$: 359.1577, found: 359.1579.

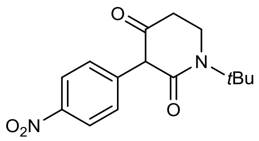

1-*tert*-Butyl-3-(4-nitrophenyl)piperidine-2,4-dione (1i). To a suspension of NaH (60% in mineral oil, 1.19 g, 31.0 mmol) in THF (20 mL) at 0 °C was added a solution of **S12** (2.60 g, 7.70 mmol) in THF (10 mL). The mixture was stirred for 10 min and then heated at 70 °C for 12 h. The reaction was quenched carefully with 10% aqueous HCl solution and the aqueous layer was separated and extracted with CH_2Cl_2 (2 × 25 mL). The combined organic layers were dried ($MgSO_4$), filtered, and concentrated *in vacuo*. Purification of the residue by flash column chromatography (10% acetone/petrol) gave the *lactam* **1i** as a yellow amorphous solid (1.15 g, 51%). R_f 0.43 (30% acetone/petrol); m.p. 159–160 °C (20% acetone/petrol); IR 1719 (C=O), 1648 (C=O), 1522, 1412, 1346, 1298, 1010, 845 cm^{-1}; ^{1}H NMR (400 MHz, $CDCl_3$) δ 8.26–8.20 (2H, m, Ar**H**), 7.36–7.30 (2H, m, Ar**H**), 4.74 (1H, s, C**H**C=O), 3.89–3.70 (2H, m, NC**H**$_2$CH$_2$), 2.81–2.58 (2H, m, CH$_2$C**H**$_2$C=O), 1.51 (9H, s, C(C**H**$_3$)$_3$); ^{13}C NMR (100.6 MHz, $CDCl_3$) δ 202.6 (C), 166.0 (C), 147.4 (C), 139.7 (C), 131.1 (2 × CH), 123.5 (2 × CH), 66.4 (CH), 58.7 (C), 39.2 (CH$_2$), 38.6 (CH$_2$), 28.7 (3 × CH$_3$); HRMS (ESI +ve) Exact mass calculated for $C_{15}H_{19}N_2O_4$ [M+H]$^+$: 291.1339, found: 291.1338.

Preparation of Substrate 1j

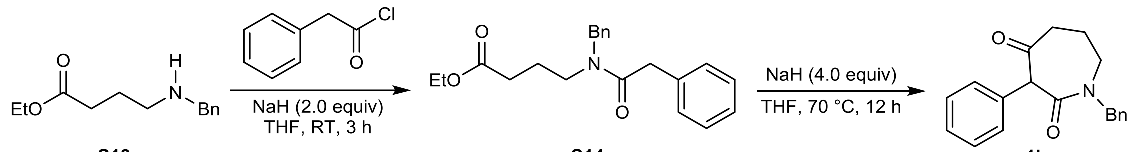

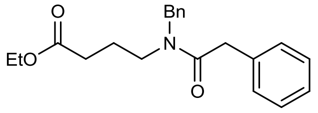

Ethyl 4-(*N*-benzyl-2-phenylacetamido)butanoate (S14). To a suspension of NaH (60% in mineral oil, 1.92 g, 50.0 mmol) in THF (50 mL) at 0 °C was added a solution of **S13**[8] (5.52 g, 25.0 mmol) in THF (25 mL), and the mixture was stirred for 30 min. Phenylacetyl chloride (3.30 mL, 25.0 mmol) was added slowly slowly and the mixture was stirred at room temperature for 3 h. The reaction was quenched carefully with HCl (10%) and the aqueous layer was separated and extracted with CH_2Cl_2 (2 × 50 mL). The combined organic layers were dried ($MgSO_4$), filtered, and concentrated *in vacuo*. Purification of the residue by flash column chromatography (8% acetone/petrol) gave the *amide* **S14** as a 1:0.9 mixture of rotamers (by ^{1}H NMR analysis) as a colorless viscous oil (4.60 g, 54%). R_f 0.50 (30% acetone/petrol); IR 1728 (C=O), 1644 (C=O), 1601, 1496, 1453, 1373, 1150, 1030, 731 cm^{-1}; HRMS (ESI +ve) Exact mass calculated for $C_{21}H_{25}NNaO_3$ [M+Na]$^+$: 362.1727, found: 362.1729.

Major rotamer: ^{1}H NMR (400 MHz, $CDCl_3$) δ 7.38–7.19 (8H, m, Ar**H**), 7.12 (2H, d, *J* = 7.0 Hz, Ar**H**), 4.53 (2H, s, NC**H**$_2$Ph), 4.17–4.04 (2H, m, OC**H**$_2$), 3.70 (2H, s, C**H**$_2$Ph), 3.46–3.37 (2H, m, NC**H**$_2$CH$_2$), 2.29 (2H, t, *J* = 7.4 Hz, CH$_2$C**H**$_2$C=O), 1.94–1.75 (2H, m, CH$_2$C**H**$_2$CH$_2$), 1.29–1.18 (3H, m, OCH$_2$C**H**$_3$); ^{13}C NMR (100.6 MHz, $CDCl_3$) δ 172.8 (C), 171.3 (C), 136.5 (C), 134.8 (C), 128.7 (2 × CH), 128.51 (2 × CH), 128.46 (CH), 127.8 (2 × CH), 127.4 (CH), 126.1 (2 × CH), 60.1 (CH_2), 51.1 (CH_2), 45.2 (CH_2), 40.8 (CH_2), 31.3 (CH_2), 22.5 (CH_2), 13.99 (CH_3).

Minor rotamer: ^{1}H NMR (400 MHz, $CDCl_3$) δ 7.38–7.19 (10H, m, Ar**H**), 4.63 (2H, s, NC**H**$_2$Ph), 4.17–4.04 (2H, m, OC**H**$_2$CH$_3$), 3.83 (2H, s, C**H**$_2$Ph), 3.30–3.19 (2H, m, NC**H**$_2$CH$_2$), 2.22 (2H, t, *J* = 6.9 Hz, CH$_2$C**H**$_2$C=O), 1.94–1.75 (2H, m, CH$_2$C**H**$_2$CH$_2$), 1.29–1.18 (3H, m, OCH$_2$C**H**$_3$); ^{13}C NMR (100.6 MHz, $CDCl_3$) δ 172.4 (C), 170.9 (C), 137.4 (C), 135.1 (C), 128.6 (2 × CH), 128.5 (CH), 128.4 (2 × CH), 128.3 (2 × CH), 127.1 (CH), 126.6 (2 × CH), 60.4 (CH_2), 47.8 (CH_2), 46.2 (CH_2), 40.5 (CH_2), 30.7 (CH_2), 23.2 (CH_2), 14.0 (CH_3).

8. Prepared according to: Ram, S.; Spicer, L. D. *Tetrahedron*, **1987**, *28*, 515–516.

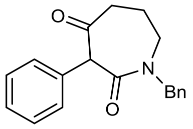

1-Benzyl-3-phenylazepane-2,4-dione (1j). To a suspension of NaH (60% in mineral oil, 1.25 g, 28.0 mmol) in THF (20 mL) at 0 °C was added a solution of **S14** (2.37 g, 7.00 mmol) in THF (10 mL). The mixture was stirred for 10 min and then heated at 70 °C for 12 h. The reaction was quenched carefully with 10% aqueous HCl solution and the aqueous layer was separated and extracted with CH_2Cl_2 (2 × 25 mL). The combined organic layers were dried ($MgSO_4$), filtered, and concentrated *in vacuo*. Purification of the residue by flash column chromatography (10% acetone/petrol) gave the *lactam* **1j** as an off-white amorphous solid (1.40 g, 68%). R_f 0.48 (30% acetone/petrol); m.p. 142–143 °C (20% acetone/petrol); IR 1718 (C=O), 1636 (C=O), 1605, 1478, 1249, 951, 748 cm^{-1}; ^{1}H NMR (400 MHz, $(CD_3)_2SO$) δ 7.50–7.15 (10H, m, Ar**H**), 5.62 (1H, s, C**H**C=O), 4.70 (1H, d, J = 14.6 Hz, NC**H**$_2$Ph), 4.50 (1H, d, J = 14.6 Hz, NC**H**$_2$Ph), 4.32–4.04 (1H, m, NC**H**$_2$CH$_2$), 3.43 (1H, d, J = 15.0 Hz, NC**H**$_2$CH$_2$), 2.86 (1H, dd, J = 17.9, 10.5 Hz, C**H**$_2$C=O), 2.05–1.90 (1H, m, C**H**$_2$C=O), 1.85–1.65 (1H, m, CH$_2$C**H**$_2$CH$_2$), 2.46-2.40 (1H, m, CH$_2$C**H**$_2$CH$_2$); ^{13}C NMR (100.6 MHz, $(CD_3)_2SO$) δ 204.1 (C), 166.9 (C), 138.0 (C), 134.4 (C), 130.9 (2 × CH), 128.5 (2 × CH), 127.9 (2 × CH), 127.3 (CH), 127.2 (2 × CH), 126.9 (CH), 62.2 (CH), 50.6 (CH$_2$), 46.2 (CH$_2$), 43.0 (CH$_2$), 27.1 (CH$_2$); HRMS (ESI) Exact mass calculated for $C_{19}H_{19}NNaO_2$ $[M+Na]^+$: 316.1308, found: 316.1298.

1-Benzyl-4-hydroxy-5,7-dimethyl-3-phenyl-1,2-dihydroquinolin-2-one (1k)

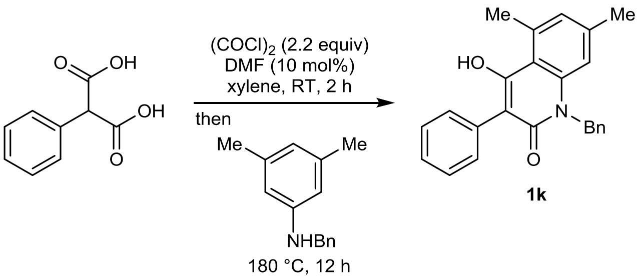

To a stirred suspension of phenylmalonic acid (5.00 g, 27.7 mmol) and DMF (0.21 mL, 2.77 mmol) in xylene (75 mL) was added oxalyl chloride (5.3 mL, 61.0 mmol) dropwise, and the resulting mixture was stirred at room temperature until effervescence had ceased. *N*-Benzyl-3,5-dimethylaniline[9] (5.8 mL, 27.7 mmol) was added and the resulting mixture was heated at 180 °C for 12 h. The mixture was concentrated *in vacuo* and the residue was purified by trituration (15% acetone/petrol) to give the *lactam* **1k** as an off-white solid (8.61 g, 87%). R_f 0.30 (30% acetone/petrol); m.p. 243–244 °C (EtOH); IR 1609 (C=O), 1565, 1493, 1443, 1301, 1260, 1186, 1151, 831, 755, 703 cm^{-1}; ^{1}H NMR (400 MHz, $(CD_3)_2SO$) δ 9.63 (1H, br s, O**H**), 7.47–7.30 (7H, m, Ar**H**), 7.26–7.17 (3H, m, Ar**H**), 7.04 (1H, s, Ar**H**), 6.83 (1H, m, Ar**H**), 5.49 (2H, s, NC**H**$_2$), 2.71

9. Prepared according to: Yao-Bing, H.; Chu-Ting, Y.; Jun, Y,; Xiao-Jian, D., Yao, F., Lei, L. *J. Org. Chem.* **2011**, *76*, 800–810.

(3H, s, C**H**$_3$), 2.25 (3H, s, C**H**$_3$); ^{13}C NMR (100.6 MHz, $(CD_3)_2SO$) δ 161.8 (C), 159.5 (C), 139.9 (C), 139.8 (C), 138 (C), 137.1 (C), 133.5 (C), 131.6 (2 × CH), 128.6 (2 × CH), 128.1 (2 × CH), 127.2 (CH), 126.9 (CH), 126.8 (CH), 126.5 (2 × CH), 113.43 (CH), 113.35 (C), 111.5 (C), 45.3 (CH_2), 24.6 (CH_3), 21.3 (CH_3); HRMS (ESI +ve) Exact mass calculated for $C_{24}H_{22}NO_2$ $[M+H]^+$: 356.1645, found: 356.1646.

5,7-Dimethyl-3-phenyl-3,4-dihydro-2*H*-1-benzopyran-2,4-dione (1l)

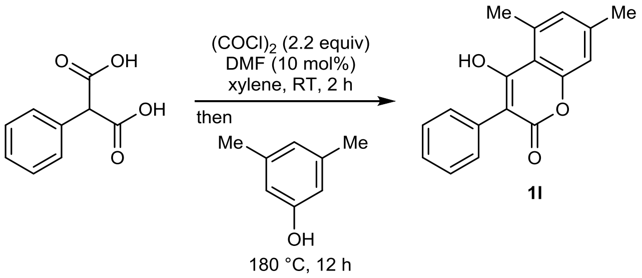

To a stirred suspension of phenylmalonic acid (5.00 g, 27.7 mmol) and DMF (0.21 mL, 2.77 mmol) in xylene (75 mL) was added oxalyl chloride (5.25 mL, 61.0 mmol) dropwise, and the resulting mixture was stirred at room temperature until effervescence had ceased. 3,5-Dimethylphenol (3.4 mL, 27.8 mmol) was added and the resulting mixture was heated at 180 °C for 12 h. The mixture was concentrated *in vacuo* to and the residue was purified by trituration (20% acetone/petrol) to give the *pyranone* **1l** as an off-white solid (5.20 g, 70%). R_f 0.30 (20% acetone/petrol); m.p. 273–274 °C (EtOH); IR 1717 (C=O), 1611, 1597, 1118, 1083, 853, 778, 734 cm^{-1}; ^{1}H NMR (400 MHz, $(CD_3)_2SO$) δ 11.01 (1H, br s, O**H**), 7.46–7.39 (2H, m, Ar**H**), 7.38–7.31 (3H, m, Ar**H**), 7.07–7.03 (1H, m, Ar**H**), 7.00–6.93 (1H, m, Ar**H**), 2.66 (3H, s, C**H**$_3$), 2.36 (3H, s, C**H**$_3$); ^{13}C NMR (100.6 MHz, $(CD_3)_2SO$) δ 163.3 (C), 161.5 (C), 153.8 (C), 141.8 (C), 137.0 (C), 131.9 (C), 131.3 (2 × CH), 128.6 (CH), 128.4 (2 × CH), 127.6 (CH), 114.6 (CH), 112.7 (C), 105.3 (C), 23.2 (CH_3), 20.8 (CH_3); HRMS (ESI +ve) Exact mass calculated for $C_{17}H_{14}NaO_3$ $[M+Na]^+$: 289.0835, found: 289.0820.

4-Hydroxy-3-phenyl-5,6,7,8-tetrahydro-2*H*-chromen-2-one (1m)

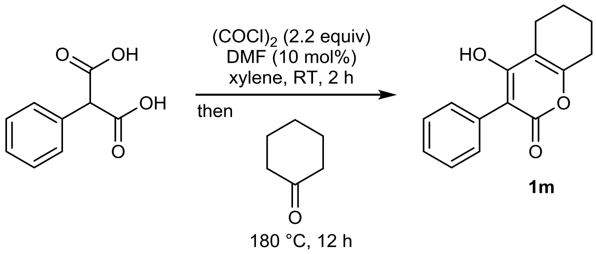

To a stirred suspension of phenylmalonic acid (5.00 g, 27.7 mmol) in xylene (75 mL) and DMF

(0.21 mL, 2.77 mmol) was added oxalyl chloride (5.25 mL, 61.0 mmol) dropwise, and the resulting mixture was stirred at room temperature until effervescence had ceased. Cyclohexanone (4.3 mL, 41.6 mmol) was added and the resulting mixture was heated at 180 °C for 12 h. The mixture was concentrated *in vacuo* to and the residue was purified by trituration (15% acetone/petrol) to give the *pyranone* **1m** as an off-white solid (3.66 g, 55%). R_f 0.30 (30% acetone/petrol); m.p. 152–153 °C (EtOH); IR 1683 (C=O), 1673, 1653, 1559, 1541, 1436, 1237, 1132, 1095, 773, 758 cm^{-1}; ^1H NMR (400 MHz, $(CD_3)_2SO$) δ 10.51 (1H, br s, O**H**), 7.42–7.35 (2H, m, Ar**H**), 7.35–7.27 (3H, m, Ar**H**), 2.54–2.44 (2H, m, =CC**H**$_2$), 2.40–2.34 (2H, m, =CC**H**$_2$), 1.85–1.60 (4H, m, CH_2C**H**$_2$C**H**$_2$$CH_2$); ^{13}C NMR (100.6 MHz, $(CD_3)_2SO$) δ 164.4 (C), 163.0 (C), 158.5 (C), 132.4 (C), 130.9 (2 × CH), 127.9 (2 × CH), 127.0 (CH), 108.6 (C), 103.4 (C), 26.9 (CH_2), 21.2 (2 × CH_2), 20.9 (CH_2); HRMS (ESI +ve) Exact mass calculated for $C_{15}H_{14}NaO_3$ $[M+Na]^+$: 265.0835, found: 265.0841.

4,4-Dimethyl-2-phenylcyclohexane-1,3-dione (1n)

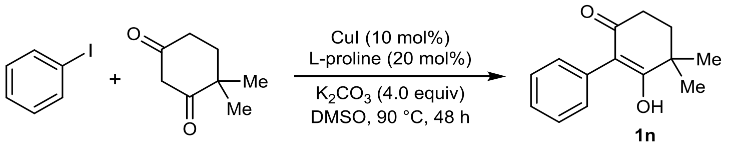

Iodobenzene (1.37 mL, 12.3 mmol) was added to a stirred solution of CuI (234 mg, 1.22 mmol), L-proline (282 mg, 2.45 mmol), K_2CO_3 (6.77 g, 49.0 mmol), and dimedone (5.15 g, 35.7 mmol) in anhydrous DMSO (50 mL), and the mixture was stirred at 90 °C for 48 h. The reaction was cooled to 0 °C, quenched with 10% aqueous HCl solution (100 mL) and extracted with EtOAc (2 × 100 mL). The combined organic extracts were washed with brine (50 mL), dried ($MgSO_4$), filtered, and concentrated *in vacuo*. Purification of the residue by flash column chromatography (15% acetone/hexane) gave the *2-aryl-cyclic 1,3-dicarbonyl* **1n** as an off-white solid (1.43 g, 54%). R_f 0.20 (20% acetone/petrol); m.p. 163–164 °C (EtOH); IR 1605 (C=O), 1594, 1374, 1355, 1308, 1268, 980, 754, 705 cm^{-1}; ^1H NMR (400 MHz, $(CD_3)_2SO$) δ 10.42 (1H, br s, O**H**), 7.28–7.23 (2H, m, Ar**H**), 7.19–7.13 (1H, m, Ar**H**), 7.08–7.04 (2H, m, Ar**H**), 2.61 (2H, t, J = 5.7 Hz, C**H**$_2$C=O), 1.82 (2H, t, J = 6.4 Hz, C**H**$_2$$CH_2$C=O), 1.07 (6H, s, C(C**H**$_3$)$_2$); ^{13}C NMR (100.6 MHz, $(CD_3)_2SO$) δ 200.6 (C), 170.1 (C), 134.8 (C), 131.0 (2 × CH), 127.1 (2 × CH), 125.7 (CH), 114.5 (C), 39.2 (C), 33.6 (CH_2), 26.3 (CH_2), 24.9 (2 × CH_3); HRMS (ESI +ve) Exact mass calculated for $C_{14}H_{16}NaO_2$ $[M+Na]^+$: 239.1043, found: 239.1040.

Preparation of Substrates 1o and 1p

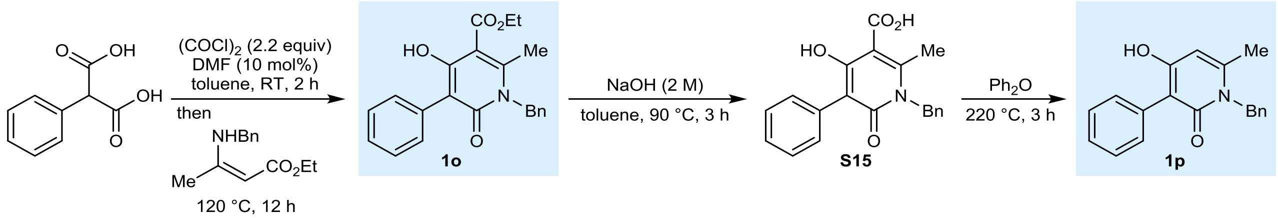

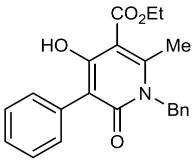

Ethyl 1-benzyl-4-hydroxy-2-methyl-6-oxo-5-phenyl-1,6-dihydropyridine-3-carboxylate (1o). To a stirred suspension of phenylmalonic acid (15.0 g, 83.3 mmol) and DMF (0.64 mL, 8.32 mmol) in toluene (250 mL) was added oxalyl chloride (15.7 mL, 183 mmol) dropwise, and the resulting mixture was stirred at room temperature until effervescence had ceased. Ethyl (*Z*)-3-(benzylamino)but-2-enoate[10] (18.2 mL, 83.3 mmol) was added and the resulting mixture was heated at 120 °C for 12 h. The mixture was concentrated *in vacuo* and the residue was purified by flash column chromatography (15% acetone/petrol) to give the *lactam* **1o** as a pale brown solid (23.2 g, 77%). R_f 0.30 (20% acetone/petrol); m.p. 105–106 °C (EtOAc/petrol); IR 1658 (C=O), 1630, 1534, 1423, 1373, 1284, 1209, 1103, 1010, 824, 770, 746 cm^{-1}; ^{1}H NMR (300 MHz, $CDCl_3$) δ 11.53 (1H, s, O**H**), 7.57–7.50 (2H, m, Ar**H**), 7.46–7.39 (2H, m, Ar**H**), 7.38–7.25 (4H, m, Ar**H**), 7.23–7.17 (2H, m, Ar**H**), 5.52 (2H, br s, NC**H**$_2$), 4.42 (2H, q, *J* = 7.1 Hz, OC**H**$_2$), 2.70 (3H, s, C**H**$_3$), 1.40 (3H, t, *J* = 7.1 Hz, OCH_2C**H**$_3$); ^{13}C NMR (100.6 MHz, $CDCl_3$) δ 170.1 (C), 162.7 (C), 162.1 (C), 154.3 (C), 136.2 (C), 132.7 (C), 130.7 (2 × CH), 128.9 (2 × CH), 127.8 (2 × CH), 127.5 (CH), 127.2 (CH), 126.4 (2 × CH), 110.0 (C), 100.7 (C), 62.4 (CH_2), 48.1 (CH_2), 20.0 (CH_3), 14.1 (CH_3); HRMS (ESI +ve) Exact mass calculated for $C_{22}H_{22}NO_4$ $[M+H]^+$: 364.1543, found: 364.1545.

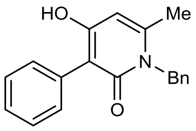

1-Benzyl-4-hydroxy-6-methyl-3-phenyl-1,2-dihydropyridin-2-one (1p). To a stirred solution of ester **1o** (7.00 g, 19.3 mmol) in toluene (80 mL) was added aqueous 2 M NaOH solution (28.9 mL, 57.8 mmol) slowly, and the reaction mixture was stirred at 90 °C for 3 h. After cooling to room temperature, the aqueous layer was separated and washed with toluene (50 mL). The aqueous layer was acidified to pH (2–3) using aqueous 10% HCl solution and extracted with EtOAc (2 × 100 mL). The combined organic extracts were washed with water (100 mL), brine (100 mL), dried (Na_2SO_4), and evaporated under reduced pressure to leave the *carboxylic acid* **S15** as a pale brown solid which was used immediately in the

10. Prepared according to: (a) Rimoli, M.G.; Avallone, L.; Zanarone, S.; Abignente, E.; Mangoni, A. *J. Heterocycl. Chem.* **2002**, *39*, 1117–1122. For spectroscopic data, see: (b) Xin, D.; Burgess, K. *Org. Lett.* **2014**, *16*, 2108–2110.

next step without further purification.

Ph_2O (80 mL) was added to the acid obtained above and the solution was heated at 220 °C for 3 h. The mixture was cooled to room temperature, diluted with petrol, and stirred for 1 h which upon filtration afforded a light brown solid which was purification by trituration (20% acetone/petrol) to give the *lactam* **1p** as a pale brown solid (3.97 g, 71%). R_f 0.20 (20% acetone/petrol); m.p. 212–213 °C (EtOH); IR 1558 (C=O), 1433, 1398, 1379, 1361, 1346, 1232, 1150, 1089, 945, 838, 781, 731 cm^{-1}; ^{1}H NMR (400 MHz, $(CD_3)_2SO$) δ 10.32 (1H, br s, O**H**), 7.42–7.37 (2H, m, Ar**H**), 7.36–7.28 (4H, m, Ar**H**), 7.27–7.22 (1H, m, Ar**H**), 7.22–7.12 (3H, m, Ar**H**), 5.98 (1H, s, C**H**=CCH_3), 5.26 (2H, s, $NC\mathbf{H}_2$), 5.98 (3H, s, $C\mathbf{H}_3$); ^{13}C NMR (100.6 MHz, $(CD_3)_2SO$) δ 163.1 (C), 161.5 (C), 145.8 (C), 137.8 (C), 134.5 (C), 130.9 (2 × CH), 128.6 (2 × CH), 127.1 (2 × CH), 126.9 (CH), 126.2 (2 × CH), 125.9 (CH), 108.4 (C), 100.0 (CH), 46.0 (CH_2), 20.1 (CH_3); HRMS (ESI +ve) Exact mass calculated for $C_{19}H_{18}NO_2$ $[M+H]^+$: 292.1332, found: 292.1325.

1-Benzyl-4-hydroxy-3-phenyl-1,2,5,6,7,8-hexahydroquinolin-2-one (1q)

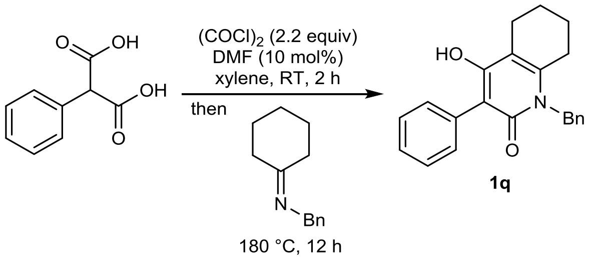

To a stirred suspension of phenylmalonic acid (5.00 g, 27.7 mmol) in xylene (75 mL) and DMF (0.21 mL, 2.77 mmol) was added oxalyl chloride (5.25 mL, 61.0 mmol) dropwise, and the resulting mixture was stirred at room temperature until effervescence had ceased. Freshly prepared *N*-benzylcyclohexanimine[11] (7.80 g, 41.6 mmol) was added and the resulting mixture was heated at 180 °C for 12 h. The mixture was concentrated *in vacuo* and the residue was purified by trituration (10% acetone/petrol) to give the *lactam* **1q** as an off-white solid (2.62 g, 28%). R_f 0.30 (20% acetone/petrol); m.p. 183–184 °C (EtOH); IR 1634 (C=O), 1558, 1541, 1507, 1457, 1253, 1200, 1178, 780, 744 cm^{-1}; ^{1}H NMR (400 MHz, $(CD_3)_2SO$) δ 9.20 (1H, br s, O**H**), 7.42–7.29 (6H, m, Ar**H**), 7.28–7.19 (2H, m, Ar**H**), 7.13 (2H, d, *J* = 7.4 Hz, Ar**H**), 5.27 (2H, br s, $NC\mathbf{H}_2$), 2.62–2.51 (2H, m, 2 × $=CC\mathbf{H}_2$), 2.47–2.36 (2H, m, 2 × $=CC\mathbf{H}_2$), 1.70–1.53 (4H, m, $CH_2C\mathbf{H}_2C\mathbf{H}_2CH_2$); ^{13}C NMR (100.6 MHz, $(CD_3)_2SO$) δ 162.1 (C), 160.3 (C), 143.1 (C), 138.0 (C), 134.3 (C), 131.2 (2 × CH), 128.6 (2 × CH), 127.6 (2 × CH), 126.8 (CH), 126.4 (CH), 126.1 (2 × CH), 109.8 (C), 107.7 (C), 45.3 (CH_2), 26.4 (CH_2), 22.1 (CH_2), 21.9 (CH_2), 21.1 (CH_2); HRMS (ESI +ve) Exact mass

11. Prepared according to: (a) Armesto, D.; Esteban, S.; Horspool, W.; Martin, J.; Martinez-Alcazar, P.; Perez-Ossorio, R. *J. Chem. Soc., Perkin Trans. 1* **1989**, 751–755. For spectroscopic data, see: (b) Faiza, D., Eva, R.; Josep, B. *Org. Lett.* **2007**, *9*, 2633–2636.

calculated for $C_{22}H_{22}NO_2$ $[M+H]^+$: 332.1645, found: 332.1655.

1-Methyl-5-phenyl-1,3-diazinane-2,4,6-trione (1r)[12]

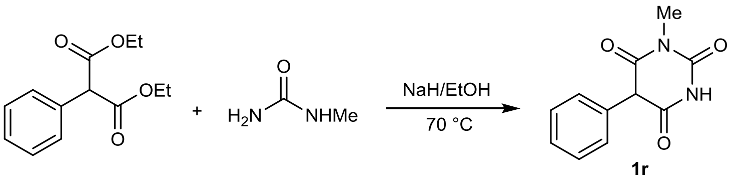

Substrate **1r** was prepared according to a literature procedure.[12]

Preparation of Alkynes

The following alkynes were purchased from commercial sources:

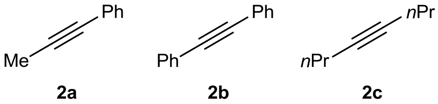

The following alkynes were prepared according to literature procedures.[3]

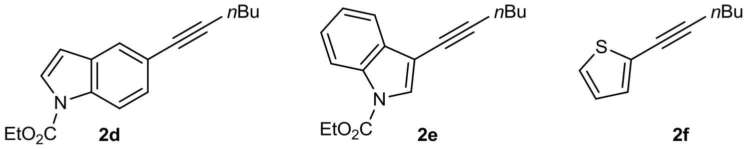

12. Bolz, I.; Schaarschmidt, D.; Rffer, T.; Lang, H.; Spange, S. *Angew. Chem., Int. Ed.* **2009**, *48*, 7440–7443.

Preparation of Chiral Cyclopentadienyl Rh(I) Metal Complexes[13]

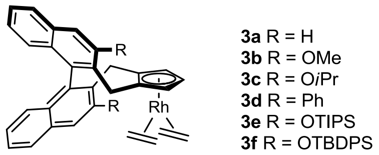

The chiral Rh(I) complexes **3a-3f** were prepared according to literature procedures,[13,14] with the following modifications to the routes:

(*R*)-3,3'-Dimethoxy-2,2'-bis(methoxymethoxy)-1,1'-binaphthalene (S17)[14,15]

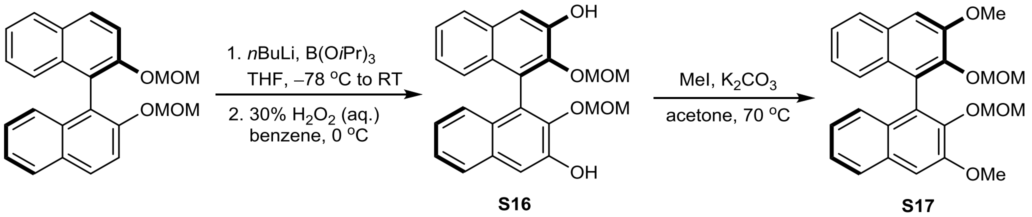

Following a literature procedure:[14] To a solution of (*R*)-2,2'-bis(methoxymethoxy)-1,1'-binaphthalene (7.00 g, 18.7 mmol) in THF (50 mL) at –78 °C was added a solution of *n*BuLi (2.5 M in THF, 18.0 mL, 44.9 mmol) dropwise. The mixture was slowly warmed to 0 °C, stirred for 4 h at the same temperature, and recooled to –78 °C, before the dropwise addition of triisopropyl borate (13.0 mL, 56.1 mmol). (**Note:** High-quality triisopropyl borate is important for the success of this reaction. Triisipropyl borate obtained from Sigma Aldrich was used in this case.) The mixture was again slowly warmed to room temperature and then stirred for 12 h before being concentrated *in vacuo*. The residue was dissolved in benzene (50 mL) and cooled to 0 °C, before a solution of H_2O_2 (30% in H_2O, 14 mL, 123.4 mmol) was added dropwise. The resulting mixture was stirred at 0 °C for 4 h.

Modified workup procedure*:* At this stage, the workup procedure was changed slightly from the literature method,[14] which in our hands often gave unidentified material after the solvent was removed. Instead, we kept the product as a solution in benzene. The modified procedure is as follows: The reaction was quenched with H_2O (20 mL), and the layers were separated. The organic layer was passed through a short pad of Na_2SO_4 under nitrogen atmosphere and the aqueous layer was extracted with benzene (2 × 20 mL). The combined organic extracts were passed through a short pad of Na_2SO_4 under nitrogen atmosphere, and then combined with the first organic layer to give a solution of 2,2'-bis(methoxymethoxy)-[1,1'-binaphthalene]-3,3'-diol (**S16**) in benzene which was used immediately in the next step.

13. Ye, B.; Cramer, N. *J. Am. Chem. Soc.* **2013**, *135*, 636–639.
14. Chau, S. T.; Lutz, J. P.; Wu, K.; Doyle, A. G. *Angew. Chem., Int. Ed.* **2013**, *52*, 9153–9156.
15. Ooi, T.; Kameda, M.; Maruoka, K. *J. Am. Chem. Soc.* **2003**, *125*, 5139–5151.

To a cold (0 °C) solution of 2,2'-bis(methoxymethoxy)-[1,1'-binaphthalene]-3,3'-diol obtained as described above was added K_2CO_3 (7.75 g, 56.1 mmol) and acetone (100 mL), before iodomethane (5.8 mL, 93.5 mmol) was added dropwise. The mixture was then heated at 70 °C for 24 h. After cooling to room temperature, the mixture was filtered on a short pad of silica concentrated *in vacuo*. Purification of the residue by flash column chromatography (4% EtOAc/hexane) to give the dimethyl ether **S17** as a white solid (6.42 g, 79%) that displayed spectroscopic data consistent with those reported previously.[15] R_f 0.41 (10% EtOAc/hexane).

(*R*)-3,3'-Dimethoxy-[1,1'-binaphthalene]-2,2'-diol (S18)

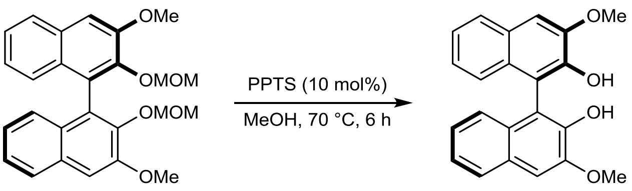

S17 **S18**

Similar literature procedures[14,15] employ HCl for MOM deprotection. We found it more convenient/higher yielding to use PPTS in MeOH:

To a solution of **S17** (12.6 g, 29.1 mmol, 1 equiv) in MeOH (80 mL) was added PPTS (730 mg, 2.91 mmol) and the mixture was heated at 70 °C for 6 h. After cooling to room temperature, the solvent was removed *in vacuo*, the residue was dissolved in 50% EtOAc/hexane, and filtered on a short pad of silica to give the binaphthol **S18** as a white solid (9.95 g, 99%) that displayed spectroscopic data consistent with those reported previously.[15] R_f 0.32 (20% EtOAc/hexane).

2,2'-Bis(bromomethyl)-3,3'-dimethoxy-1,1'-binaphthalene (S19)

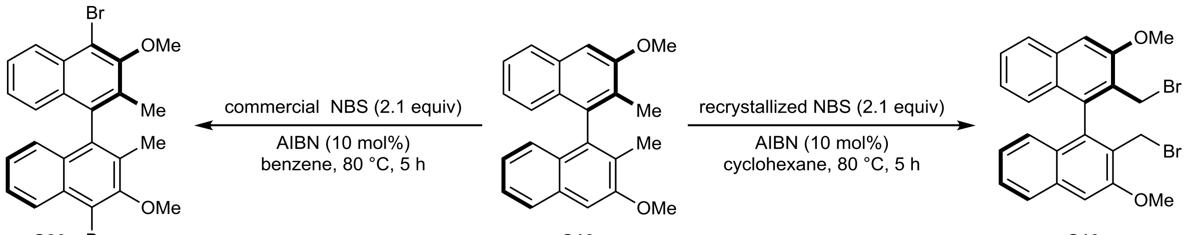

S20 **S18** **S19**

A similar procedure to that described in the literature[13] was used, but we found greater reproducibility was obtained when freshly recrystallized NBS was employed (whether in cyclohexane or benezene as the solvent). Use of unrecrystallized NBS sometimes resulted in aromatic bromination to give **S20**, rather than benzylic bromination.

With Recrystallized NBS: A mixture of **S18** (2.00 g, 5.85 mmol), freshly recrystallized NBS (2.18 g, 12.3 mmol), and AIBN (96 mg, 0.58 mmol) in cyclohexane (30 mL) was heated at reflux for 5 h. After cooling the reaction mixture to 23 °C, EtOAc (100 mL) was added and washed with water (2

× 50 mL). The organic layer was dried (Na_2SO_4), filtered, and concentrated *in vacuo*. Purification of the residue by flash column chromatography (2% EtOAc/petrol) gave the dibromide **S19** as an off-white solid (2.82 g, 97%) that displayed spectroscopic data consistent with those reported previously.[13]

Use of unrecrystallized NBS in benzene often gave the aromatic dibromide **S20** as an off-white solid which had the following NMR data: ^{1}H NMR (400 MHz, $CDCl_3$) δ 8.34 (2H, d, *J* = 8.4 Hz, Ar**H**), 7.52 (2H, t, *J* = 7.1 Hz, Ar**H**), 7.26-7.18 (2H, m, Ar**H**), 6.99 (2H, d, *J* = 8.3 Hz, Ar**H**), 3.99 (6H, s, 2 × OC**H**$_3$), 2.03 (6H, s, 2 × C**H**$_3$).

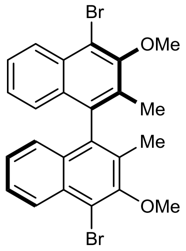

S20

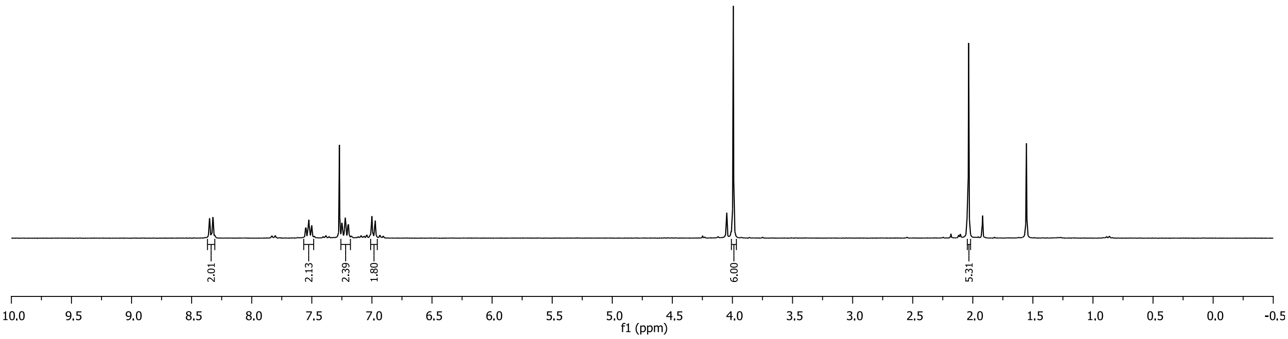

3,5-Dihydrospiro[cyclohepta[1,2-*a*:7,6-*a*']dinaphthalene-4,1'-cyclopenta[2,4]diene]-2,6-diol (S22)[13]

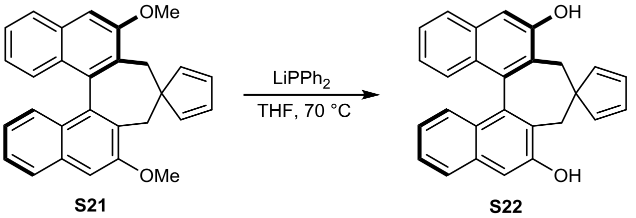

S21 **S22**

The following method was preferred over the literature procedure,[13] which uses toxic/malodorous

reagents. The procedure was adapted from one contained in a University of Nottingham thesis.[16] To a solution of PPh_3 (4.97 g, 18.9 mmol) in THF (70 mL) at 0 °C was added lithium metal (262 mg, 37.8 mmol) in portions and the mixture was heated at 60 °C for 6 h to generate a mixture of lithium diphenylphosphine and phenyl lithium as a bright red-colored solution which was transferred *via* cannula to a solution of the dimethyl ether **S21**[13] (1.53 g, 3.79 mmol) in dry THF (10 mL) at ambient temperature. The mixture was heated at reflux for 6 h, cooled to 0 °C, quenched carefully with 10% aqueous HCl solution (50 mL) and extracted with Et_2O (2 x 100 mL). The combined organic layers were dried (Na_2SO_4), filtered, and concentrated *in vacuo*. Purification of the residue by flash column chromatography (15% acetone/petrol) gave the diol **S22** as a pale yellow solid (1.37 g, 96%) that displayed spectroscopic data consistent with those reported previously.[13] R_f 0.32 (20% EtOAc/hexane).

16. Prepared according to the procedure contained in the Ph.D. thesis of Rebecca E. Meadows: The University of Nottingham. **2008**, pp 93–94. http://ethos.bl.uk/OrderDetails.do?uin=uk.bl.ethos.479265.

Enantioselective Rhodium-Catalyzed Spiroannulation Reactions: General Procedure

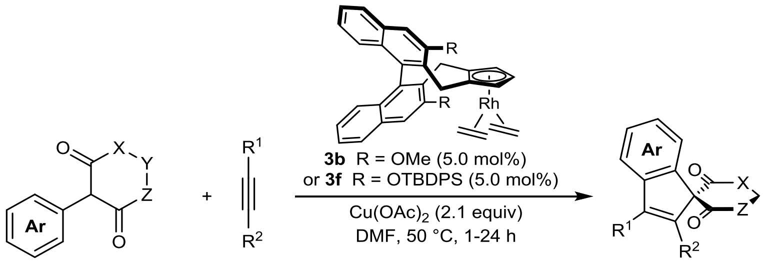

To a mixture of the appropriate 2-aryl-1,3-dicarbonyl compound, $Cu(OAc)_2$ (2.1 equiv), and the appropriate chiral cyclopentadienyl rhodium complex (5 mol%) in DMF (2.5 mL) was added the appropriate alkyne (1.5 equiv) in DMF (0.5 mL), and the resulting mixture was stirred at 50 °C for the indicated time (1–24 h). The reaction was filtered through a pad of silica gel eluting with EtOAc (50 mL) and the filtrate was washed with H_2O (25 mL) and brine (25 mL). The organic layer was dried ($MgSO_4$), filtered, and concentrated *in vacuo*. Purification of the residue by flash column chromatography gave the spiroindene.

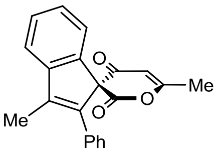

(*S*)-3,6'-Dimethyl-2-phenyl-2',4'-dihydrospiro[indene-1,3'-pyran]-2',4'-dione (4a). The title compound was prepared according to the General Procedure from substrate **1a** (60.6 mg, 0.30 mmol), **3f** (15.2 mg, 15 µmol), $Cu(OAc)_2$ (114 mg, 0.63 mmol), and alkyne **2a** (56 µL, 0.45 mmol) for a reaction time of 1 h and was purified by flash column chromatography (10→20% EtOAc/petrol) to give a pale yellow solid (79.4 mg, 84%). R_f 0.34 (50% EtOAc/petrol); $[\alpha]_D^{20}$ +92.1 (*c* 0.50, $CHCl_3$); m.p. 174−175 °C (CH_2Cl_2/hexane); IR 1767 (C=O), 1695 (C=O), 1327, 1253, 1131, 990, 757, 703 cm^{-1}; ^1H NMR (400 MHz, $CDCl_3$) δ 7.46–7.30 (7H, m, Ar**H**), 7.30–7.21 (2H, m, Ar**H**), 5.87 (1H, q, *J* = 0.8 Hz, CH_3C=C**H**), 2.28 (3H, d, *J* = 0.8 Hz, C**H**$_3$), 2.27 (3H, s, C**H**$_3$); ^{13}C NMR (100.6 MHz, $CDCl_3$) δ 189.0 (C), 168.3 (C), 166.4 (C), 146.4 (C), 142.0 (C), 141.3 (C), 139.6 (C), 134.3 (C), 129.3 (CH), 129.1 (2 × CH), 128.5 (2 × CH), 127.8 (CH), 126.4 (CH), 121.1 (CH), 120.9 (CH), 107.2 (CH), 74.7 (C), 20.5 (CH_3), 12.0 (CH_3); HRMS (ESI +ve) Exact mass calculated for $C_{21}H_{16}NaO_3$ $[M+Na]^+$: 339.0992, found: 339.0996; Enantiomeric excess was determined by HPLC with a Chiralpak AD-H column (95:5 *iso*-hexane:*iso*-PrOH, 0.8 mL/min, 254 nm, 25 °C); t_r (minor) = 20.6 min, t_r (major) = 22.1 min; 95% ee.

Data file: C:\CHEM32\1\DATA\SURESH\DEF_LC 2015-01-09 11-16-26\S_CHI-03-13A1.D
Sample name: S_Chi-03-13a1
Instrument: AGILENT 1260
Injection date: 1/9/2015 11:27:43 AM
Acq. method: ADH95B05A.50MIN.0.8 ML.M

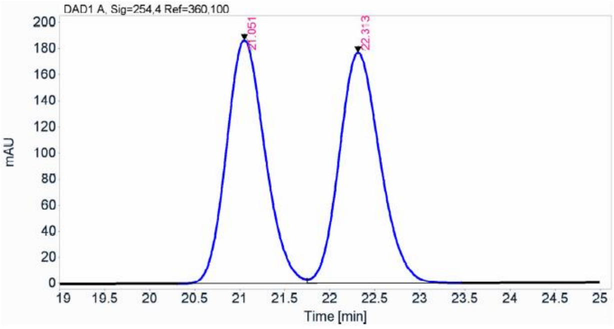

Signal: DAD1 A, Sig=254,4 Ref=360,100

RT [min]	Type	Width [min]	Area	Height	Area%
21.051	BV	0.4584	5548.844	186.9025	49.98
22.313	VB	0.4858	5552.626	177.0776	50.02

Data file: C:\CHEM32\1\DATA\DAVE\DEF_LC 2015-03-09 14-16-26\DJB7-27-D.D
Sample name: DJB7-27-D
Instrument: AGILENT 1260
Injection date: 3/9/2015 4:07:54 PM
Acq. method: ADH95B05A.50MIN.0.8 ML.M

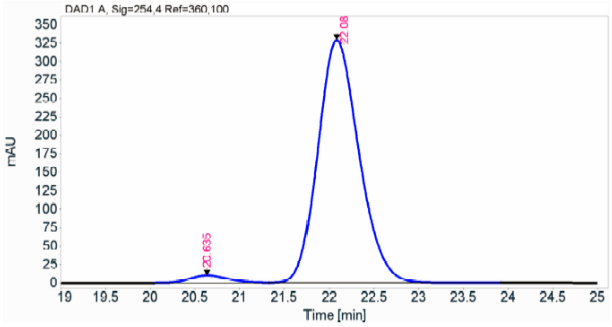

Signal: DAD1 A, Sig=254,4 Ref=360,100

RT [min]	Type	Width [min]	Area	Height	Area%
20.635	BB	0.4511	285.896	9.8348	2.66
22.080	BB	0.4887	10459.993	329.1808	97.34

At room temperature: The title compound was prepared according to a slight modification of the General Procedure (the reaction was conducted at room temperature rather than 50 °C) from substrate **1a** (41 mg, 0.20 mmol) **3f** (10.1 mg, 10 µmol), $Cu(OAc)_2$ (76.3 mg, 0.42 mmol), and alkyne **2a** (38 µL, 0.30 mmol) for 24 h, and was purified by column chromatography (9% acetone/petrol) to give an off-white solid (49.3 mg, 78%). Enantiomeric excess was determined by HPLC with a Chiralpak AD-H column (95:5 *iso*-hexane:*iso*-PrOH, 0.8 mL/min, 254 nm, 25 °C); t_r (minor) = 21.5 min, t_r (major) = 23.1 min; 97% ee.

Data file: C:\CHEM32\1\DATA\SURESH\DEF_LC 2015-01-09 11-16-26\S_CHI-03-13A1.D
Sample name: S_Chi-03-13a1
Instrument: AGILENT 1260
Injection date: 1/9/2015 11:27:43 AM
Acq. method: ADH95B05A.50MIN.0.8 ML.M

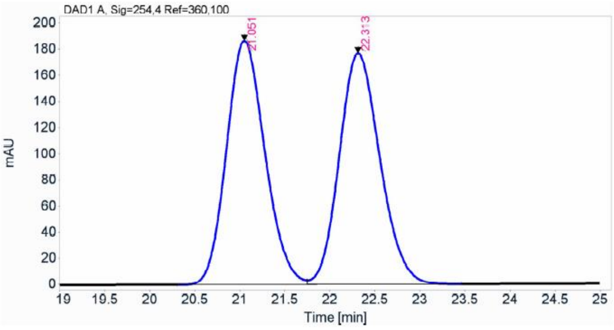

Signal: DAD1 A, Sig=254,4 Ref=360,100

RT [min]	Type	Width [min]	Area	Height	Area%
21.051	BV	0.4584	5548.844	186.9025	49.98
22.313	VB	0.4858	5552.626	177.0776	50.02

Data file: C:\CHEM32\1\DATA\SURESH\DEF_LC 2015-05-11 12-47-05\S_CHI-04-26.D
Sample name: s_chi-04-26
Instrument: AGILENT 1260
Injection date: 5/11/2015 12:59:43 PM
Acq. method: ADH95B05A.50MIN.0.8 ML.M

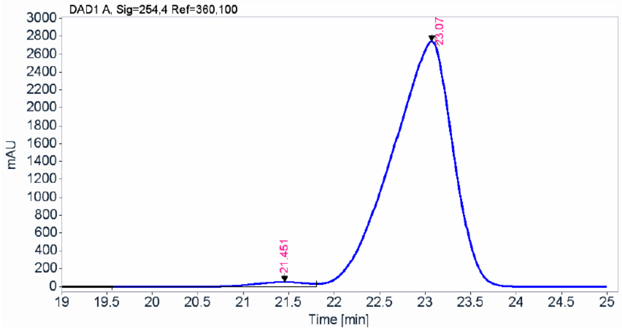

Signal: DAD1 A, Sig=254,4 Ref=360,100

RT [min]	Type	Width [min]	Area	Height	Area%
21.451	BV	0.6360	2186.748	53.3750	1.68
23.070	VB	0.6647	128196.625	2745.5950	98.32

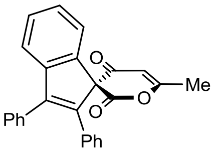

(*S*)-6'-Methyl-2,3-diphenyl-2',4'-dihydrospiro[indene-1,3'-pyran]-2',4'-dione (4b). The title compound was prepared according to the General Procedure from substrate **1a** (60.6 mg, 0.30 mmol), **3f** (15.2 mg, 15 µmol), $Cu(OAc)_2$ (114 mg, 0.63 mmol), and alkyne **2b** (80.2 mg, 0.45 mmol) for a reaction time of 5.5 h and was purified by flash column chromatography (10→20% acetone/petrol) to give a pale yellow solid (75.9 mg, 67%). R_f 0.34 (30% acetone/petrol); $[\alpha]_D^{20}$ +101.4 (*c* 1.00, $CHCl_3$); m.p. 173−174 °C (CH_2Cl_2/hexane); IR 1778 (C=O), 1681 (C=O), 1326, 1251, 1125, 981, 757 cm^{-1}; ^{1}H NMR (400 MHz, $CDCl_3$) δ 7.58–7.54 (2H, m, Ar**H**), 7.53–7.39 (6H, m, Ar**H**), 7.35–7.30 (1H, m, Ar**H**), 7.30–7.20 (5H, m, Ar**H**), 6.03 (1H, q, *J* = 0.8 Hz, CH_3C=C**H**), 2.42 (3H, d, *J* = 0.8 Hz, C**H**$_3$); ^{13}C NMR (100.6 MHz, $CDCl_3$) δ 188.4 (C), 168.3 (C), 166.0 (C), 145.9 (C), 145.7 (C), 141.1 (C), 140.4 (C), 133.9 (C), 133.7 (C), 129.3 (3 × CH), 129.1 (2 × CH), 128.7 (2 × CH), 128.3 (2 × CH), 128.1 (CH), 127.7 (CH), 126.6 (CH), 122.4 (CH), 121.1 (CH), 107.0 (CH), 74.4 (C), 20.5 (CH_3); HRMS (ESI +ve) Exact mass calculated for $C_{26}H_{18}NaO_3$ $[M+Na]^+$: 401.1148, found: 401.1151; Enantiomeric excess was determined by HPLC with a Chiralpak AD-H column (95:5 *iso*-hexane:*iso*-PrOH, 0.8 mL/min, 254 nm, 25 °C); t_r (minor) = 23.3 min, t_r (major) = 51.3 min; 93% ee.

Data file: C:\CHEM32\1\DATA\IK\DEF_LC 2015-05-29 15-26-57\IK-739F.D
Sample name: IK-739f
Instrument: AGILENT 1260
Injection date: 5/29/2015 3:39:21 PM
Acq. method: ADH95B05A.80MIN.0.8 ML.M

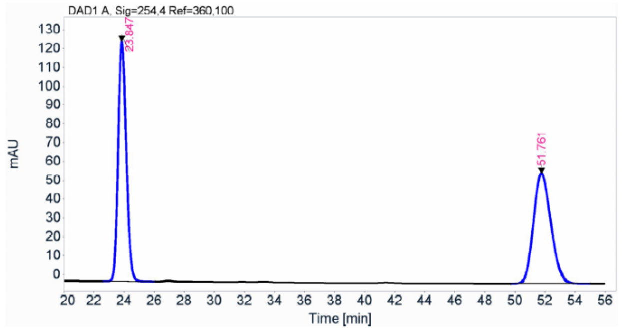

Signal: DAD1 A, Sig=254,4 Ref=360,100

RT [min]	Type	Width [min]	Area	Height	Area%
23.847	BB	0.5848	4790.690	127.3678	50.20
51.761	BB	1.2672	4752.538	58.5378	49.80

Data file: C:\CHEM32\1\DATA\DAVE\DEF_LC 2015-04-13 07-04-29\DJB7-33-1-2.D
Sample name: DJB7-33-1-2
Instrument: AGILENT 1260
Injection date: 4/13/2015 8:00:20 AM
Acq. method: ADH95B05A.60MIN.0.8 ML.M

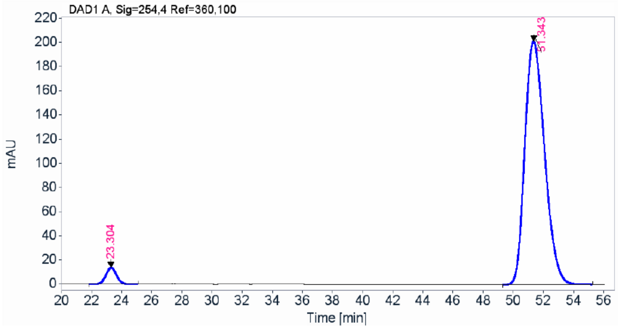

Signal: DAD1 A, Sig=254,4 Ref=360,100

RT [min]	Type	Width [min]	Area	Height	Area%
23.304	BB	0.7552	679.828	13.7419	3.58
51.343	BB	1.4270	18326.160	201.5161	96.42

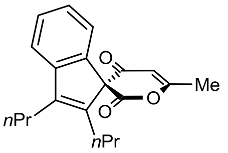

(*S*)-6'-Methyl-2,3-dipropyl-2',4'-dihydrospiro[indene-1,3'-pyran]-2',4'-dione (4c). The title compound was prepared according to the General Procedure from substrate **1a** (40.4 mg, 0.20 mmol), **3f** (10.1 mg, 10 µmol), $Cu(OAc)_2$ (76.3 mg, 0.42 mmol), and alkyne **2c** (44 µL, 0.30 mmol) for a reaction time of 24 h and was purified by flash column chromatography (10→20% EtOAc/petrol) to give a pale yellow solid (34.9 mg, 56%). R_f 0.63 (50% EtOAc/petrol); $[\alpha]_D^{20}$ +57.5 (*c* 1.00, $CHCl_3$); IR 2961, 2933, 1783 (C=O), 1670 (C=O), 1326, 1251, 1126, 986, 766 cm^{-1}; ^1H NMR (400 MHz, $CDCl_3$) δ 7.33 (1H, td, *J* = 7.4, 1.1 Hz, Ar**H**), 7.28 (1H, d, *J* = 7.4 Hz, Ar**H**), 7.18 (1H, d, *J* = 7.4 Hz, Ar**H**), 7.11 (1H, td, *J* = 7.4, 1.1 Hz, Ar**H**), 5.89 (1H, q, *J* = 0.8 Hz, CH_3C=C**H**), 2.60 (2H, t, *J* = 7.5 Hz, C$\mathbf{H}_2CH_2CH_3$), 2.46–2.39 (2H, m, C$\mathbf{H}_2CH_2CH_3$), 2.36 (3H, d, *J* = 0.8 Hz, C$\mathbf{H}_3$), 1.75–1.62 (2H, m, C$\mathbf{H}_2CH_3$), 1.51–1.39 (2H, m, C$\mathbf{H}_2CH_3$), 1.03 (3H, t, *J* = 7.4 Hz, $CH_2C\mathbf{H}_3$), 0.97 (3H, t, *J* = 7.3 Hz, $CH_2C\mathbf{H}_3$); ^{13}C NMR (100.6 MHz, $CDCl_3$) δ 189.3 (C), 168.2 (C), 166.8 (C), 146.0 (C), 144.4 (C), 141.3 (C), 141.2 (C), 129.0 (CH), 125.3 (CH), 120.7 (CH), 120.3 (CH), 107.1 (CH), 73.3 (C), 29.8 (CH_2), 27.5 (CH_2), 22.1 (CH_2), 21.6 (CH_2), 20.6 (CH_3), 14.7 (CH_3), 14.0 (CH_3); HRMS (ESI +ve) Exact mass calculated for $C_{20}H_{22}NaO_3$ $[M+Na]^+$: 333.1461, found: 33.1445; Enantiomeric excess was determined by HPLC with a Chiralpak AD-H column (95:5 *iso*-hexane:*iso*-PrOH, 0.8 mL/min, 254 nm, 25 °C); t_r (minor) = 8.6 min, t_r (major) = 11.6 min; 82% ee.

Data file: C:\CHEM32\1\DATA\IK\DEF_LC 2015-03-11 16-50-45\IK-739E.D
Sample name: IK-739e
Instrument: AGILENT 1260
Injection date: 3/11/2015 8:38:56 PM
Acq. method: ADH95B05A.50MIN.0.8 ML.M

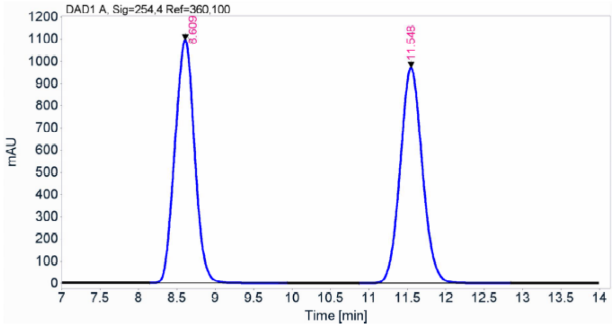

Signal: DAD1 A, Sig=254,4 Ref=360,100

RT [min]	Type	Width [min]	Area	Height	Area%
8.609	BB	0.2829	19407.504	1094.5066	49.96
11.548	BB	0.3094	19440.746	971.6356	50.04

Data file: C:\CHEM32\1\DATA\DAVE\DEF_LC 2015-04-13 07-04-29\DJB7-46-B-2.D
Sample name: DJB7-46-B-2
Instrument: AGILENT 1260
Injection date: 4/13/2015 7:38:52 AM
Acq. method: ADH95B05A.20MIN.0.8 ML.M

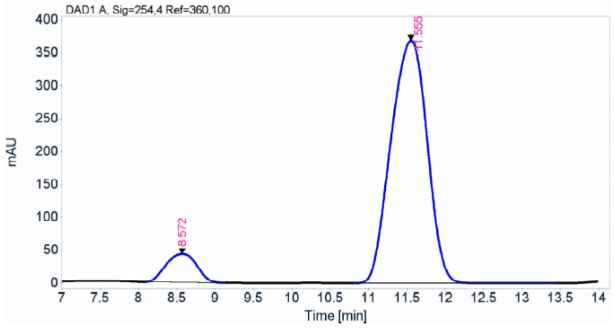

Signal: DAD1 A, Sig=254,4 Ref=360,100

RT [min]	Type	Width [min]	Area	Height	Area%
8.572	BB	0.4704	1215.946	43.1946	9.13
11.555	BB	0.5407	12098.212	368.8196	90.87

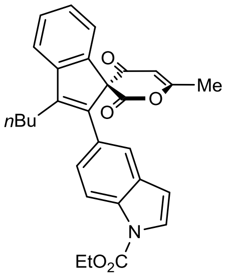

5-[(*S*)-3-Butyl-6'-methyl-2',4'-dioxo-2',4'-dihydrospiro[indene-1,3'-pyran]-2-yl]-1*H*-indole-1-carboxylate (4d). The title compound was prepared according to the General Procedure from substrate **1a** (60.6 mg, 0.30 mmol), **3f** (15.2 mg, 15 µmol), $Cu(OAc)_2$ (114 mg, 0.63 mmol), and alkyne **2d** (121 mg, 0.45 mmol) for a reaction time of 4 h to give a 19:1 mixture of regioisomers (by ^{1}H NMR analysis) which was purified by flash column chromatography (10→20% EtOAc/petrol) to give a pale yellow solid (115 mg, 82%, 19:1 rr). R_f 0.59 (50% EtOAc/petrol); $[\alpha]_D^{20}$ +74.8 (*c* 1.00, $CHCl_3$); m.p. 128−129 °C (CH_2Cl_2/hexane); IR 1750 (C=O), 1682 (C=O), 1465, 1373, 1325, 1236, 1084, 1043, 985, 753, 734 cm^{-1}; ^{1}H NMR for major regioisomer (400 MHz, $CDCl_3$) δ 8.19 (1H, d, *J* = 8.3 Hz, Ar**H**), 7.68 (1H, d, *J* = 3.7 Hz, Ar**H**), 7.56 (1H, d, *J* = 1.2 Hz, Ar**H**), 7.49–7.41 (2H, m, Ar**H**), 7.36–7.21 (3H, m, Ar**H**), 6.63 (1H, dd, *J* = 3.7, 0.5 Hz, Ar**H**), 5.83 (1H q, *J* = 0.8 Hz, $CH_3C=C\mathbf{H}$), 4.54 (2H, q, *J* = 7.1 Hz, $OC\mathbf{H}_2$), 2.66–2.59 (2H, m, $C\mathbf{H}_2CH_2CH_2CH_3$), 2.21 (3H, d, *J* = 0.7 Hz, $C\mathbf{H}_3C=$), 1.73–1.62 (2H, m, $C\mathbf{H}_2CH_2CH_3$), 1.51 (3H, t, *J* = 7.1 Hz, $OCH_2C\mathbf{H}_3$), 1.45–1.34 (2H, m, $CH_2C\mathbf{H}_2CH_3$), 0.88 (3H, t, *J* = 7.3 Hz, $CH_2C\mathbf{H}_3$); ^{1}H NMR diagnostic signals for minor regioisomer (400 MHz, $CDCl_3$) δ 8.33 (1H, d, *J* = 8.0 Hz, Ar**H**), 7.73 (1H, d, *J* = 3.7 Hz, Ar**H**), 7.71 (1H, *J* = 1.0 Hz, Ar**H**), 6.69 (1H, d, *J* = 3.2 Hz, Ar**H**), 5.98 (1H, q, *J* = 0.8 Hz, $CH_3C=C\mathbf{H}$), 2.43 (3H, d, *J* = 0.7 Hz, $C\mathbf{H}_3$), 0.83 (3H, t, *J* = 7.3 Hz, $CH_2C\mathbf{H}_3$); ^{13}C NMR for major regioisomer (100.6 MHz, $CDCl_3$) δ 189.2 (C), 168.1 (C), 166.5 (C), 150.9 (C), 146.6 (C), 145.8 (C), 141.9 (C), 140.2 (C), 134.7 (C), 130.5 (C), 129.2 (C), 129.1 (CH), 126.1 (CH), 126.0 (2 × CH), 121.9 (CH), 121.3 (2 × CH), 115.0 (CH), 108.1 (CH), 107.3 (CH), 75.3 (C), 63.3 (CH_2), 30.6 (CH_2), 25.8 (CH_2), 22.6 (CH_2), 20.4 (CH_3), 14.4 (CH_3), 13.8 (CH_3); HRMS (ESI +ve) Exact mass calculated for $C_{29}H_{27}NNaO_5$ $[M+Na]^+$: 492.1781, found: 492.1772; Enantiomeric excess was determined by HPLC with a Chiralpak AD-H column (95:5 *iso*-hexane:*iso*-PrOH, 0.8 mL/min, 280 nm, 25 °C); t_r (minor) = 23.8 min, t_r (major) = 34.1 min; 89% ee.

Data file: C:\CHEM32\1\DATA\IK\DEF_LC 2015-03-11 16-50-45\IK-739B.D
Sample name: IK-739b
Instrument: AGILENT 1260
Injection date: 3/11/2015 6:14:58 PM
Acq. method: ADH95B05A.50MIN.0.8 ML.M

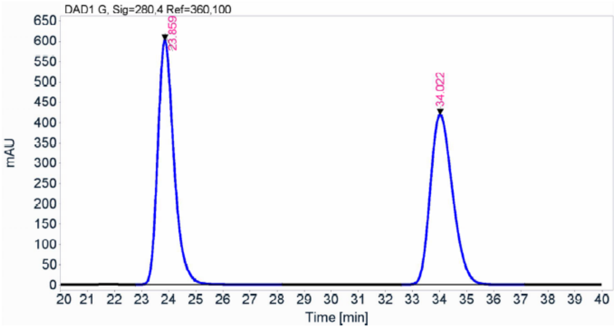

Signal: DAD1 G, Sig=280,4 Ref=360,100

RT [min]	Type	Width [min]	Area	Height	Area%
23.859	BB	0.6394	25056.605	602.2017	50.96
34.022	BB	0.8938	24114.682	420.0058	49.04

Data file: C:\CHEM32\1\DATA\DAVE\DEF_LC 2015-04-13 11-37-55\DJB7-44-A-3.D
Sample name: DJB7-44-A-3
Instrument: AGILENT 1260
Injection date: 4/13/2015 4:35:54 PM
Acq. method: ADH95B05A.60MIN.0.8 ML.M

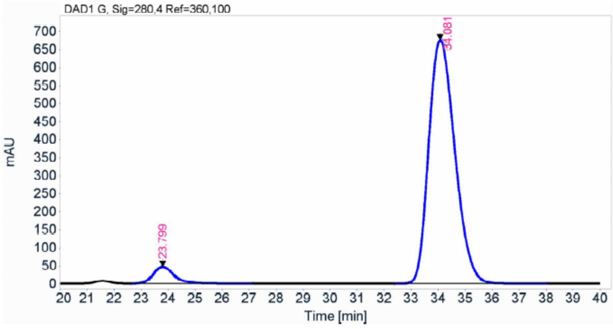

Signal: DAD1 G, Sig=280,4 Ref=360,100

RT [min]	Type	Width [min]	Area	Height	Area%
23.799	BB	0.8315	2604.954	47.5260	5.42
34.081	BB	1.0554	45418.320	676.3924	94.58

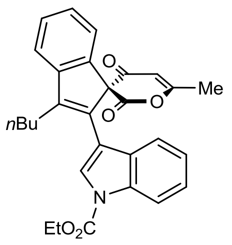

3-[(*S*)-3-Butyl-6'-methyl-2',4'-dioxo-2',4'-dihydrospiro[indene-1,3'-pyran]-2-yl]-1*H*-indole-1-carboxylate (4e). The title compound was prepared according to the General Procedure from substrate **1a** (60.6 mg, 0.30 mmol), **3f** (15.2 mg, 15 µmol), $Cu(OAc)_2$ (114 mg, 0.63 mmol), and alkyne **2e** (121 mg, 0.45 mmol) for a reaction time of 4 h and was purified by flash column chromatography (10→20% EtOAc/petrol) to give a colorless foam (104 mg, 74%). R_f 0.59 (50% EtOAc/petrol); $[\alpha]_D^{20}$ +75.1 (*c* 1.00, $CHCl_3$); m.p. 150−151 °C (CH_2Cl_2/hexane); IR 1745 (C=O), 1682 (C=O), 1454, 1380, 1251, 1232, 1104, 1063, 989, 760, 749 cm^{-1}; ^{1}H NMR (400 MHz, $CDCl_3$) δ 8.22 (1H, d, *J* = 8.1 Hz, Ar**H**), 7.63 (1H, d, *J* = 7.5 Hz, Ar**H**), 7.59 (1H, s, Ar**H**), 7.50–7.41 (2H, m, Ar**H**), 7.41–7.34 (1H, m, Ar**H**), 7.34–7.22 (3H, m, Ar**H**), 5.79 (1H, d, *J* = 0.8 Hz, CH_3C=C**H**), 4.51 (1H, q, *J* = 7.1 Hz, OC**H**$_2$), 2.57 (2H, t, *J* = 8.0 Hz, C**H**$_2CH_2CH_2CH_3$), 2.20 (3H, d, *J* = 0.8 Hz, C**H**$_3$C=), 1.66–1.56 (2H, m, C**H**$_2CH_2CH_3$), 1.48 (3H, t, *J* = 7.1 Hz, OCH_2C**H**$_3$), 1.36–1.25 (2H, m, CH_2C**H**$_2CH_3$), 0.80 (3H, t, *J* = 7.3 Hz, CH_2CH_2C**H**$_3$); ^{13}C NMR (100.6 MHz, $CDCl_3$) δ 188.7 (C), 168.2 (C), 166.3 (C), 150.8 (C), 149.4 (C), 145.0 (C), 141.6 (C), 134.9 (C), 131.6 (C), 130.0 (C), 129.2 (CH), 126.3 (CH), 124.8 (CH), 124.5 (CH), 123.1 (CH), 121.3 (CH), 121.2 (CH), 121.0 (CH), 115.4 (C), 114.9 (CH), 107.1 (CH), 75.4 (C), 63.3 (CH_2), 30.4 (CH_2), 26.4 (CH_2), 22.3 (CH_2), 20.4 (CH_3), 14.4 (CH_3), 13.8 (CH_3); HRMS (ESI +ve) Exact mass calculated for $C_{29}H_{27}NNaO_5$ $[M+Na]^+$: 492.1781, found: 492.1781; Enantiomeric excess was determined by HPLC with a Chiralpak OD-H column (95:5 *iso*-hexane:*iso*-PrOH, 0.8 mL/min, 254 nm, 25 °C); t_r (minor) = 18.1 min, t_r (major) = 34.0 min; 97% ee.

Data file: C:\CHEM32\1\DATA\IK\DEF_LC 2015-03-12 11-01-45\IK-739A.D
Sample name: IK-739a
Instrument: AGILENT 1260
Injection date: 3/12/2015 11:13:52 AM
Acq. method: ODH95B05A.0.8ML.60 MIN.M

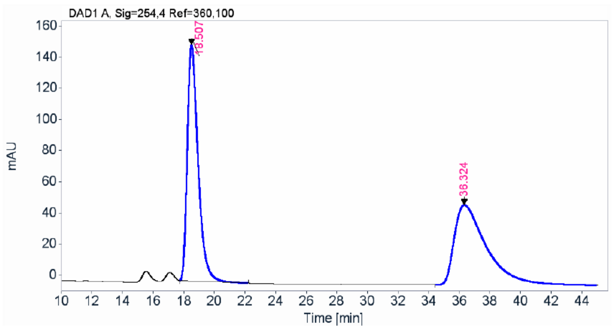

Signal: DAD1 A, Sig=254,4 Ref=360,100

RT [min]	Type	Width [min]	Area	Height	Area%
18.507	BB	0.7471	7428.627	151.7660	49.74
36.324	BB	2.1263	7505.731	51.2602	50.26

Data file: C:\CHEM32\1\DATA\IK\DEF_LC 2015-05-01 16-00-44\SCH-23B.D
Sample name: Sch-23b
Instrument: AGILENT 1260
Injection date: 5/1/2015 4:59:34 PM
Acq. method: ODH95B05A.0.8ML.60 MIN.M

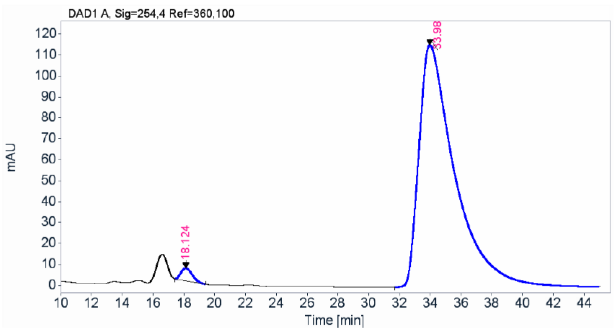

Signal: DAD1 A, Sig=254,4 Ref=360,100

RT [min]	Type	Width [min]	Area	Height	Area%
18.124	BB	0.7966	307.502	6.0132	1.61
33.980	BB	2.2902	18767.170	115.5888	98.39

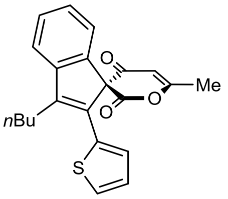

(*S*)-3-Butyl-6'-methyl-2-(thiophen-3-yl)-2',4'-dihydrospiro[indene-1,3'-pyran]-2',4'-dione (4f). The title compound was prepared according to the General Procedure from substrate **1a** (60.6 mg, 0.30 mmol), **3f** (15.2 mg, 15 µmol), $Cu(OAc)_2$ (114 mg, 0.63 mmol), and alkyne **2f** (73.8 mg, 0.45 mmol) for a reaction time of 4 h and was purified by flash column chromatography (10→30% EtOAc/petrol) to give a pale yellow solid (102 mg, 93%). R_f 0.38 (50% EtOAc/petrol); $[\alpha]_D^{20}$ +134.0 (*c* 1.00, $CHCl_3$); m.p. 128−129 °C (CH_2Cl_2/hexane); IR 1775 (C=O), 1674 (C=O), 1325, 1250, 1128, 986, 759, 704 cm^{-1}; ^1H NMR (400 MHz, $CDCl_3$) δ 7.43–7.37 (2H, m, Ar**H**), 7.33 (1H, dd, *J* = 5.1, 1.1 Hz, Ar**H**), 7.28–7.25 (1H, m, Ar**H**), 7.22–7.17 (1H, m, Ar**H**), 7.03 (1H, dd, *J* = 5.1, 3.7 Hz, Ar**H**), 6.82 (1H, dd, *J* = 3.7, 1.1 Hz, Ar**H**), 5.95 (1H, q, *J* = 0.8 Hz, $CH_3C=C\mathbf{H}$), 2.98–2.87 (2H, m, $C\mathbf{H}_2CH_2CH_2CH_3$), 2.39 (2H, d, *J* = 0.8 Hz, $C\mathbf{H}_3C=$), 1.80–1.69 (2H, m, $C\mathbf{H}_2CH_2CH_3$), 1.62–1.49 (2H, m, $C\mathbf{H}_2CH_3$), 1.00 (3H, t, *J* = 7.3 Hz, $CH_2C\mathbf{H}_3$); ^{13}C NMR (100.6 MHz, $CDCl_3$) δ 188.4 (C), 168.3 (C), 166.0 (C), 145.9 (C), 145.5 (C), 140.6 (C), 136.2 (C), 132.8 (C), 129.5 (CH), 127.2 (CH), 126.4 (CH), 126.3 (CH), 125.9 (CH), 121.3 (CH), 120.8 (CH), 106.9 (CH), 74.2 (C), 30.2 (CH_2), 26.6 (CH_2), 23.0 (CH_2), 20.6 (CH_3), 14.0 (CH_3); HRMS (ESI +ve) Exact mass calculated for $C_{22}H_{20}NaO_3S$ $[M+Na]^+$: 387.1025, found: 387.1034; Enantiomeric excess was determined by HPLC with a Chiralpak AD-H column (95:5 *iso*-hexane:*iso*-PrOH, 0.8 mL/min, 254 nm, 25 °C); t_r (minor) = 22.8 min, t_r (major) = 29.9 min; 93% ee.

Data file: C:\CHEM32\1\DATA\IK\DEF_LC 2015-03-11 16-50-45\IK-739C.D
Sample name: IK-739c
Instrument: AGILENT 1260
Injection date: 3/11/2015 7:26:57 PM
Acq. method: ADH95B05A.50MIN.0.8 ML.M

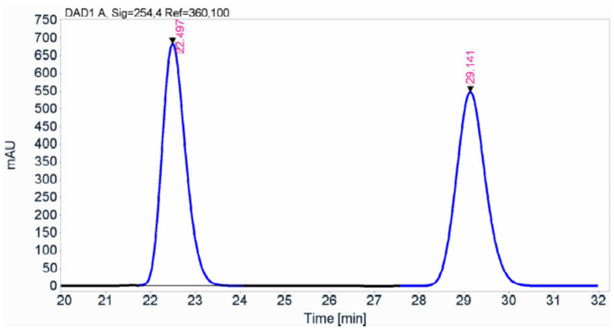

Signal: DAD1 A, Sig=254,4 Ref=360,100

RT [min]	Type	Width [min]	Area	Height	Area%
22.497	BB	0.5695	25029.740	683.0684	49.88
29.141	BB	0.7073	25152.002	548.1411	50.12

Data file: C:\CHEM32\1\DATA\DAVE\DEF_LC 2015-06-01 08-15-44\DJB7-45-C-RPT.D
Sample name: DJB7-45-C-rpt
Instrument: AGILENT 1260
Injection date: 6/1/2015 10:51:36 AM
Acq. method: ADH95B05A.60MIN.0.8 ML.M

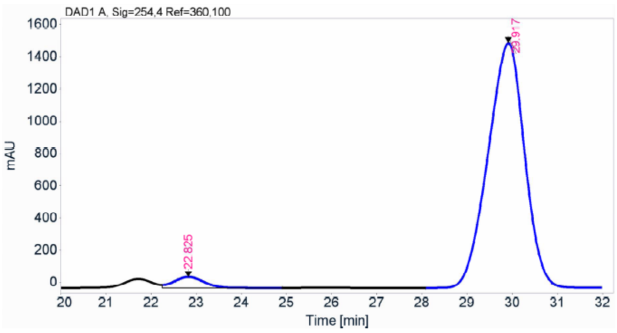

Signal: DAD1 A, Sig=254,4 Ref=360,100

RT [min]	Type	Width [min]	Area	Height	Area%
22.825	VB	0.7205	3235.404	69.8312	3.65
29.917	BB	0.8670	85326.297	1519.8173	96.35

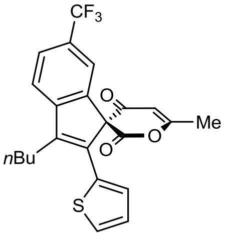

(*S*)-3-Butyl-6'-methyl-2-(thiophen-2-yl)-6-(trifluoromethyl)-2',4'-dihydrospiro[indene-1,3'-pyran]-2',4'-dione (4g). The title compound was prepared according to the General Procedure from substrate **1b** (54 mg, 0.20 mmol), **3f** (10.1 mg, 10 μmol), $Cu(OAc)_2$ (76.3 mg, 0.42 mmol), and alkyne **2f** (49.2 mg, 0.30 mmol) for a reaction time of 4 h and was purified by flash column chromatography (7% acetone/petrol) to give a light brown solid (58 mg, 67%). R_f 0.54 (30% acetone/petrol); $[\alpha]_D^{20}$ +4.0 (*c* 1.30, $CHCl_3$); m.p. 177–178 °C (acetone/petrol); IR 1776 (C=O), 1677 (C=O), 1649, 1463, 1322, 1281, 1123, 987, 836 cm^{-1}; ^1H NMR (500 MHz, $CDCl_3$) δ 7.71–7.65 (1H, m, Ar**H**), 7.49 (1H, d, *J* = 8.0 Hz, Ar**H**), 7.43–7.40 (1H, m, Ar**H**), 7.38 (1H, dd, *J* = 5.1, 1.0 Hz, Ar**H**), 7.07–7.02 (1H, m, Ar**H**), 6.86 (1H, dd, *J* = 3.7, 1.1 Hz, Ar**H**), 5.99 (1H, q, *J* = 0.8 Hz, $CH_3C=C\mathbf{H}$), 3.00–2.85 (2H, m, $C\mathbf{H}_2CH_2CH_2CH_3$), 2.43 (3H, d, *J* = 0.8 Hz, $C\mathbf{H}_3C=$), 1.79–1.66 (2H, m, $C\mathbf{H}_2CH_2CH_3$), 1.61–1.47 (2H, m, $C\mathbf{H}_2CH_3$), 0.99 (3H, t, *J* = 7.3 Hz, $CH_2C\mathbf{H}_3$); ^{13}C NMR (125.8 MHz, $CDCl_3$) δ 187.5 (C), 168.8 (C), 165.3 (C), 149.3 (C), 144.9 (C), 141.1 (C), 136.0 (C), 135.5 (C), 128.3 (C, q, *J* = 32.7 Hz), 127.4 (CH), 127.1 (CH), 127.0 (CH, q, *J* = 3.8 Hz), 126.9 (CH), 124.0 (C, q, *J* = 272.0 Hz), 121.3 (CH), 117.7 (CH, q, *J* = 3.8 Hz), 106.9 (CH), 73.8 (C), 30.1 (CH_2), 26.5 (CH_2), 23.0 (CH_2), 20.8 (CH_3), 13.9 (CH_3); ^{19}F NMR (376 MHz, $CDCl_3$) δ –61.8 (3F, s, CF_3); HRMS (ESI +ve) Exact mass calculated for $C_{23}H_{19}F_3NaO_3S$ $[M+Na]^+$: 455.0899, found: 455.0913. Enantiomeric excess was determined by HPLC with a Chiralpak AD-H column (95:5 *iso*-hexane:*iso*-PrOH, 0.8 mL/min, 254 nm, 25 °C); t_r (minor) = 14.6 min, t_r (major) = 23.5 min; 96% ee.

Data file: C:\CHEM32\1\DATA\IK\DEF_LC 2015-04-15 10-10-48\IK-761A.D
Sample name: IK-761a
Instrument: AGILENT 1260
Injection date: 4/15/2015 1:07:49 PM
Acq. method: ADH95B05A.60MIN.0.8 ML.M

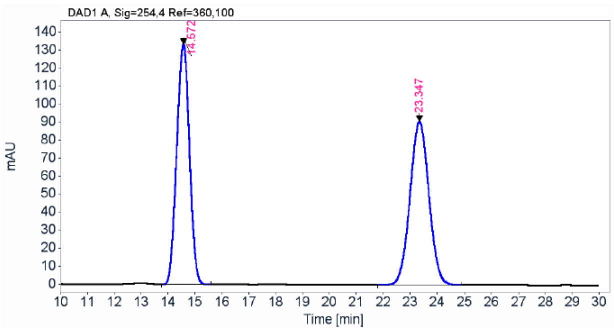

Signal: DAD1 A, Sig=254,4 Ref=360,100

RT [min]	Type	Width [min]	Area	Height	Area%
14.572	BB	0.5205	4397.250	133.4075	49.78
23.347	BB	0.7619	4435.854	90.8039	50.22

Data file: C:\CHEM32\1\DATA\IK\DEF_LC 2015-04-16 08-31-54\IK-761.D
Sample name: IK-761
Instrument: AGILENT 1260
Injection date: 4/16/2015 1:36:43 PM
Acq. method: ADH95B05A.50MIN.0.8 ML.M

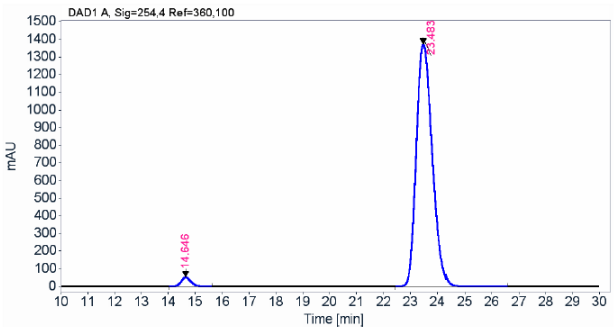

Signal: DAD1 A, Sig=254,4 Ref=360,100

RT [min]	Type	Width [min]	Area	Height	Area%
14.646	BB	0.3790	1293.767	52.5880	2.25
23.483	BB	0.6447	56330.527	1372.7688	97.75

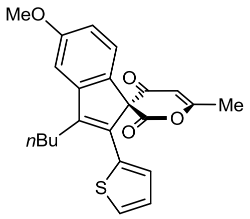

(*S*)-3-Butyl-5-methoxy-6'-methyl-2-(thiophen-2-yl)-2',4'-dihydrospiro[indene-1,3'-pyran]-2',4'-dione (4h). The title compound was prepared according to the General Procedure from substrate **1c** (46.4 mg, 0.20 mmol), **3f** (10.1 mg, 10 μmol), $Cu(OAc)_2$ (76.3 mg, 0.42 mmol), and alkyne **2f** (49.2 mg, 0.30 mmol) for a reaction time of 4 h and was purified by flash column chromatography (10% acetone/petrol) to give a light brown solid (67 mg, 85%). R_f 0.45 (30% acetone/petrol); $[\alpha]_D^{20}$ +10.9 (*c* 1.20, $CHCl_3$); m.p. 184–185 °C (acetone/petrol); IR 1778 (C=O), 1672 (C=O), 1645, 1596, 1479, 1328, 1257, 1130, 989, 857, 722 cm^{-1}; ^1H NMR (400 MHz, $CDCl_3$) δ 7.32 (1H, dd, *J* = 5.1, 1.1 Hz, Ar**H**), 7.15 (1H, d, *J* = 8.3 Hz, Ar**H**), 7.02 (1H, dd, *J* = 5.1, 3.7 Hz, Ar**H**), 6.93 (1H, d, *J* = 2.4 Hz, Ar**H**), 6.81 (1H, dd, *J* = 3.7, 1.1 Hz, Ar**H**), 6.70 (1H, dd, *J* = 8.3, 2.4 Hz, Ar**H**), 5.92 (1H, q, *J* = 0.8 Hz, CH_3C=C**H**), 3.84 (3H, s, OC**H**$_3$), 2.93–2.84 (2H, m, C**H**$_2CH_2CH_2CH_3$), 2.37 (3H, d, *J* = 0.8 Hz, C**H**$_3$C=), 1.79–1.67 (2H, m, C**H**$_2CH_2CH_3$), 1.59–1.49 (2H, m, CH_2C**H**$_2CH_3$), 0.99 (3H, t, *J* = 7.3 Hz, CH_2C**H**$_3$); ^{13}C NMR (100.6 MHz, $CDCl_3$) δ 188.6 (C), 168.2 (C), 166.2 (C), 161.1 (C), 147.2 (C), 145.7 (C), 136.2 (C), 134.0 (C), 132.9 (C), 127.2 (CH), 126.4 (CH), 126.0 (CH), 121.5 (CH), 111.1 (CH), 107.7 (CH), 106.8 (CH), 73.5 (C), 55.6 (CH_3), 30.2 (CH_2), 26.5 (CH_2), 23.0 (CH_2), 20.6 (CH_3), 13.9 (CH_3); HRMS (ESI +ve) Exact mass calculated for $C_{23}H_{22}NaO_4S$ $[M+Na]^+$: 417.1131, found: 417.1129. Enantiomeric excess was determined by HPLC with a Chiralpak IC column (90:10 *iso*-hexane:EtOH, 1.0 mL/min, 254 nm, 25 °C); t_r (minor) = 10.3 min, t_r (major) = 10.9 min; 95% ee.

Data file:	C:\CHEM32\1\DATA\IK\DEF_LC 2015-04-20 09-27-03\SCH-98D4.D
Sample name:	SCh-98d4
Instrument:	AGILENT 1260
Injection date:	4/20/2015 4:59:43 PM
Acq. method:	IC90B10D.30MIN.1.0M L.M

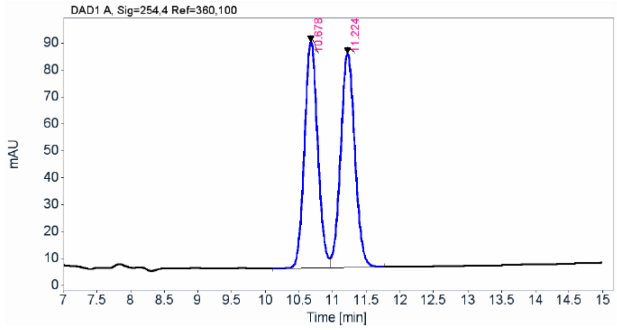

Signal: DAD1 A, Sig=254,4 Ref=360,100

RT [min]	Type	Width [min]	Area	Height	Area%
10.678	BV	0.2139	1151.551	83.9976	49.80
11.224	VB	0.2264	1160.720	79.4868	50.20

Data file:	C:\CHEM32\1\DATA\IK\DEF_LC 2015-04-20 09-27-03\IK-760A2.D
Sample name:	IK-760a2
Instrument:	AGILENT 1260
Injection date:	4/20/2015 5:36:12 PM
Acq. method:	IC90B10D.30MIN.1.0M L.M

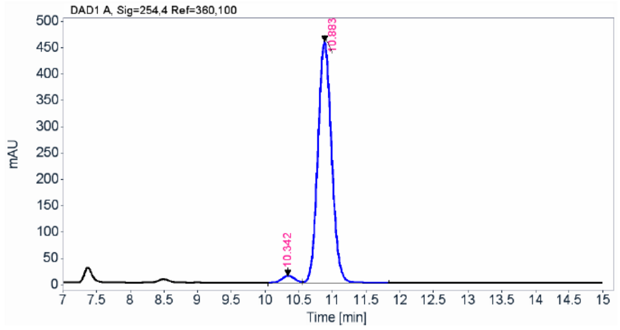

Signal: DAD1 A, Sig=254,4 Ref=360,100

RT [min]	Type	Width [min]	Area	Height	Area%
10.342	BV	0.2053	177.612	13.5206	2.68
10.883	VB	0.2206	6455.207	457.4149	97.32

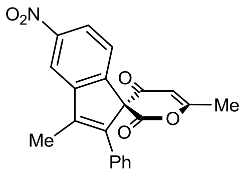

(*S*)-3,6'-Dimethyl-5-nitro-2-phenyl-2',4'-dihydrospiro[indene-1,3'-pyran]-2',4'-dione (4i). The title compound was prepared according to the General Procedure from substrate **1d** (74.1 mg, 0.30 mmol), **3f** (15.2 mg, 15 μmol), $Cu(OAc)_2$ (114 mg, 0.63 mmol), and alkyne **2a** (56 μL, 0.45 mmol) for a reaction time of 8 h and was purified by trituration with Et_2O to give a white solid (70.2 mg, 65%). R_f 0.45 (50% EtOAc/petrol); $[\alpha]_D^{20}$ +135.0 (*c* 1.00, $CHCl_3$); m.p. 182−183 °C (CH_2Cl_2); IR 1776 (C=O), 1673 (C=O), 1524, 1360, 1249, 1127, 987, 801, 762, 739 cm^{-1}; ^1H NMR (400 MHz, $CDCl_3$) δ 8.21 (1H, d, *J* = 2.0 Hz, Ar**H**), 8.15 (1H, dd, *J* = 8.2, 2.0 Hz, Ar**H**), 7.46–7.36 (4H, m, Ar**H**), 7.33–7.27 (2H, m, Ar**H**), 5.87 (1H, q, *J* = 0.8 Hz, CH_3C=C**H**), 2.28 (3H, s, C**H**$_3$), 2.25 (3H, d, *J* = 0.8 Hz, C**H**$_3$); ^{13}C NMR (100.6 MHz, $CDCl_3$) δ 187.4 (C), 168.9 (C), 165.1 (C), 149.1 (C), 148.4 (C), 147.2 (C), 143.2 (C), 140.6 (C), 133.0 (C), 128.9 (2 × CH), 128.7 (2 × CH), 128.6 (CH), 121.94 (CH), 121.89 (CH), 115.7 (CH), 107.1 (CH), 74.6 (C), 20.5 (CH_3), 11.8 (CH_3); HRMS (ESI +ve) Exact mass calculated for $C_{21}H_{15}NNaO_5$ $[M+Na]^+$: 384.0842, found: 384.0836; Enantiomeric excess was determined by HPLC with a Chiralpak AD-H column (95:5 *iso*-hexane:*iso*-PrOH, 0.8 mL/min, 280 nm, 25 °C); t_r (major) = 39.0 min, t_r (minor) = 46.7 min; 94% ee.

**Data file:**	C:\CHEM32\1\DATA\DAVE\DEF_LC 2015-04-22 07-24-47\DJB7-56-RAC.D
**Sample name:**	DJB7-56-Rac
**Instrument:**	AGILENT 1260
**Injection date:**	4/22/2015 7:37:12 AM
**Acq. method:**	ADH95B05A.50MIN.0.8 ML.M

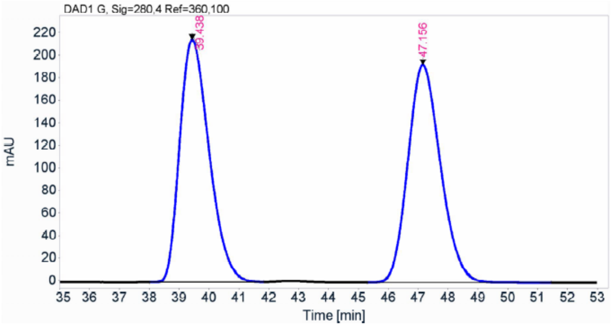

Signal: DAD1 G, Sig=280,4 Ref=360,100

RT [min]	Type	Width [min]	Area	Height	Area%
39.438	BB	1.0697	14735.232	214.9780	50.05
47.156	BB	1.1777	14708.007	193.2951	49.95

**Data file:**	C:\CHEM32\1\DATA\DAVE\DEF_LC 2015-04-22 07-24-47\DJB7-56-1.D
**Sample name:**	DJB7-56-1
**Instrument:**	AGILENT 1260
**Injection date:**	4/22/2015 8:38:17 AM
**Acq. method:**	ADH95B05A.50MIN.0.8 ML.M

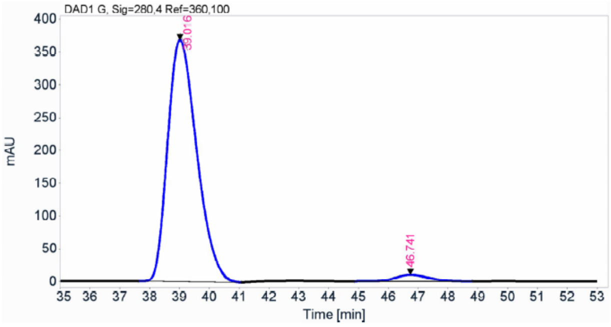

Signal: DAD1 G, Sig=280,4 Ref=360,100

RT [min]	Type	Width [min]	Area	Height	Area%
39.016	BB	1.0578	24974.324	368.9200	96.91
46.741	BB	1.1484	797.534	10.4975	3.09

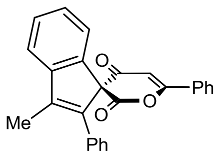

(*S*)-3-Methyl-2,6'-diphenyl-2',4'-dihydrospiro[indene-1,3'-pyran]-2',4'-dione (4j). The title compound was prepared according to the General Procedure from substrate **1e** (52.8 mg, 0.20 mmol), **3f** (10.1 mg, 10 μmol), $Cu(OAc)_2$ (76.3 mg, 0.42 mmol), and alkyne **2a** (38 µL, 0.30 mmol) for a reaction time of 4 h and was purified by flash column chromatography (10% acetone/petrol) to give a light yellow amorphous solid (56 mg, 74%). R_f 0.39 (30% acetone/petrol); $[\alpha]_D^{20}$ +9.3 (*c* 1.00, $CHCl_3$); m.p. 186–187 °C (acetone/petrol); IR 1774 (C=O), 1676 (C=O), 1621, 1597, 1466, 1331, 1245, 1092, 754 cm^{-1}; ^1H NMR (400 MHz, $CDCl_3$) δ 7.97–7.91 (2H, m, Ar**H**), 7.62 (1H, ddd, *J* = 7.3, 3.7, 1.1 Hz, Ar**H**), 7.58–7.51 (2H, m, Ar**H**), 7.45–7.35 (6H, m, Ar**H**), 7.34–7.28 (2H, m, Ar**H**), 7.24–7.17 (1H, m, Ar**H**), 6.56 (1H, s, CPh=C**H**), 2.32 (3H, s, C**H**$_3$); ^{13}C NMR (100.6 MHz, $CDCl_3$) δ 189.4 (C), 166.1 (C), 164.8 (C), 146.3 (C), 142.1 (C), 141.4 (C), 139.7 (C), 134.4 (C), 133.0 (CH), 129.7 (C), 129.4 (CH), 129.2 (2 × CH), 129.1 (2 × CH), 128.5 (2 × CH), 127.8 (CH), 126.8 (2 × CH), 126.5 (CH), 121.1 (CH), 120.9 (CH), 103.5 (CH), 75.0 (C), 12.1 (CH_3); HRMS (ESI) Exact mass calculated for $C_{26}H_{18}NaO_3$ $[M+Na]^+$: 401.1148, found: 401.1150. Enantiomeric excess was determined by HPLC with a Chiralpak IC column (98:2 *iso*-hexane:*iso*-PrOH, 0.8 mL/min, 210 nm, 25 °C); t_r (minor) = 40.0 min, t_r (major) = 47.8 min; 94% ee.

Data file: C:\CHEM32\1\DATA\IK\DEF_LC 2015-04-15 15-40-46\IK-755C.D
Sample name: IK-755c
Instrument: AGILENT 1260
Injection date: 4/15/2015 4:08:17 PM
Acq. method: IC98B02A.60MIN.0.8M L.M

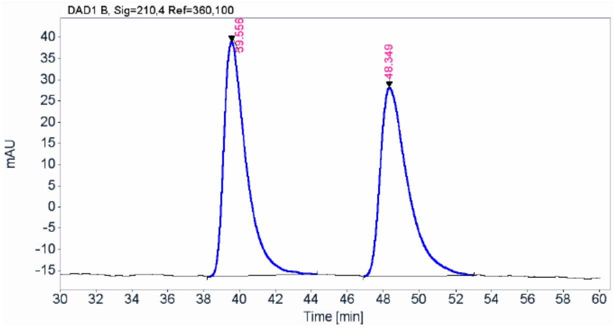

Signal: DAD1 B, Sig=210,4 Ref=360,100

RT [min]	Type	Width [min]	Area	Height	Area%
39.556	BB	1.2757	4775.345	55.1749	50.24
48.349	BB	1.5665	4729.481	44.4096	49.76

Data file: C:\CHEM32\1\DATA\IK\DEF_LC 2015-04-16 08-31-54\IK-756B.D
Sample name: IK-756b
Instrument: AGILENT 1260
Injection date: 4/16/2015 3:55:30 PM
Acq. method: IC98B02A.60MIN.0.8M L.M

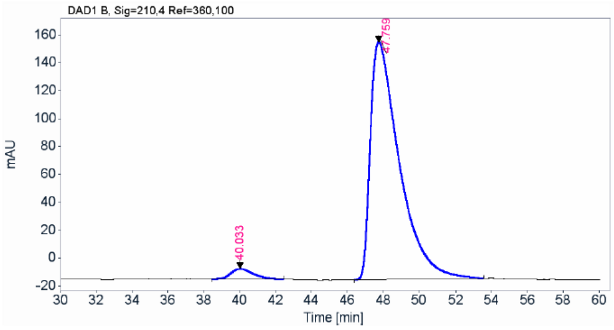

Signal: DAD1 B, Sig=210,4 Ref=360,100

RT [min]	Type	Width [min]	Area	Height	Area%
40.033	BB	1.1501	627.056	7.3444	3.18
47.759	BB	1.6496	19066.197	170.1091	96.82

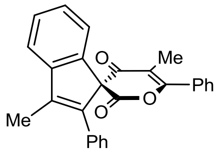

(*S*)-3,5'-Dimethyl-2,6'-diphenyl-2',4'-dihydrospiro[indene-1,3'-pyran]-2',4'-dione (4k). The title compound was prepared according to the General Procedure from substrate **1f** (55.6 mg, 0.20 mmol), **3f** (10.1 mg, 10 µmol), $Cu(OAc)_2$ (76.3 mg, 0.42 mmol), and alkyne **2a** (38 µL, 0.30 mmol) for a reaction time of 4 h and was purified by flash column chromatography (10% acetone/petrol) to give a light yellow amorphous solid (73.4 mg, 94%). R_f 0.44 (30% acetone/petrol); $[\alpha]_D^{20}$ +5.8 (*c* 1.50, $CHCl_3$); m.p. 158–159 °C (acetone/petrol); IR 1774 (C=O), 1673 (C=O), 1626, 1493, 1343, 1099, 1010, 751 cm^{-1}; ^1H NMR (400 MHz, $CDCl_3$) δ 7.66–7.61 (2H, m, Ar**H**), 7.55–7.48 (3H, m, Ar**H**), 7.46–7.43 (2H, m, Ar**H**), 7.42–7.31 (6H, m, Ar**H**), 7.26 (1H, ddd, *J* = 7.6, 5.7, 3.2 Hz, Ar**H**), 2.26 (3H, s, C**H**$_3$), 2.00 (3H, s, C**H**$_3$); ^{13}C NMR (100.6 MHz, $CDCl_3$) δ 190.4 (C), 166.3 (C), 161.7 (C), 146.3 (C), 141.7 (C), 141.2 (C), 139.9 (C), 134.5 (C), 131.5 (C), 131.1 (CH), 129.3 (2 × CH), 129.2 (CH), 128.8 (2 × CH), 128.4 (4 × CH), 127.9 (CH), 126.5 (CH), 121.2 (CH), 121.1 (CH), 113.6 (C), 75.0 (C), 11.9 (CH_3), 11.8 (CH_3); HRMS (ESI +ve) Exact mass calculated for $C_{27}H_{20}NaO_3$ $[M+Na]^+$: 415.1305, found: 415.1313. Enantiomeric excess was determined by HPLC with a Chiralpak OD-H column (98:2 *iso*-hexane:*iso*-PrOH, 0.8 mL/min, 280 nm, 25 °C); t_r (minor) = 20.3 min, t_r (major) = 25.3 min; 95% ee.

Data file: C:\CHEM32\1\DATA\IK\DEF_LC 2015-04-15 17-31-36\IK-755A.D
Sample name: IK-755a
Instrument: AGILENT 1260
Injection date: 4/15/2015 8:42:21 PM
Acq. method: ODH98B02A.0.8ML.45 MIN.M

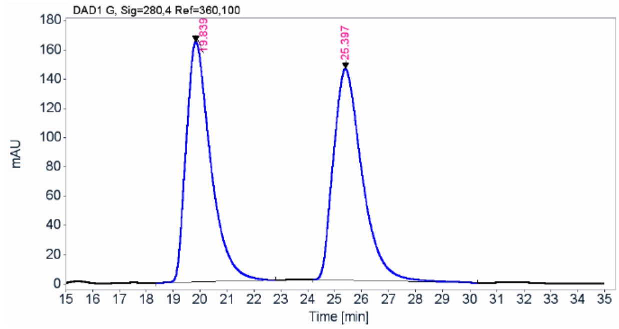

Signal: DAD1 G, Sig=280,4 Ref=360,100

RT [min]	Type	Width [min]	Area	Height	Area%
19.839	BB	0.9845	10388.679	164.1196	49.23
25.397	BB	1.1645	10713.565	144.5744	50.77

Data file: C:\CHEM32\1\DATA\IK\DEF_LC 2015-04-16 08-31-54\IK-756A.D
Sample name: IK-756a
Instrument: AGILENT 1260
Injection date: 4/16/2015 2:48:40 PM
Acq. method: ODH98B02A.0.8ML.45 MIN.M

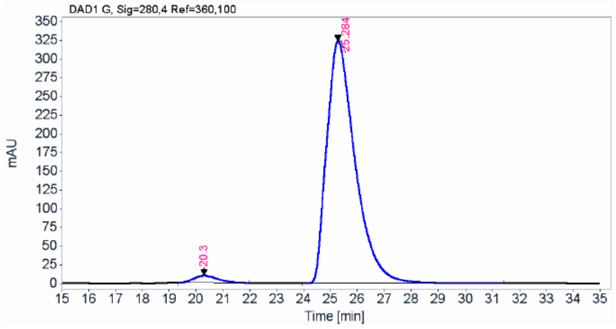

Signal: DAD1 G, Sig=280,4 Ref=360,100

RT [min]	Type	Width [min]	Area	Height	Area%
20.300	BB	0.9494	583.220	9.5114	2.40
25.284	BB	1.0723	23750.174	323.5766	97.60

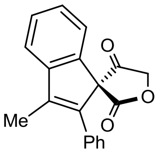

(*S*)-3-Methyl-2-phenylspiro[indene-1,3'-oxolane]-2',4'-dione (4l). The title compound was prepared according to the General Procedure from substrate **1g** (35.2 mg, 0.20 mmol), **3f** (10.1 mg, 10 µmol), $Cu(OAc)_2$ (76.3 mg, 0.42 mmol), and alkyne **2a** (38 µL, 0.30 mmol) for a reaction time of 12 h and was purified by flash column chromatography (10% acetone/petrol) to give an off-white amorphous solid (13 mg, 22%). R_f 0.45 (30% acetone/petrol); $[\alpha]_D^{20}$ –9.8 (*c* 1.00, $CHCl_3$); m.p. 142–143 °C (acetone/petrol); IR 1753 (C=O), 1676 (C=O), 1621, 1598, 1470, 1330, 1255, 1049, 757 cm^{-1}; ^{1}H NMR (500 MHz, $CDCl_3$) δ 7.48–7.43 (2H, m, Ar**H**), 7.42–7.38 (2H, m, Ar**H**), 7.37–7.34 (1H, m, Ar**H**), 7.30–7.25 (3H, m, Ar**H**), 7.20 (1H, dt, *J* = 7.5, 0.9 Hz, Ar**H**), 4.90 (1H, d, *J* = 17.1 Hz, C**H**$_2$C=O), 4.54 (1H, d, *J* = 17.1 Hz, C**H**$_2$C=O), 2.21 (3H, s, C**H**$_3$); ^{13}C NMR (125.8 MHz, $CDCl_3$) δ 205.3 (C), 172.3 (C), 146.9 (C), 143.0 (C), 140.3 (C), 137.8 (C), 133.6 (C), 129.3 (CH), 129.0 (2 × CH), 128.7 (2 × CH), 128.4 (CH), 126.9 (CH), 121.5 (CH), 121.0 (CH), 74.1 (CH_2), 68.7 (C), 11.7 (CH_3); HRMS (ESI +ve) Exact mass calculated for $C_{19}H_{14}NaO_3$ $[M+Na]^+$: 313.0835, found: 313.0822. Enantiomeric excess was determined by HPLC with a Chiralpak AD-H column (95:5 *iso*-hexane:*iso*-PrOH, 0.8 mL/min, 254 nm, 25 °C); t_r (minor) = 18.3 min, t_r (major) = 19.8 min; 90% ee.

Data file: C:\CHEM32\1\DATA\IK\DEF_LC 2015-04-02 08-40-11\IK-749II.D
Sample name: IK-749II
Instrument: AGILENT 1260
Injection date: 4/2/2015 10:34:18 AM
Acq. method: ADH95B05A.50MIN.0.8 ML.M

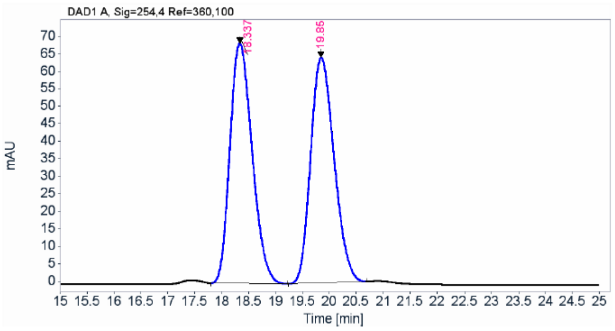

Signal: DAD1 A, Sig=254,4 Ref=360,100

RT [min]	Type	Width [min]	Area	Height	Area%
18.337	BB	0.4485	1964.738	68.5341	50.20
19.850	BB	0.4729	1949.075	64.4289	49.80

Data file: C:\CHEM32\1\DATA\CC\DEF_LC 2015-04-02 14-45-34\IK-749A.D
Sample name: IK-749a
Instrument: AGILENT 1260
Injection date: 4/2/2015 4:02:02 PM
Acq. method: ADH95B05A.50MIN.0.8 ML.M

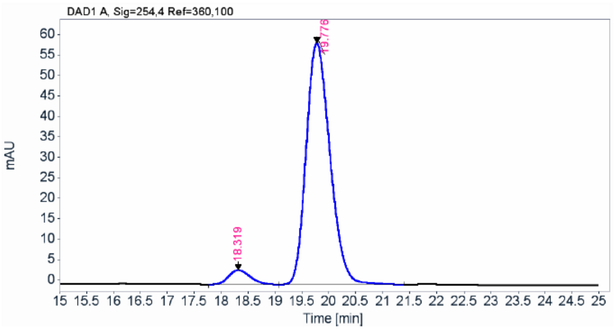

Signal: DAD1 A, Sig=254,4 Ref=360,100

RT [min]	Type	Width [min]	Area	Height	Area%
18.319	BB	0.4399	98.206	3.4944	5.14
19.776	BB	0.4766	1812.219	58.9621	94.86

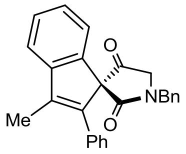

(*S*)-1'-Benzyl-3-methyl-2-phenylspiro[indene-1,3'-pyrrolidine]-2',4'-dione (4m). The title compound was prepared according to the General Procedure from substrate **1h** (53.0 mg, 0.20 mmol), **3f** (10.1 mg, 10 µmol), $Cu(OAc)_2$ (76.3 mg, 0.42 mmol), and alkyne **2a** (38 µL, 0.30 mmol) for a reaction time of 1 h and was purified by flash column chromatography (10% acetone/petrol) to give an off-white solid (60.6 mg, 80%). R_f 0.45 (30% acetone/petrol); $[\alpha]_D^{20}$ –7.5 (*c* 1.70, $CHCl_3$); m.p. 154–155 °C (acetone/petrol); IR 1753 (C=O), 1689 (C=O), 1470, 1255, 1049, 751 cm^{-1}; ^1H NMR (400 MHz, $CDCl_3$) δ 7.42–7.38 (2H, m, Ar**H**), 7.37–7.30 (6H, m, Ar**H**), 7.29–7.25 (2H, m, Ar**H**), 7.22 (1H, ddd, *J* = 7.5, 4.9, 3.7 Hz, Ar**H**), 7.15–7.07 (3H, m, Ar**H**), 4.87 (1H, d, *J* = 14.6 Hz, NC**H**$_2$Ph), 4.61 (1H, d, *J* = 14.6 Hz, NC**H**$_2$Ph), 3.95 (1H, d, *J* = 17.8 Hz, NC**H**$_2$C=O), 3.64 (1H, d, *J* = 17.8 Hz, NC**H**$_2$C=O), 2.21 (s, 3H, C**H**$_3$); ^{13}C NMR (100.6 MHz, $CDCl_3$) δ 205.2 (C), 170.6 (C), 147.1 (C), 141.9 (C), 141.8 (C), 139.1 (C), 134.8 (C), 134.5 (C), 128.9 (2 × CH), 128.8 (2 × CH), 128.63 (CH), 128.61 (2 × CH), 128.2 (2 × CH), 128.0 (CH), 127.9 (CH), 126.4 (CH), 121.2 (CH), 120.6 (CH), 72.3 (C), 55.9 (CH_2), 46.4 (CH_2), 11.6 (CH_3); HRMS (ESI +ve) Exact mass calculated for $C_{26}H_{21}NNaO_2$ $[M+Na]^+$: 402.1465, found: 402.1461. Enantiomeric excess was determined by HPLC with a Chiralpak IC column (90:10 *iso*-hexane:*iso*-PrOH, 1.0 mL/min, 210 nm, 25 °C); t_r (major) = 18.3 min, t_r (minor) = 27.0 min; 84% ee.

Data file: C:\CHEM32\1\DATA\IK\DEF_LC 2015-03-23 14-00-36\IK-741A-IC.D
Sample name: IK-741a
Instrument: AGILENT 1260
Injection date: 3/23/2015 5:13:31 PM
Acq. method: IC90B10A.70MIN.1.0M
L.M

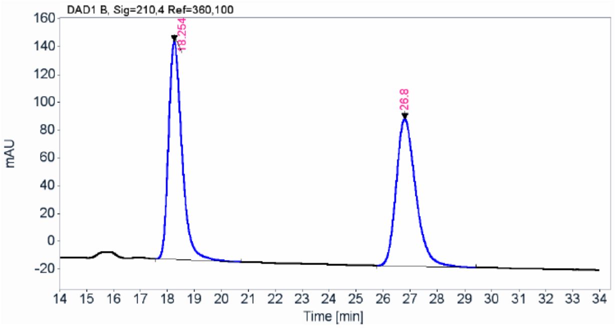

Signal: DAD1 B, Sig=210,4 Ref=360,100

RT [min]	Type	Width [min]	Area	Height	Area%
18.254	BB	0.5252	5399.629	157.0319	50.24
26.800	BB	0.7735	5348.754	105.8526	49.76

Data file: C:\CHEM32\1\DATA\IK\DEF_LC 2015-03-26 14-27-39\IK-745A.D
Sample name: IK-745a
Instrument: AGILENT 1260
Injection date: 3/26/2015 2:44:45 PM
Acq. method: IC90B10A.70MIN.1.0M
L.M

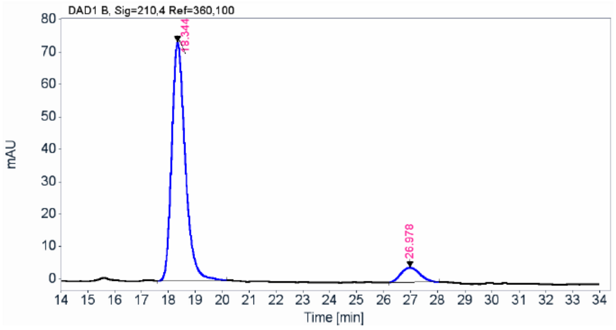

Signal: DAD1 B, Sig=210,4 Ref=360,100

RT [min]	Type	Width [min]	Area	Height	Area%
18.344	BB	0.5294	2557.144	73.5980	92.01
26.978	BB	0.6728	221.993	4.5374	7.99

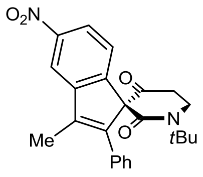

(*S*)-1'-*tert*-Butyl-3-methyl-5-nitro-2-phenylspiro[indene-1,3'-piperidine]-2',4'-dione (4n). The title compound was prepared according to the General Procedure from substrate **1i** (58.0 mg, 0.20 mmol), **3f** (10.1 mg, 10 μmol), $Cu(OAc)_2$ (76.3 mg, 0.42 mmol), and alkyne **2a** (38 μL, 0.30 mmol) for a reaction time of 1 h and was purified by flash column chromatography (10% acetone/petrol) to give a light yellow amorphous solid (64.5 mg, 80%). R_f 0.40 (30% acetone/petrol); $[\alpha]_D^{20}$ –9.4 (*c* 1.70, $CHCl_3$); m.p. 103–104 °C (acetone/petrol); IR 1731 (C=O), 1644 (C=O), 1522, 1463, 1339, 1196, 802, 738 cm^{-1}; ^1H NMR (400 MHz, $CDCl_3$) δ 8.15 (1H, d, *J* = 1.7 Hz, Ar**H**), 8.12 (1H, dd, *J* = 8.1, 2.2 Hz, Ar**H**), 7.42–7.35 (3H, m, Ar**H**), 7.30–7.23 (3H, m, Ar**H**), 3.66 (1H, ddd, *J* = 12.7, 7.5, 4.9 Hz, NC**H**$_2$), 3.35 (1H, ddd, *J* = 13.0, 8.1, 4.7 Hz, NC**H**$_2$), 2.83 (1H, ddd, *J* = 15.0, 8.1, 4.9 Hz, C**H**$_2$C=O), 2.65–2.54 (1H, m, C**H**$_2$C=O), 2.16 (3H, s, =CC**H**$_3$), 1.42 (9H, s, C(C**H**$_3$)$_3$); ^{13}C NMR (100.6 MHz, $CDCl_3$) δ 203.1 (C), 166.8 (C), 151.5 (C), 149.2 (C), 148.4 (C), 145.6 (C), 139.2 (C), 134.3 (C), 129.0 (2 × CH), 128.5 (2 × CH), 128.4 (CH), 121.7 (CH), 121.5 (CH), 115.2 (CH), 78.1 (C), 59.1 (C), 41.3 (CH_2), 38.6 (CH_2), 28.0 (CH_3), 11.4 (CH_3); HRMS (ESI +ve) Exact mass calculated for $C_{24}H_{24}N_2NaO_4$ $[M+Na]^+$: 427.1628, found: 428.1641. Enantiomeric excess was determined by HPLC with a Chiralpak IC column (90:10 *iso*-hexane:*iso*-PrOH, 1.0 mL/min, 254 nm, 25 °C); t_r (minor) = 33.4 min, t_r (major) = 36.7 min; 97% ee.

Data file: C:\CHEM32\1\DATA\IK\DEF_LC 2015-05-14 17-25-56\IK-768B2.D
Sample name: IK-768b2
Instrument: AGILENT 1260
Injection date: 5/14/2015 5:43:13 PM
Acq. method: IC90B10A.70MIN.1.0M L.M

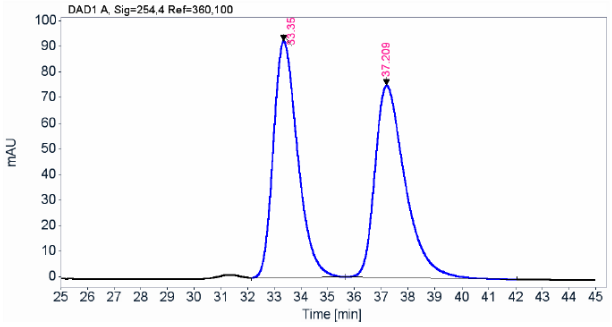

Signal: DAD1 A, Sig=254,4 Ref=360,100

RT [min]	Type	Width [min]	Area	Height	Area%
33.350	BB	0.9716	5799.638	92.2376	49.68
37.209	BB	1.1823	5873.966	74.9507	50.32

Data file: C:\CHEM32\1\DATA\IK\DEF_LC 2015-05-15 08-22-50\IK-768D2.D
Sample name: IK-768d2
Instrument: AGILENT 1260
Injection date: 5/15/2015 8:39:56 AM
Acq. method: IC90B10A.70MIN.1.0M L.M

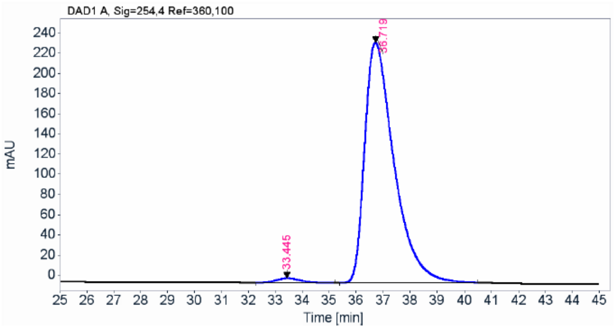

Signal: DAD1 A, Sig=254,4 Ref=360,100

RT [min]	Type	Width [min]	Area	Height	Area%
33.445	BB	0.9605	282.049	4.4438	1.54
36.719	BB	1.1409	18007.910	237.9591	98.46

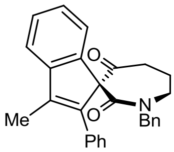

(*S*)-1-Benzyl-3'-methyl-2'-phenylspiro[azepane-3,1'-indene]-2,4-dione (4o). The title compound was prepared according to the General Procedure from substrate **1j** (58.6 mg, 0.20 mmol), **3f** (10.1 mg, 10 µmol), $Cu(OAc)_2$ (76.3 mg, 0.42 mmol), and alkyne **2a** (38 µL, 0.30 mmol) for a reaction time of 1 h and was purified by flash column chromatography (10% acetone/petrol) to give an off-white solid (59.4 mg, 73%). R_f 0.51 (30% acetone/petrol); $[\alpha]_D^{20}$ –2.5 (*c* 1.30, $CHCl_3$); m.p. 178–179 °C (acetone/petrol); IR 1713 (C=O), 1627 (C=O), 1600, 1415, 1365, 1216, 1006, 934, 755, 704 cm^{-1}; ^1H NMR (400 MHz, $CDCl_3$) δ 7.43 (1H, d, *J* = 7.5 Hz, Ar**H**), 7.41–7.36 (2H, m, Ar**H**), 7.35–7.30 (5H, m, Ar**H**), 7.29–7.25 (2H, m, Ar**H**), 7.25–7.19 (4H, m, Ar**H**), 4.84 (1H, d, *J* = 14.2 Hz, NC**H**$_2$Ph), 4.52 (1H, d, *J* = 14.2 Hz, NC**H**$_2$Ph), 3.15 (1H, dt, *J* = 15.8, 4.9 Hz, NC**H**$_2$CH$_2$), 3.06–2.89 (2H, m, NC**H**$_2$CH$_2$ and C**H**$_2$C=O), 2.51 (1H, ddd, *J* = 11.4, 8.3, 3.5 Hz, C**H**$_2$C=O), 2.00 (3H, s, C**H**$_3$), 1.94–1.80 (1H, m, CH$_2$C**H**$_2$CH$_2$), 1.77–1.65 (1H, m, CH$_2$C**H**$_2$CH$_2$); ^{13}C NMR (100.6 MHz, $CDCl_3$) δ 206.1 (C), 169.3 (C), 146.4 (C), 142.8 (C), 141.3 (C), 141.1 (C), 137.1 (C), 135.6 (C), 129.8 (2 × CH), 128.8 (2 × CH), 128.6 (2 × CH), 128.3 (CH), 128.1 (2 × CH), 127.7 (CH), 127.7 (CH), 126.2 (CH), 122.2 (CH), 120.6 (CH), 80.2 (C), 51.6 (CH_2), 44.1 (CH_2), 38.3 (CH_2), 25.5 (CH_2), 11.2 (CH_3); HRMS (ESI +ve) Exact mass calculated for $C_{28}H_{25}NNaO_2$ $[M+Na]^+$: 430.1778, found: 430.1773. Enantiomeric excess was determined by HPLC with a Chiralpak OD-H column (95:5 *iso*-hexane:*iso*-PrOH, 0.8 mL/min, 230 nm, 25 °C); t_r (minor) = 45.8 min, t_r (major) = 52.7 min; 94% ee.

Data file: C:\CHEM32\1\DATA\IK\DEF_LC 2015-03-26 09-10-05\IK-741B3.D
Sample name: IK-741b3
Instrument: AGILENT 1260
Injection date: 3/26/2015 11:07:56 AM
Acq. method: ODH95B05A.0.8ML.60 MIN.M

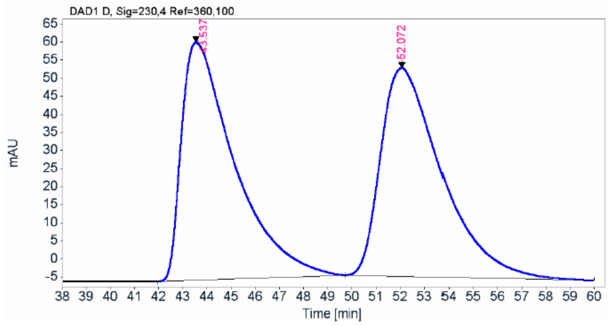

Signal: DAD1 D, Sig=230,4 Ref=360,100

RT [min]	Type	Width [min]	Area	Height	Area%
43.537	BB	2.1792	10019.199	65.7717	49.92
52.072	BBA	2.3999	10050.245	57.6486	50.08

Data file: C:\CHEM32\1\DATA\IK\DEF_LC 2015-03-31 15-01-12\IK-745BR.D
Sample name: IK-745bR
Instrument: AGILENT 1260
Injection date: 3/31/2015 3:13:20 PM
Acq. method: ODH95B05A.0.8ML.60 MIN.M

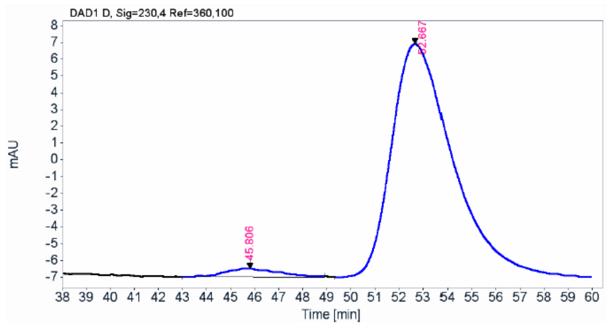

Signal: DAD1 D, Sig=230,4 Ref=360,100

RT [min]	Type	Width [min]	Area	Height	Area%
45.806	MM	2.6228	78.937	0.5016	3.07
52.667	MM	2.9838	2495.817	13.9411	96.93

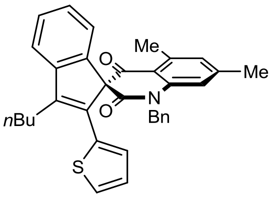

(*S*)-1'-Benzyl-3-butyl-5',7'-dimethyl-2-(thiophen-2-yl)-2',4'-dihydro-1'*H*-spiro[indene-1,3'-quinoline]-2',4'-dione (4p). The title compound was prepared according to the General Procedure from substrate **1k** (71 mg, 0.20 mmol), **3f** (10.1 mg, 10 µmol), $Cu(OAc)_2$ (76.3 mg, 0.42 mmol), and alkyne **2f** (49.2 mg, 0.30 mmol) for a reaction time of 4 h and was purified by flash column chromatography (6% acetone/petrol) to give a yellow solid (87 mg, 84%). R_f 0.35 (20% acetone/petrol); $[\alpha]_D^{20}$ +0.7 (*c* 1.30, $CHCl_3$); m.p. 137–138 °C (acetone/petrol); IR 1763 (C=O), 1661 (C=O), 1621, 1572, 1454, 1317, 1264, 1189, 762 cm^{-1}; ^1H NMR (400 MHz, $CDCl_3$) δ 7.42 (1H, d, *J* = 7.3 Hz, Ar**H**), 7.35 (1H, dd, *J* = 7.5, 1.0 Hz, Ar**H**), 7.34–7.31 (2H, m, Ar**H**), 7.30 (1H, s, Ar**H**), 7.29–7.23 (3H, m, Ar**H**), 7.07–7.03 (1H, m, Ar**H**), 7.02–7.00 (1H, m, Ar**H**), 6.99 (1H, s, Ar**H**), 6.94 (1H, d, *J* = 7.4 Hz, Ar**H**), 6.91 (1H, dd, *J* = 3.7, 1.0 Hz, Ar**H**), 6.83 (1H, br s, Ar**H**), 5.51 (1H, d, *J* = 15.9 Hz, NC**H**$_2$Ph), 5.16 (1H, d, *J* = 15.9 Hz, NC**H**$_2$Ph), 3.08–2.93 (2H, m, C**H**$_2$CH$_2$CH$_2$CH$_3$), 2.50 (3H, s, ArC**H**$_3$), 2.34 (3H, s, ArC**H**$_3$), 1.88–1.76 (2H, m, C**H**$_2$CH$_2$CH$_3$), 1.68–1.54 (2H, m, C**H**$_2$CH$_3$), 1.03 (3H, t, *J* = 7.3 Hz, CH$_2$C**H**$_3$); ^{13}C NMR (100.6 MHz, $CDCl_3$) δ 191.0 (C), 168.4 (C), 146.0 (C), 145.6 (C), 144.3 (C), 143.9 (C), 143.3 (C), 141.7 (C), 137.5 (C), 136.4 (C), 134.9 (C), 128.8 (3 × CH), 128.1 (CH), 127.3 (CH), 126.9 (CH), 126.7 (2 × CH), 126.6 (CH), 125.7 (CH), 125.4 (CH), 121.1 (CH), 120.9 (CH), 118.2 (C), 114.8 (CH), 77.2 (C), 47.1 (CH_2), 30.4 (CH_2), 26.6 (CH_2), 23.1 (CH_2), 22.6 (CH_3), 22.2 (CH_3), 14.0 (CH_3); HRMS (ESI +ve) Exact mass calculated for $C_{34}H_{31}NNaO_2S$ $[M+Na]^+$: 540.1968, found: 540.1960. Enantiomeric excess was determined by HPLC with a Chiralpak IC column (95:5 *iso*-hexane:*iso*-PrOH, 0.8 mL/min, 254 nm, 25 °C); t_r (major) = 18.9 min, t_r (minor) = 24.4 min; 68% ee.

Data file: C:\CHEM32\1\DATA\DAVE\DEF_LC 2015-04-22 07-24-47\IK-767III.D
Sample name: IK-767III
Instrument: AGILENT 1260
Injection date: 4/22/2015 10:21:03 PM
Acq. method: IC95B05A.60MIN.0.8ML.M

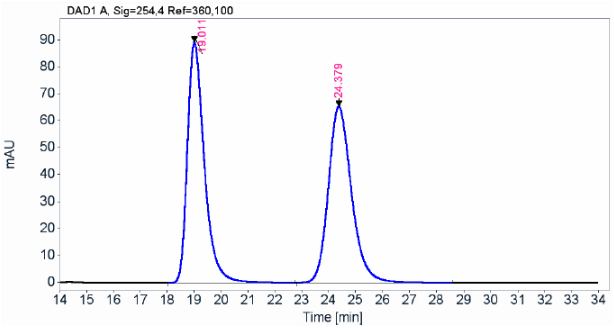

Signal: DAD1 A, Sig=254,4 Ref=360,100

RT [min]	Type	Width [min]	Area	Height	Area%
19.011	BB	0.6665	3911.505	89.3907	49.88
24.379	BB	0.9179	3930.231	65.5125	50.12

Data file: C:\CHEM32\1\DATA\DAVE\DEF_LC 2015-04-23 07-37-38\IK-767A2.D
Sample name: IK-767a2
Instrument: AGILENT 1260
Injection date: 4/23/2015 1:59:10 PM
Acq. method: IC95B05A.60MIN.0.8ML.M

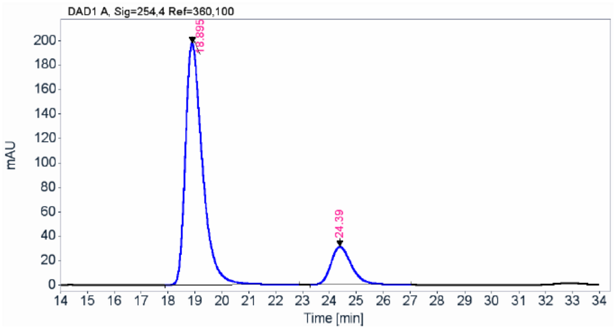

Signal: DAD1 A, Sig=254,4 Ref=360,100

RT [min]	Type	Width [min]	Area	Height	Area%
18.895	BB	0.6688	8688.475	197.6535	84.02
24.390	BB	0.8131	1652.504	31.0412	15.98

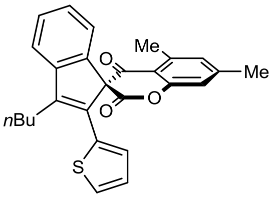

(*S*)-3'-Butyl-5,7-dimethyl-2'-(thiophen-2-yl)-2,4-dihydrospiro[1-benzopyran-3,1'-indene]-2,4-dione (4q). The title compound was prepared according to the General Procedure from substrate **1l** (53.2 mg, 0.20 mmol), **3f** (10.1 mg, 10 µmol), $Cu(OAc)_2$ (76.3 mg, 0.42 mmol), and alkyne **2f** (49.2 mg, 0.30 mmol) for a reaction time of 4 h and was purified by flash column chromatography (7% acetone/petrol) to give a yellow solid (57 mg, 66%). R_f 0.53 (30% acetone/petrol); $[\alpha]_D^{20}$ +1.7 (*c* 1.40, $CHCl_3$); m.p. 150–151 °C (acetone/petrol); IR 1765 (C=O), 1683 (C=O), 1618, 1569, 1455, 1266, 1186, 1067, 761, 703 cm^{-1}; ^1H NMR (400 MHz, $CDCl_3$) δ 7.43–7.39 (1H, m, Ar**H**), 7.36 (1H, td, *J* = 7.5, 1.0 Hz, Ar**H**), 7.31 (1H, dd, *J* = 2.1, 1.1 Hz, Ar**H**), 7.10–7.03 (2H, m, Ar**H**), 7.00 (1H, dd, *J* = 5.1, 3.7 Hz, Ar**H**), 6.99–6.92 (2H, m, Ar**H**), 6.85 (1H, dd, *J* = 3.7, 1.0 Hz, Ar**H**), 3.02–2.89 (2H, m, C**H**$_2$CH$_2$CH$_2$CH$_3$), 2.54 (3H, s, ArC**H**$_3$), 2.48 (3H, s, ArC**H**$_3$), 1.84–1.72 (2H, m, C**H**$_2$CH$_2$CH$_3$), 1.64–1.51 (2H, m, C**H**$_2$CH$_3$), 1.01 (3H, t, *J* = 7.3 Hz, CH$_2$C**H**$_3$); ^{13}C NMR (100.6 MHz, $CDCl_3$) δ 187.7 (C), 165.5 (C), 156.1 (C), 147.8 (C), 145.6 (C), 145.3 (C), 143.1 (C), 140.6 (C), 136.6 (C), 133.4 (C), 129.5 (CH), 129.3 (CH), 127.1 (CH), 126.6 (CH), 126.2 (CH), 125.8 (CH), 121.2 (CH), 121.1 (CH), 116.0 (CH), 115.4 (C), 75.6 (C), 30.3 (CH_2), 26.6 (CH_2), 23.1 (CH_2), 21.93 (CH_3), 21.91 (CH_3), 14.0 (CH_3); HRMS (ESI +ve) Exact mass calculated for $C_{27}H_{24}NaO_3S$ $[M+Na]^+$: 451.1338, found: 451.1340. Enantiomeric excess was determined by HPLC with a Chiralpak AD-H column (95:5 *iso*-hexane:*iso*-PrOH, 0.8 mL/min, 254 nm, 25 °C); t_r (minor) = 11.4 min, t_r (major) = 22.7 min; 81% ee.

Data file: C:\CHEM32\1\DATA\IK\DEF_LC 2015-04-06 15-56-25\IK-752B.D
Sample name: IK-752b
Instrument: AGILENT 1260
Injection date: 4/6/2015 5:50:29 PM
Acq. method: ADH95B05A.50MIN.0.8 ML.M

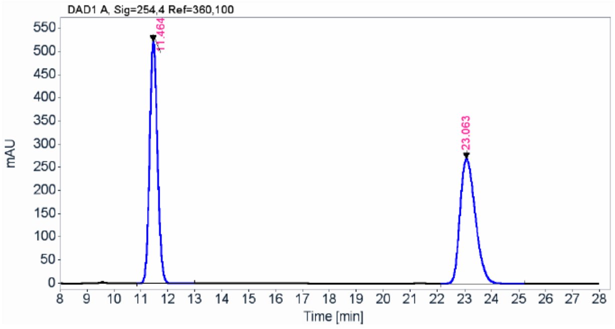

Signal: DAD1 A, Sig=254,4 Ref=360,100

RT [min]	Type	Width [min]	Area	Height	Area%
11.464	BB	0.3126	10593.686	522.2654	49.97
23.063	BB	0.6190	10607.825	269.6021	50.03

Data file: C:\CHEM32\1\DATA\IK\DEF_LC 2015-04-17 08-59-28\IK-753IIR.D
Sample name: IK-753IIR
Instrument: AGILENT 1260
Injection date: 4/18/2015 2:09:59 AM
Acq. method: ADH95B05A.50MIN.0.8 ML.M

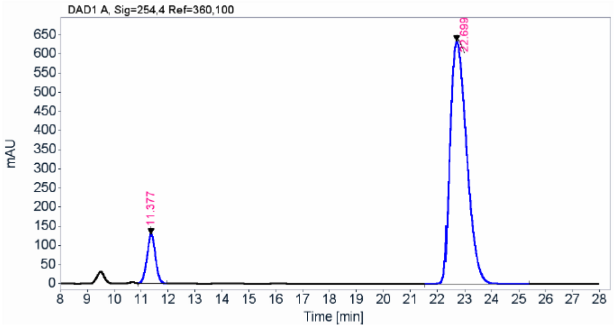

Signal: DAD1 A, Sig=254,4 Ref=360,100

RT [min]	Type	Width [min]	Area	Height	Area%
11.377	VB	0.3354	2830.329	129.2778	9.54
22.699	BB	0.6723	26842.902	633.5305	90.46

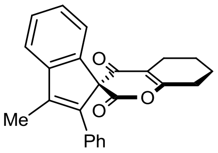

(*S*)-3'-Methyl-2'-phenyl-2,4,5,6,7,8-hexahydrospiro[1-benzopyran-3,1'-indene]-2,4-dione (4r). The title compound was prepared according to the General Procedure from substrate **1m** (48.4 mg, 0.20 mmol), **3f** (10.1 mg, 10 *µ*mol), $Cu(OAc)_2$ (76.3 mg, 0.42 mmol), and alkyne **2a** (38 µL, 0.30 mmol) for a reaction time of 12 h and was purified by flash column chromatography (7% acetone/petrol) to give an off-white amorphous solid (58 mg, 82%). R_f 0.56 (30% acetone/petrol); $[\alpha]_D^{20}$ +3.5 (*c* 1.00, $CHCl_3$); m.p. 199–200 °C (acetone/petrol); IR 1770 (C=O), 1674 (C=O), 1641, 1379, 1259, 1127, 1035, 945, 759 cm^{-1}; ^{1}H NMR (400 MHz, $CDCl_3$) δ 7.46–7.36 (4H, m, Ar**H**), 7.35–7.29 (3H, m, Ar**H**), 7.26–7.22 (2H, m, Ar**H**), 2.54 (1H, dt, *J* = 12.4, 5.5 Hz, =CC**H**$_2$), 2.44–7.25 (3H, m, C**H**$_2$CH$_2$CH$_2$C**H**$_2$), 2.22 (3H, s, C**H**$_3$), 1.92–1.58 (4H, m, CH$_2$C**H**$_2$C**H**$_2$CH$_2$); ^{13}C NMR (100.6 MHz, $CDCl_3$) δ 189.0 (C), 166.6 (C), 165.5 (C), 146.3 (C), 141.5 (C), 141.3 (C), 139.9 (C), 134.5 (C), 129.2 (2 × CH), 129.1 (CH), 128.4 (2 × CH), 127.8 (CH), 126.5 (CH), 121.1 (CH), 121.0 (CH), 114.9 (C), 75.5 (C), 27.6 (CH_2), 21.7 (CH_2), 21.3 (CH_2), 20.9 (CH_2), 11.9 (CH_3). HRMS (ESI +ve) Exact mass calculated for $C_{24}H_{20}NaO_3$ $[M+Na]^+$: 379.1305, found: 379.1308. Enantiomeric excess was determined by HPLC with a Chiralpak OD-H column (99:1 *iso*-hexane:EtOH, 0.8 mL/min, 280 nm, 25 °C); t_r (major) = 16.3 min, t_r (minor) = 18.4 min; 94% ee.

Data file: C:\CHEM32\1\DATA\IK\DEF_LC 2015-04-23 17-15-22\IK-763V.D
Sample name: IK-763V
Instrument: AGILENT 1260
Injection date: 4/23/2015 10:50:23 PM
Acq. method: ODH99B01D.0.8ML.60 MIN.M

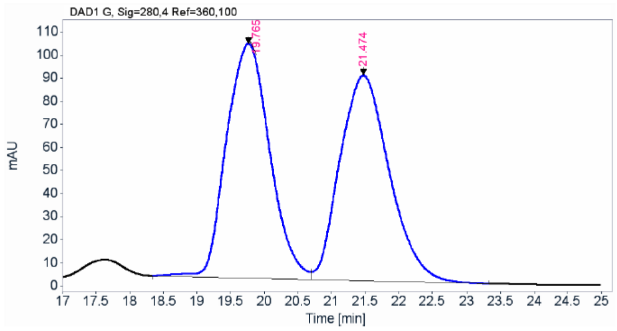

Signal: DAD1 G, Sig=280,4 Ref=360,100

RT [min]	Type	Width [min]	Area	Height	Area%
19.765	BV	0.7236	4537.399	101.4975	50.05
21.474	VB	0.8161	4527.524	88.9035	49.95

Data file: C:\CHEM32\1\DATA\IK\DEF_LC 2015-04-24 08-25-28\IK-763A.D
Sample name: IK-763a
Instrument: AGILENT 1260
Injection date: 4/24/2015 8:42:41 AM
Acq. method: ODH99B01D.0.8ML.60 MIN.M

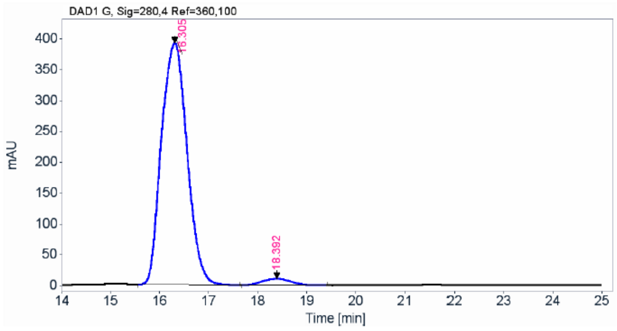

Signal: DAD1 G, Sig=280,4 Ref=360,100

RT [min]	Type	Width [min]	Area	Height	Area%
16.305	BB	0.5890	14204.534	391.8159	97.16
18.392	BB	0.6495	414.665	10.3915	2.84

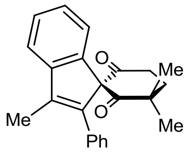

(*S*)-3,3,3'-Trimethyl-2'-phenylspiro[cyclohexane-1,1'-indene]-2,6-dione (4s). The title compound was prepared according to the General Procedure from substrate **1n** (43.2 mg, 0.20 mmol), **3f** (10.1 mg, 10 µmol), $Cu(OAc)_2$ (76.3 mg, 0.42 mmol), and alkyne **2a** (38 µL, 0.30 mmol) for a reaction time of 24 h and was purified by flash column chromatography (30% EtOAc/petrol) to give a colorless foam (51.1 mg, 77%). R_f 0.62 (50% EtOAc/petrol); $[\alpha]_D^{20}$ +48.1 (*c* 1.00, $CHCl_3$); m.p. 121−122 °C (CH_2Cl_2); IR 1692 (C=O), 1469, 1229, 1070, 751, 740, 704 cm^{-1}; ^1H NMR (400 MHz, $CDCl_3$) δ 7.41–7.29 (5H, m, Ar**H**), 7.24–7.13 (4H, m, Ar**H**), 2.88 (1H, dt, J = 18.6, 6.8 Hz, C**H**$_2$C=O), 2.60 (1H, ddd, J = 18.6, 8.0, 6.6 Hz, C**H**$_2$C=O), 2.11–2.00 (1H, m, C**H**$_2$CH$_2$=O), 2.05 (3H, s, =CC**H**$_3$), 1.72–1.61 (1H, m, C**H**$_2$CH$_2$=O), 1.28 (3H, s, C(C**H**$_3$)$_2$), 0.88 (3H, s, C(C**H**$_3$)$_2$); ^{13}C NMR (100.6 MHz, $CDCl_3$) δ 209.7 (C), 206.8 (C), 147.3 (C), 144.9 (C), 143.0 (C), 141.1 (C), 135.6 (C), 129.5 (2 × CH), 128.2 (2 × CH), 128.1 (CH), 127.9 (CH), 126.0 (CH), 121.5 (CH), 120.6 (CH), 82.8 (C), 43.9 (C), 36.0 (CH_2), 30.3 (CH_2), 24.7 (CH_3), 24.0 (CH_3), 11.2 (CH_3); HRMS (ESI +ve) Exact mass calculated for $C_{23}H_{22}NaO_2$ $[M+Na]^+$: 353.1512, found: 353.1527; Enantiomeric excess was determined by HPLC with a Chiralpak AS-H column (95:5 *iso*-hexane:*iso*-PrOH, 0.8 mL/min, 254 nm, 25 °C); t_r (minor) = 12.1 min, t_r (major) = 15.1 min; 78% ee.

Data file: C:\CHEM32\1\DATA\SURESH\DEF_LC 2014-10-11 13-17-43\S_CHI-580.D
Sample name: S_Chi-580
Instrument: AGILENT 1260
Injection date: 10/11/2014 2:41:57 PM
Acq. method: ASH95B05A.35MIN.0.8 ML.M

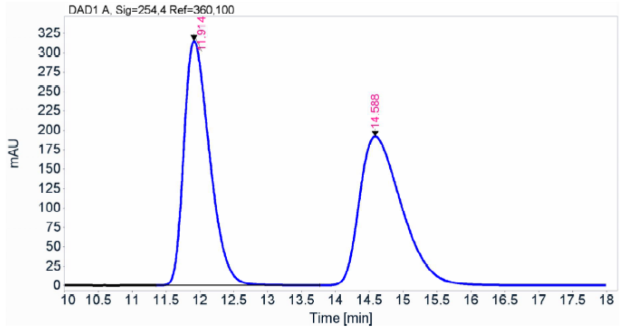

Signal: DAD1 A, Sig=254,4 Ref=360,100

RT [min]	Type	Width [min]	Area	Height	Area%
11.914	BB	0.4073	8295.150	314.9521	49.98
14.588	BB	0.6735	8303.130	191.6141	50.02

Data file: C:\CHEM32\1\DATA\DAVE\DEF_LC 2015-04-13 11-37-55\DJB7-50-1-2.D
Sample name: DJB7-50-1-2
Instrument: AGILENT 1260
Injection date: 4/13/2015 3:43:35 PM
Acq. method: ASH95B05A.35MIN.0.8 ML.M

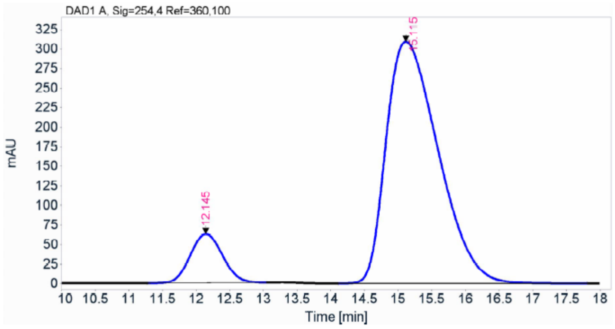

Signal: DAD1 A, Sig=254,4 Ref=360,100

RT [min]	Type	Width [min]	Area	Height	Area%
12.145	BB	0.5238	2103.866	62.9615	11.21
15.115	BB	0.8520	16660.613	309.5148	88.79

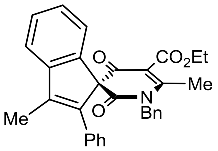

(*S*)-1'-Benzyl-3,6'-dimethyl-2',4'-dioxo-2-phenyl-2',4'-dihydro-1'*H*-spiro[indene-1,3'-pyridine]-5'-carboxylate (4t). The title compound was prepared according to the General Procedure from substrate **1o** (109 mg, 0.30 mmol), **3b** (8.4 mg, 15 μmol), $Cu(OAc)_2$ (114 mg, 0.63 mmol), and alkyne **2a** (56 μL, 0.45 mmol) for a reaction time of 24 h and was purified by flash column chromatography (20→50% EtOAc/petrol) to give a pale yellow solid (70.2 mg, 73%). R_f 0.47 (50% EtOAc/petrol); $[\alpha]_D^{20}$ +108.0 (*c* 2.00, $CHCl_3$); m.p. 137−138 °C (CH_2Cl_2/hexane); IR 1731 (C=O), 1692 (C=O) 1657 (C=O), 1594, 1406, 1321, 1243, 1140, 1041, 761 cm^{-1}; ^1H NMR (400 MHz, $CDCl_3$) δ 7.46–7.32 (8H, m, Ar**H**), 7.31–7.20 (4H, m, Ar**H**), 7.02–6.96 (2H, m, Ar**H**), 5.13–5.00 (2H, m, NC**H**$_2$Ph), 4.30 (2H, q, *J* = 7.1 Hz, OC**H**$_2$), 2.39 (3H, s, =CC**H**$_3$), 2.33 (3H, s, =CC**H**$_3$), 1.31 (3H, t, *J* = 7.1 Hz, OCH_2C**H**$_3$); ^{13}C NMR (100.6 MHz, $CDCl_3$) δ 187.4 (C), 170.7 (C), 165.7 (C), 156.5 (C), 146.7 (C), 142.7 (C), 141.1 (C), 141.0 (C), 135.9 (C), 134.8 (C), 129.2 (2 × CH), 128.9 (3 × CH), 128.4 (CH), 127.6 (CH), 127.5 (CH), 126.4 (2 × CH), 126.1 (CH), 121.0 (CH), 120.8 (CH), 116.1 (C), 76.4 (C), 61.6 (CH_2), 46.7 (CH_2), 18.8 (CH_3), 14.0 (CH_3), 12.0 (CH_3); HRMS (ESI +ve) Exact mass calculated for $C_{31}H_{27}NNaO_4$ $[M+Na]^+$: 500.1832, found: 500.1831; Enantiomeric excess was determined by HPLC with a Chiralpak AD-H column (80:20 *iso*-hexane:*iso*-PrOH, 1.5 mL/min, 254 nm, 25 °C); t_r (major) = 6.6 min, t_r (minor) = 12.7 min; 81% ee.

Data file: C:\CHEM32\1\DATA\IK\DEF_LC 2015-05-29 15-26-57\DJB7-45-A-RAC.D
Sample name: DJB7-45-A-Rac
Instrument: AGILENT 1260
Injection date: 5/29/2015 6:18:29 PM
Acq. method: ADH80B20A.30MIN.1.5 ML.M

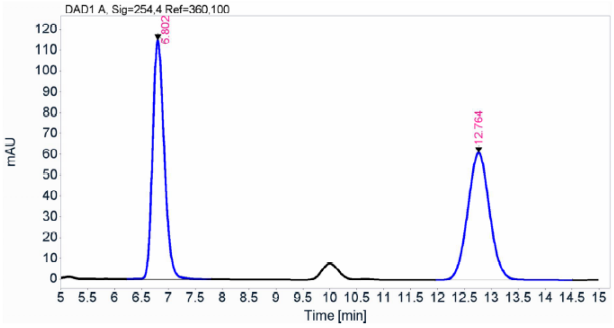

Signal: DAD1 A, Sig=254,4 Ref=360,100

RT [min]	Type	Width [min]	Area	Height	Area%
6.802	BB	0.2329	1720.336	114.7864	49.87
12.764	BB	0.4379	1729.624	61.1605	50.13

Data file: C:\CHEM32\1\DATA\DAVE\DEF_LC 2015-04-13 11-37-55\DJB7-45-A-3.D
Sample name: DJB7-45-A-3
Instrument: AGILENT 1260
Injection date: 4/13/2015 8:26:52 PM
Acq. method: ADH80B20A.30MIN.1.5 ML.M

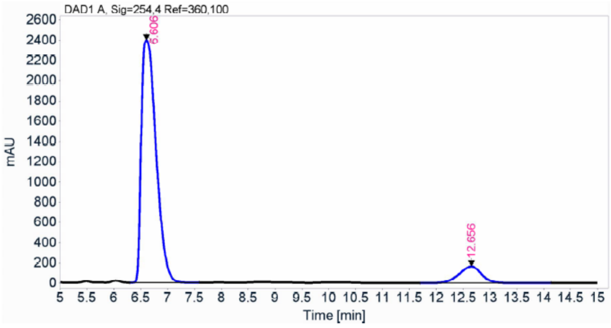

Signal: DAD1 A, Sig=254,4 Ref=360,100

RT [min]	Type	Width [min]	Area	Height	Area%
6.606	BB	0.2987	45787.059	2398.3577	90.43
12.656	BB	0.4385	4848.110	168.0769	9.57

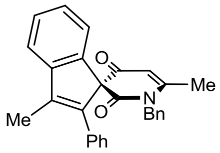

(*S*)-1'-Benzyl-3,6'-dimethyl-2-phenyl-2',4'-dihydro-1'*H*-spiro[indene-1,3'-pyridine]-2',4'-dione (4u). The title compound was prepared according to the General Procedure from substrate **1p** (87.3 mg, 0.30 mmol), **3b** (8.4 mg, 15 µmol), $Cu(OAc)_2$ (114 mg, 0.63 mmol), and alkyne **2a** (56 µL, 0.45 mmol) for a reaction time of 24 h and was purified by flash column chromatography (30→50% EtOAc/petrol) to give a yellow foam (114 mg, 94%). R_f 0.40 (50% EtOAc/petrol); $[\alpha]_D^{20}$ +243.1 (*c* 1.00, $CHCl_3$); IR 1648 (C=O), 1613 (C=O), 1406, 1271, 1169, 754, 733 cm^{-1}; ^1H NMR (400 MHz, $CDCl_3$) δ 7.47–7.33 (7H, m, Ar**H**), 7.32–7.25 (4H, m, Ar**H**), 7.20 (1H, td, *J* = 7.3, 1.6 Hz, Ar**H**), 7.05–6.97 (2H, m, Ar**H**), 5.76 (1H, s, CH_3C=C**H**), 5.11 (1H, d, *J* = 16.1 Hz, NC**H**$_2$Ph), 4.95 (1H, d, *J* = 16.1 Hz, NC**H**$_2$Ph), 2.39 (3H, s, =CC**H**$_3$), 2.28 (3H, s, =CC**H**$_3$); ^{13}C NMR (100.6 MHz, $CDCl_3$) δ 190.9 (C), 171.6 (C), 157.2 (C), 146.8 (C), 143.6 (C), 141.5 (C), 140.8 (C), 136.3 (C), 135.0 (C), 128.9 (CH), 128.7 (CH), 128.6 (CH), 128.3 (CH), 127.4 (CH), 127.3 (CH), 126.3 (CH), 125.8 (CH), 120.9 (CH), 120.4 (CH), 108.6 (CH), 76.4 (C), 46.2 (CH_2), 21.6 (CH_3), 12.0 (CH_3); HRMS (ESI +ve) Exact mass calculated for $C_{28}H_{23}NNaO_2$ $[M+Na]^+$: 428.1621, found: 428.1622; Enantiomeric excess was determined by HPLC with a Chiralpak AD-H column (80:20 *iso*-hexane:*iso*-PrOH, 1.5 mL/min, 254 nm, 25 °C); t_r (major) = 8.2 min, t_r (minor) = 20.0 min; 91% ee.

Data file: C:\CHEM32\1\DATA\SURESH\DEF_LC 2015-02-13 15-09-40\S_CHI-03-43C.D
Sample name: S_Chi-03-43c
Instrument: AGILENT 1260
Injection date: 2/13/2015 3:26:53 PM
Acq. method: ADH80B20A.30MIN.1.5 ML.M

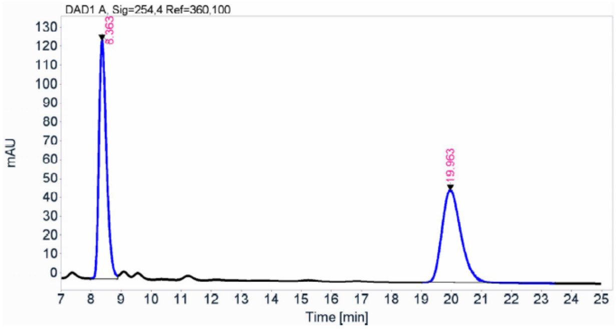

Signal: DAD1 A, Sig=254,4 Ref=360,100

RT [min]	Type	Width [min]	Area	Height	Area%
8.363	BV	0.2676	2176.085	125.9569	49.99
19.963	BB	0.6872	2177.025	48.9163	50.01

Data file: C:\CHEM32\1\DATA\DAVE\DEF_LC 2015-04-13 11-37-55\DJB7-45-B-2.D
Sample name: DJB7-45-B-2
Instrument: AGILENT 1260
Injection date: 4/13/2015 2:46:15 PM
Acq. method: ADH80B20A.30MIN.1.5 ML.M

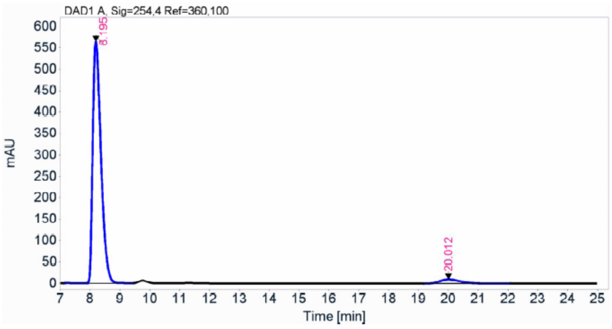

Signal: DAD1 A, Sig=254,4 Ref=360,100

RT [min]	Type	Width [min]	Area	Height	Area%
8.195	MM	0.3209	10874.789	564.8427	95.51
20.012	BB	0.7411	511.766	10.6413	4.49

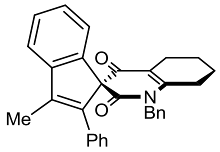

(*S*)-1'-Benzyl-3-methyl-2-phenyl-2',4',5',6',7',8'-hexahydro-1'*H*-spiro[indene-1,3'-quinoline]-2',4'-dione (4v). The title compound was prepared according to the General Procedure from substrate **1q** (99.3 mg, 0.30 mmol), **3b** (8.4 mg, 15 µmol), $Cu(OAc)_2$ (114 mg, 0.63 mmol), and alkyne **2a** (56 µL, 0.45 mmol) for a reaction time of 12 h and was purified by flash column chromatography (8% acetone/petrol) to give an off-white solid (119 mg, 89%). R_f 0.20 (15% acetone/petrol); $[\alpha]_D^{20}$ +131.9 (*c* 0.80, $CHCl_3$); m.p. 155–166 °C (EtOAc/hexane); IR 1682 (C=O), 1642, 1608, 1410, 1306, 1284, 1165, 765, 720 cm^{-1}; ^1H NMR (400 MHz, $CDCl_3$) δ 7.42–7.35 (6H, m, Ar**H**), 7.35–7.29 (1H, m, Ar**H**), 7.28–7.24 (3H, m, Ar**H**), 7.23–7.15 (2H, m, Ar**H**), 7.05–6.95 (2H, m, Ar**H**), 5.05 (2H, m, NC$\mathbf{H}_2$), 2.68–2.55 (1H, m, =CC$\mathbf{H}_2$), 2.47 (2H, dt, J = 6.2, 16.9 Hz, =CC$\mathbf{H}_2$), 2.31 (3H, s, C$\mathbf{H}_3$), 2.29–2.22 (1H, m, =CC$\mathbf{H}_2$), 1.81–1.48 (4H, m, $CH_2C\mathbf{H}_2C\mathbf{H}_2CH_2$); ^{13}C NMR (100.6 MHz, $CDCl_3$) δ 191.2 (C), 170.9 (C), 153.1 (C), 146.9 (C), 143.5 (C), 141.7 (C), 140.4 (C), 136.9 (C), 135.4 (C), 129.2 (2 × CH), 128.7 (2 × CH), 128.5 (CH), 128.2 (2 × CH), 127.28 (CH), 127.27 (CH), 126.3 (2 × CH), 125.8 (CH), 120.9 (CH), 120.5 (CH), 116.2 (C), 76.6 (C), 45.4 (CH_2), 27.4 (CH_2), 22.4 (CH_2), 22.0 (CH_2), 20.9 (CH_2), 12.0 (CH_3); HRMS (ESI +ve) Exact mass calculated for $C_{31}H_{28}NO_2$ $[M+H]^+$: 446.2115, found: 446.2123. Enantiomeric excess was determined by HPLC with a Chiralpak AD-H column (90:10 *iso*-hexane:*i*-PrOH, 1.5 mL/min, 254 nm, 25 °C); t_r (major) = 18.4 min, t_r (minor) = 34.1 min; 97% ee.

Data file: C:\CHEM32\1\DATA\IK\DEF_LC 2015-04-27 13-36-36\S_CHI-04-11A.D
Sample name: s_chi-04-11a
Instrument: AGILENT 1260
Injection date: 4/27/2015 2:56:30 PM
Acq. method: ADH90B10A.40MIN.1.5 ML.M

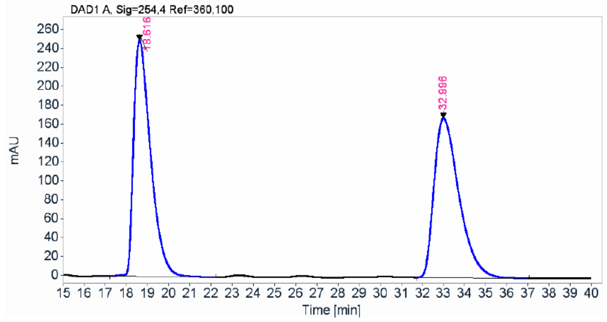

Signal: DAD1 A, Sig=254,4 Ref=360,100

RT [min]	Type	Width [min]	Area	Height	Area%
18.616	BB	0.8512	13910.523	249.2341	50.25
32.996	BB	1.2446	13773.867	168.3316	49.75

Data file: C:\CHEM32\1\DATA\IK\DEF_LC 2015-05-11 09-26-11\S_CHI-04-27.D
Sample name: s_chi-04-27
Instrument: AGILENT 1260
Injection date: 5/11/2015 11:10:46 AM
Acq. method: ADH90B10A.40MIN.1.5 ML.M

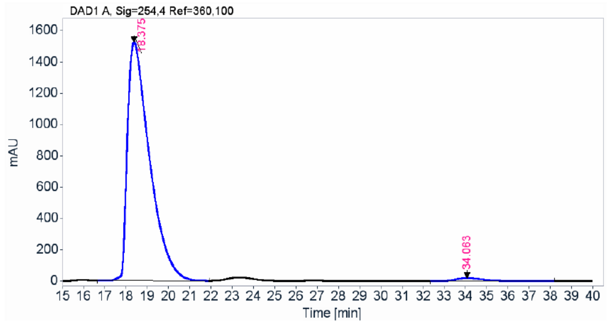

Signal: DAD1 A, Sig=254,4 Ref=360,100

RT [min]	Type	Width [min]	Area	Height	Area%
18.375	BB	1.0634	110278.383	1525.4082	98.47
34.063	BB	1.3851	1719.052	18.9011	1.53

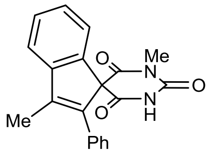

(*R*/*S*)-1,3'-Dimethyl-2'-phenylspiro[1,5-diazinane-3,1'-indene]-2,4,6-trione (4w) (Absolute stereochemistry not known with certainty). The title compound was prepared according to the General Procedure from substrate **1r** (43.6 mg, 0.20 mmol), **3b** (8.4 mg, 10 μmol), $Cu(OAc)_2$ (76.3 mg, 0.42 mmol), and alkyne **2a** (38 µL, 0.30 mmol) for a reaction time of 24 h and was purified by flash column chromatography (30% EtOAc/petrol) to give a colorless foam (50.3 mg, 76%). R_f 0.47 (50% EtOAc/petrol); $[\alpha]_D^{20}$ +75.8 (*c* 1.00, $CHCl_3$); m.p. 191−192 °C (CH_2Cl_2); IR 3222 (N−H), 1683 (C=O), 1437, 1358, 1250, 986, 757, 703 cm^{-1}; ^{1}H NMR (400 MHz, $CDCl_3$) δ 8.83 (1H, br s, N**H**), 7.46–7.32 (5H, m, Ar**H**), 7.32–7.26 (2H, m, Ar**H**), 7.26–7.20 (2H, m, Ar**H**), 3.29 (3H, s, NC**H**$_3$), 2.23 (3H, s, C**H**$_3$);^{13}C NMR (100.6 MHz, $CDCl_3$) δ 167.4 (C), 166.6 (C), 150.1 (C), 146.7 (C), 142.7 (C), 142.2 (C), 139.5 (C), 133.9 (C), 129.6 (CH), 128.8 (CH), 128.7 (CH), 128.2 (CH), 126.7 (CH), 121.2 (CH), 120.6 (CH), 70.0 (C), 28.5 (CH_3), 11.9 (CH_3); HRMS (ESI +ve) Exact mass calculated for $C_{20}H_{16}N_2NaO_3$ $[M+Na]^+$: 355.1053, found: 355.1059; Enantiomeric excess was determined by HPLC with a Chiralpak AD-H column (90:10 *iso*-hexane:*iso*-PrOH, 1.5 mL/min, 254 nm, 25 °C); t_r (major) = 11.8 min, t_r (minor) = 19.8 min; 11% ee.

Data file: C:\CHEM32\1\DATA\IK\DEF_LC 2014-08-29 10-31-57\IK-658A.D
Sample name: IK-658a
Instrument: AGILENT 1260
Injection date: 8/29/2014 1:07:04 PM
Acq. method: ADH90B10A.40MIN.1.5 ML.M

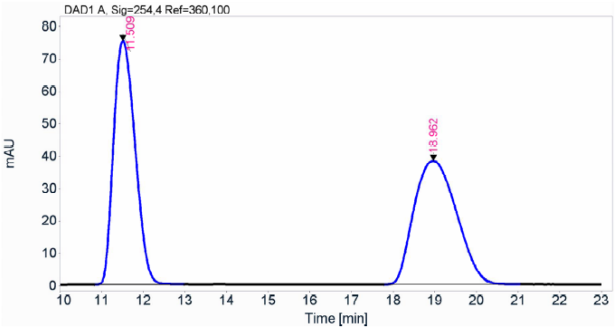

Signal: DAD1 A, Sig=254,4 Ref=360,100

RT [min]	Type	Width [min]	Area	Height	Area%
11.509	BB	0.5816	2704.695	75.2101	49.88
18.962	BB	1.1173	2718.188	38.2378	50.12

Data file: C:\CHEM32\1\DATA\DAVE\DEF_LC 2015-04-11 13-55-30\DJB7-49-1.D
Sample name: DJB7-49-1
Instrument: AGILENT 1260
Injection date: 4/11/2015 9:36:14 PM
Acq. method: ADH90B10A.40MIN.1.5 ML.M

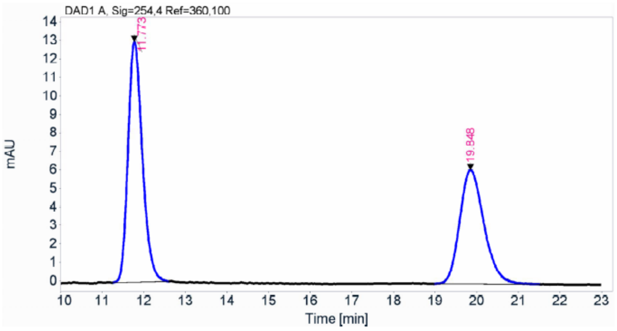

Signal: DAD1 A, Sig=254,4 Ref=360,100

RT [min]	Type	Width [min]	Area	Height	Area%
11.773	BB	0.3669	310.765	12.9990	55.52
19.848	BB	0.6234	248.925	6.1341	44.48

Stereochemical Determinations

The absolute stereochemistries of **4g**, **4h**, **4i**, **4j**, **4o**, and **4r** were determined by single crystal X-ray crystallography using a copper radiation source. All crystals were obtained by recrystallization from EtOAc/petroleum ether. The stereochemistries of the remaining products were assigned by analogy.

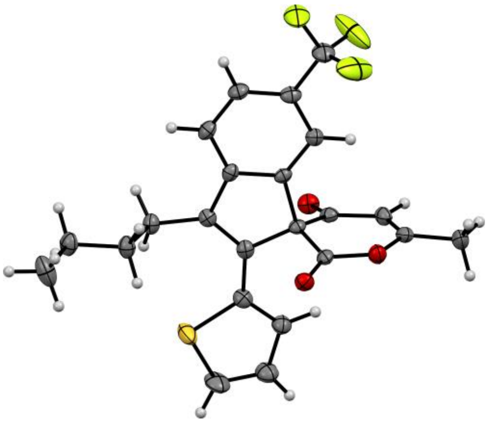

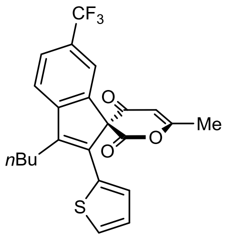

4g
CCDC 1415390
Flack parameter –0.020(15)

The CF_3 groups of **4g** are rotationally disordered over two positions (see .cif file for further details).

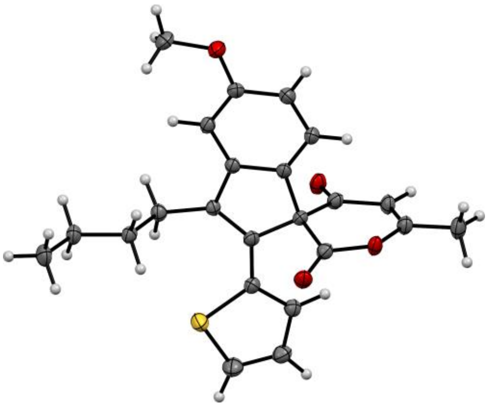

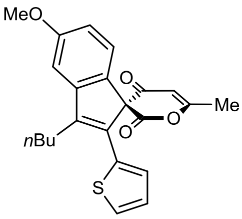

4h
CCDC 1415391
Flack parameter 0.10(3)
Hooft parameter 0.10(2)

Although the Falck and Hooft parameters of **4h** did not unambiguously indicate the presence of a single enantiomer, the P2 and P3 analysis proves that a single enantiomer is present, and that the correct hand has been determined (see .cif file for further details).

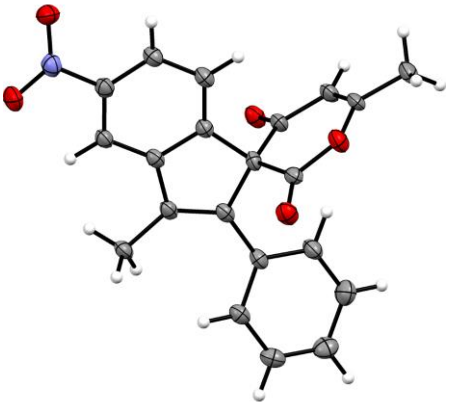

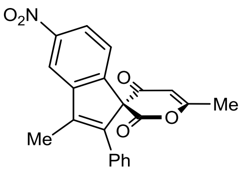

4i
CCDC 1415392
Flack parameter 0.05(16)

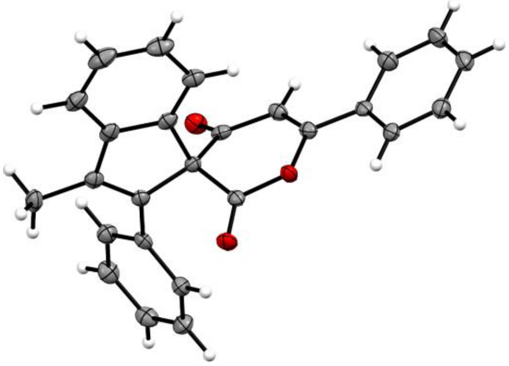

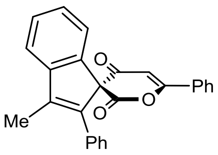

4j
CCDC 1415393
Flack parameter 0.13(19)

The absolute configuration of **4j** was confirmed by application of the Parsons quotient method as described in *Acta Cryst*. **2013,** B69, 249–259 (see .cif file for further details).

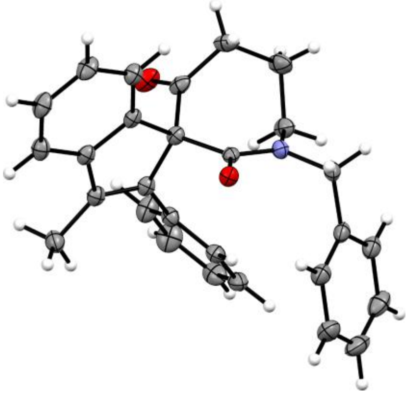

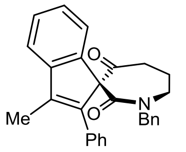

4o
CCDC 1415394
Flack parameter –0.03(9)

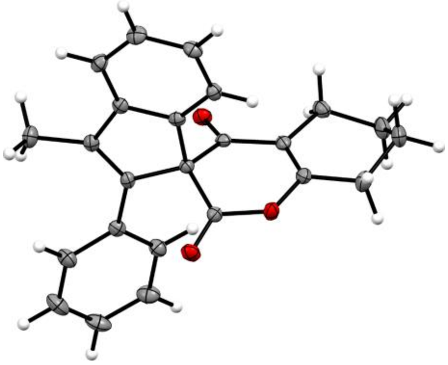

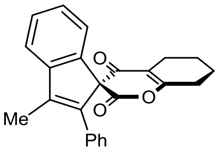

4r
CCDC 1415395
Flack parameter 0.02(8)

Deuteration Experiments

H/D Scrambling of 1c in the Absence of Alkyne

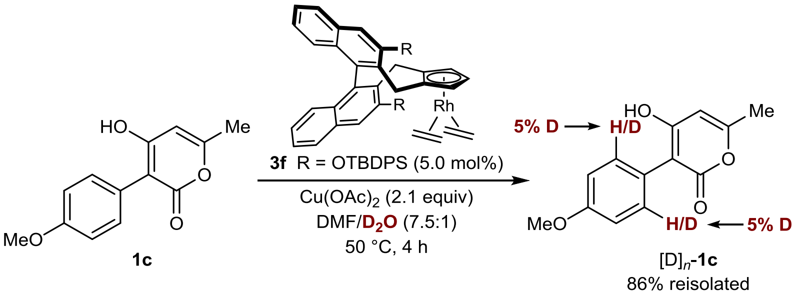

A mixture of **1c** (46.4 mg, 0.20 mmol), $Cu(OAc)_2$ (76.3 mg, 0.42 mmol), and **3f** (10.1 mg, 0.01 mmol) in DMF (1.5 mL) and D_2O (0.2 mL) was stirred at 50 ºC for 4 h. The reaction was poured into 2 M aqueous NaOH solution (20 mL) and the aqueous layer was washed with CH_2Cl_2 (2 × 30 mL). The aqueous layer was acidified to pH 3–4 with 10% aqueous HCl solution and extracted with CH_2Cl_2 (2 × 20 mL). The combined organic extracts were dried ($MgSO_4$), filtered, concentrated in *vacuo*. Purification of the residue by trituration (Et_2O) gave $[D]_n$-**1c** as an off-white solid (40.0 mg, 86%).

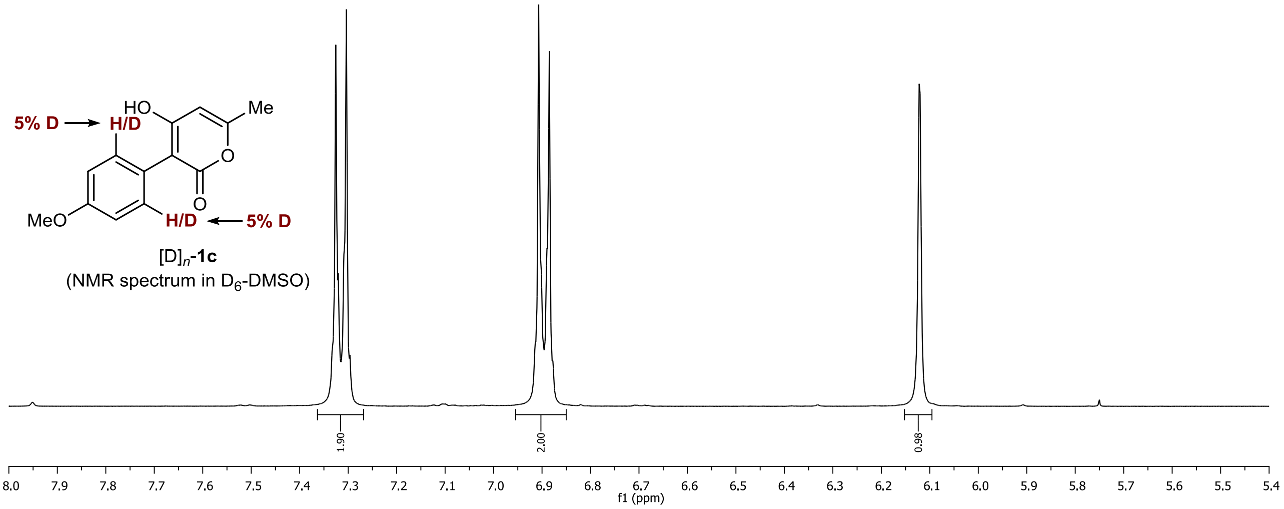

Oxidative Annulation of 1c in the Presence of D_2O

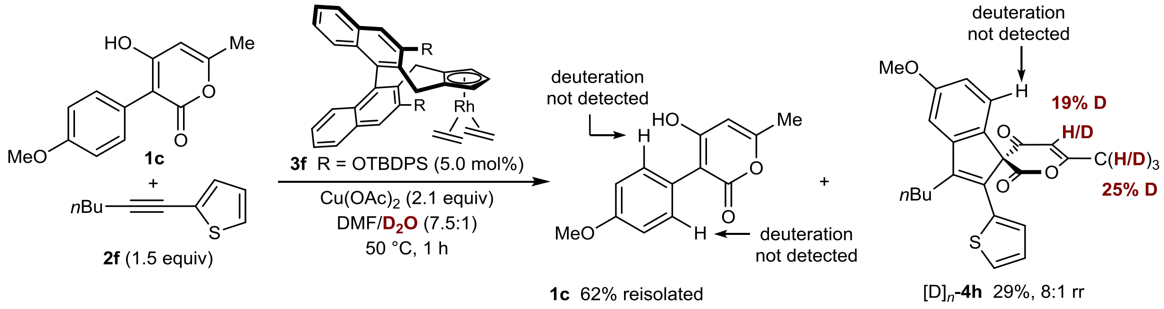

To a mixture of **1c** (46.4 mg, 0.20 mmol), $Cu(OAc)_2$ (76.3 mg, 0.42 mmol), and **3f** (10.1 mg, 0.01

mmol) in DMF (1.0 mL) and D_2O (0.2 mL) was added a solution of alkyne **2f** (49.2 mg, 0.30 mmol) in DMF (0.5 mL), and the mixture was stirred at 50 °C for 1 h. The reaction was poured into 2 M aqueous NaOH solution (20 mL) and the aqueous layer was extracted with CH_2Cl_2 (2 × 30 mL). The combined organic extracts were dried ($MgSO_4$), filtered, and concentrated *in vacuo*. Purification of the residue by flash column chromatography (10% acetone/petrol) gave spiroindene [D]$_n$-**4h** as a light brown solid as an 8:1 inseparable mixture of regioisomers (23 mg, 29%). R_f 0.45 (30% acetone/petrol).

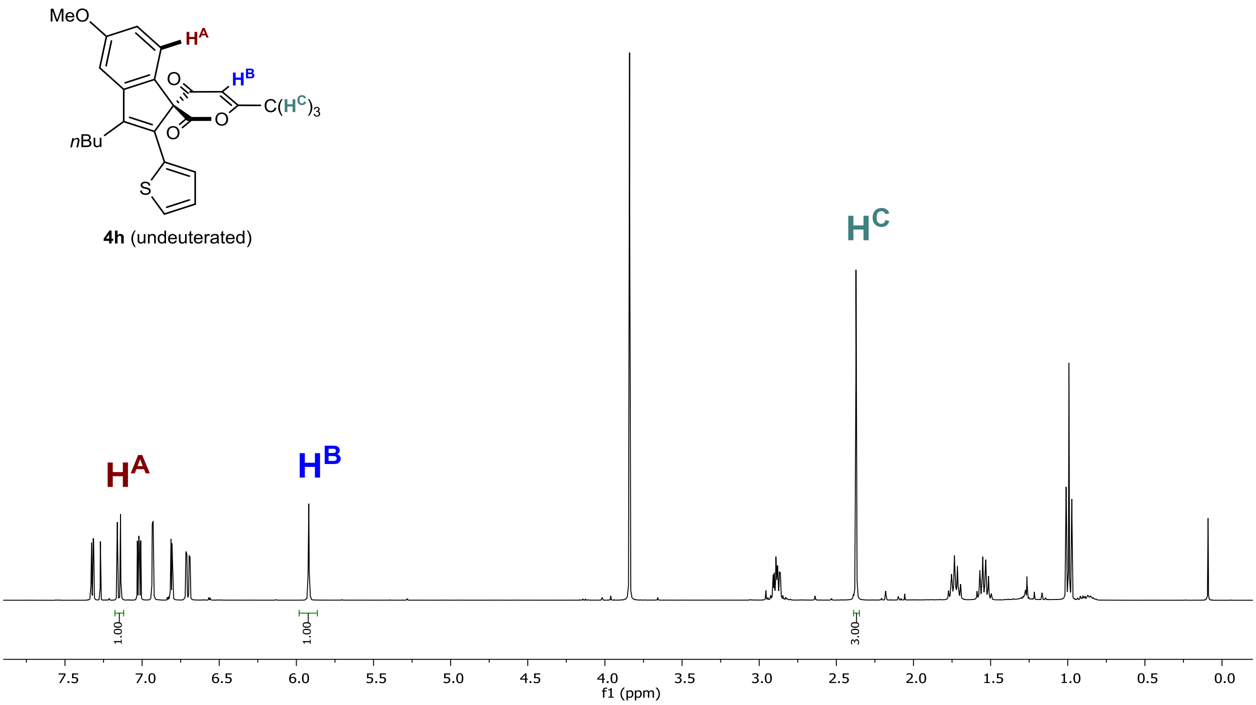

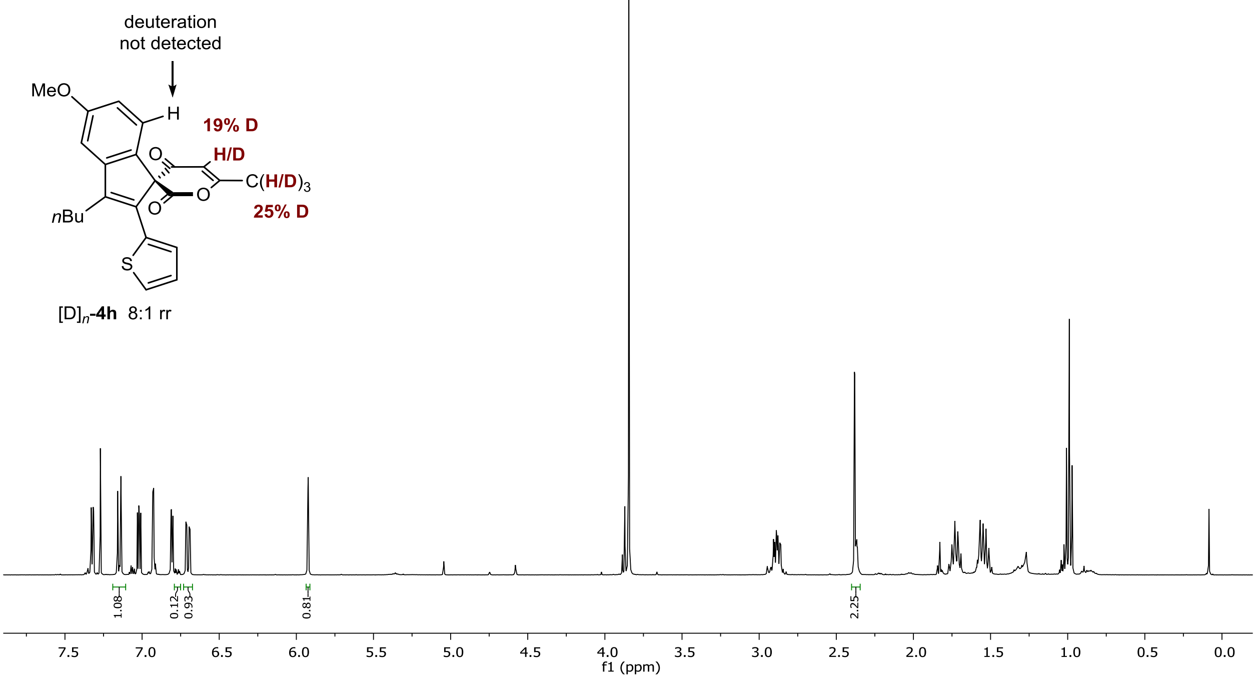

Recovered starting material: The aqueous NaOH layer was acidified to pH 3–4 with 10% aqueous HCl solution and extracted with CH_2Cl_2 (2 × 20 mL). The combined organic extracts were dried ($MgSO_4$), filtered, and concentrated *in vacuo*. Purification of the residue by trituration (Et_2O) gave recovered $[D]_n$-**1c** as an off-white solid (29.0 mg, 62%).

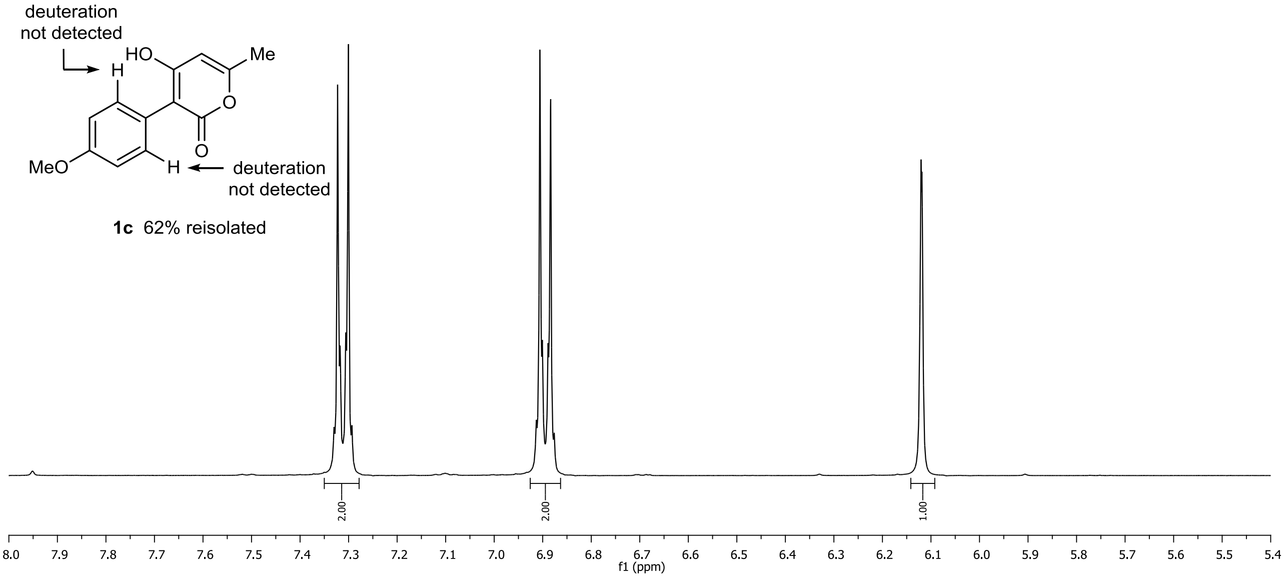

Tests for the Self-Disproportionation of Enantiomers (SDE) Phenomenon

Recently, the phenomenon termed "Self-Disproportionation of Enantiomers" (SDE) has been described, which provides the possibility for significant changes to the enantiomeric composition of samples of compounds to occur during achiral column chromatography.[17] To check whether the SDE phenomenon was observed for the series of compounds described herein, a representative product was passed through a normal silica gel column and the enantiomeric excesses of various fractions were measured, as described below. From these results, no noticeable SDE was apparent.

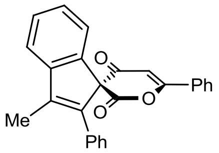

(*S*)-3-Methyl-2,6'-diphenyl-2',4'-dihydrospiro[indene-1,3'-pyran]-2',4'-dione (4j). Previously purified spiroindene **4j** (94% ee) was passed through a silica gel column (10% acetone/petrol) and collected over 9 fractions. The enantiomeric excesses for fractions 1, 5, and 8 were determined by HPLC with a Chiralpak IC column (98:2 *iso*-hexane:*iso*-PrOH, 0.8 mL/min, 210 nm, 25 °C). Fraction 1: t_r (minor) = 39.4 min, t_r (major) = 47.0 min; 93% ee. Fraction 5: t_r (minor) = 39.0 min, t_r (major) = 46.8 min; 93% ee. Fraction 8: t_r (minor) = 39.0 min, t_r (major) = 47.0 min; 92% ee.

Racemate:

Data file: C:\CHEM32\1\DATA\IK\DEF_LC 2015-04-15 15-40-46\IK-755C.D
Sample name: IK-755c
Instrument: AGILENT 1260
Injection date: 4/15/2015 4:08:17 PM
Acq. method: IC98B02A.60MIN.0.8ML.M

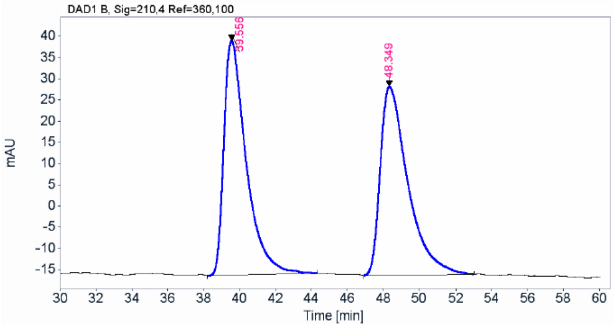

Signal: DAD1 B, Sig=210,4 Ref=360,100

RT [min]	Type	Width [min]	Area	Height	Area%
39.556	BB	1.2757	4775.345	55.1749	50.24
48.349	BB	1.5665	4729.481	44.4096	49.76

Original sample (94% ee):

Data file: C:\CHEM32\1\DATA\IK\DEF_LC 2015-04-16 08-31-54\IK-756B.D
Sample name: IK-756b
Instrument: AGILENT 1260
Injection date: 4/16/2015 3:55:30 PM
Acq. method: IC98B02A.60MIN.0.8ML.M

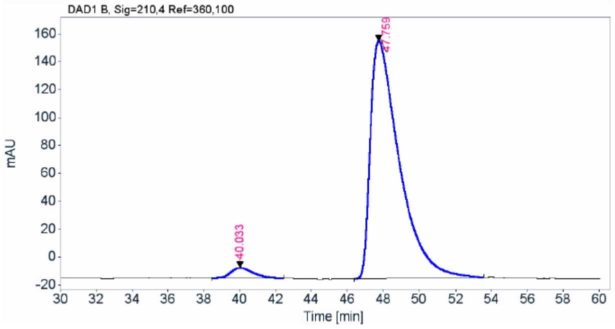

Signal: DAD1 B, Sig=210,4 Ref=360,100

RT [min]	Type	Width [min]	Area	Height	Area%
40.033	BB	1.1501	627.056	7.3444	3.18
47.759	BB	1.6496	19066.197	170.1091	96.82

17. (a) V. A. Soloshonok, *Angew. Chem., Int. Ed.* **2006**, *45*, 766-769. (b) V. A. Soloshonok, C. Roussel, O. Kitagawa, A. E. Sorochinsky, *Chem. Soc. Rev.* **2012**, *41*, 4180-4188. (c) Y. Suzuki, J. Han, O. Kitagawa, J. L. Acena, K. D. Klika, V. A. Soloshonok, *RSC Adv.* **2015**, *5*, 2988-2993.

Fraction 1: (93% ee)

Data file:	C:\CHEM32\1\DATA\MC\DEF_LC 2015-05-06 13-26-18\IK-756BFR1.D
Sample name:	IK-756bFR1
Instrument:	AGILENT 1260
Injection date:	5/6/2015 4:51:00 PM
Acq. method:	IC98B02A.60MIN.0.8ML.M

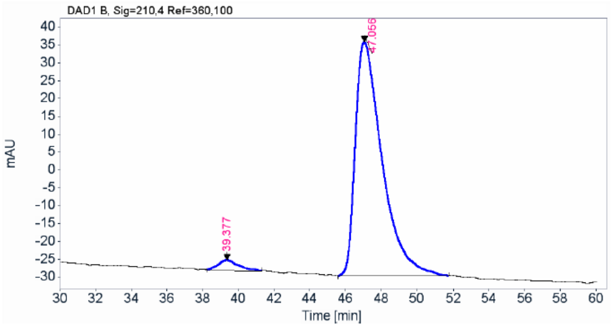

Signal: DAD1 B, Sig=210,4 Ref=360,100

RT [min]	Type	Width [min]	Area	Height	Area%
39.377	MM	1.4017	250.945	2.9838	3.55
47.056	BB	1.4942	6815.514	65.4885	96.45

Fraction 5 (93% ee):

Data file:	C:\CHEM32\1\DATA\MC\DEF_LC 2015-05-06 13-26-18\IK-756BFR2.D
Sample name:	IK-756bFR2
Instrument:	AGILENT 1260
Injection date:	5/6/2015 5:51:58 PM
Acq. method:	IC98B02A.60MIN.0.8ML.M

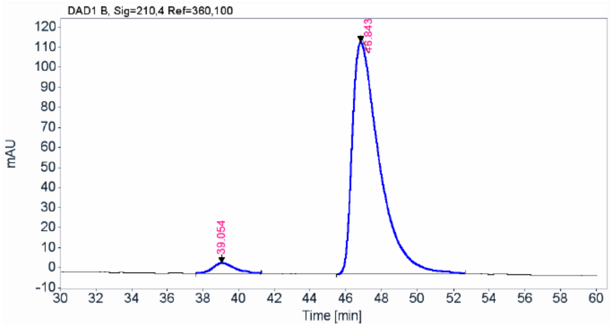

Signal: DAD1 B, Sig=210,4 Ref=360,100

RT [min]	Type	Width [min]	Area	Height	Area%
39.054	MM	1.4588	453.101	5.1765	3.59
46.843	BB	1.5535	12159.833	115.7593	96.41

Fraction 8: (92% ee)

Data file:	C:\CHEM32\1\DATA\MC\DEF_LC 2015-05-06 13-26-18\IK-756BFR3.D
Sample name:	IK-756bFR3
Instrument:	AGILENT 1260
Injection date:	5/6/2015 6:52:55 PM
Acq. method:	IC98B02A.60MIN.0.8ML.M

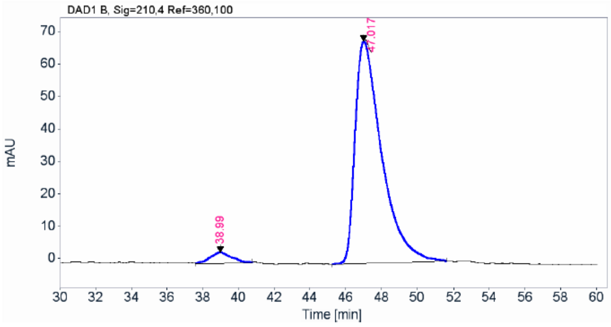

Signal: DAD1 B, Sig=210,4 Ref=360,100

RT [min]	Type	Width [min]	Area	Height	Area%
38.990	MM	1.4546	292.864	3.3556	3.99
47.017	BB	1.5257	7052.354	68.3540	96.01

NMR Spectra

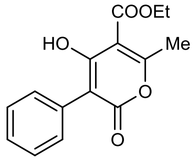

S1

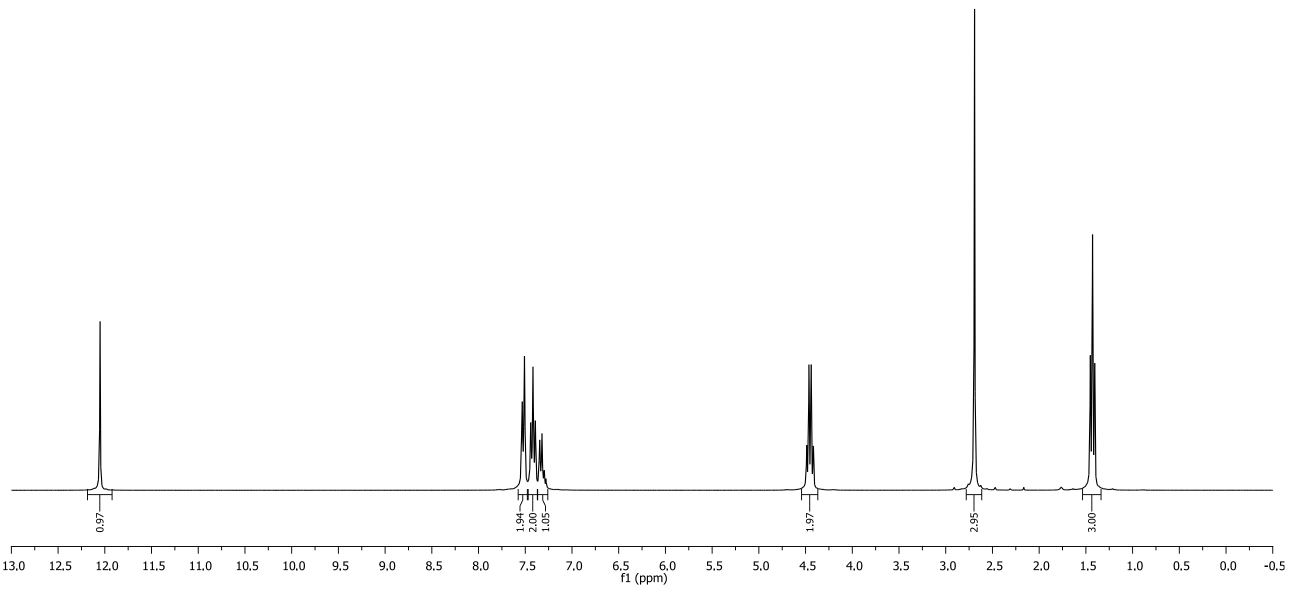

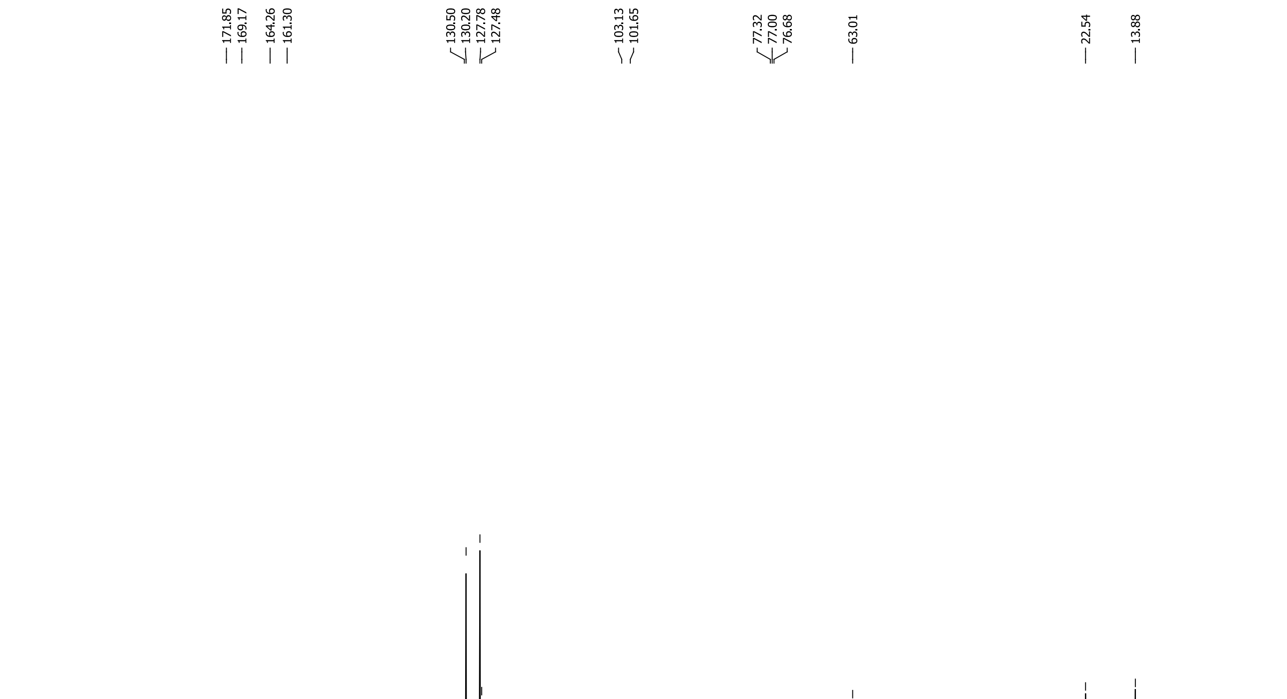

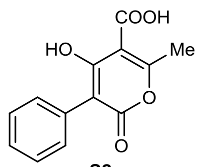
COOH
HO
Me
O
O

S2

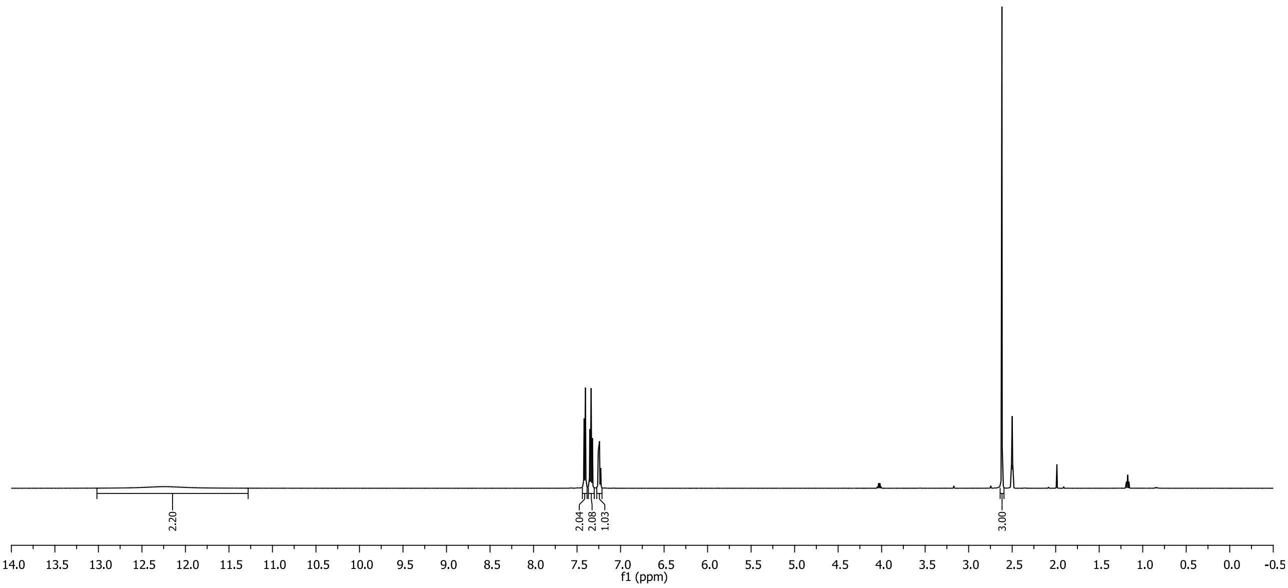
2.20
2.04
2.08
1.03
3.00
f1 (ppm)

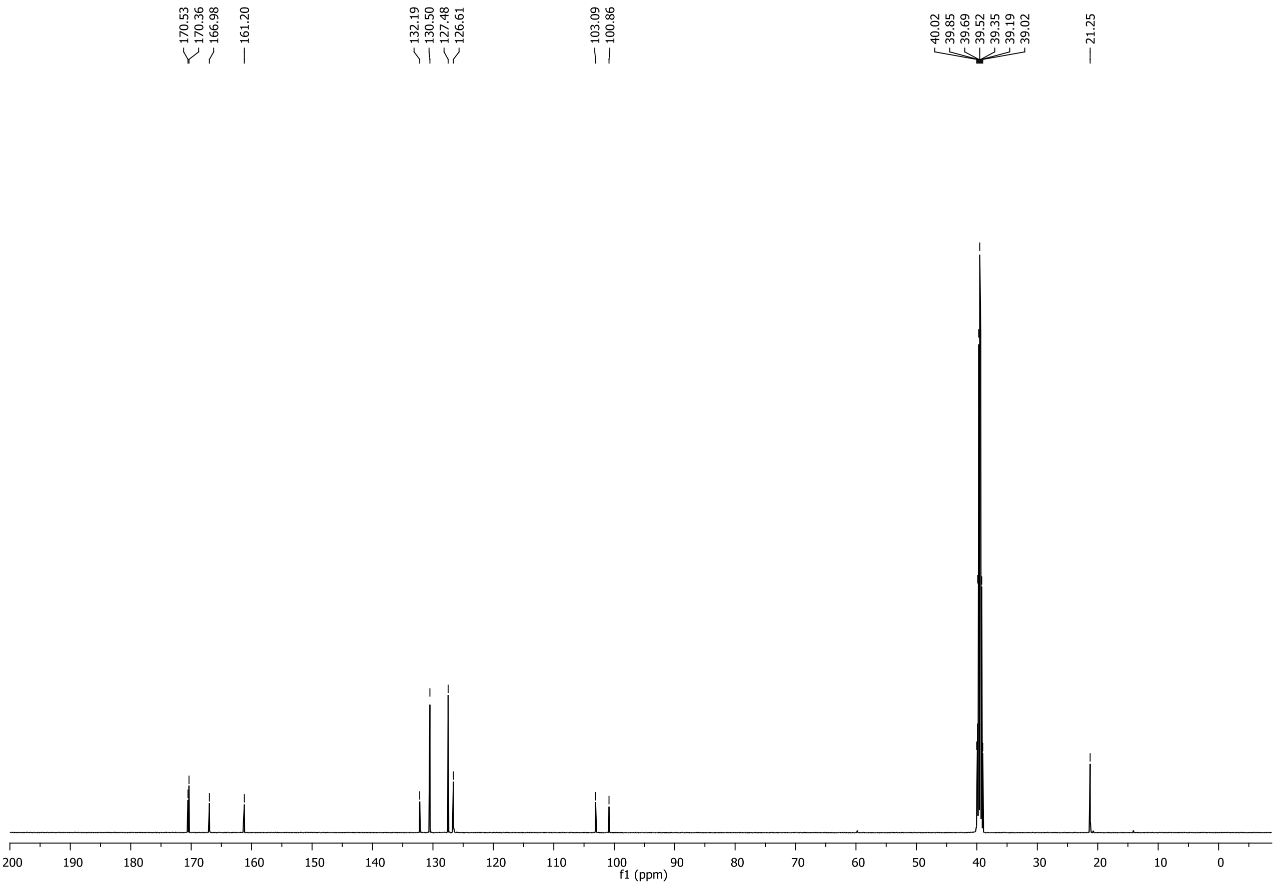
170.53
170.36
166.98
161.20
132.19
130.50
127.48
126.61
103.09
100.86
40.02
39.85
39.69
39.52
39.35
39.19
39.02
21.25
f1 (ppm)

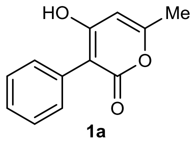
HO
Me
O
O

1a

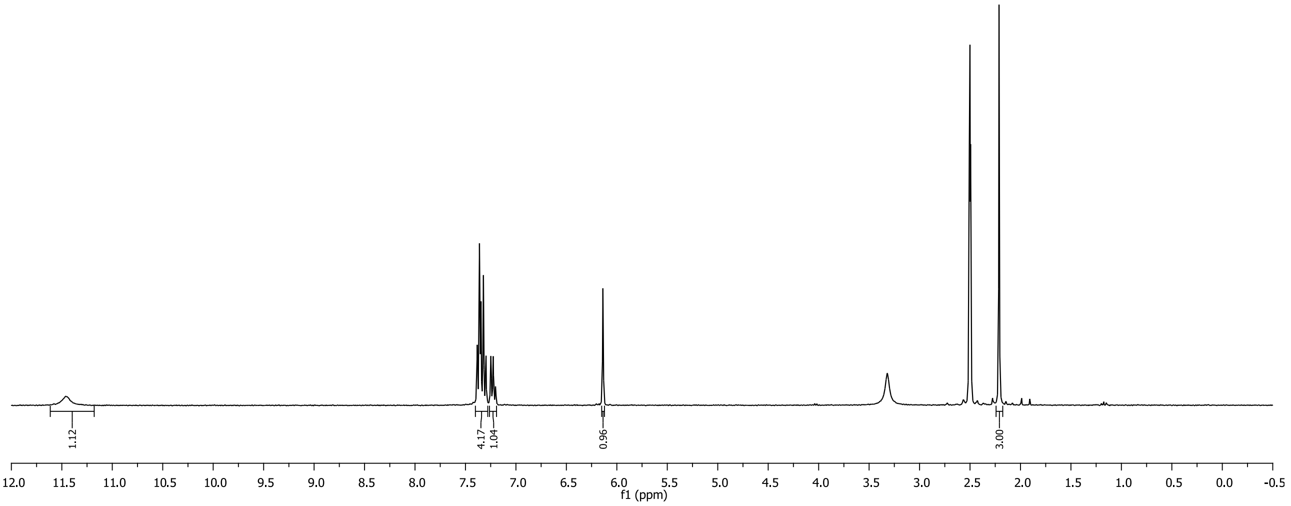
1.12
4.17
1.04
0.96
3.00
f1 (ppm)

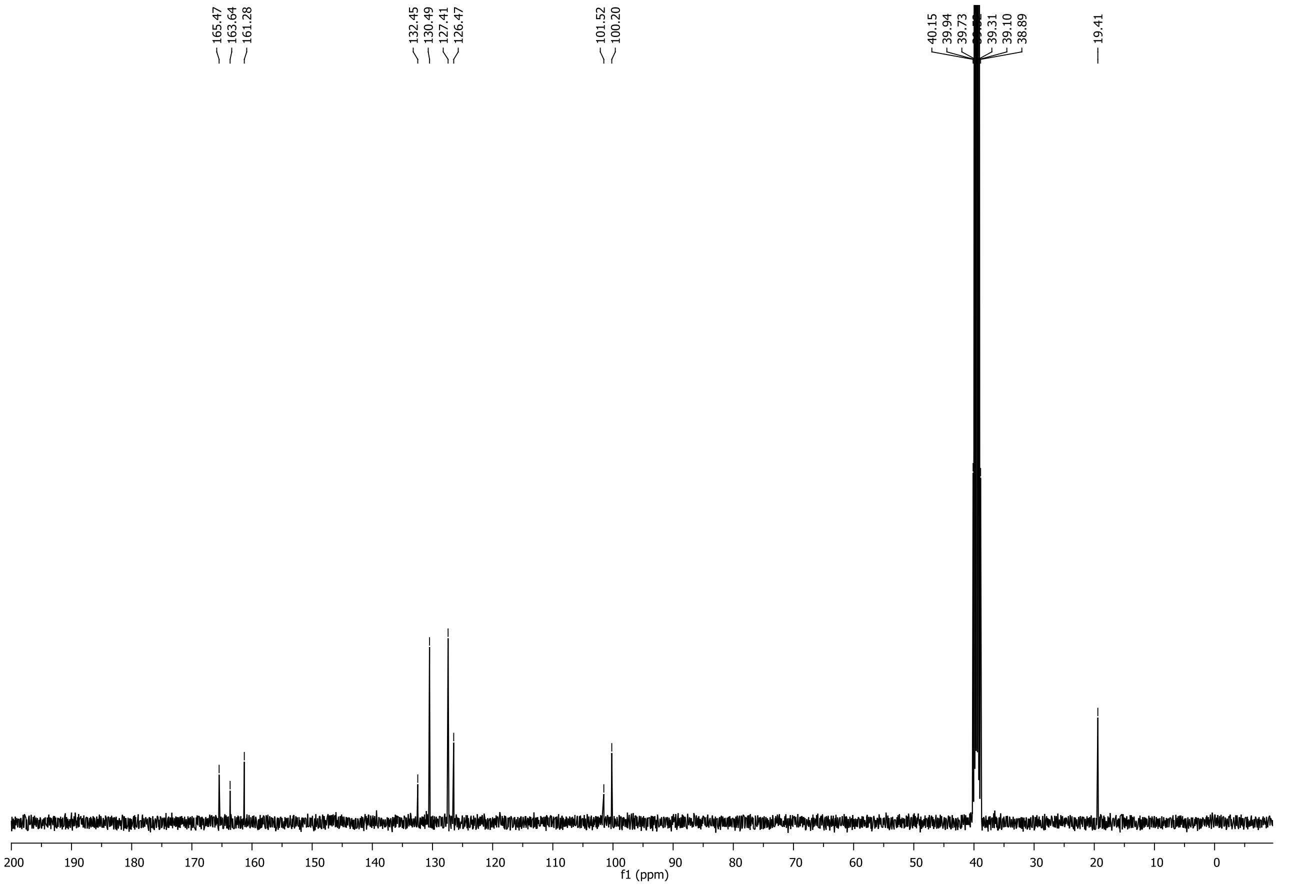
165.47
163.64
161.28
132.45
130.49
127.41
126.47
101.52
100.20
40.15
39.94
39.73
39.31
39.10
38.89
19.41
f1 (ppm)

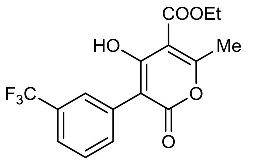
COOEt
HO
Me
F_3C
O
O

S5

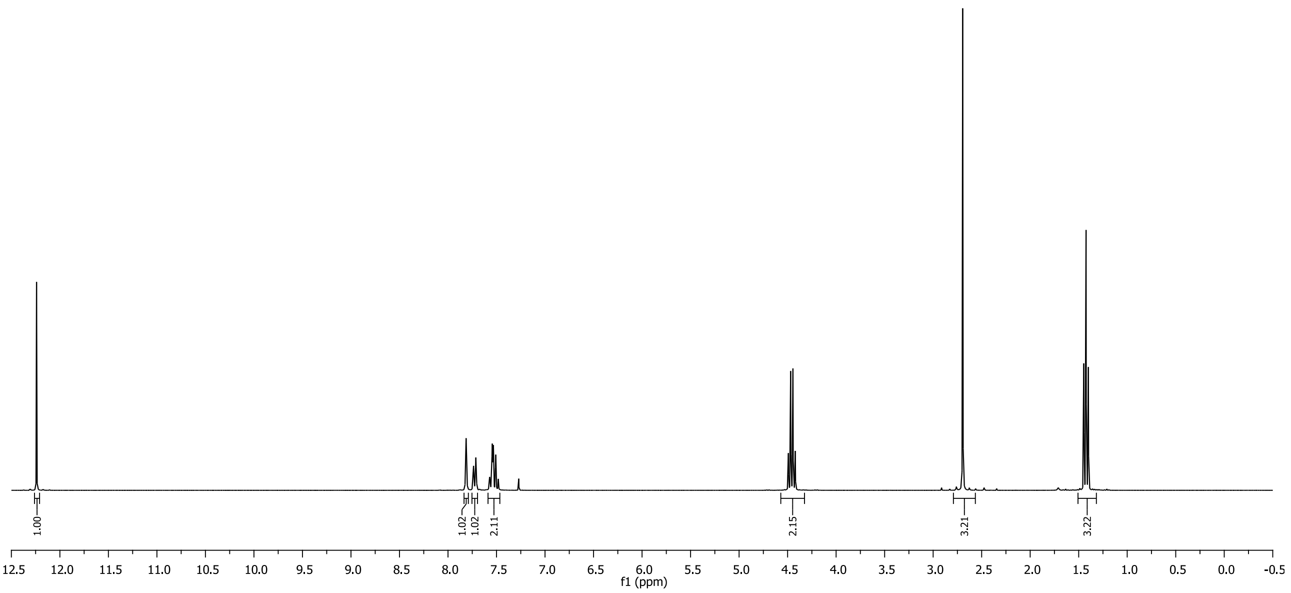
1.00
1.02
1.02
2.11
2.15
3.21
3.22
f1 (ppm)

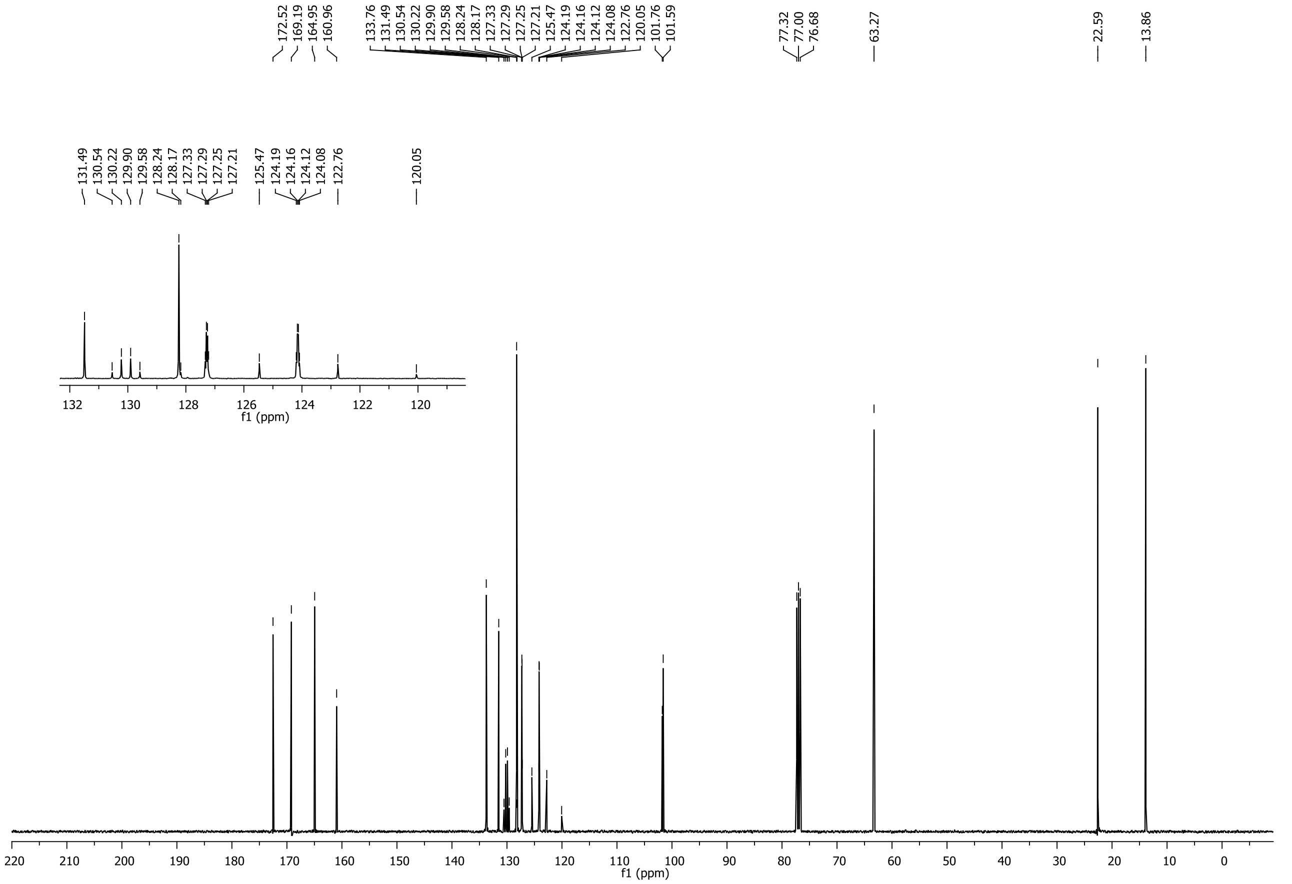
172.52
169.19
164.95
160.96
133.76
131.49
130.54
130.22
129.90
129.58
128.24
128.17
127.33
127.29
127.25
127.21
125.47
124.19
124.16
124.12
124.08
122.76
120.05
101.76
101.59
77.32
77.00
76.68
63.27
22.59
13.86
f1 (ppm)

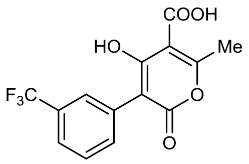
COOH
HO
Me
O
O
F3C

S6

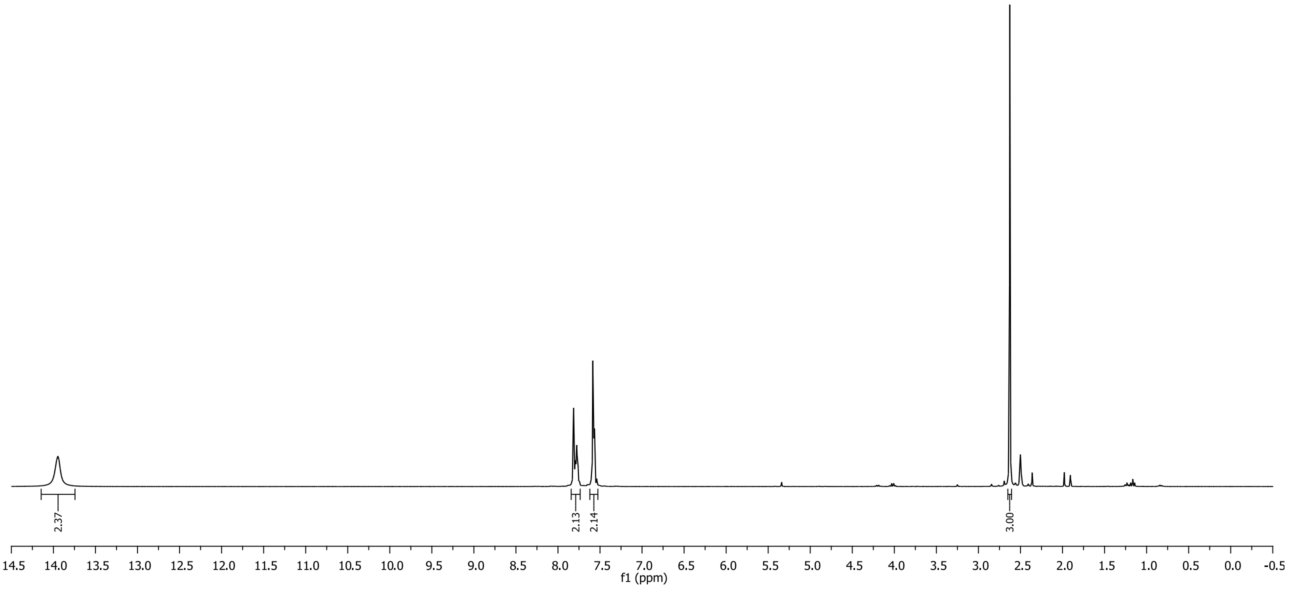
2.37
2.13
2.14
3.00
14.5 14.0 13.5 13.0 12.5 12.0 11.5 11.0 10.5 10.0 9.5 9.0 8.5 8.0 7.5 7.0 6.5 6.0 5.5 5.0 4.5 4.0 3.5 3.0 2.5 2.0 1.5 1.0 0.5 0.0 -0.5
f1 (ppm)

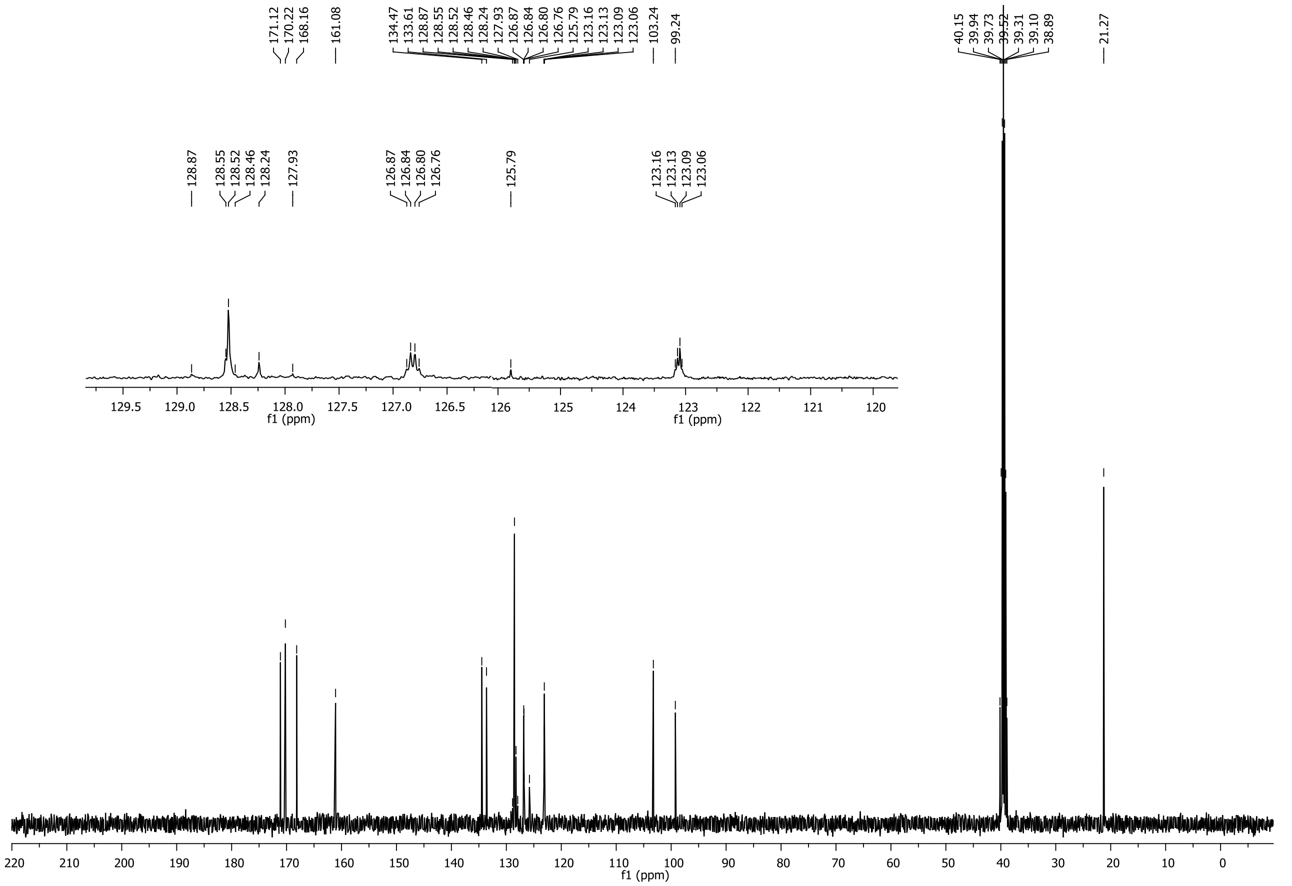
171.12
170.22
168.16
161.08
134.47
133.61
128.87
128.55
128.52
128.46
128.24
127.93
126.87
126.84
126.80
126.76
125.79
123.16
123.13
123.09
123.06
103.24
99.24
40.15
39.94
39.73
39.52
39.31
39.10
38.89
21.27
128.87
128.55
128.52
128.46
128.24
127.93
126.87
126.84
126.80
126.76
125.79
123.16
123.13
123.09
123.06
129.5 129.0 128.5 128.0 127.5 127.0 126.5 126 125 124 123 122 121 120
f1 (ppm)
f1 (ppm)
220 210 200 190 180 170 160 150 140 130 120 110 100 90 80 70 60 50 40 30 20 10 0
f1 (ppm)

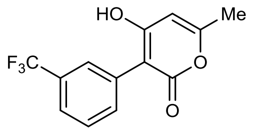
HO
Me
F_3C
O
O

1b

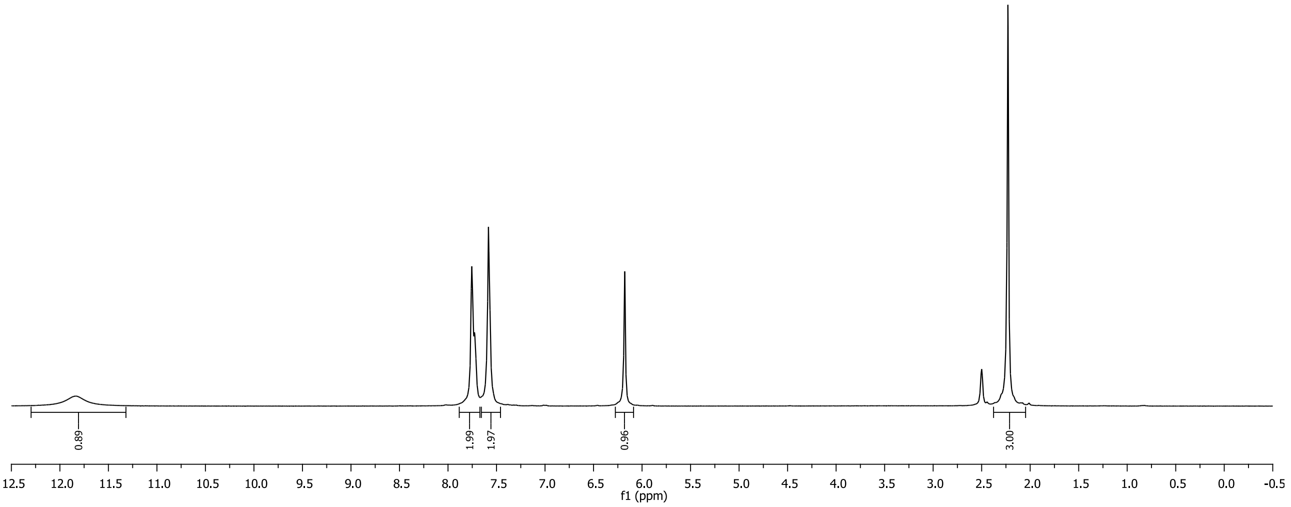
0.89
1.99
1.97
0.96
3.00
12.5 12.0 11.5 11.0 10.5 10.0 9.5 9.0 8.5 8.0 7.5 7.0 6.5 6.0 5.5 5.0 4.5 4.0 3.5 3.0 2.5 2.0 1.5 1.0 0.5 0.0 -0.5
f1 (ppm)

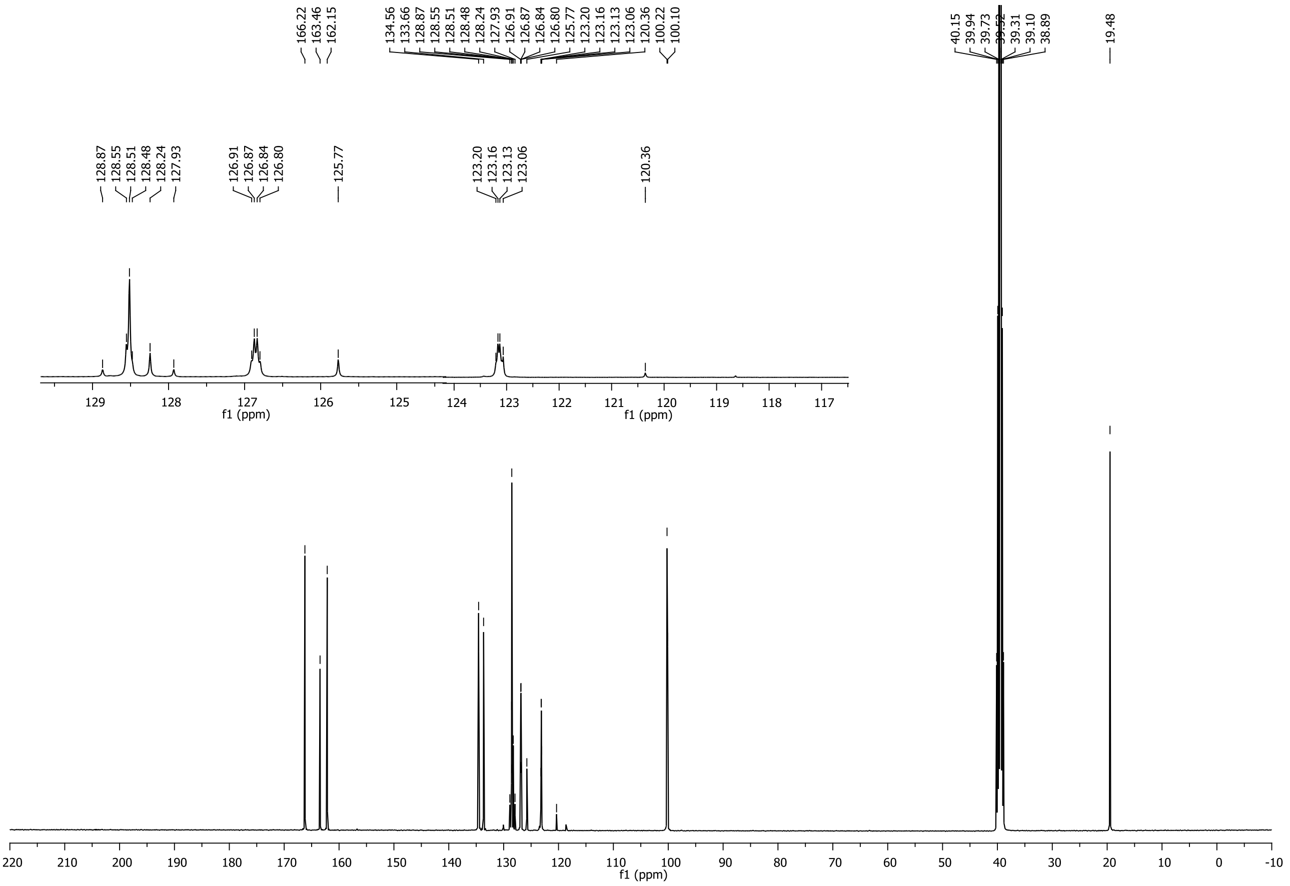
166.22
163.46
162.15
134.56
133.66
128.87
128.55
128.51
128.48
128.24
127.93
126.91
126.87
126.84
126.80
125.77
123.20
123.16
123.13
123.06
120.36
100.22
100.10
40.15
39.94
39.73
39.52
39.31
39.10
38.89
19.48
128.87
128.55
128.51
128.48
128.24
127.93
126.91
126.87
126.84
126.80
125.77
123.20
123.16
123.13
123.06
120.36
129 128 127 126 125
f1 (ppm)
124 123 122 121 120 119 118 117
f1 (ppm)
220 210 200 190 180 170 160 150 140 130 120 110 100 90 80 70 60 50 40 30 20 10 0 -10
f1 (ppm)

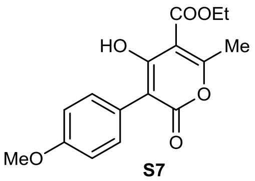
COOEt
HO
Me
O
O
MeO

S7

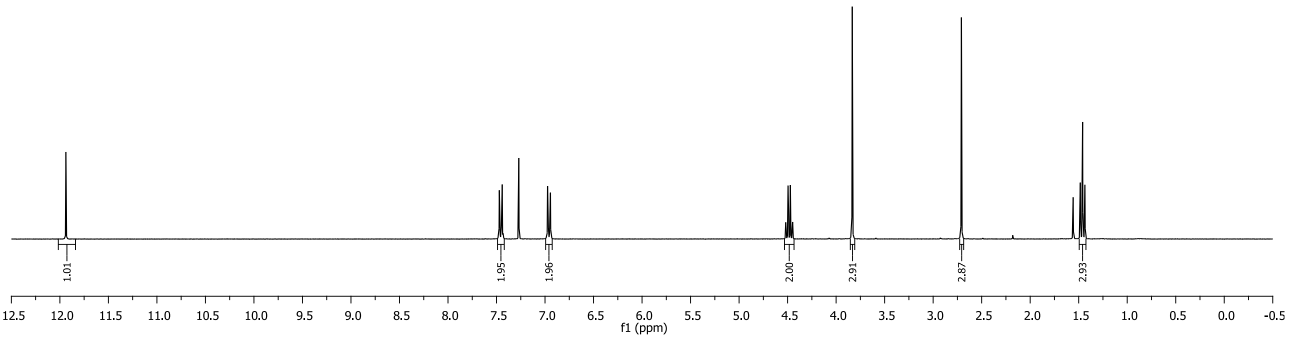
1.01
1.95
1.96
2.00
2.91
2.87
2.93
12.5 12.0 11.5 11.0 10.5 10.0 9.5 9.0 8.5 8.0 7.5 7.0 6.5 6.0 5.5 5.0 4.5 4.0 3.5 3.0 2.5 2.0 1.5 1.0 0.5 0.0 -0.5
f1 (ppm)

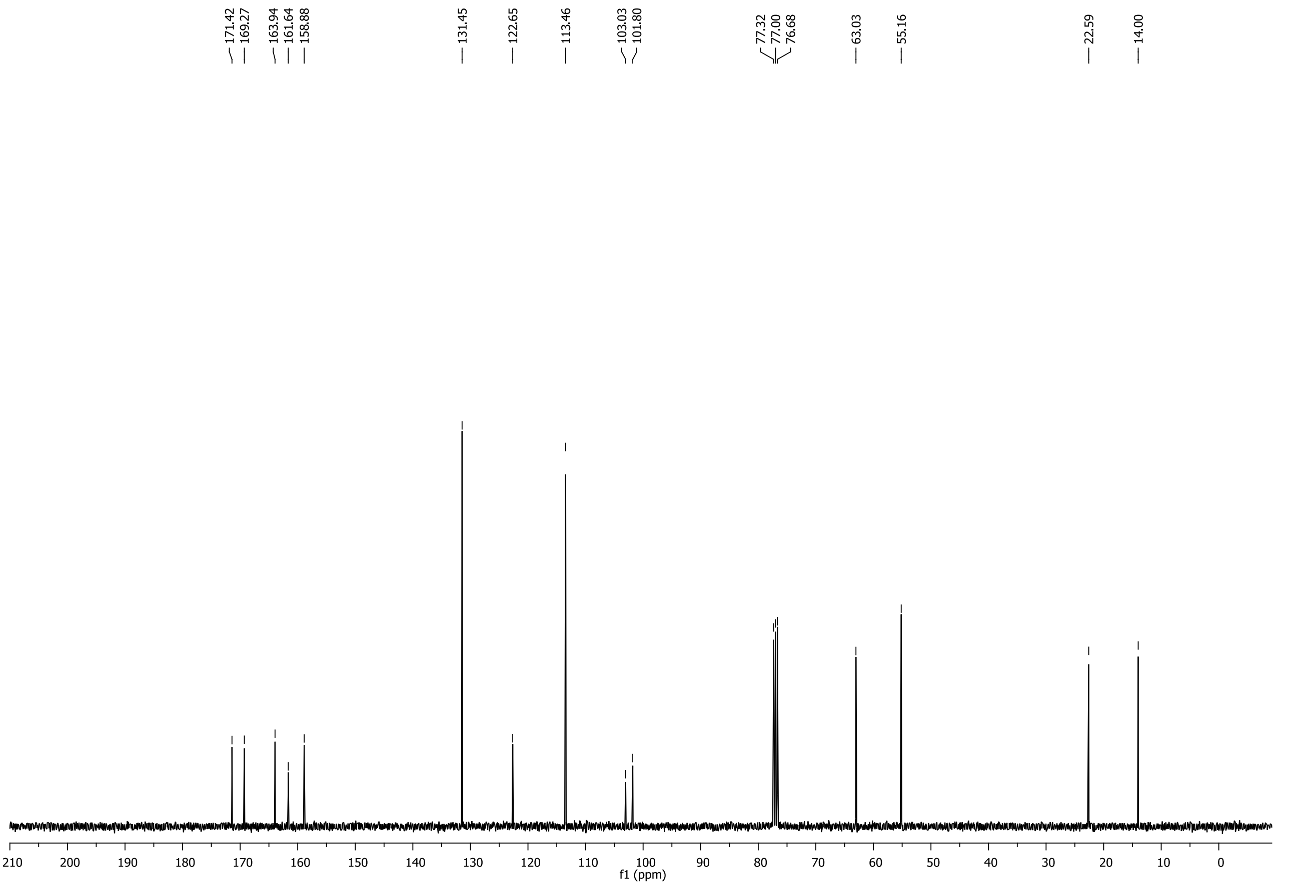
171.42
169.27
163.94
161.64
158.88
131.45
122.65
113.46
103.03
101.80
77.32
77.00
76.68
63.03
55.16
22.59
14.00
210 200 190 180 170 160 150 140 130 120 110 100 90 80 70 60 50 40 30 20 10 0
f1 (ppm)

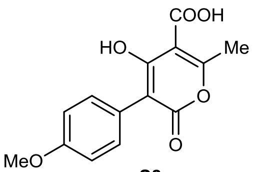
COOH
HO
Me
O
O
MeO

S8

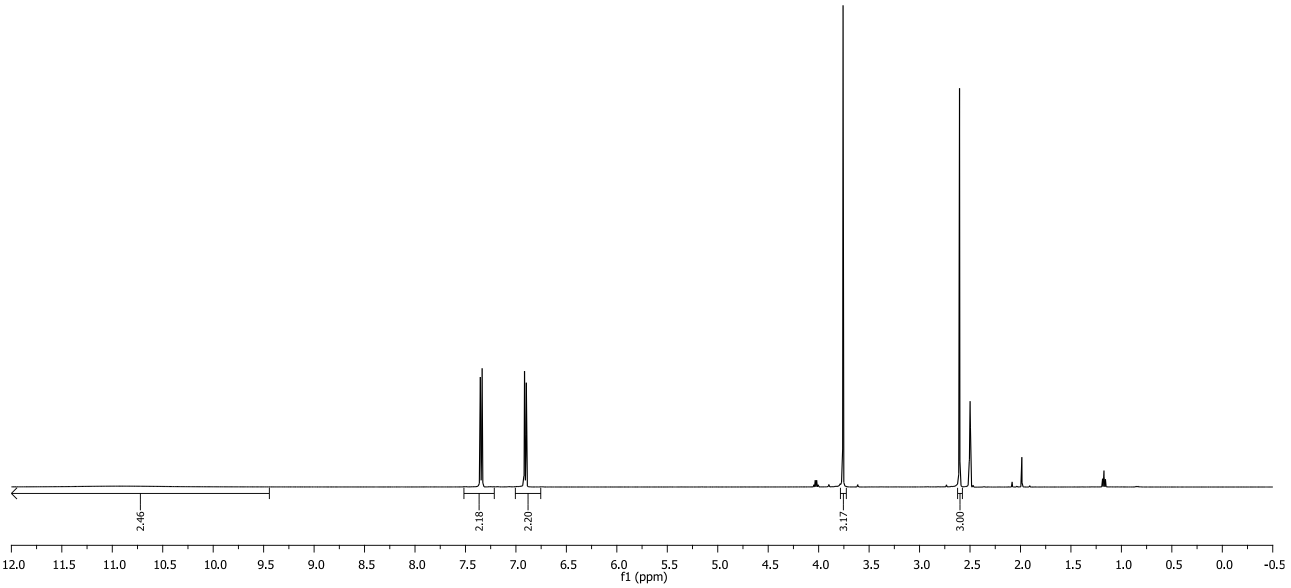
2.46
2.18
2.20
3.17
3.00
f1 (ppm)

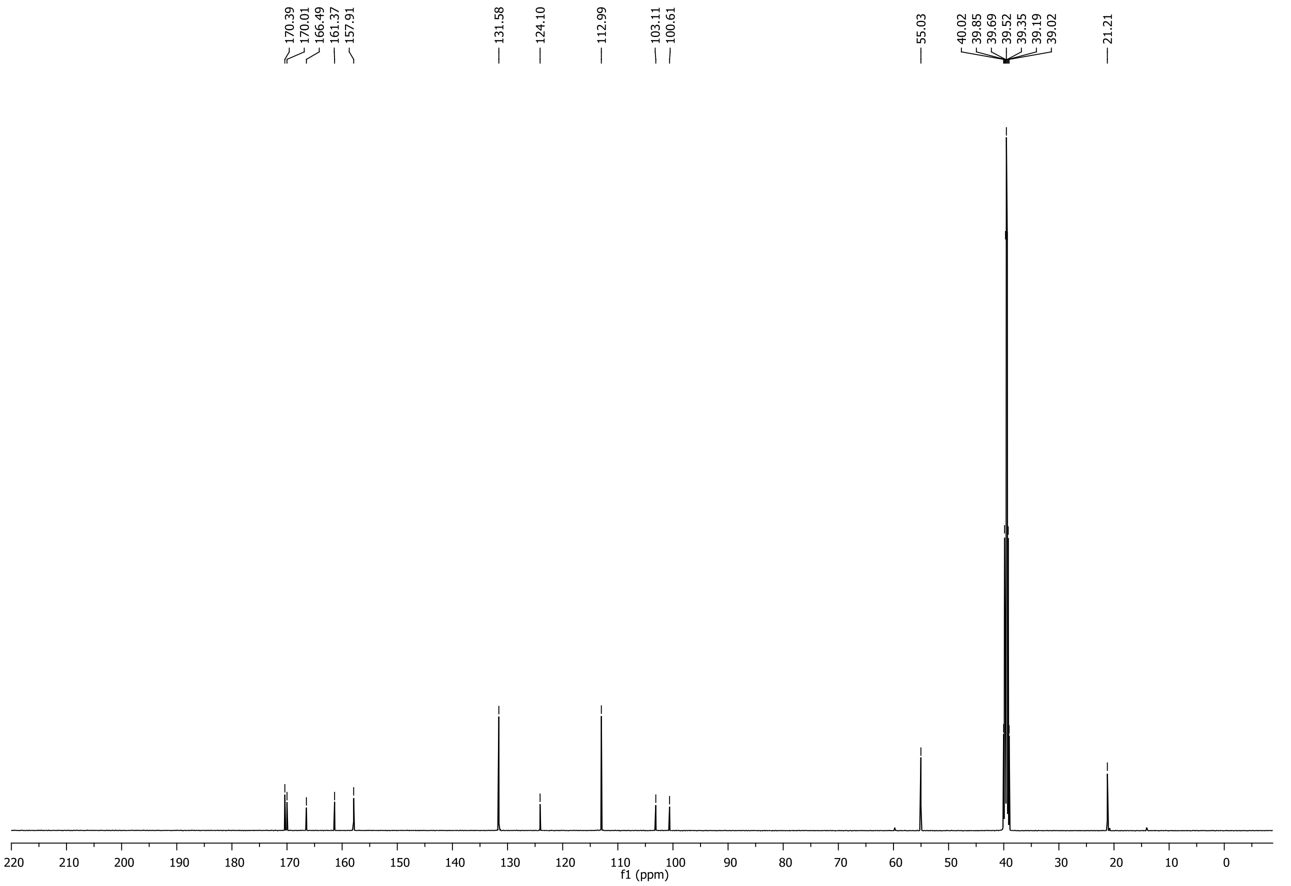
170.39
170.01
166.49
161.37
157.91
131.58
124.10
112.99
103.11
100.61
55.03
40.02
39.85
39.69
39.52
39.35
39.19
39.02
21.21
f1 (ppm)

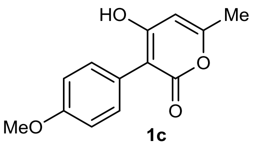
HO
Me
O
O
MeO

1c

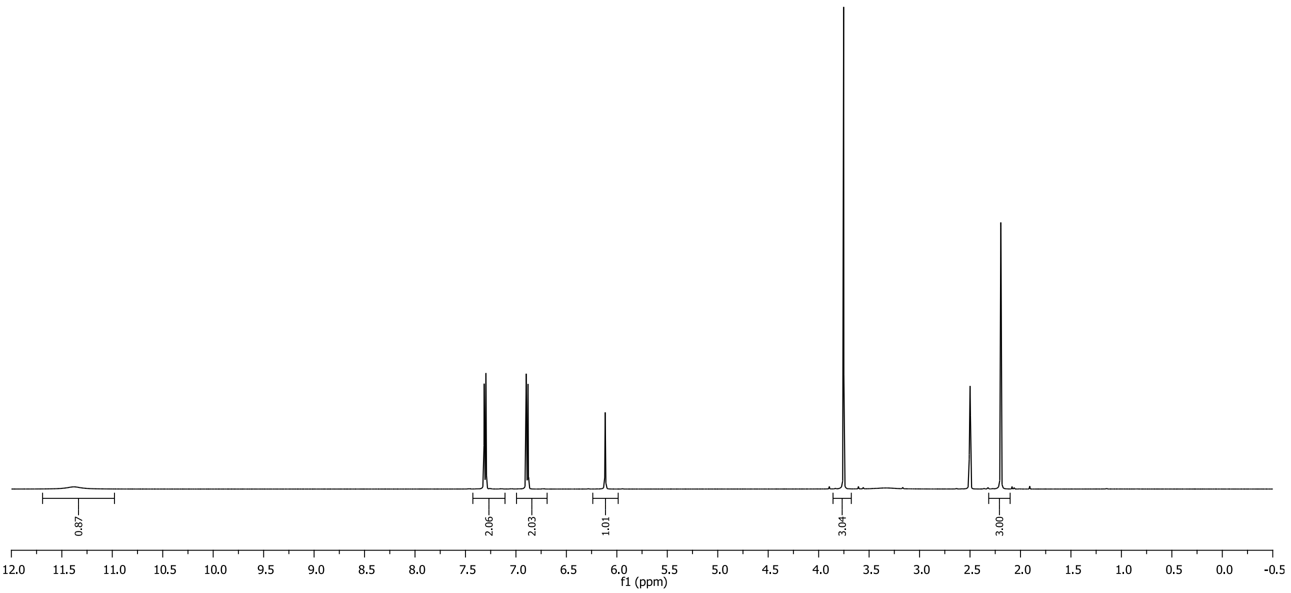
0.87
2.06
2.03
1.01
3.04
3.00
12.0 11.5 11.0 10.5 10.0 9.5 9.0 8.5 8.0 7.5 7.0 6.5 6.0 5.5 5.0 4.5 4.0 3.5 3.0 2.5 2.0 1.5 1.0 0.5 0.0 -0.5
f1 (ppm)

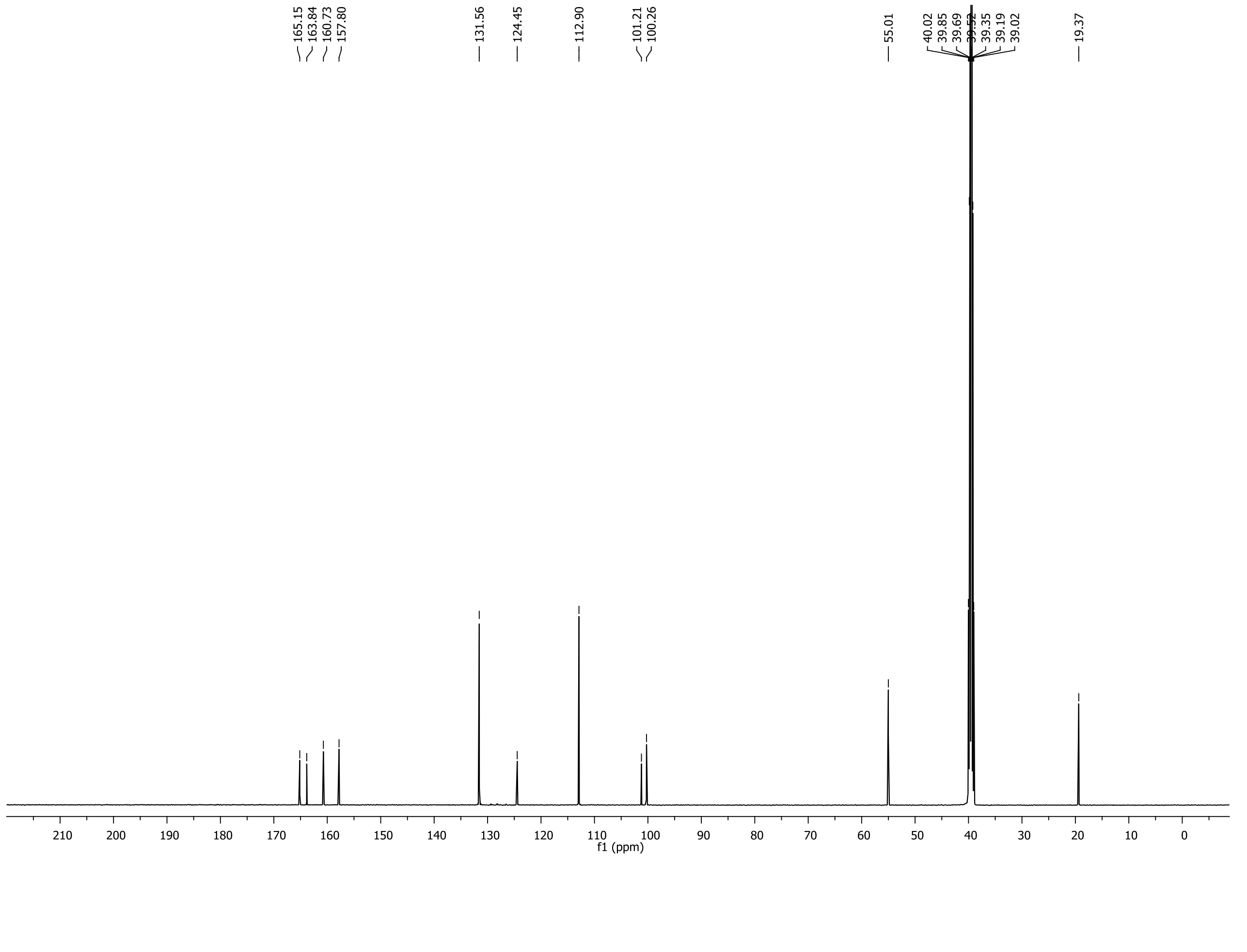
165.15
163.84
160.73
157.80
131.56
124.45
112.90
101.21
100.26
55.01
40.02
39.85
39.69
39.35
39.19
39.02
19.37
210 200 190 180 170 160 150 140 130 120 110 100 90 80 70 60 50 40 30 20 10 0
f1 (ppm)

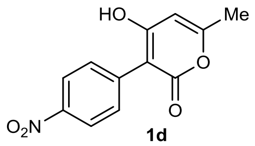
HO
Me
O
O
O2N

1d

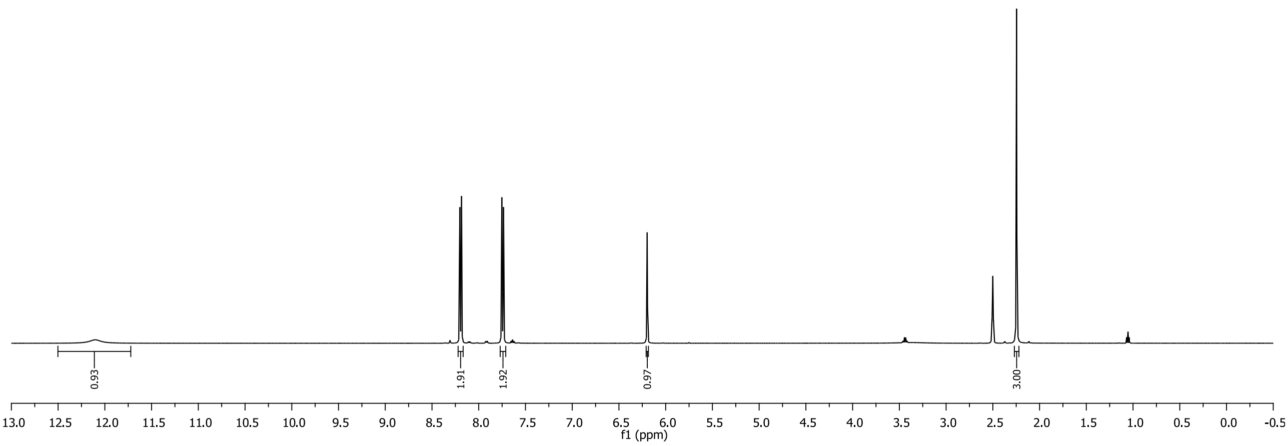
0.93
1.91
1.92
0.97
3.00
f1 (ppm)

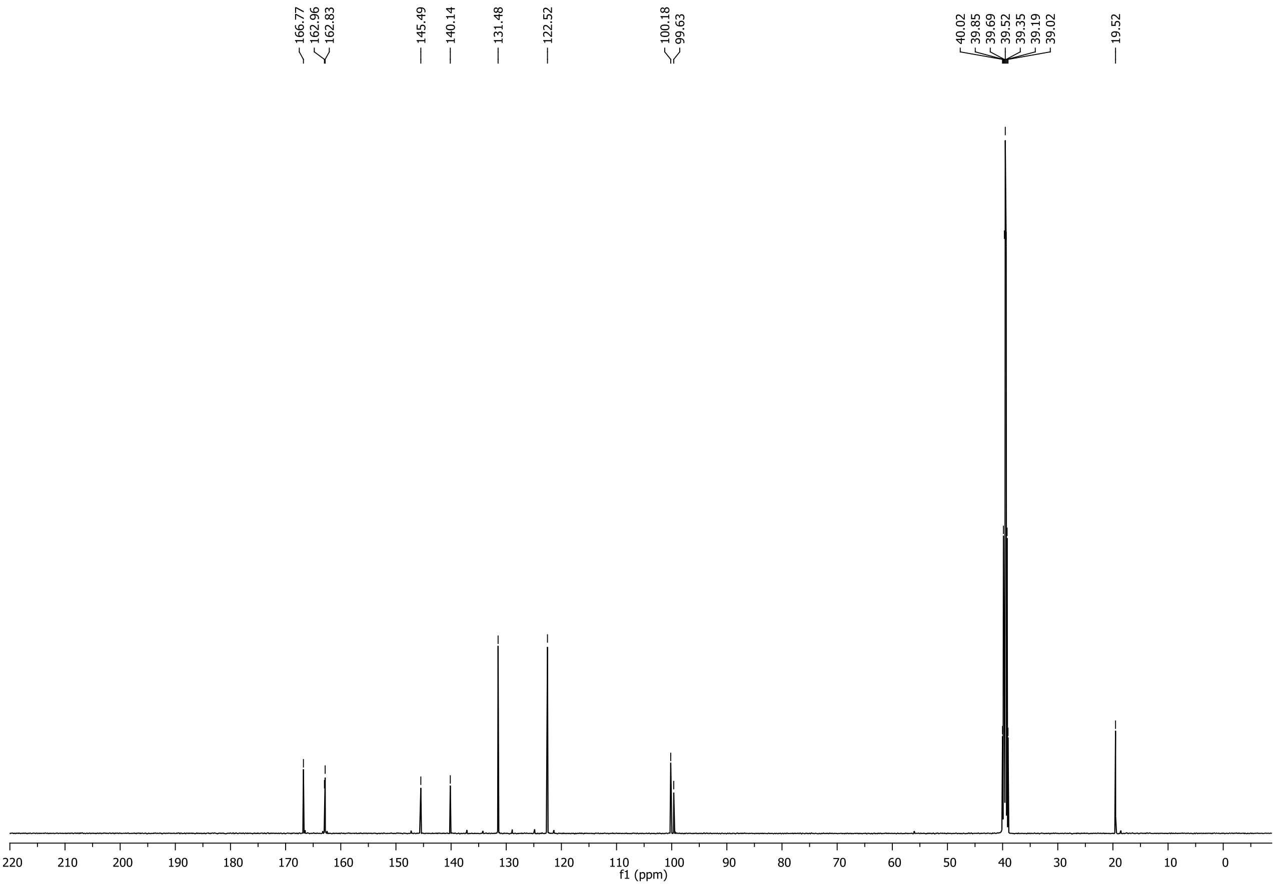
166.77
162.96
162.83
145.49
140.14
131.48
122.52
100.18
99.63
40.02
39.85
39.69
39.52
39.35
39.19
39.02
19.52
f1 (ppm)

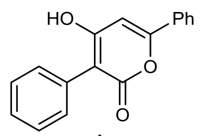
HO
Ph
O
O

1e

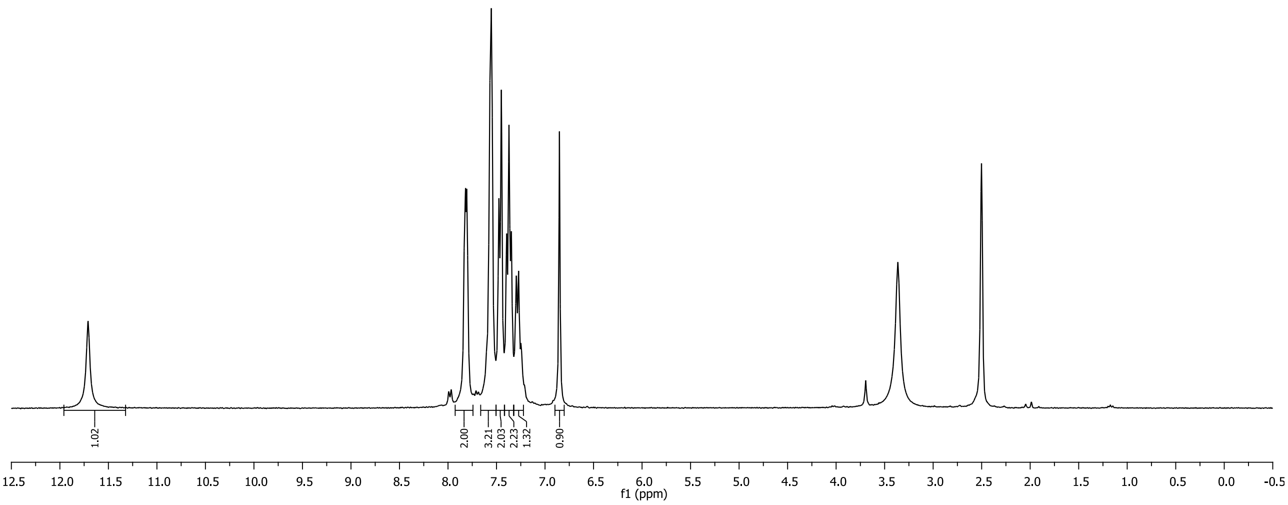
1.02
2.00
3.21
2.03
2.23
1.32
0.90
f1 (ppm)

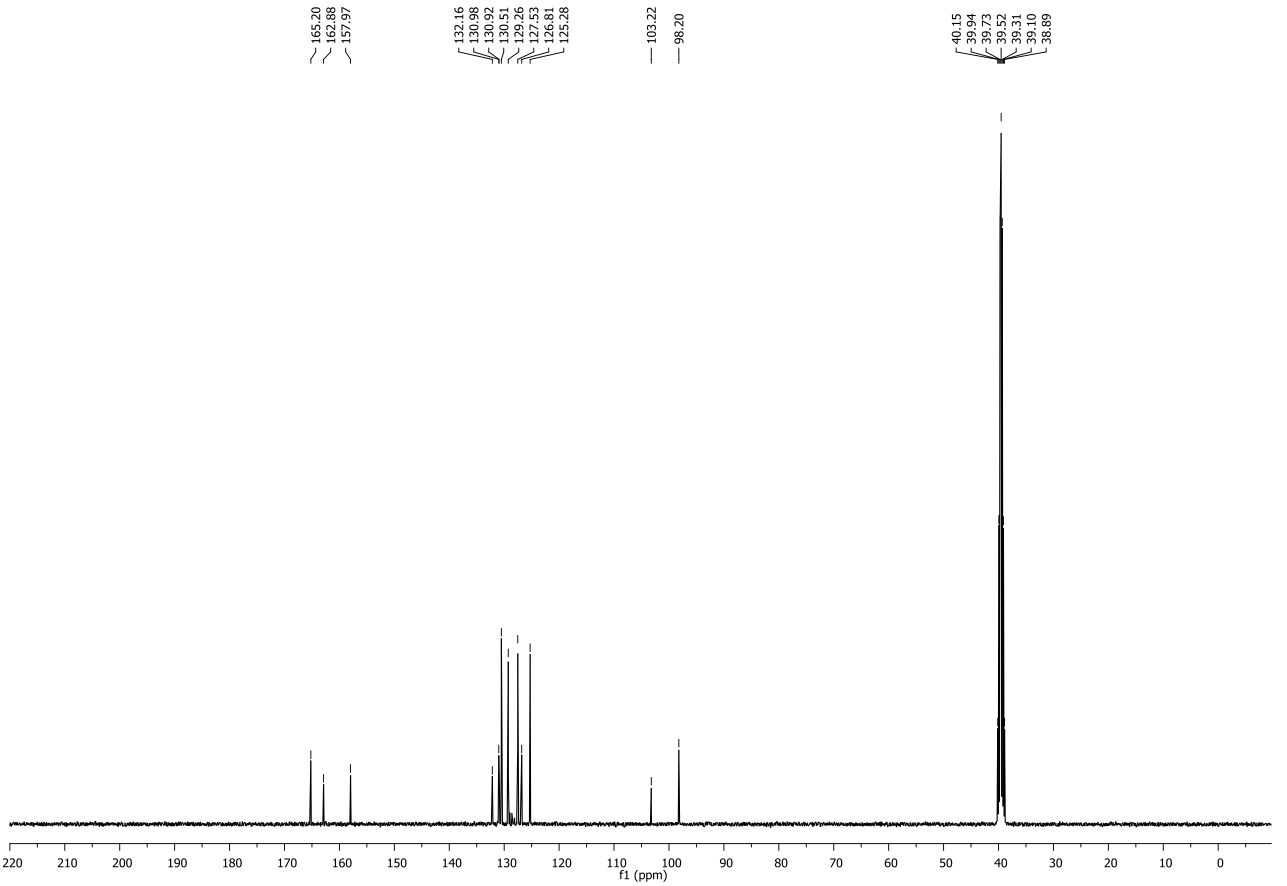
165.20
162.88
157.97
132.16
130.98
130.92
130.51
129.26
127.53
126.81
125.28
103.22
98.20
40.15
39.94
39.73
39.52
39.31
39.10
38.89
f1 (ppm)

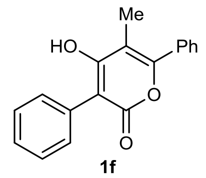
Me
HO
Ph
O
O

1f

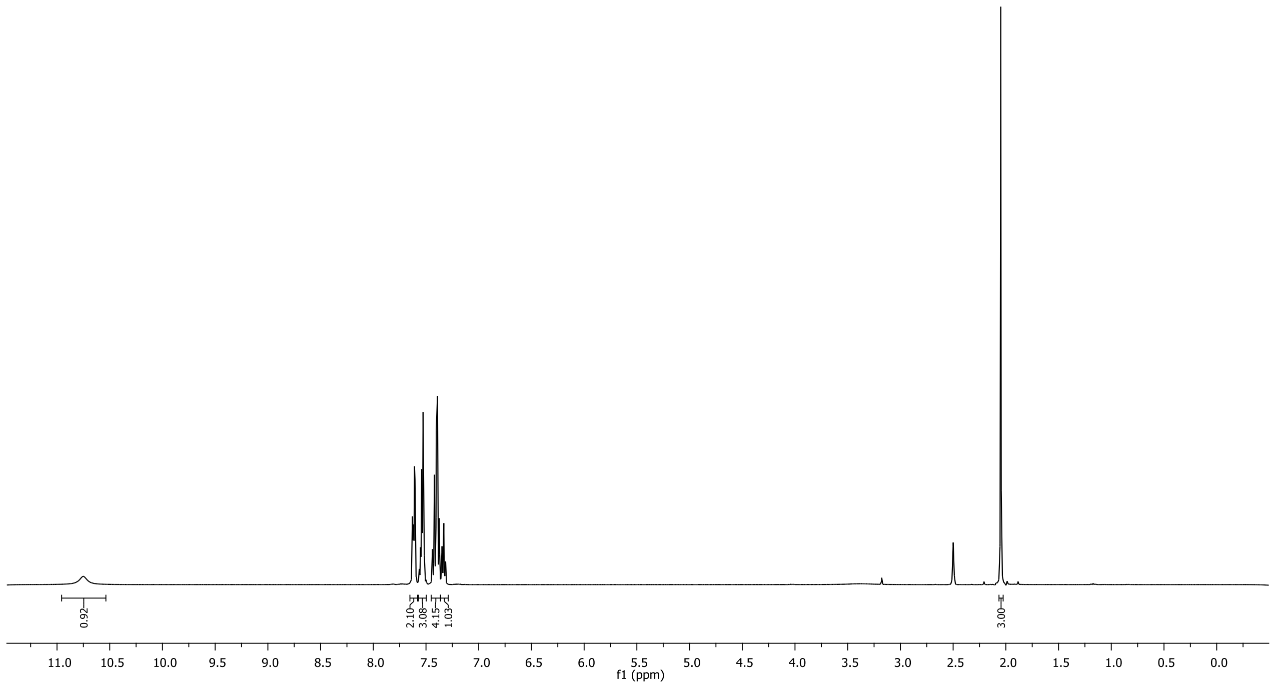
0.92
2.10
3.08
4.15
1.03
3.00
11.0 10.5 10.0 9.5 9.0 8.5 8.0 7.5 7.0 6.5 6.0 5.5 5.0 4.5 4.0 3.5 3.0 2.5 2.0 1.5 1.0 0.5 0.0
f1 (ppm)

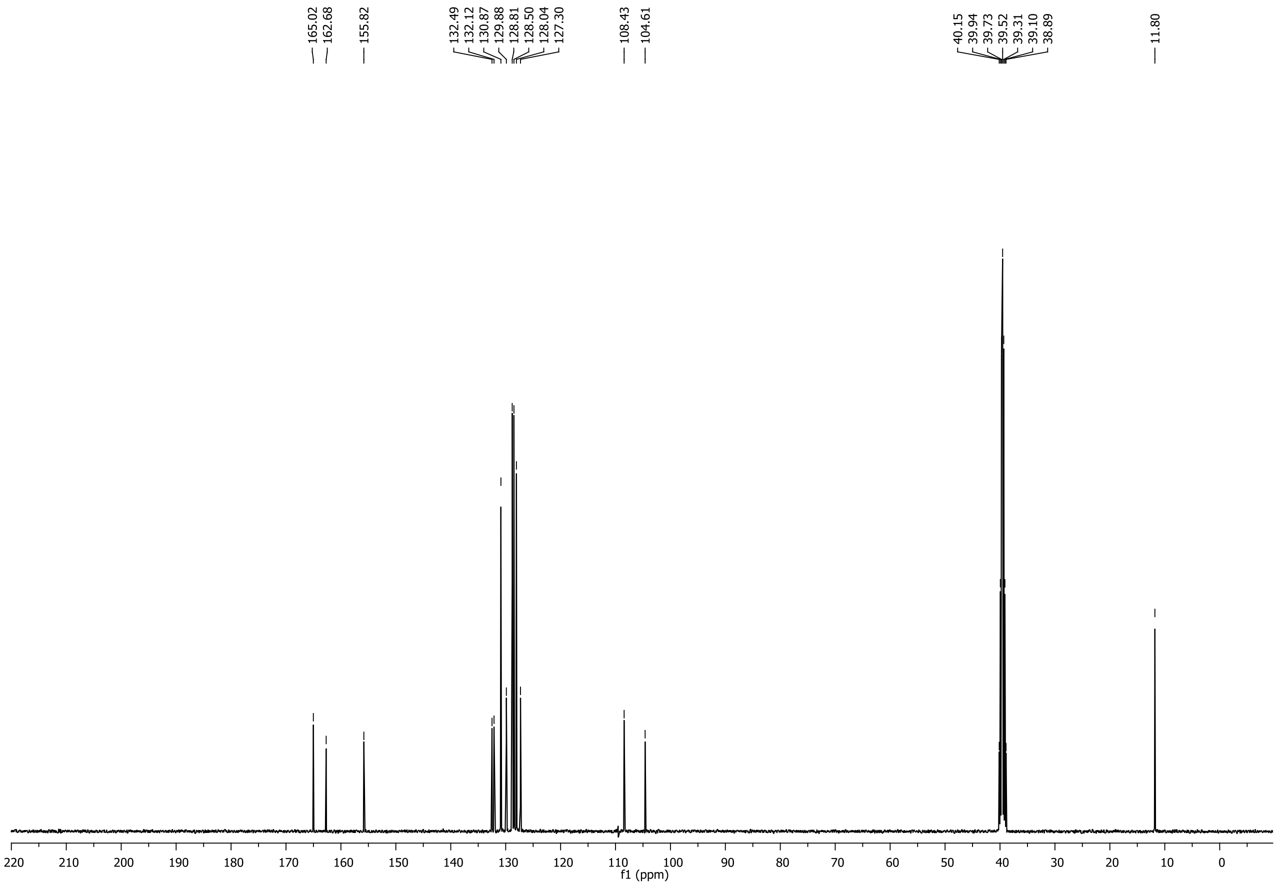
165.02
162.68
155.82
132.49
132.12
130.87
129.88
128.81
128.50
128.04
127.30
108.43
104.61
40.15
39.94
39.73
39.52
39.31
39.10
38.89
11.80
220 210 200 190 180 170 160 150 140 130 120 110 100 90 80 70 60 50 40 30 20 10 0
f1 (ppm)

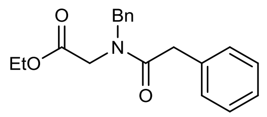
O
Bn
N
EtO
O

S10

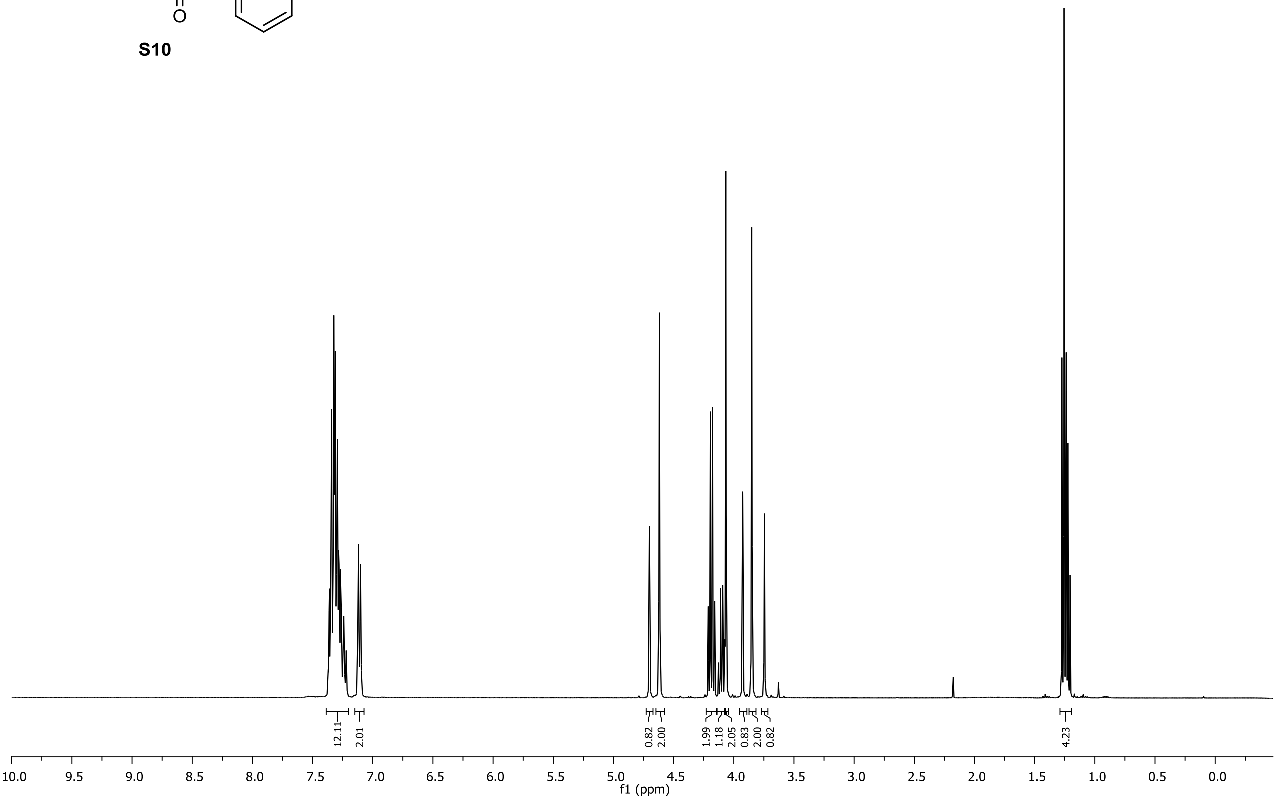
12.11
2.01
0.82
2.00
1.99
1.18
2.05
0.83
2.00
0.82
4.23
10.0 9.5 9.0 8.5 8.0 7.5 7.0 6.5 6.0 5.5 5.0 4.5 4.0 3.5 3.0 2.5 2.0 1.5 1.0 0.5 0.0
f1 (ppm)

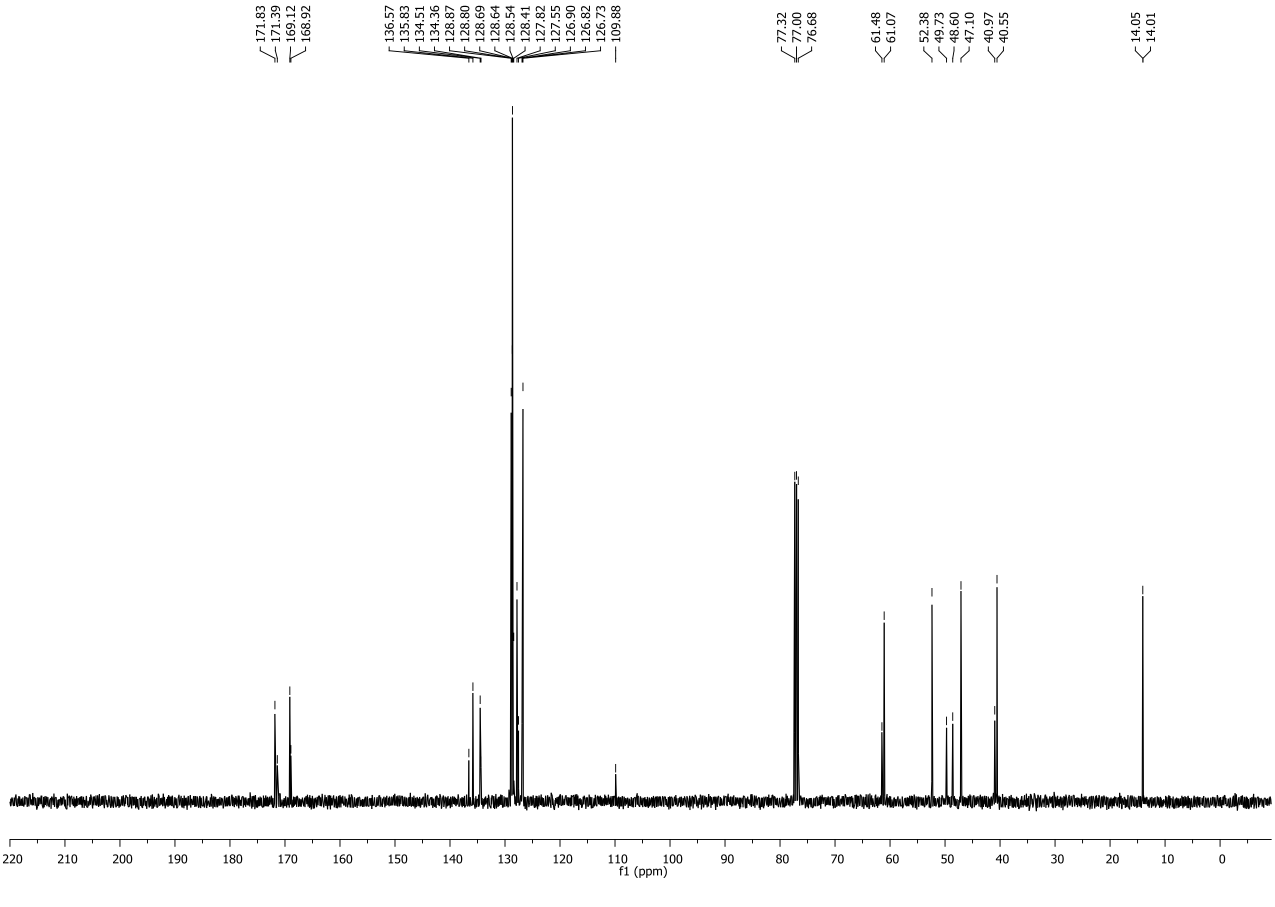
171.83
171.39
169.12
168.92
136.57
135.83
134.51
134.36
128.87
128.80
128.69
128.64
128.54
128.41
127.82
127.55
126.90
126.82
126.73
109.88
77.32
77.00
76.68
61.48
61.07
52.38
49.73
48.60
47.10
40.97
40.55
14.05
14.01
220 210 200 190 180 170 160 150 140 130 120 110 100 90 80 70 60 50 40 30 20 10 0
f1 (ppm)

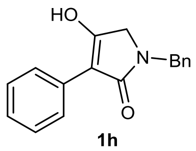
HO
N
Bn
O

1h

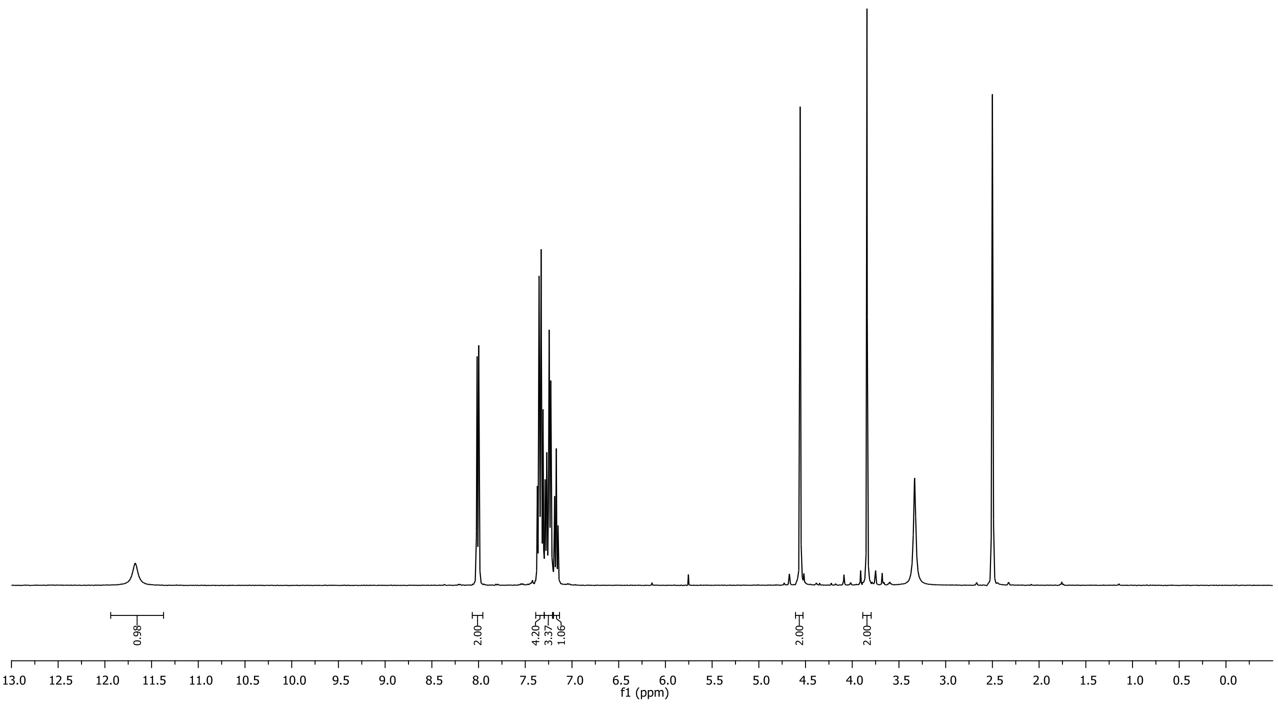
0.98
2.00
4.20
3.37
1.06
2.00
2.00
13.0 12.5 12.0 11.5 11.0 10.5 10.0 9.5 9.0 8.5 8.0 7.5 7.0 6.5 6.0 5.5 5.0 4.5 4.0 3.5 3.0 2.5 2.0 1.5 1.0 0.5 0.0
f1 (ppm)

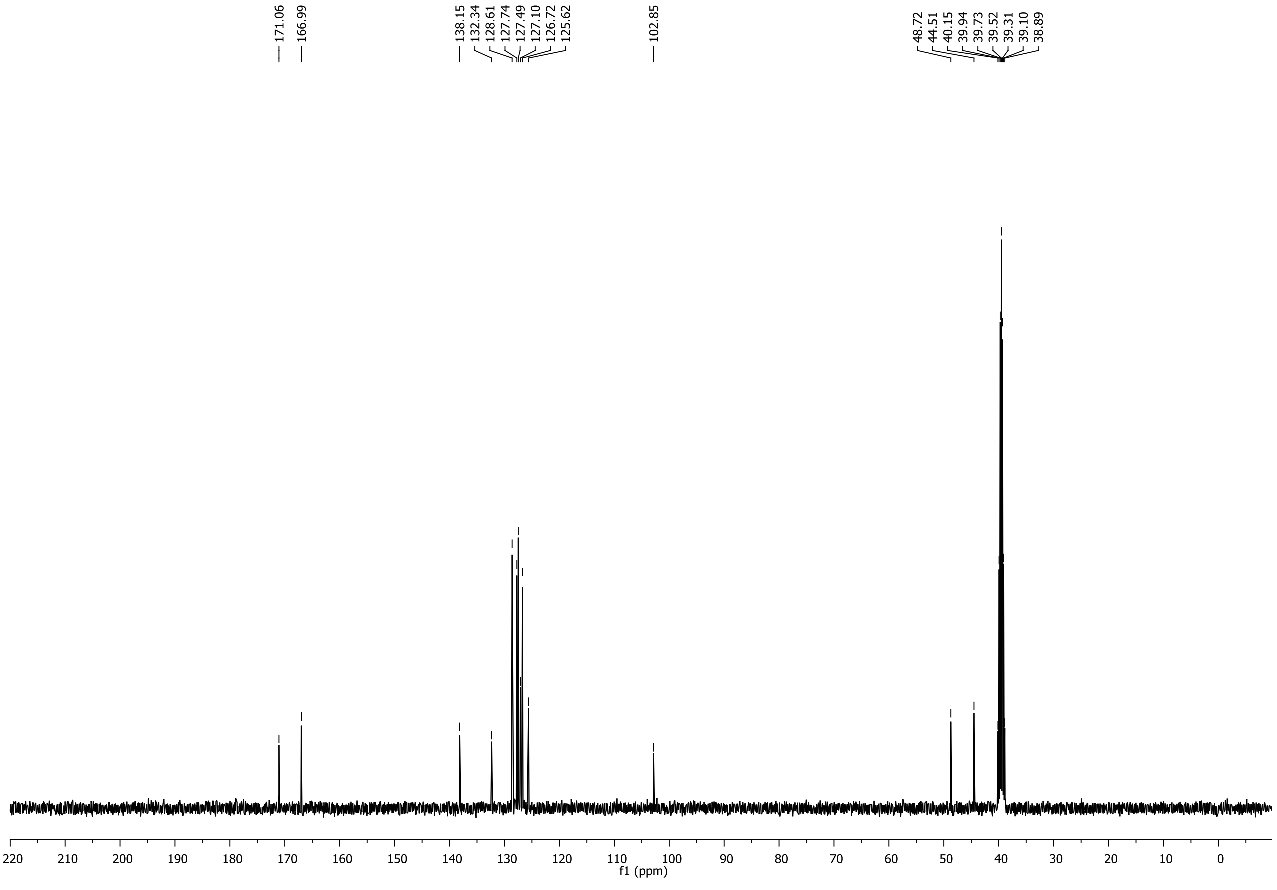
171.06
166.99
138.15
132.34
128.61
127.74
127.49
127.10
126.72
125.62
102.85
48.72
44.51
40.15
39.94
39.73
39.52
39.31
39.10
38.89
220 210 200 190 180 170 160 150 140 130 120 110 100 90 80 70 60 50 40 30 20 10 0
f1 (ppm)

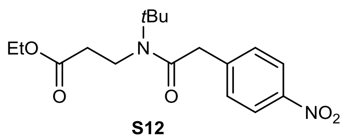
tBu
EtO
N
O
O
NO2

S12

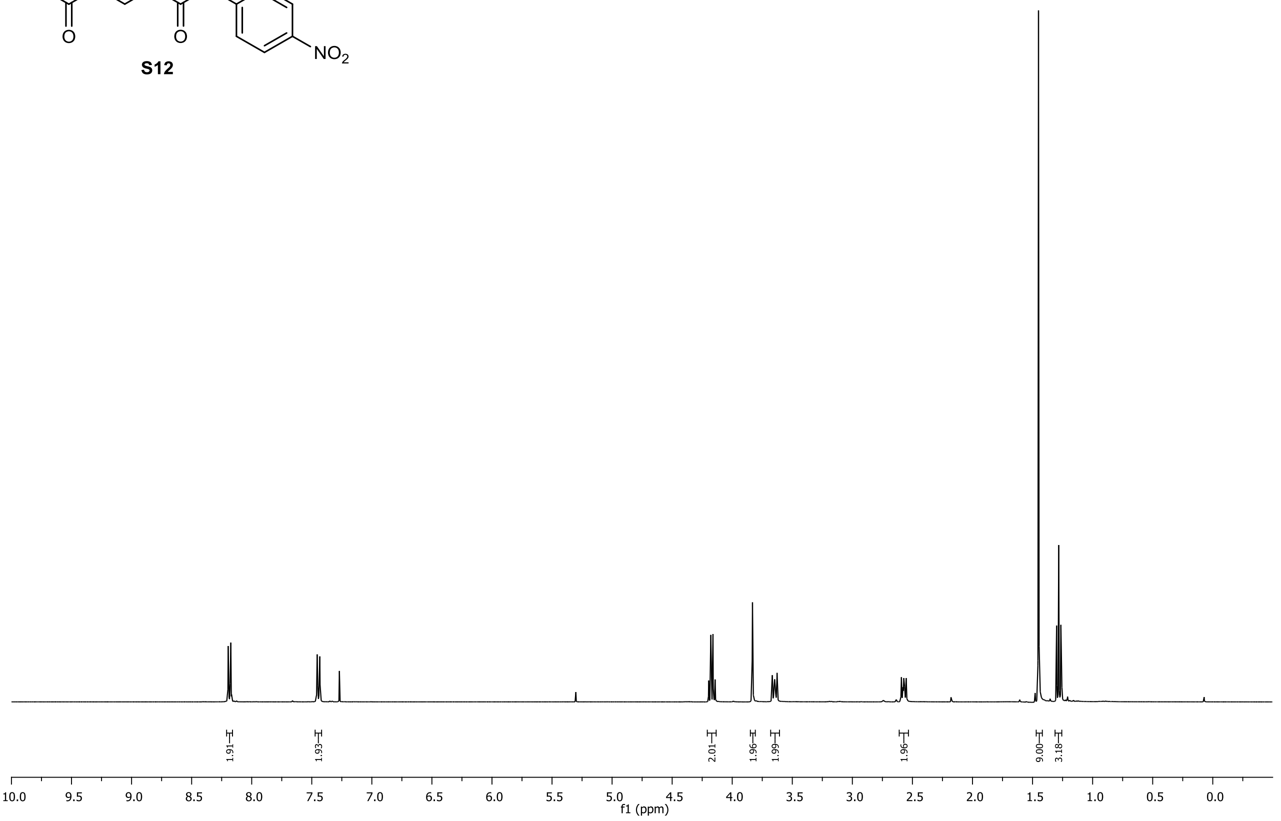
1.91
1.93
2.01
1.96
1.99
1.96
9.00
3.18
10.0 9.5 9.0 8.5 8.0 7.5 7.0 6.5 6.0 5.5 5.0 4.5 4.0 3.5 3.0 2.5 2.0 1.5 1.0 0.5 0.0
f1 (ppm)

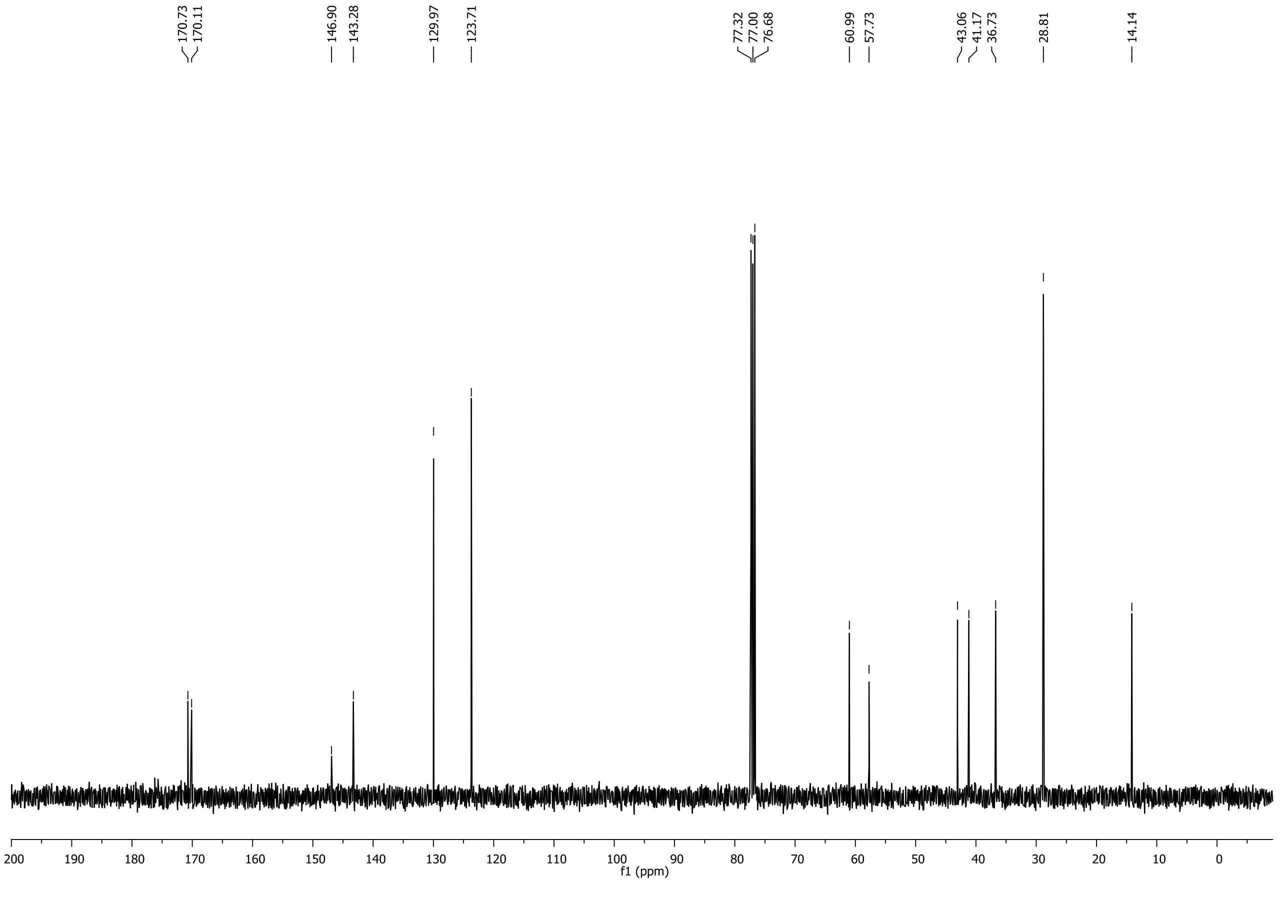
170.73
170.11
146.90
143.28
129.97
123.71
77.32
77.00
76.68
60.99
57.73
43.06
41.17
36.73
28.81
14.14
200 190 180 170 160 150 140 130 120 110 100 90 80 70 60 50 40 30 20 10 0
f1 (ppm)

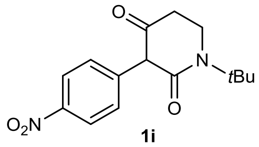
O
N
tBu
O
O_2N

1i

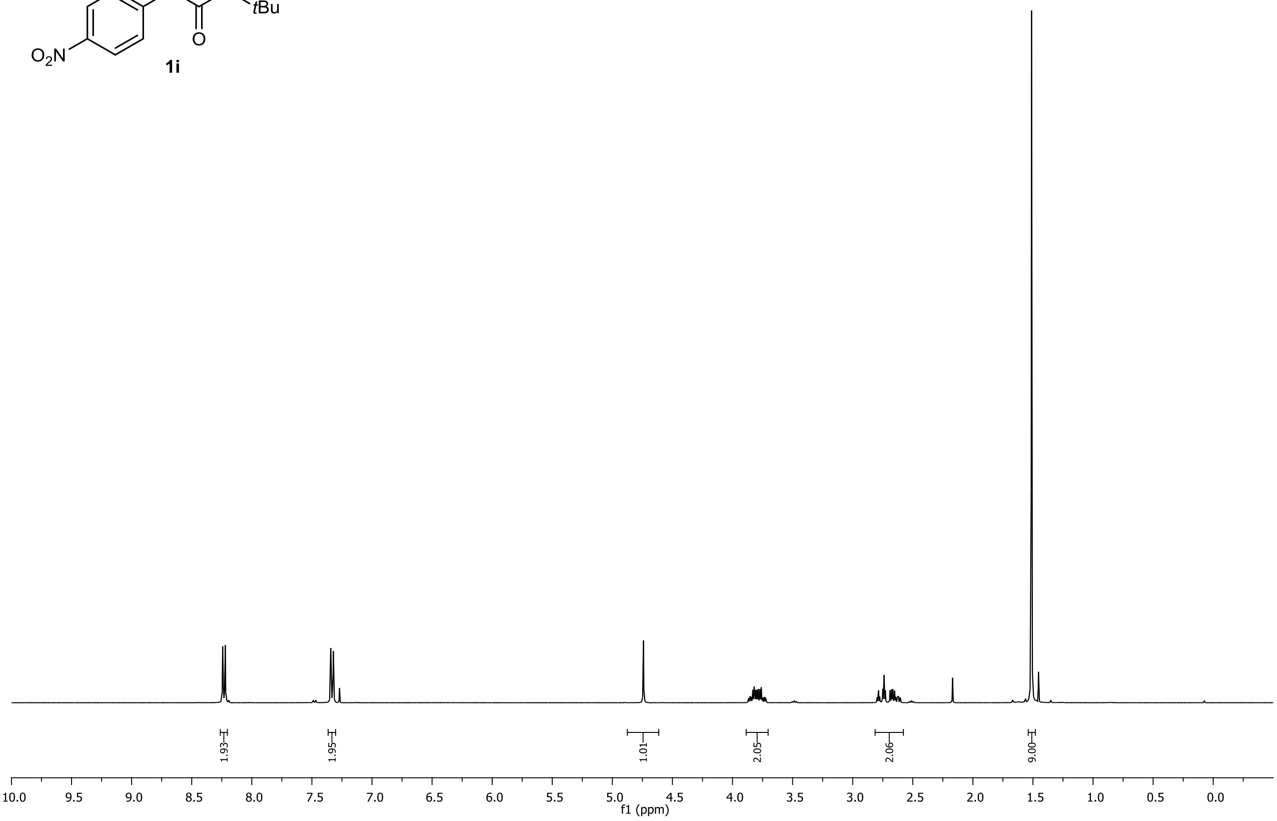
1.93
1.95
1.01
2.05
2.06
9.00
10.0 9.5 9.0 8.5 8.0 7.5 7.0 6.5 6.0 5.5 5.0 4.5 4.0 3.5 3.0 2.5 2.0 1.5 1.0 0.5 0.0
f1 (ppm)

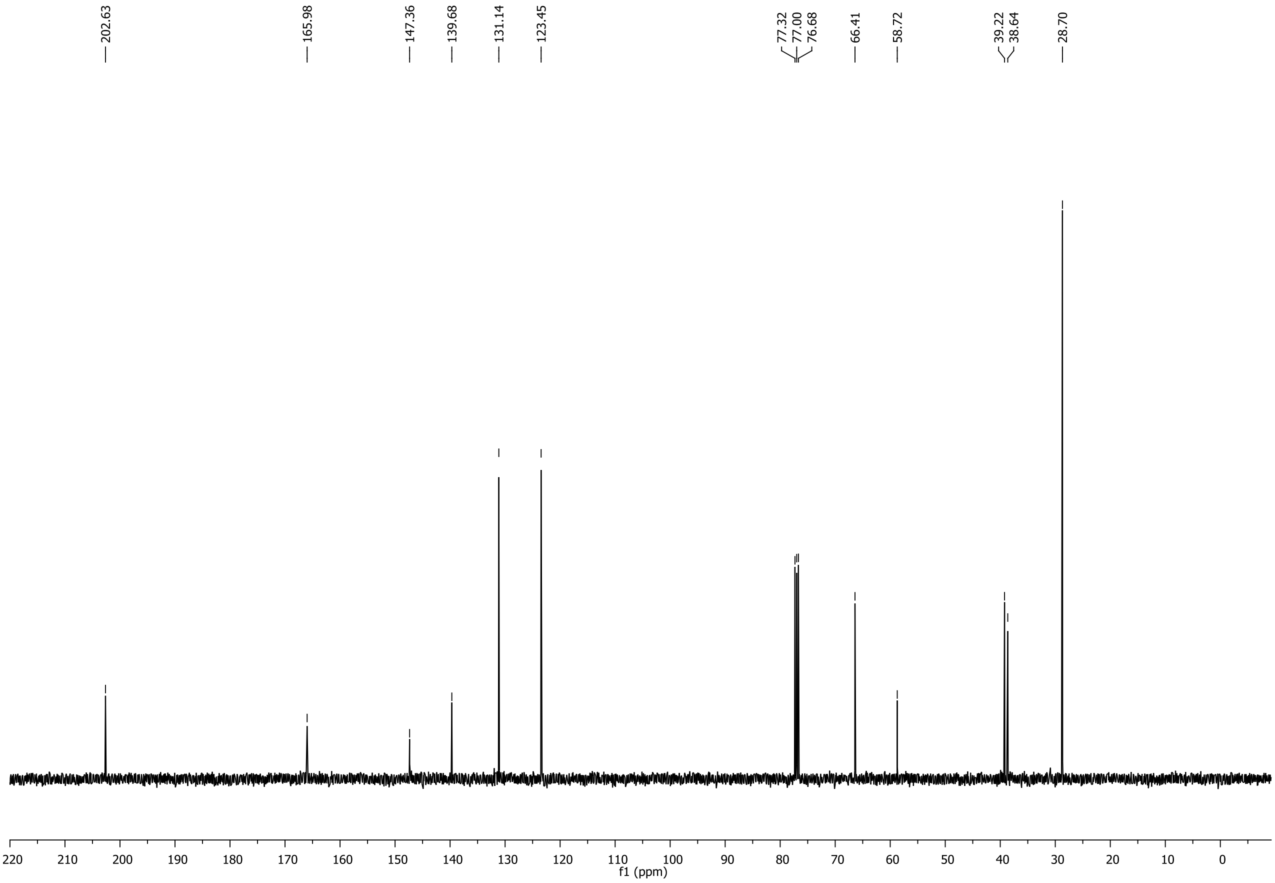
202.63
165.98
147.36
139.68
131.14
123.45
77.32
77.00
76.68
66.41
58.72
39.22
38.64
28.70
220 210 200 190 180 170 160 150 140 130 120 110 100 90 80 70 60 50 40 30 20 10 0
f1 (ppm)

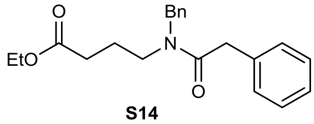
O
Bn
N
EtO
O

S14

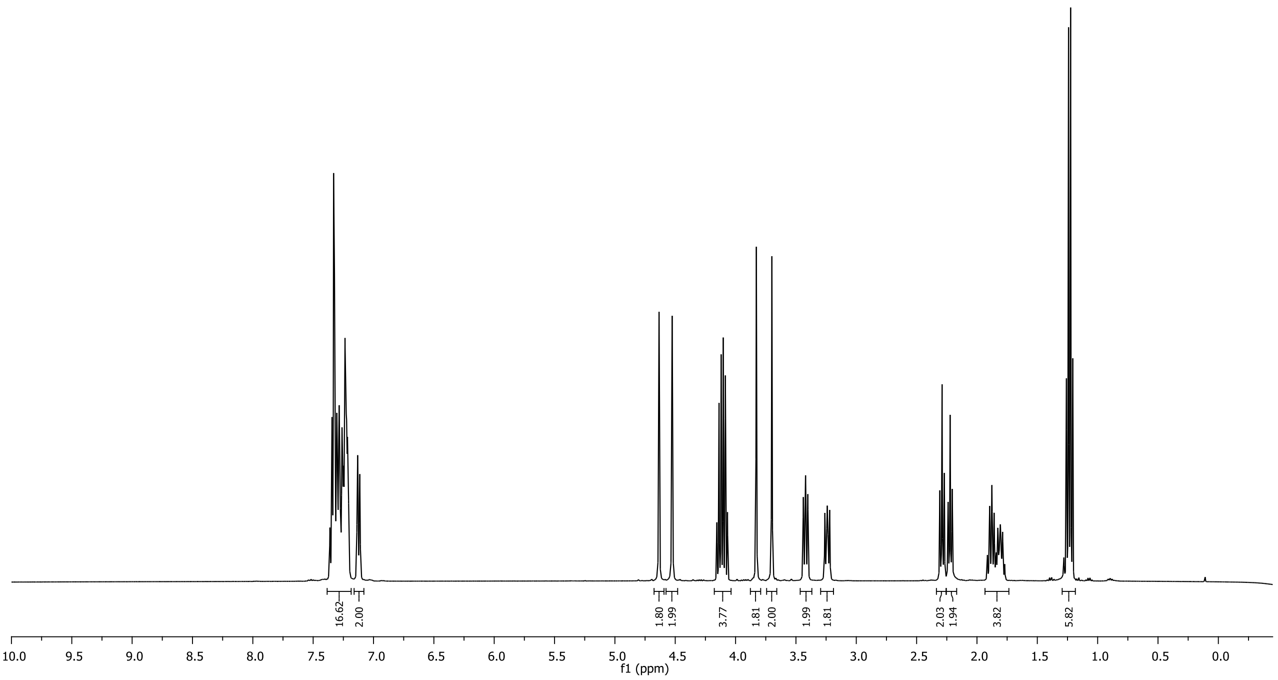
16.62
2.00
1.80
1.99
3.77
1.81
2.00
1.99
1.81
2.03
1.94
3.82
5.82
10.0 9.5 9.0 8.5 8.0 7.5 7.0 6.5 6.0 5.5 5.0 4.5 4.0 3.5 3.0 2.5 2.0 1.5 1.0 0.5 0.0
f1 (ppm)

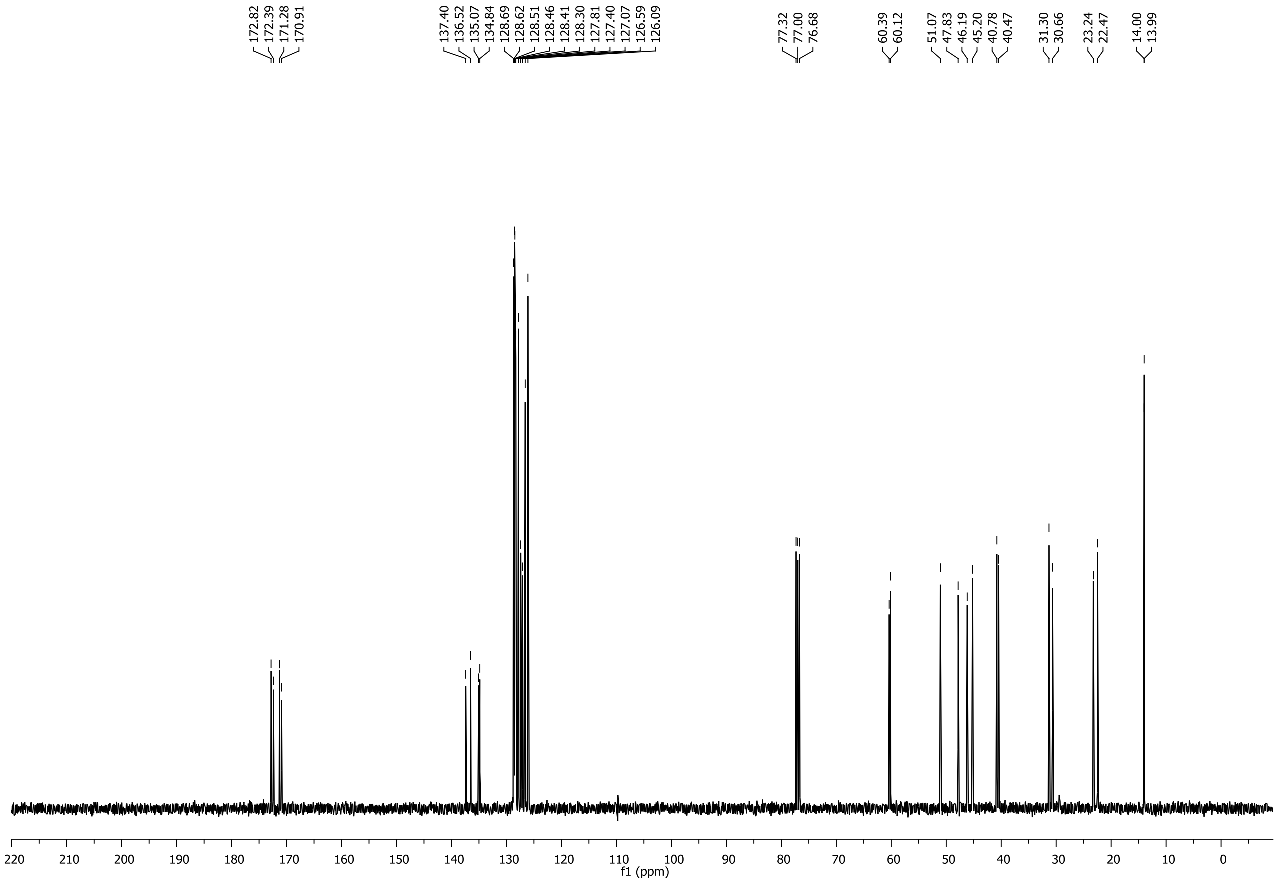
172.82
172.39
171.28
170.91
137.40
136.52
135.07
134.84
128.69
128.62
128.51
128.46
128.41
128.30
127.81
127.40
127.07
126.59
126.09
77.32
77.00
76.68
60.39
60.12
51.07
47.83
46.19
45.20
40.78
40.47
31.30
30.66
23.24
22.47
14.00
13.99
220 210 200 190 180 170 160 150 140 130 120 110 100 90 80 70 60 50 40 30 20 10 0
f1 (ppm)

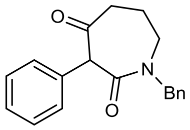
O
N
Bn
O

1j

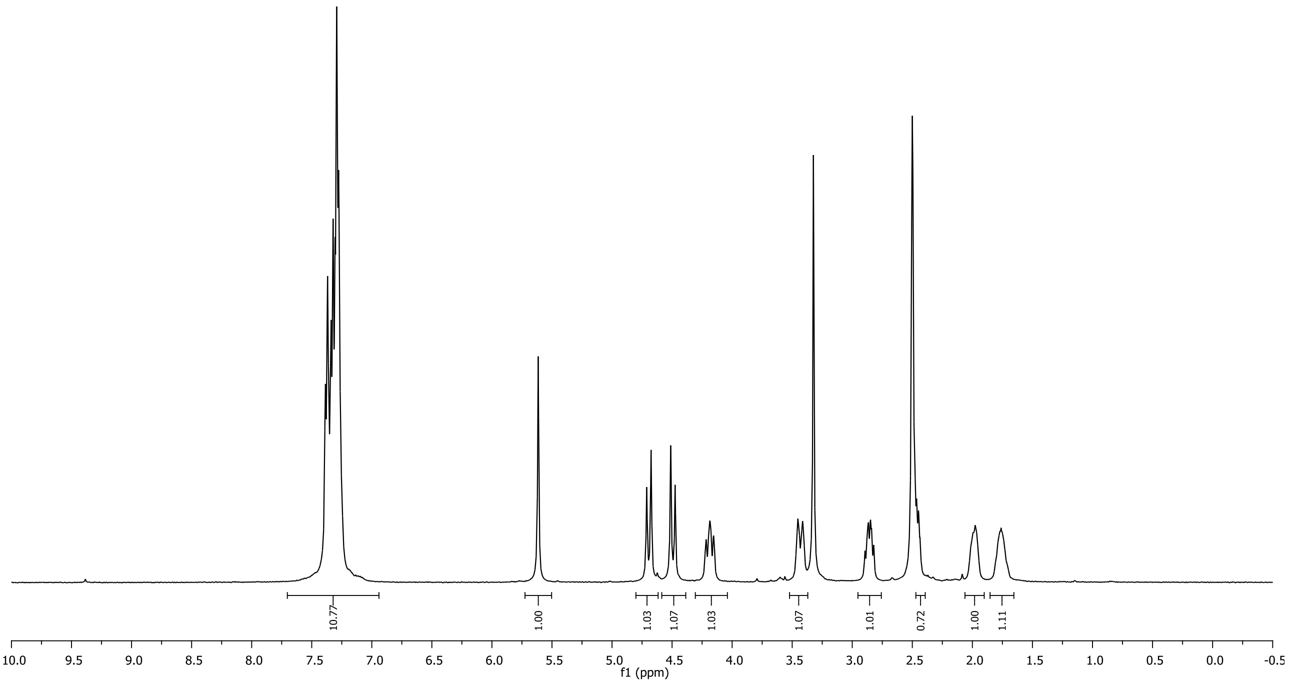
10.77
1.00
1.03
1.07
1.03
1.07
1.01
0.72
1.00
1.11
10.0 9.5 9.0 8.5 8.0 7.5 7.0 6.5 6.0 5.5 5.0 4.5 4.0 3.5 3.0 2.5 2.0 1.5 1.0 0.5 0.0 -0.5
f1 (ppm)

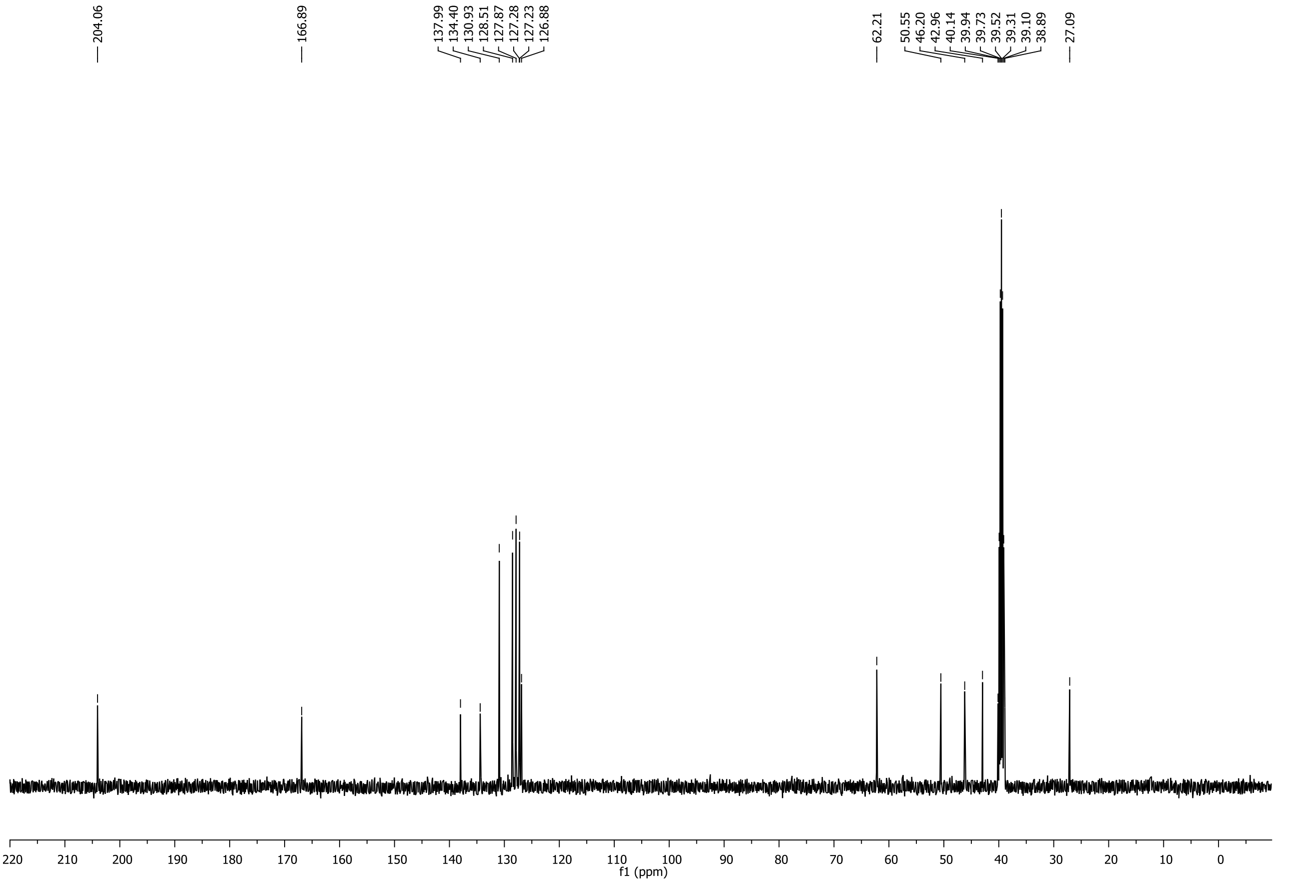
204.06
166.89
137.99
134.40
130.93
128.51
127.87
127.28
127.23
126.88
62.21
50.55
46.20
42.96
40.14
39.94
39.73
39.52
39.31
39.10
38.89
27.09
220 210 200 190 180 170 160 150 140 130 120 110 100 90 80 70 60 50 40 30 20 10 0
f1 (ppm)

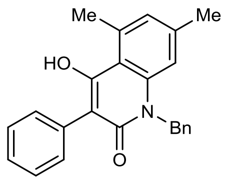
Me
Me
HO
N
Bn
O

1k

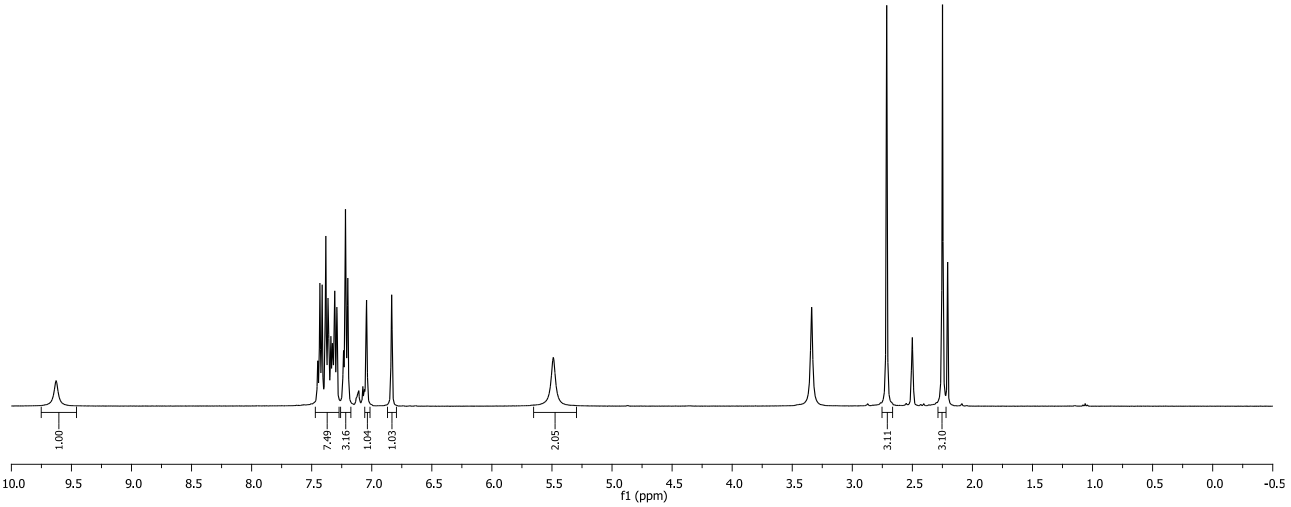
1.00
7.49
3.16
1.04
1.03
2.05
3.11
3.10
f1 (ppm)

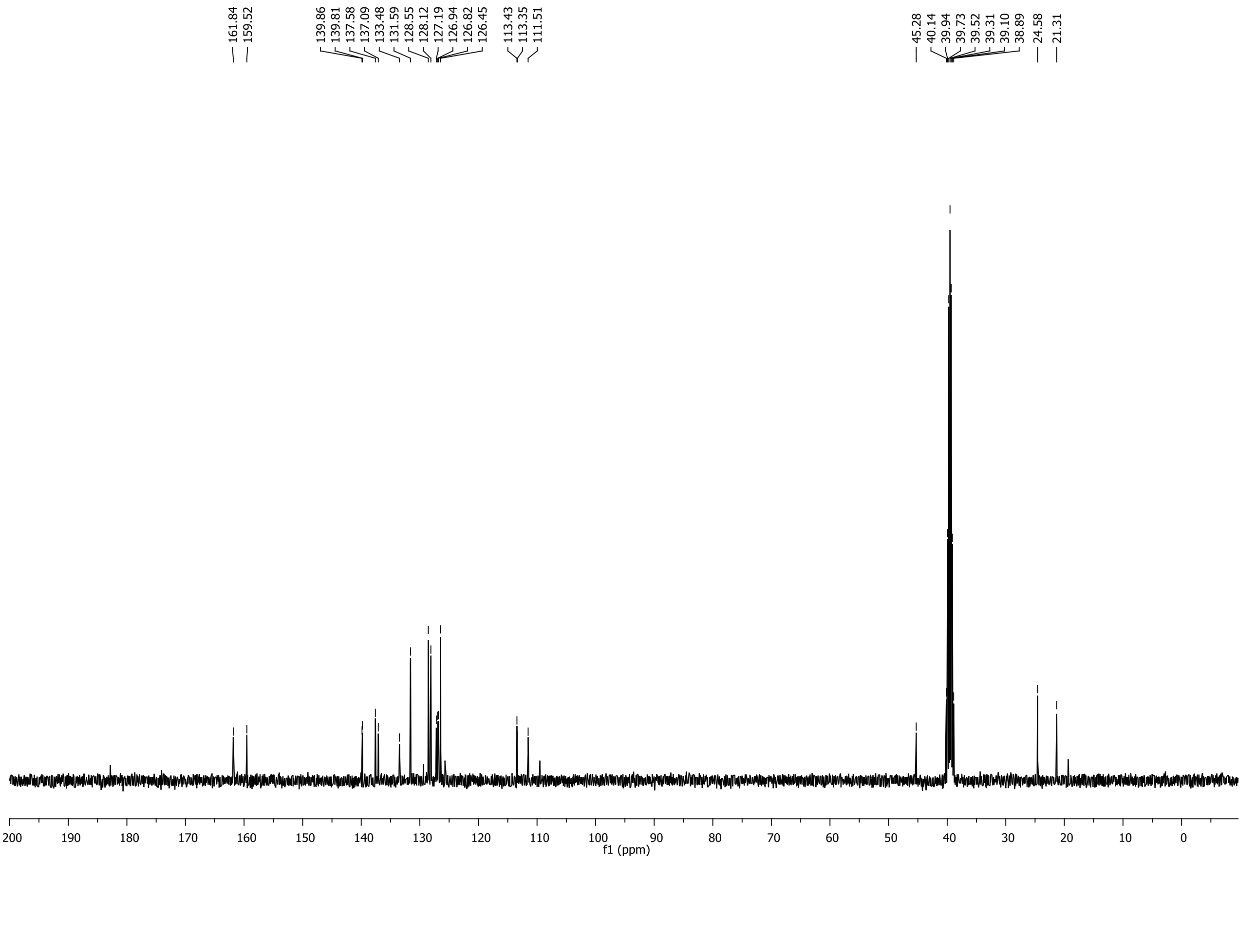
161.84
159.52
139.86
139.81
137.58
137.09
133.48
131.59
128.55
128.12
127.19
126.94
126.82
126.45
113.43
113.35
111.51
45.28
40.14
39.94
39.73
39.52
39.31
39.10
38.89
24.58
21.31
f1 (ppm)

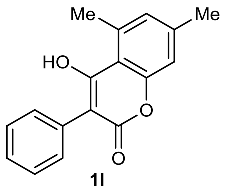
Me
Me
HO
O
O

1l

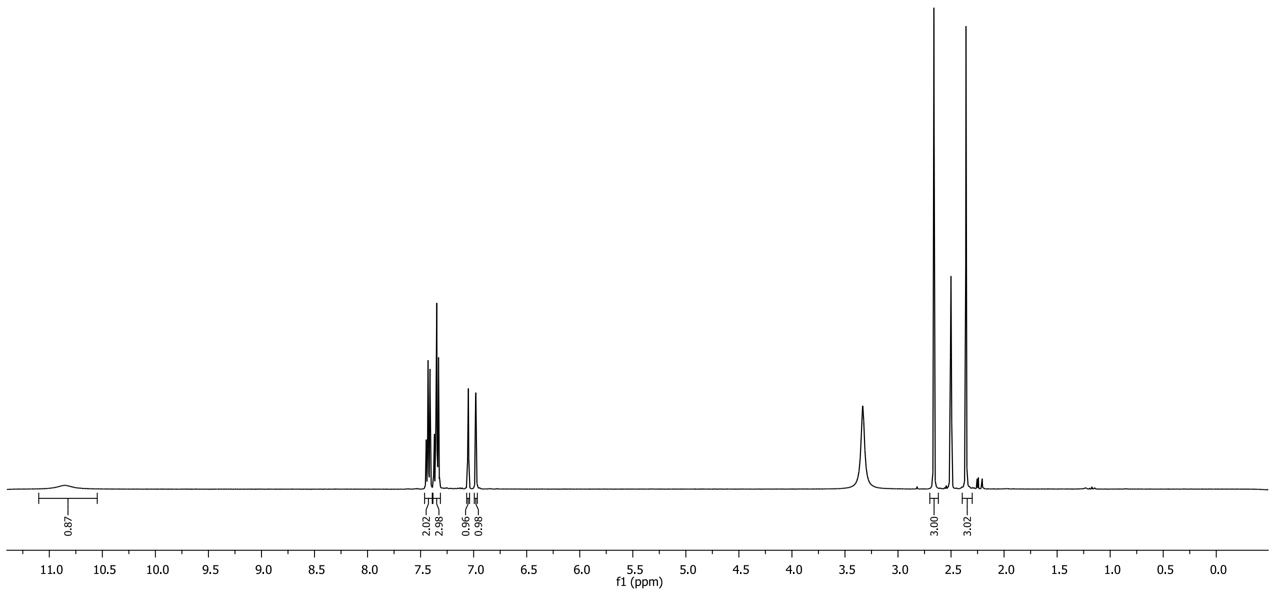
0.87
2.02
2.98
0.96
0.98
3.00
3.02
f1 (ppm)

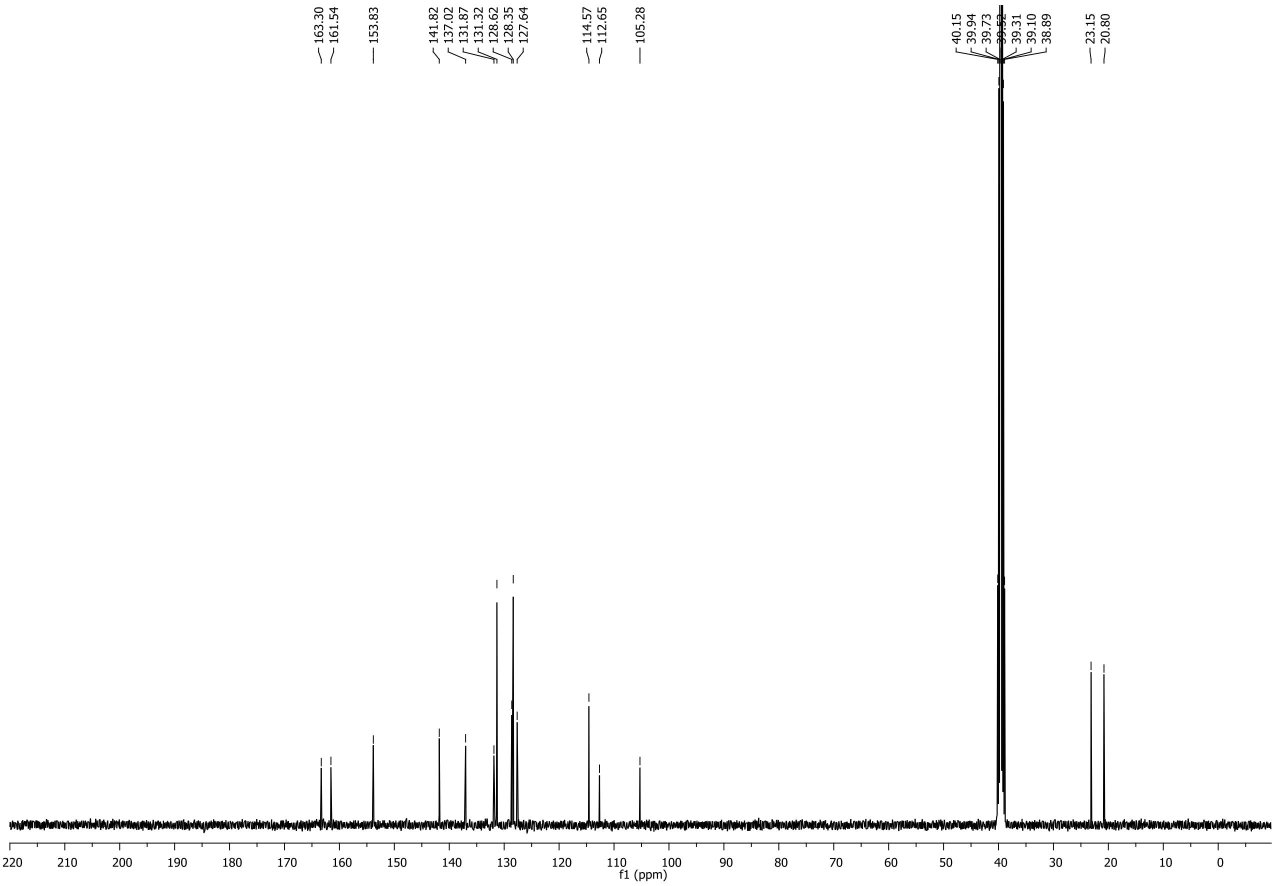
163.30
161.54
153.83
141.82
137.02
131.87
131.32
128.62
128.35
127.64
114.57
112.65
105.28
40.15
39.94
39.73
39.52
39.31
39.10
38.89
23.15
20.80
f1 (ppm)

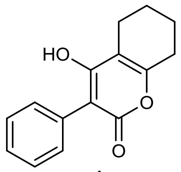

1m

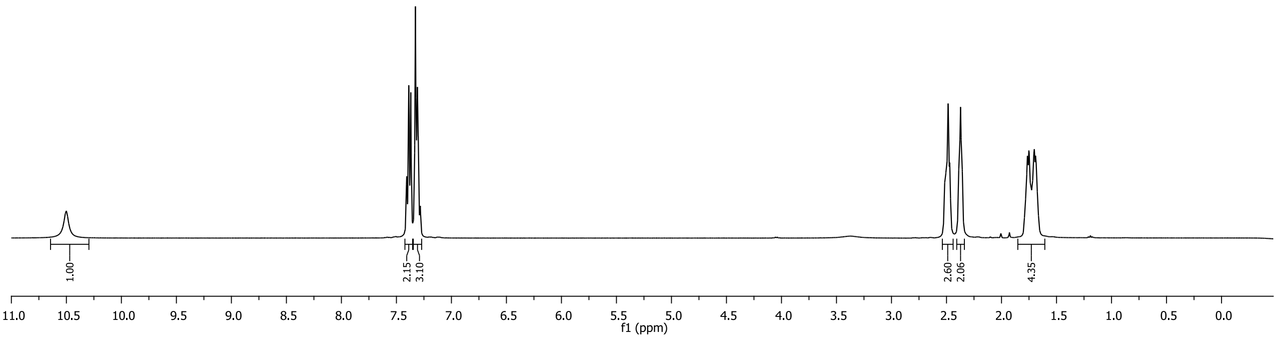
1.00
2.15
3.10
2.60
2.06
4.35
11.0 10.5 10.0 9.5 9.0 8.5 8.0 7.5 7.0 6.5 6.0 5.5 5.0 4.5 4.0 3.5 3.0 2.5 2.0 1.5 1.0 0.5 0.0
f1 (ppm)

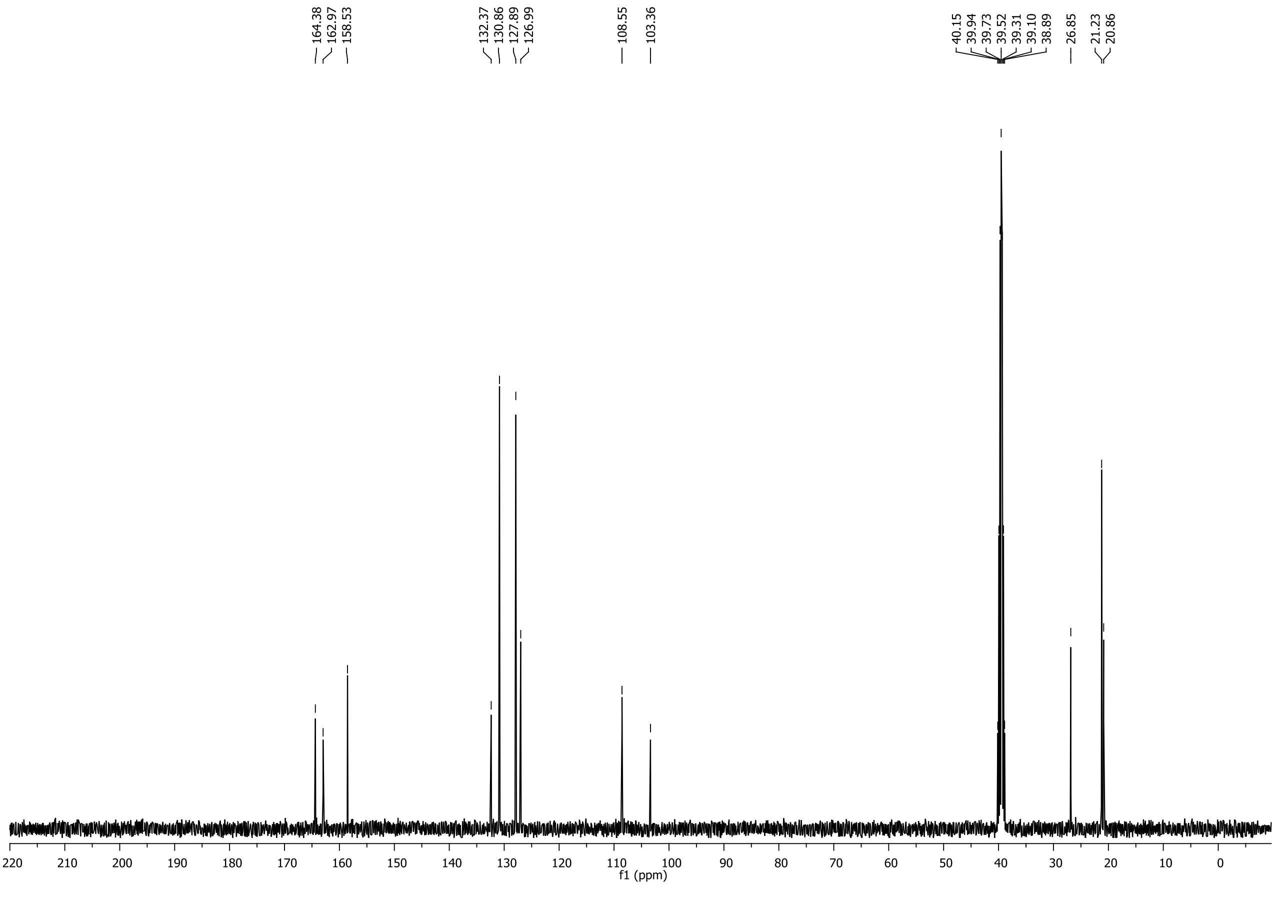
164.38
162.97
158.53
132.37
130.86
127.89
126.99
108.55
103.36
40.15
39.94
39.73
39.52
39.31
39.10
38.89
26.85
21.23
20.86
220 210 200 190 180 170 160 150 140 130 120 110 100 90 80 70 60 50 40 30 20 10 0
f1 (ppm)

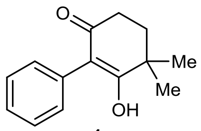
O
Me
Me
OH

1n

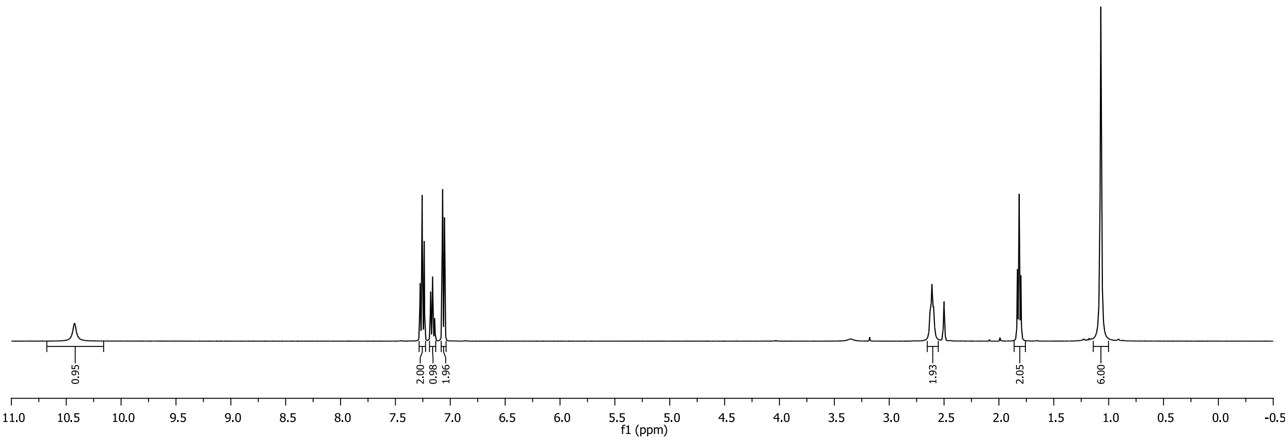
0.95
2.00
0.98
1.96
1.93
2.05
6.00
11.0 10.5 10.0 9.5 9.0 8.5 8.0 7.5 7.0 6.5 6.0 5.5 5.0 4.5 4.0 3.5 3.0 2.5 2.0 1.5 1.0 0.5 0.0 -0.5
f1 (ppm)

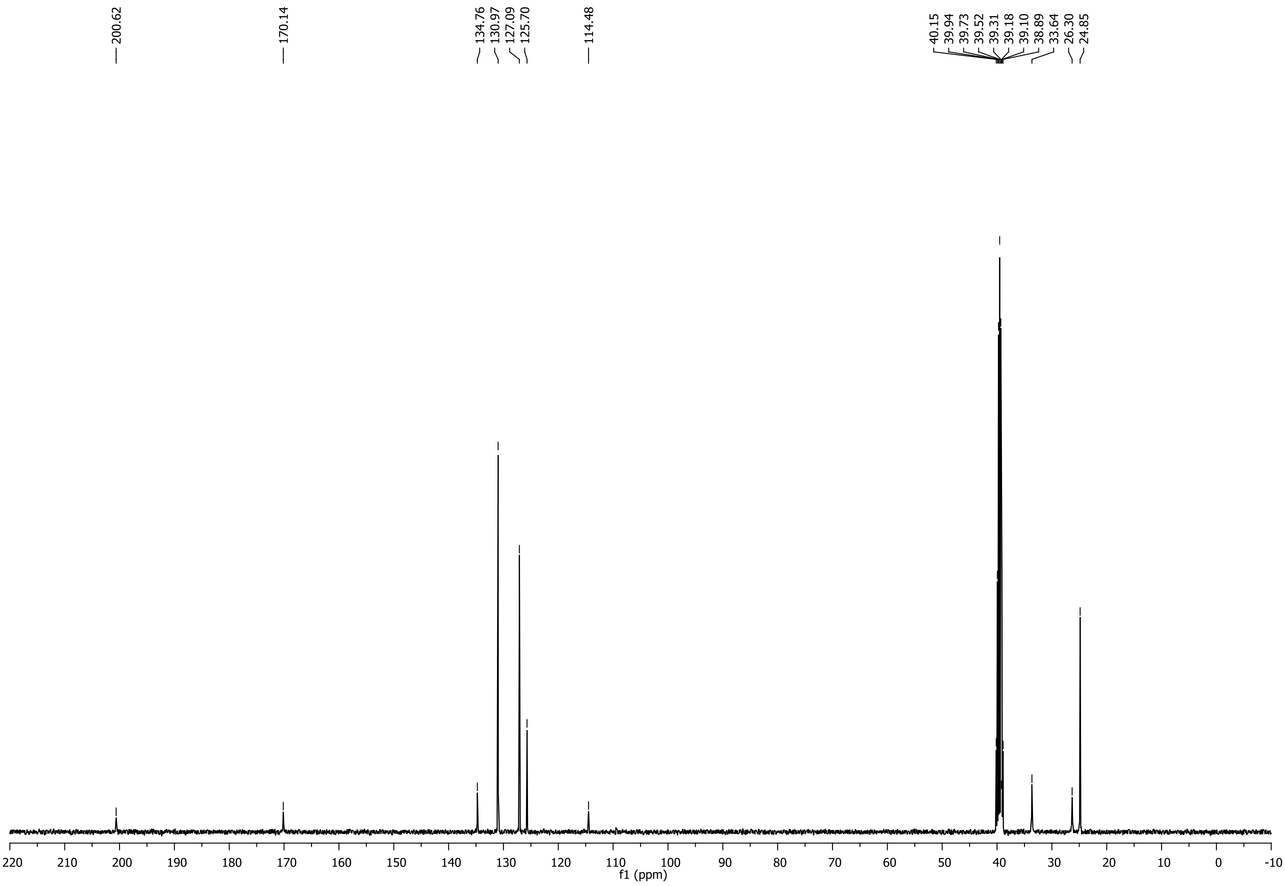
200.62
170.14
134.76
130.97
127.09
125.70
114.48
40.15
39.94
39.73
39.52
39.31
39.18
39.10
38.89
33.64
26.30
24.85
220 210 200 190 180 170 160 150 140 130 120 110 100 90 80 70 60 50 40 30 20 10 0 -10
f1 (ppm)

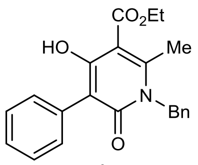
CO2Et
HO
Me
N
Bn
O

1o

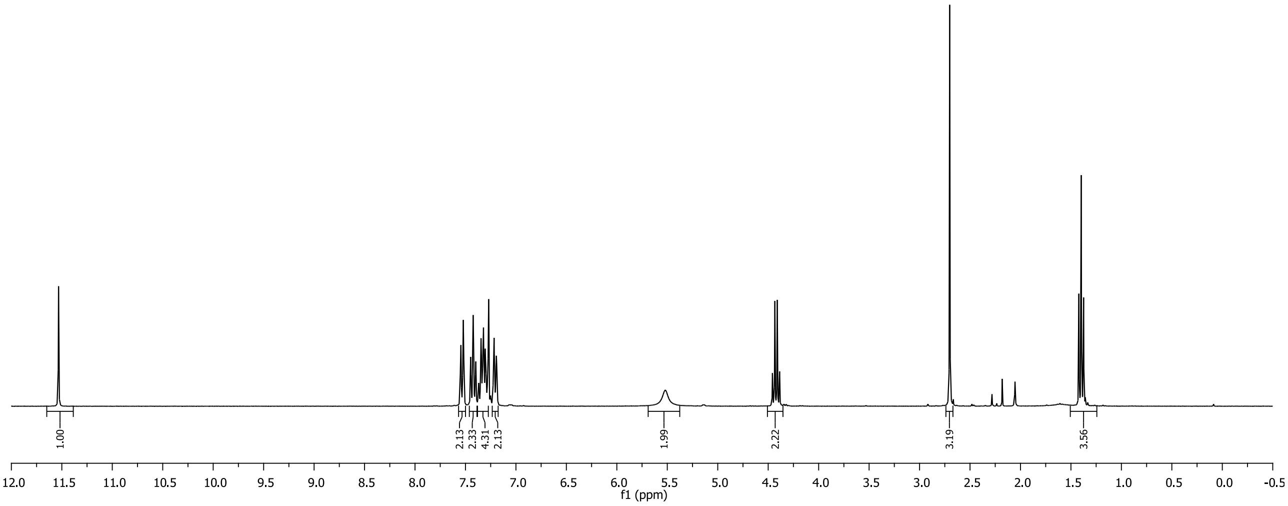
1.00
2.13
2.33
4.31
2.13
1.99
2.22
3.19
3.56
12.0 11.5 11.0 10.5 10.0 9.5 9.0 8.5 8.0 7.5 7.0 6.5 6.0 5.5 5.0 4.5 4.0 3.5 3.0 2.5 2.0 1.5 1.0 0.5 0.0 -0.5
f1 (ppm)

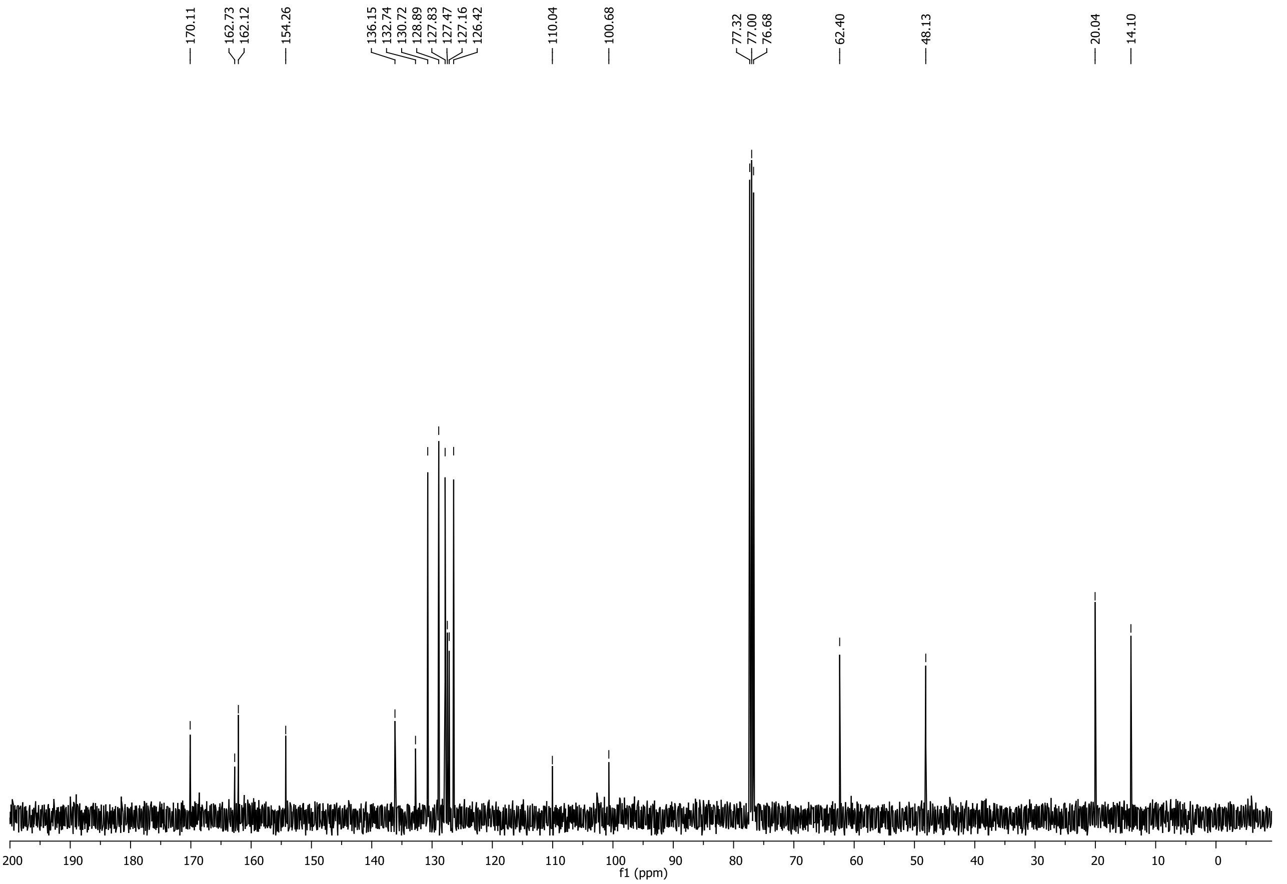
170.11
162.73
162.12
154.26
136.15
132.74
130.72
128.89
127.83
127.47
127.16
126.42
110.04
100.68
77.32
77.00
76.68
62.40
48.13
20.04
14.10
200 190 180 170 160 150 140 130 120 110 100 90 80 70 60 50 40 30 20 10 0
f1 (ppm)

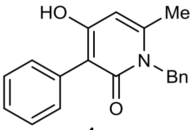

1p

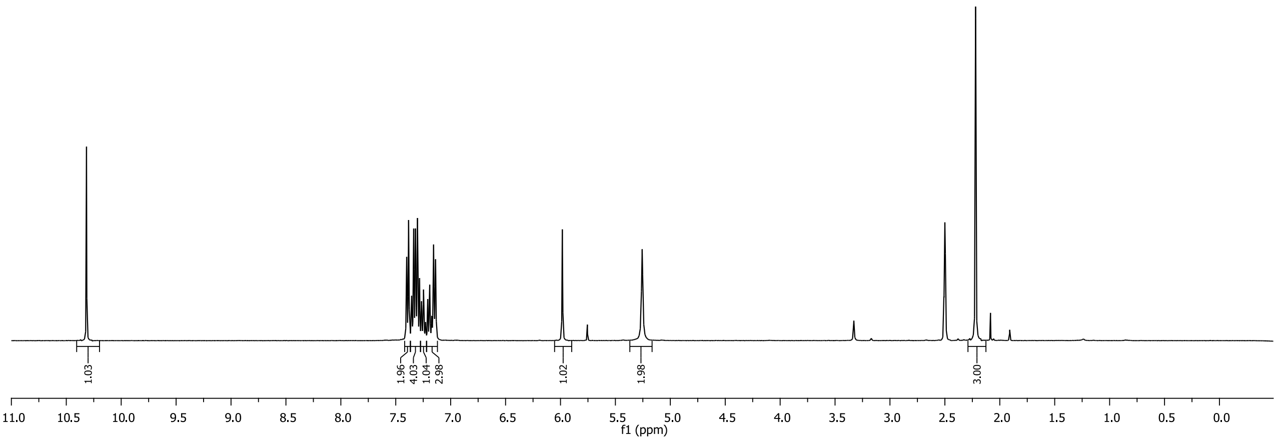

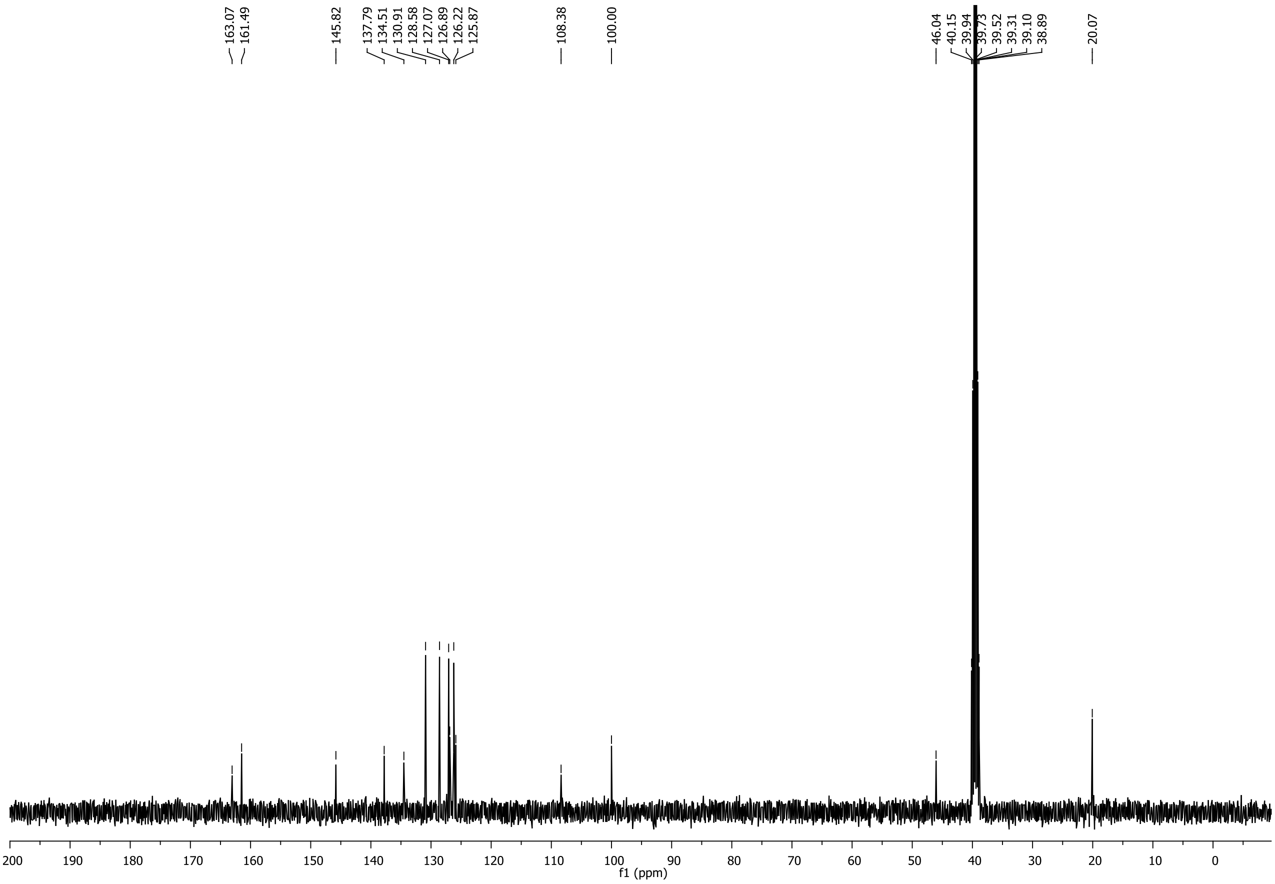

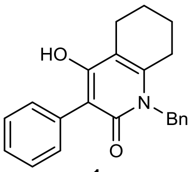
HO
N
Bn
O

1q

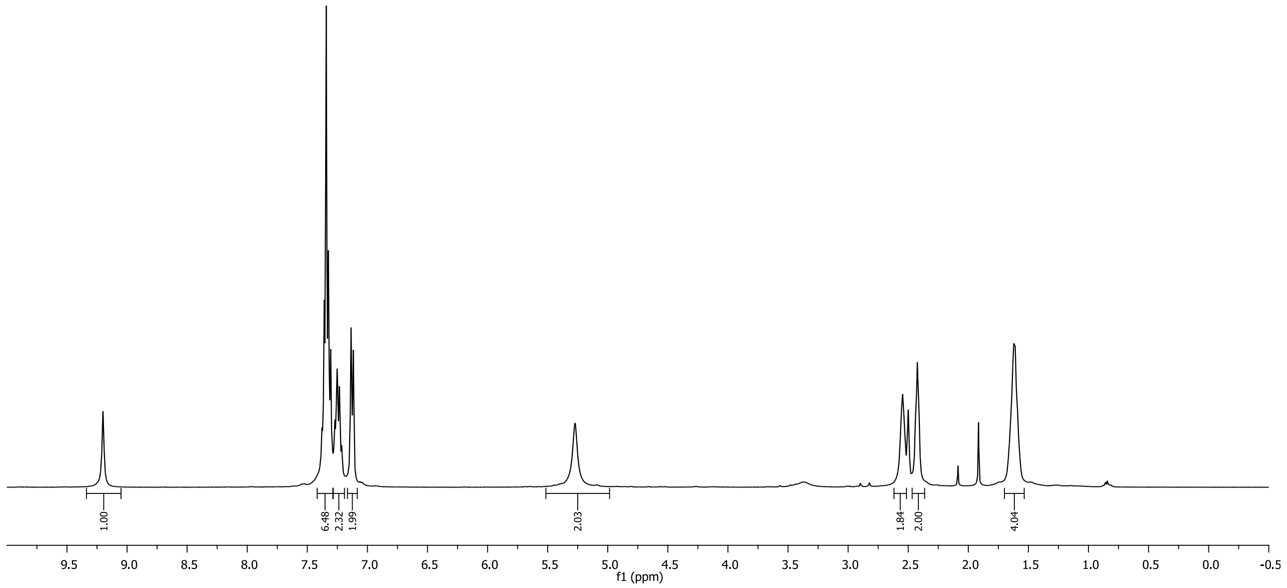
1.00
6.48
2.32
1.99
2.03
1.84
2.00
4.04
9.5 9.0 8.5 8.0 7.5 7.0 6.5 6.0 5.5 5.0 4.5 4.0 3.5 3.0 2.5 2.0 1.5 1.0 0.5 0.0 -0.5
f1 (ppm)

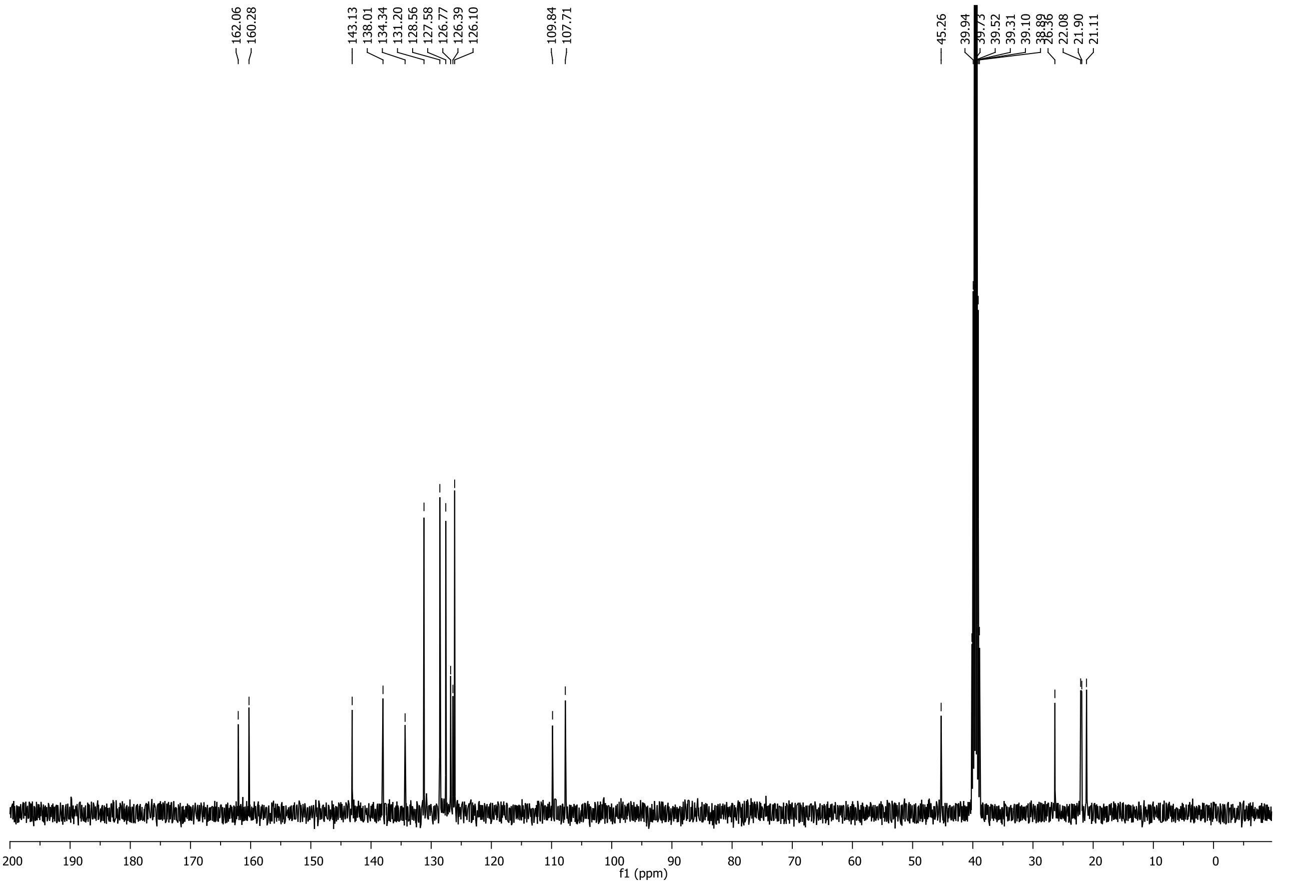
162.06
160.28
143.13
138.01
134.34
131.20
128.56
127.58
126.77
126.39
126.10
109.84
107.71
45.26
39.94
39.73
39.52
39.31
39.10
38.89
26.36
22.08
21.90
21.11
200 190 180 170 160 150 140 130 120 110 100 90 80 70 60 50 40 30 20 10 0
f1 (ppm)

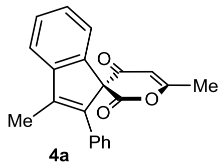
O
Me
Me
O
O
Ph

4a

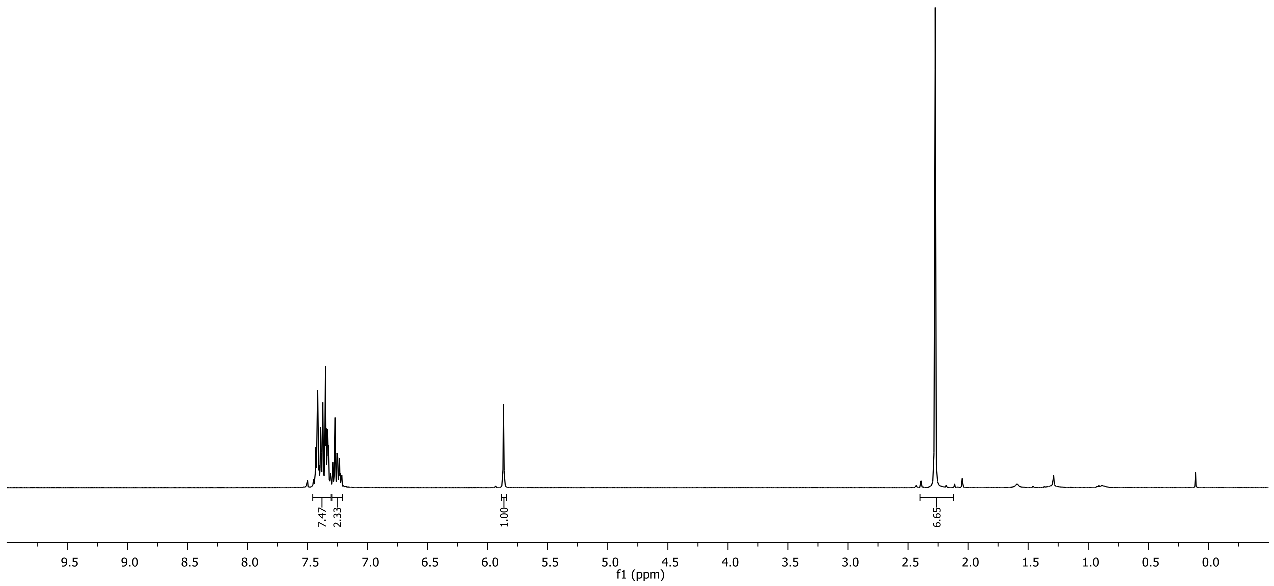
7.47
2.33
1.00
6.65
9.5 9.0 8.5 8.0 7.5 7.0 6.5 6.0 5.5 5.0 4.5 4.0 3.5 3.0 2.5 2.0 1.5 1.0 0.5 0.0
f1 (ppm)

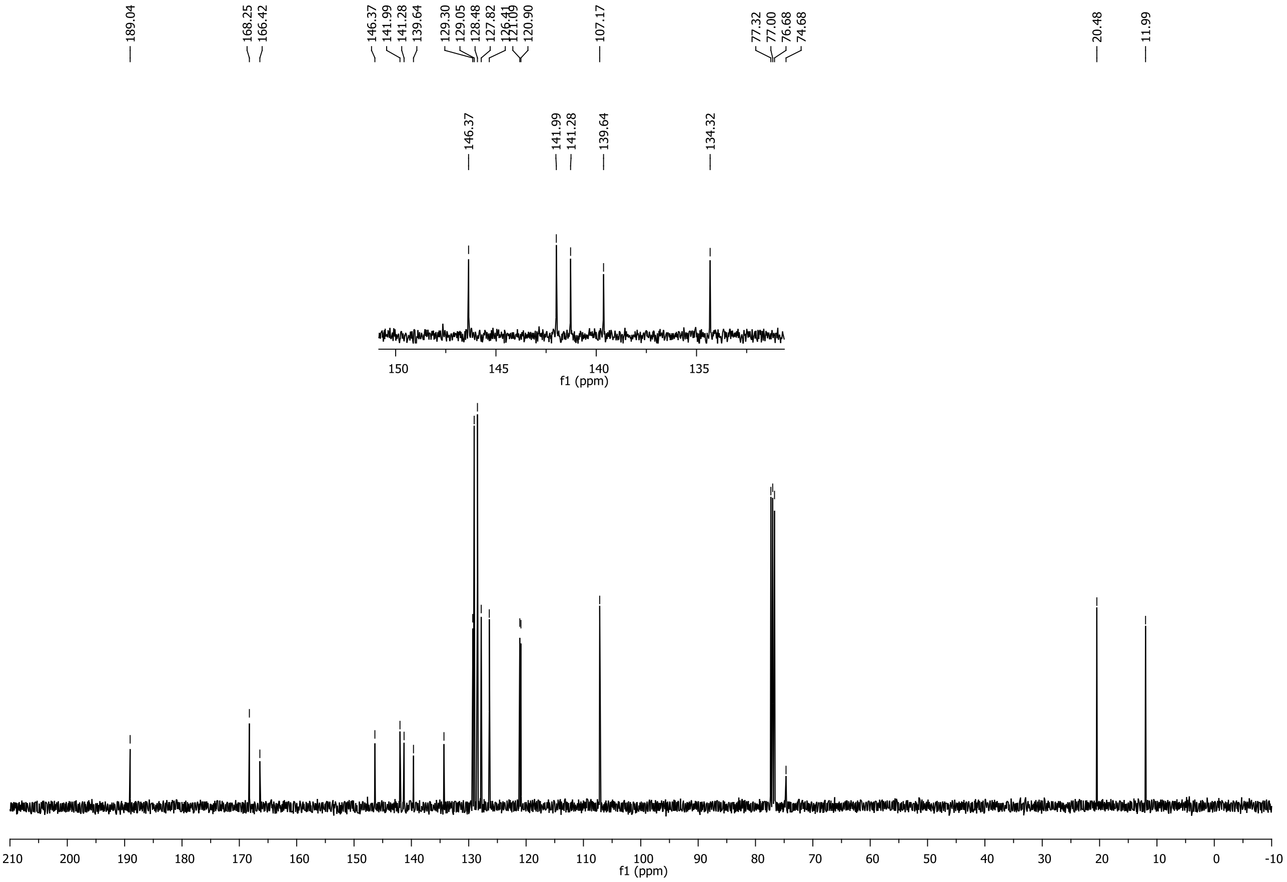
189.04
168.25
166.42
146.37
141.99
141.28
139.64
129.30
129.05
128.48
127.82
126.41
121.09
120.90
107.17
77.32
77.00
76.68
74.68
20.48
11.99
146.37
141.99
141.28
139.64
134.32
150 145 140 135
f1 (ppm)
210 200 190 180 170 160 150 140 130 120 110 100 90 80 70 60 50 40 30 20 10 0 -10
f1 (ppm)

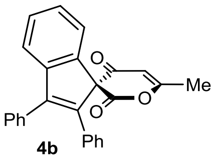
Me
Ph
Ph
O
O
O

4b

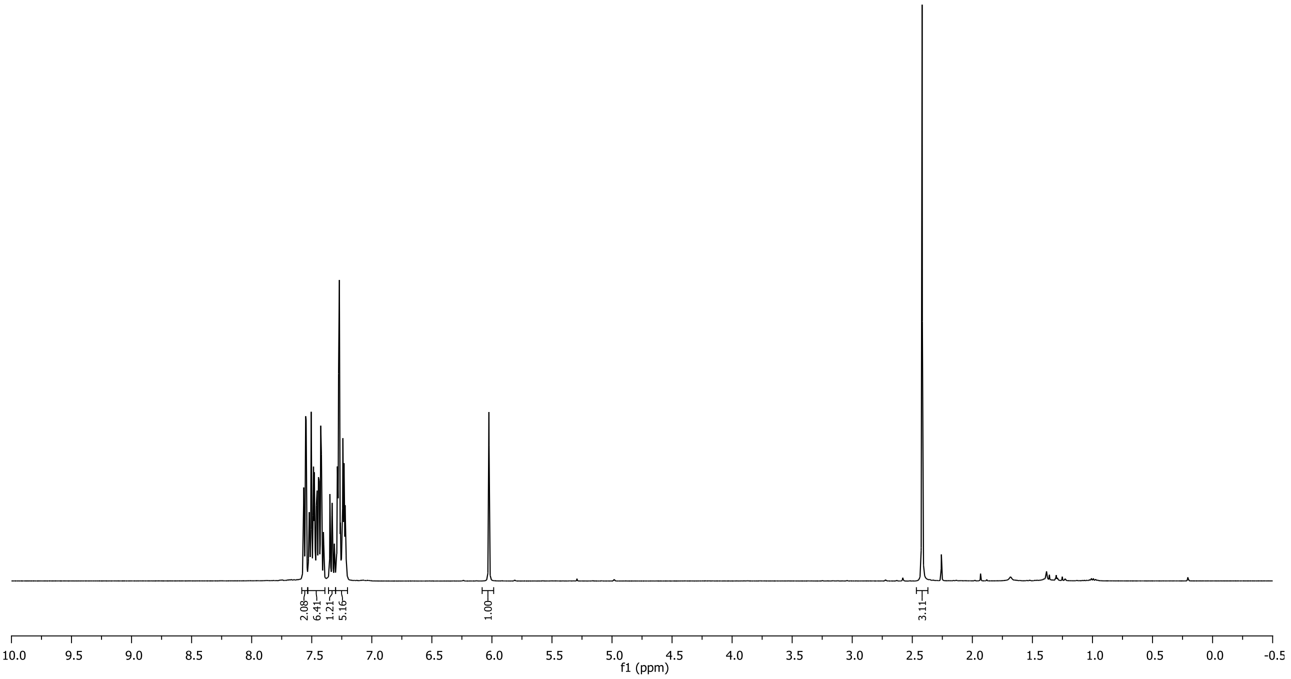
2.08
6.41
1.21
5.16
1.00
3.11
10.0 9.5 9.0 8.5 8.0 7.5 7.0 6.5 6.0 5.5 5.0 4.5 4.0 3.5 3.0 2.5 2.0 1.5 1.0 0.5 0.0 -0.5
f1 (ppm)

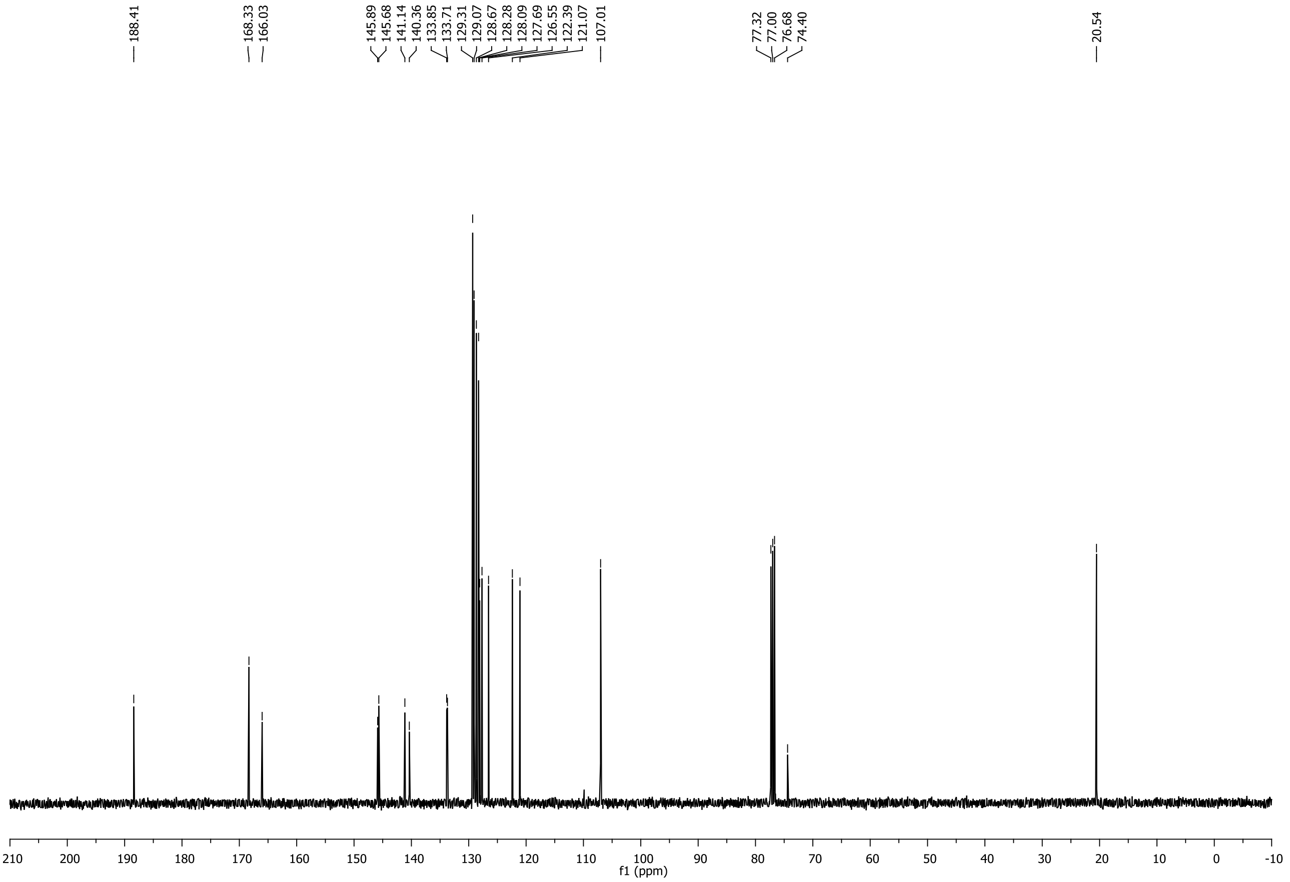
188.41
168.33
166.03
145.89
145.68
141.14
140.36
133.85
133.71
129.31
129.07
128.67
128.28
128.09
127.69
126.55
122.39
121.07
107.01
77.32
77.00
76.68
74.40
20.54
210 200 190 180 170 160 150 140 130 120 110 100 90 80 70 60 50 40 30 20 10 0 -10
f1 (ppm)

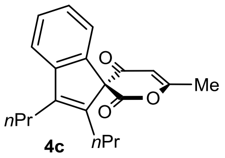
O
Me
O
O
nPr
nPr

4c

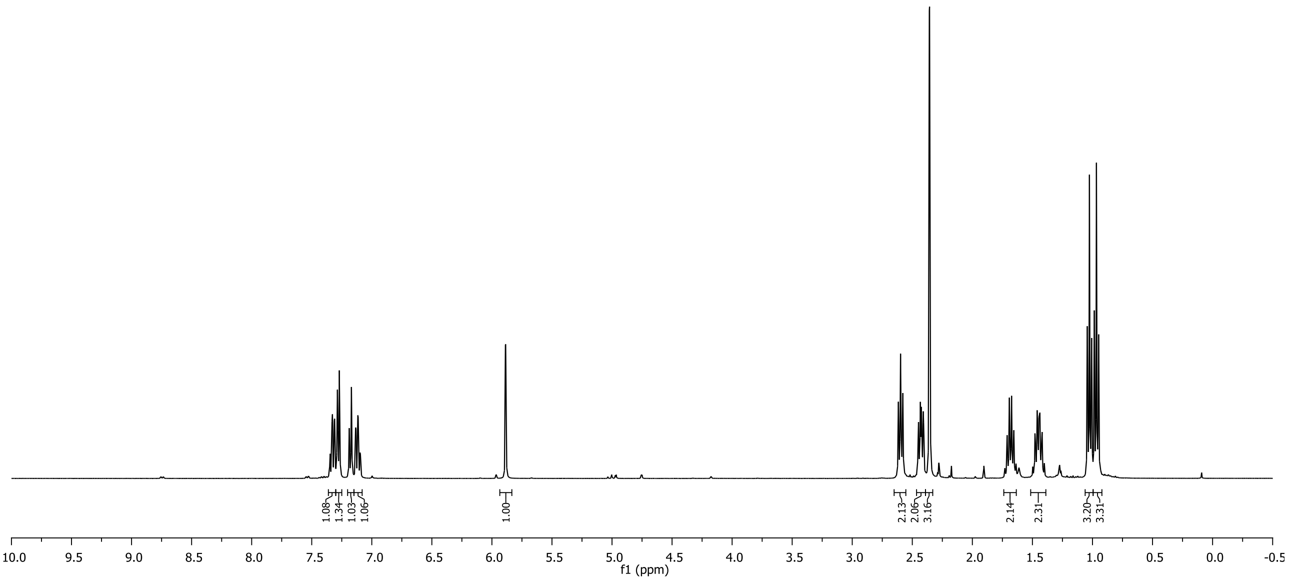
1.08
1.34
1.03
1.06
1.00
2.13
2.06
3.16
2.14
2.31
3.20
3.31
10.0 9.5 9.0 8.5 8.0 7.5 7.0 6.5 6.0 5.5 5.0 4.5 4.0 3.5 3.0 2.5 2.0 1.5 1.0 0.5 0.0 -0.5
f1 (ppm)

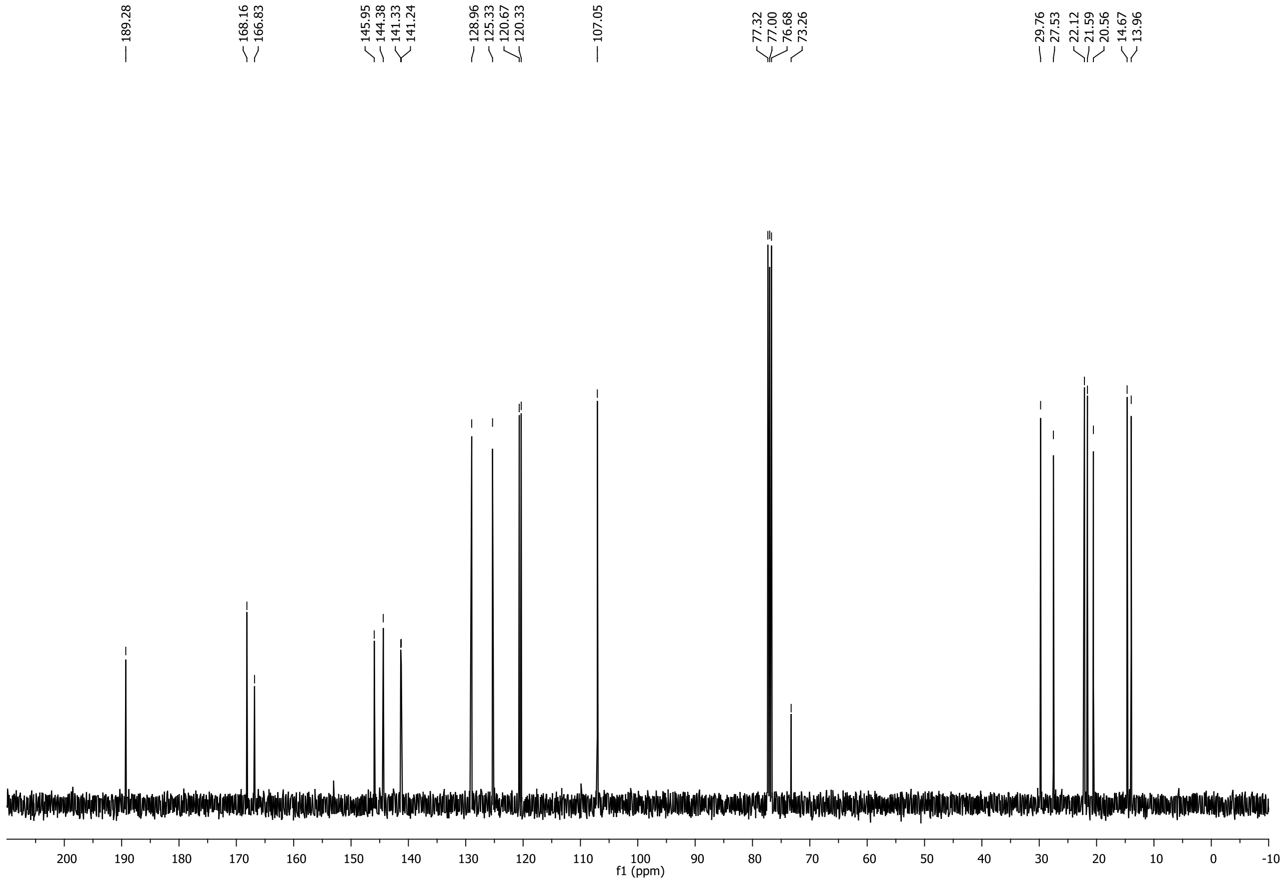
189.28
168.16
166.83
145.95
144.38
141.33
141.24
128.96
125.33
120.67
120.33
107.05
77.32
77.00
76.68
73.26
29.76
27.53
22.12
21.59
20.56
14.67
13.96
200 190 180 170 160 150 140 130 120 110 100 90 80 70 60 50 40 30 20 10 0 -10
f1 (ppm)

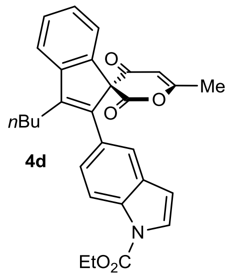
O
Me
O
O
nBu
N
EtO2C

4d

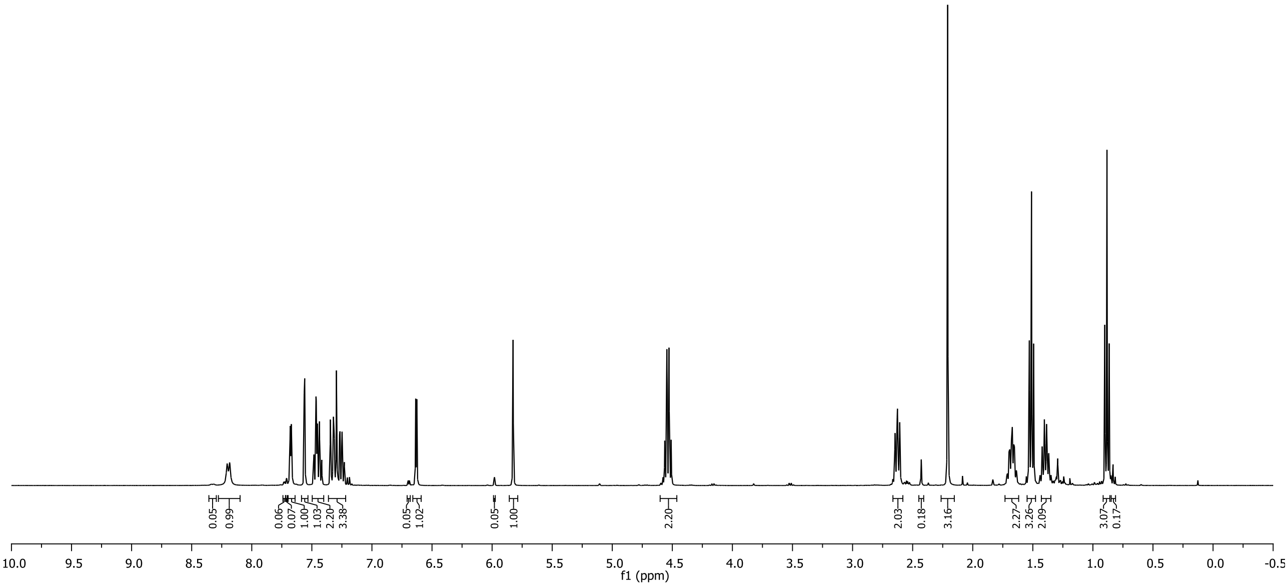
0.05
0.99
0.06
0.07
1.00
1.03
2.20
3.38
0.05
1.02
0.05
1.00
2.20
2.03
0.18
3.16
2.27
3.26
2.09
3.07
0.17
f1 (ppm)

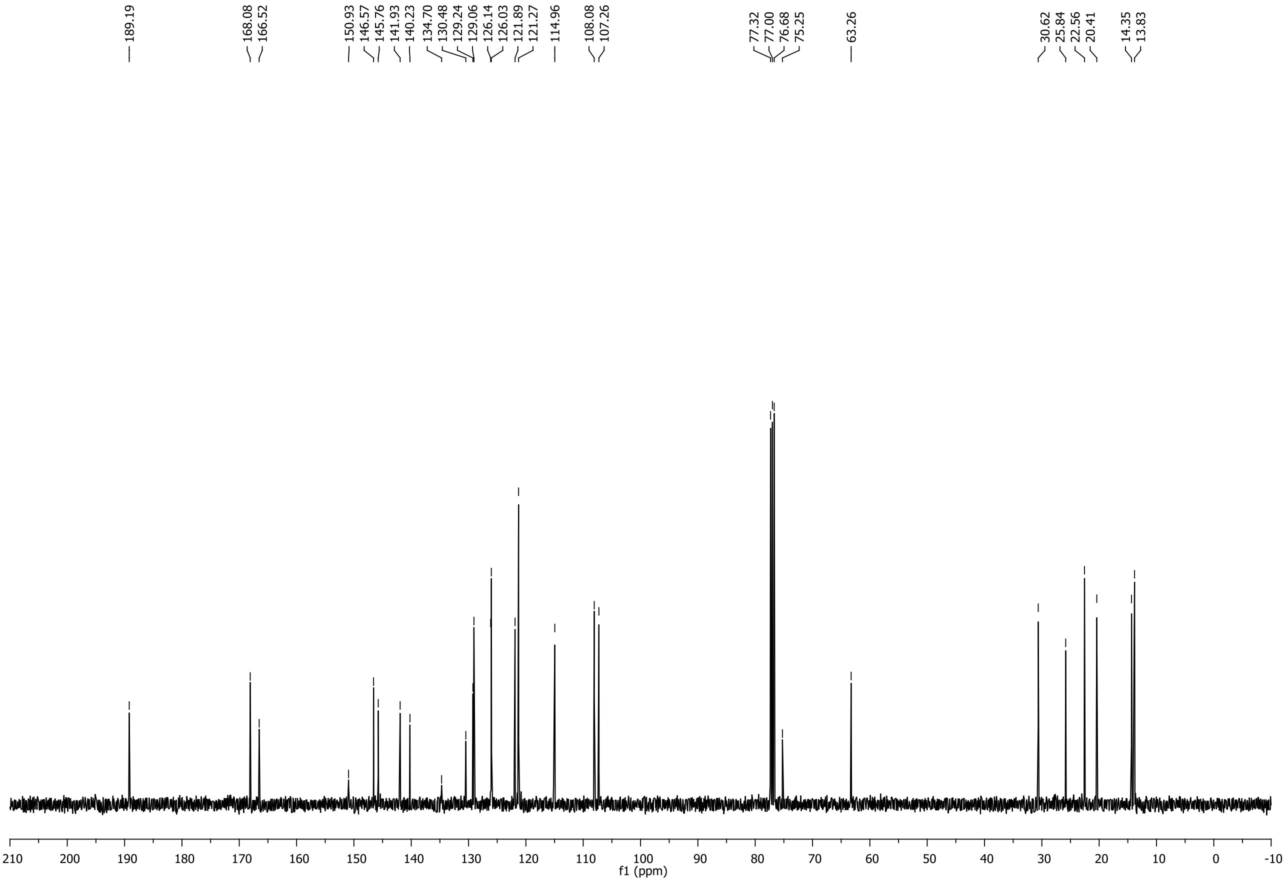
189.19
168.08
166.52
150.93
146.57
145.76
141.93
140.23
134.70
130.48
129.24
129.06
126.14
126.03
121.89
121.27
114.96
108.08
107.26
77.32
77.00
76.68
75.25
63.26
30.62
25.84
22.56
20.41
14.35
13.83
f1 (ppm)

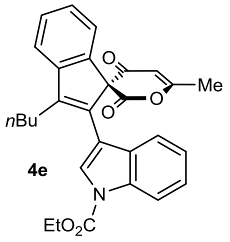
O
Me
nBu
O
O
N
EtO2C

4e

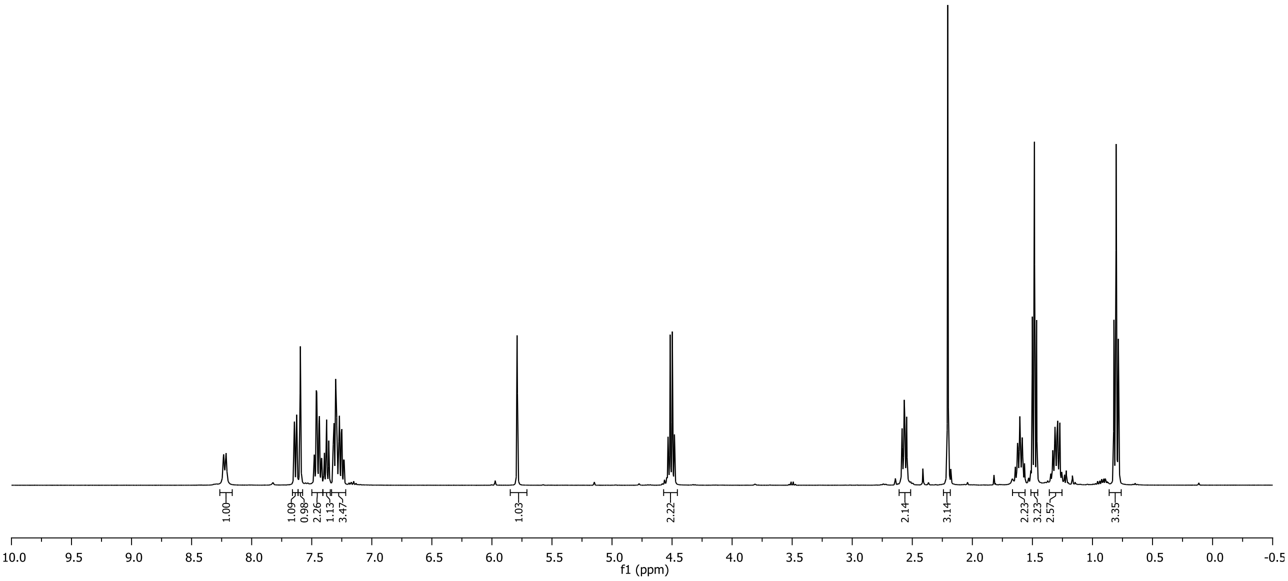
1.00
1.09
0.98
2.26
1.13
3.47
1.03
2.22
2.14
3.14
2.23
3.23
2.57
3.35
10.0 9.5 9.0 8.5 8.0 7.5 7.0 6.5 6.0 5.5 5.0 4.5 4.0 3.5 3.0 2.5 2.0 1.5 1.0 0.5 0.0 -0.5
f1 (ppm)

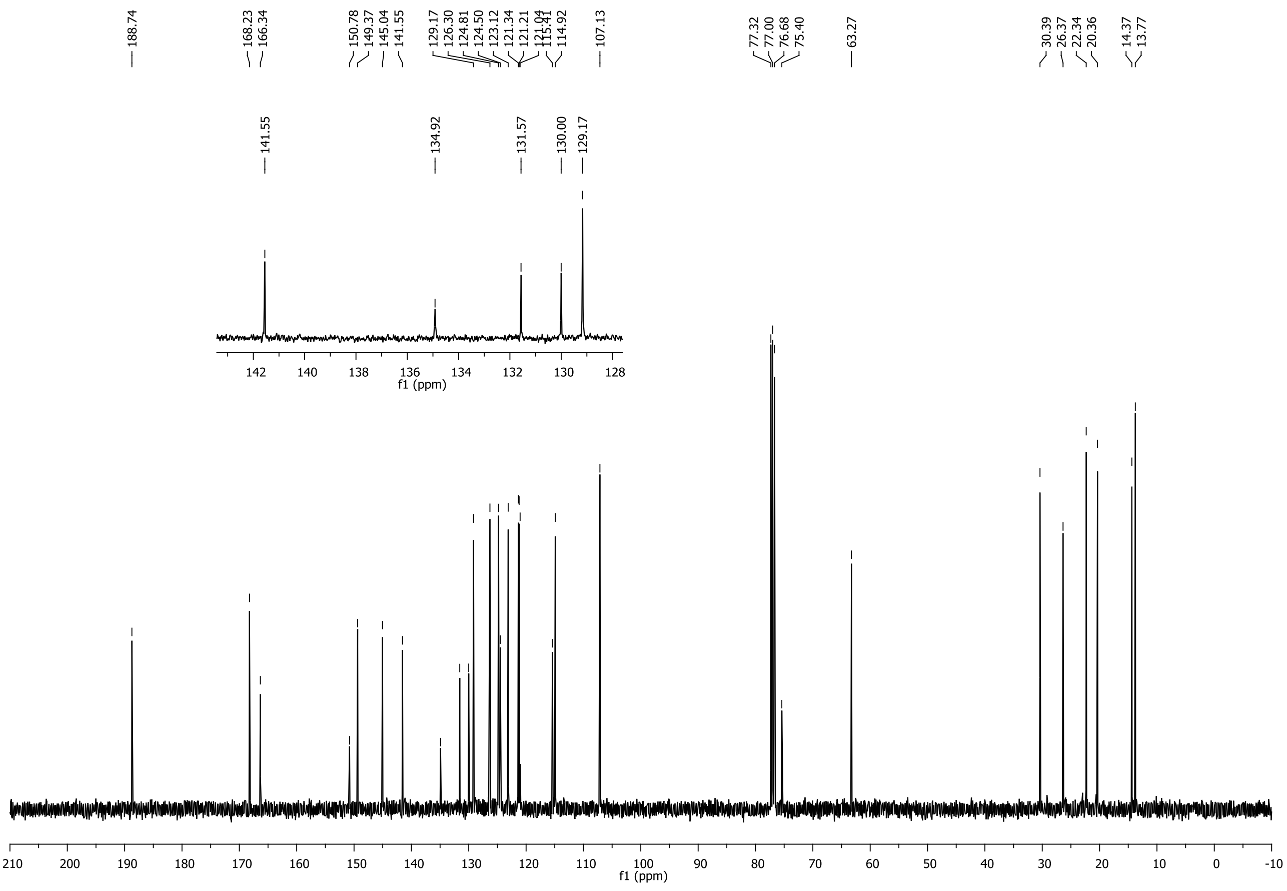
188.74
168.23
166.34
150.78
149.37
145.04
141.55
129.17
126.30
124.81
124.50
123.12
121.34
121.21
121.04
115.41
114.92
107.13
77.32
77.00
76.68
75.40
63.27
30.39
26.37
22.34
20.36
14.37
13.77
141.55
134.92
131.57
130.00
129.17
142 140 138 136 134 132 130 128
f1 (ppm)
210 200 190 180 170 160 150 140 130 120 110 100 90 80 70 60 50 40 30 20 10 0 -10
f1 (ppm)

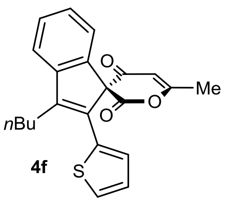
O
Me
O
O
nBu
S

4f

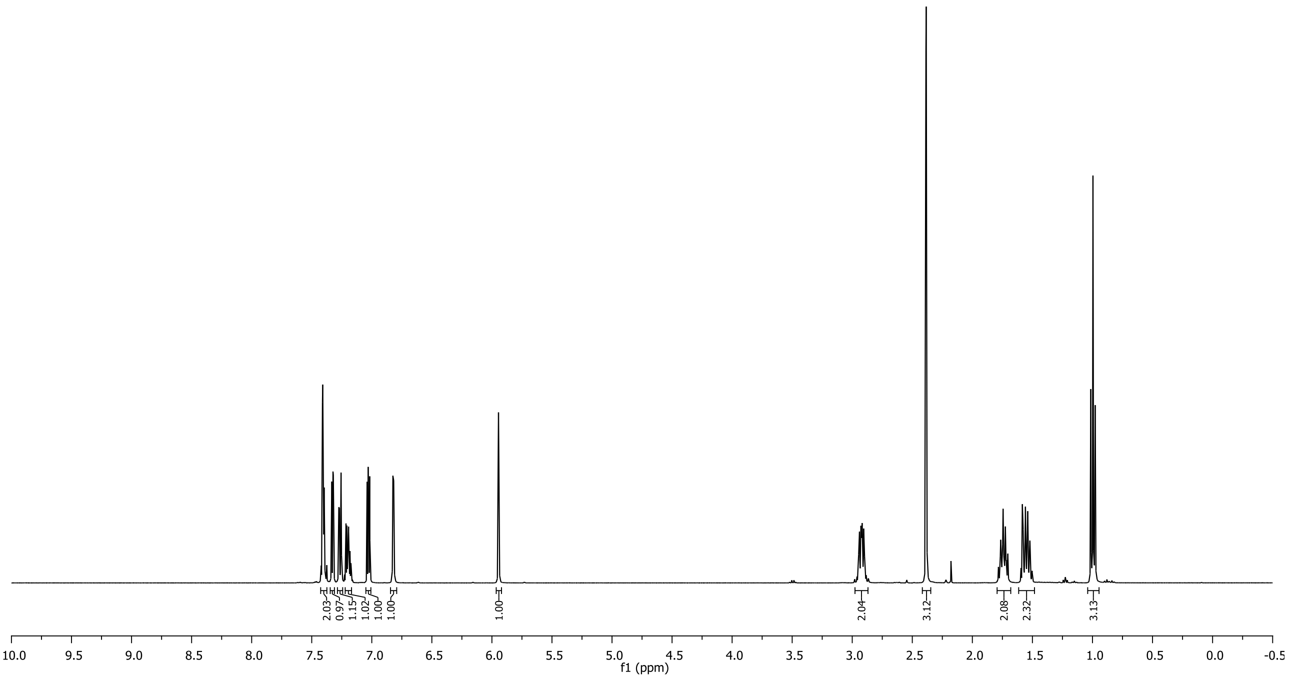
2.03
0.97
1.15
1.02
1.00
1.00
1.00
2.04
3.12
2.08
2.32
3.13
10.0 9.5 9.0 8.5 8.0 7.5 7.0 6.5 6.0 5.5 5.0 4.5 4.0 3.5 3.0 2.5 2.0 1.5 1.0 0.5 0.0 -0.5
f1 (ppm)

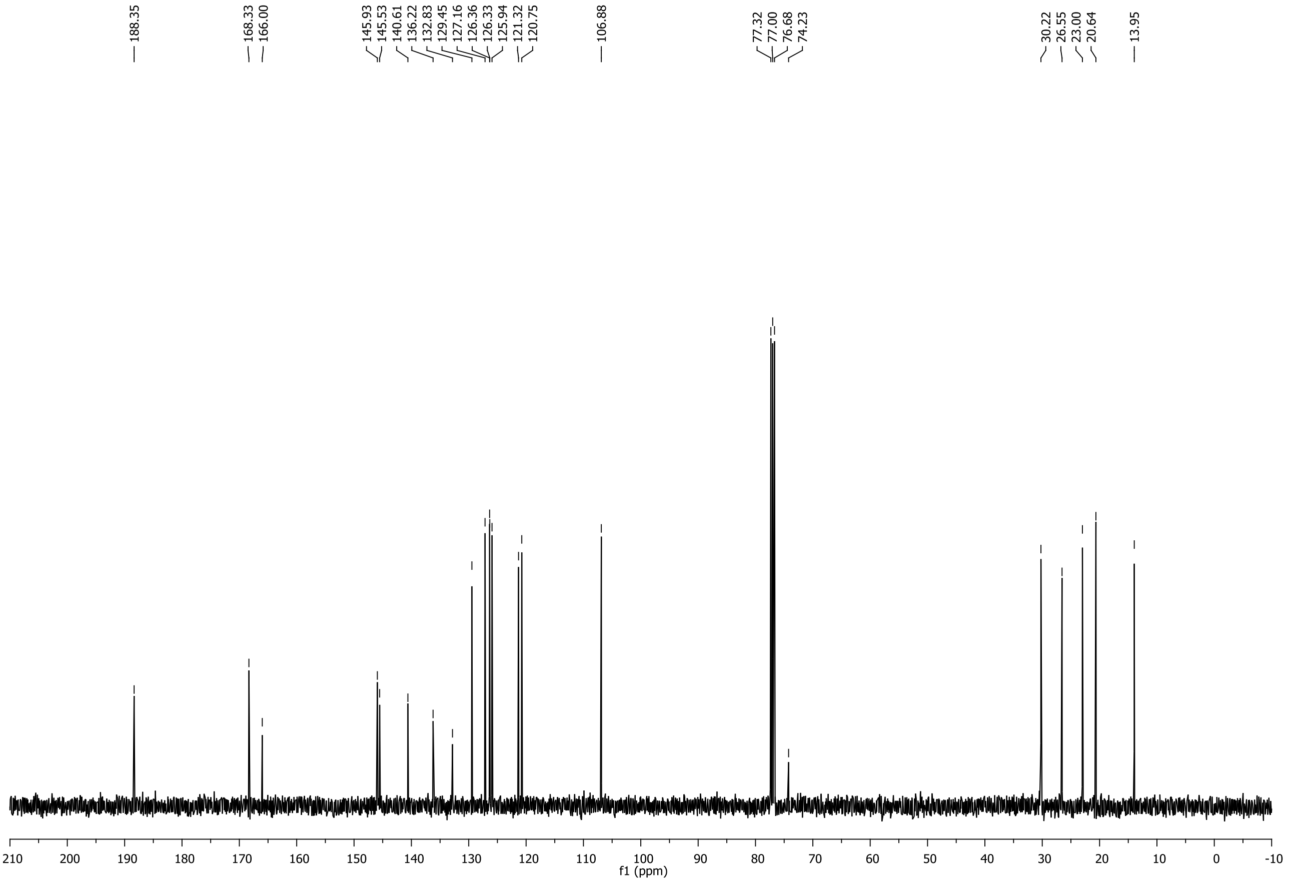
188.35
168.33
166.00
145.93
145.53
140.61
136.22
132.83
129.45
127.16
126.36
126.33
125.94
121.32
120.75
106.88
77.32
77.00
76.68
74.23
30.22
26.55
23.00
20.64
13.95
210 200 190 180 170 160 150 140 130 120 110 100 90 80 70 60 50 40 30 20 10 0 -10
f1 (ppm)

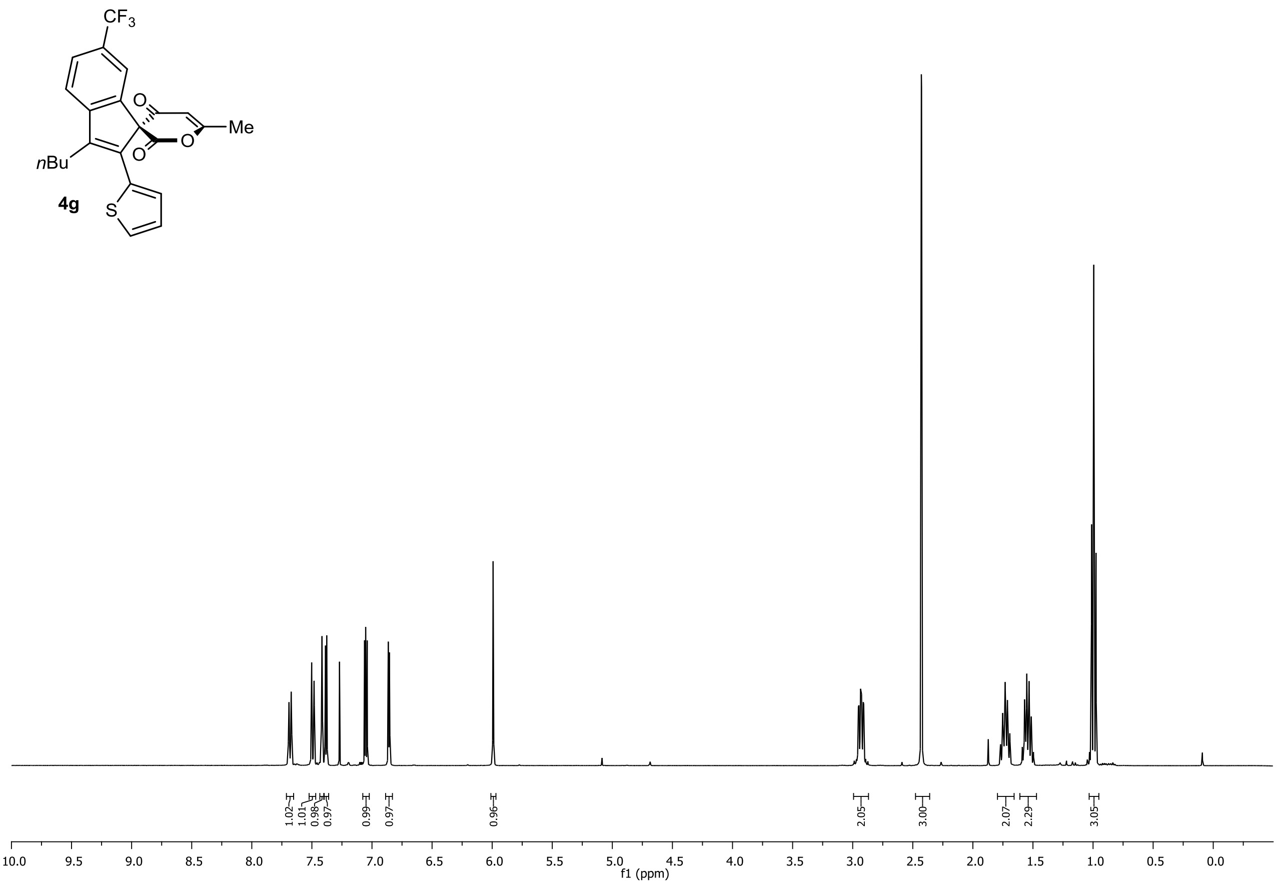
CF3
O
O
O
Me
nBu
4g
S
f1 (ppm)

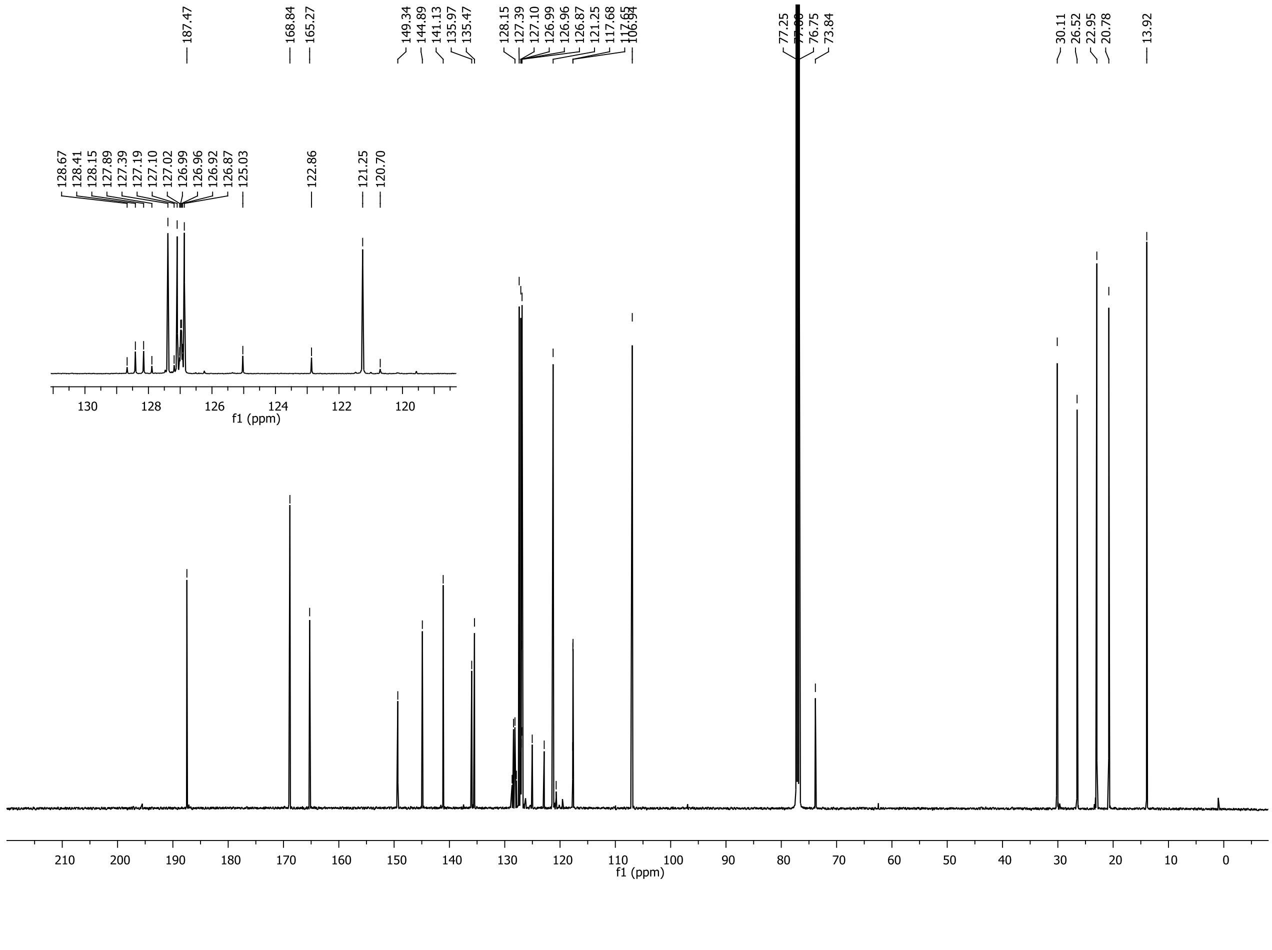
f1 (ppm)

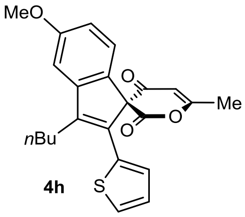

4h

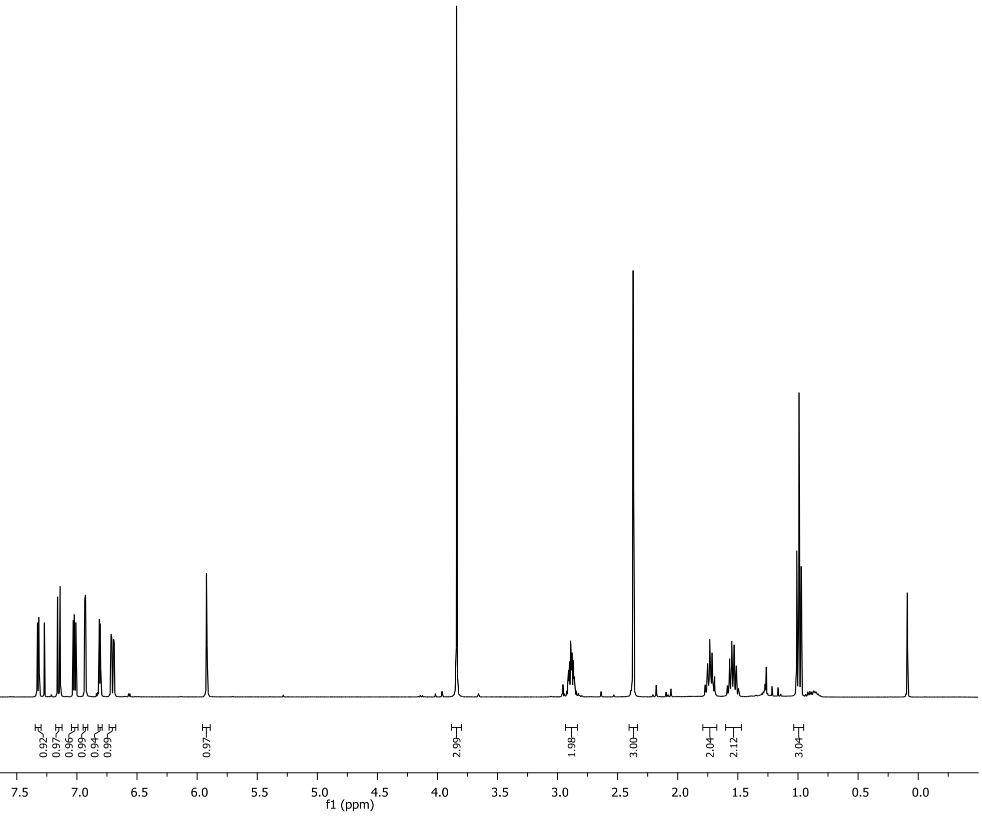

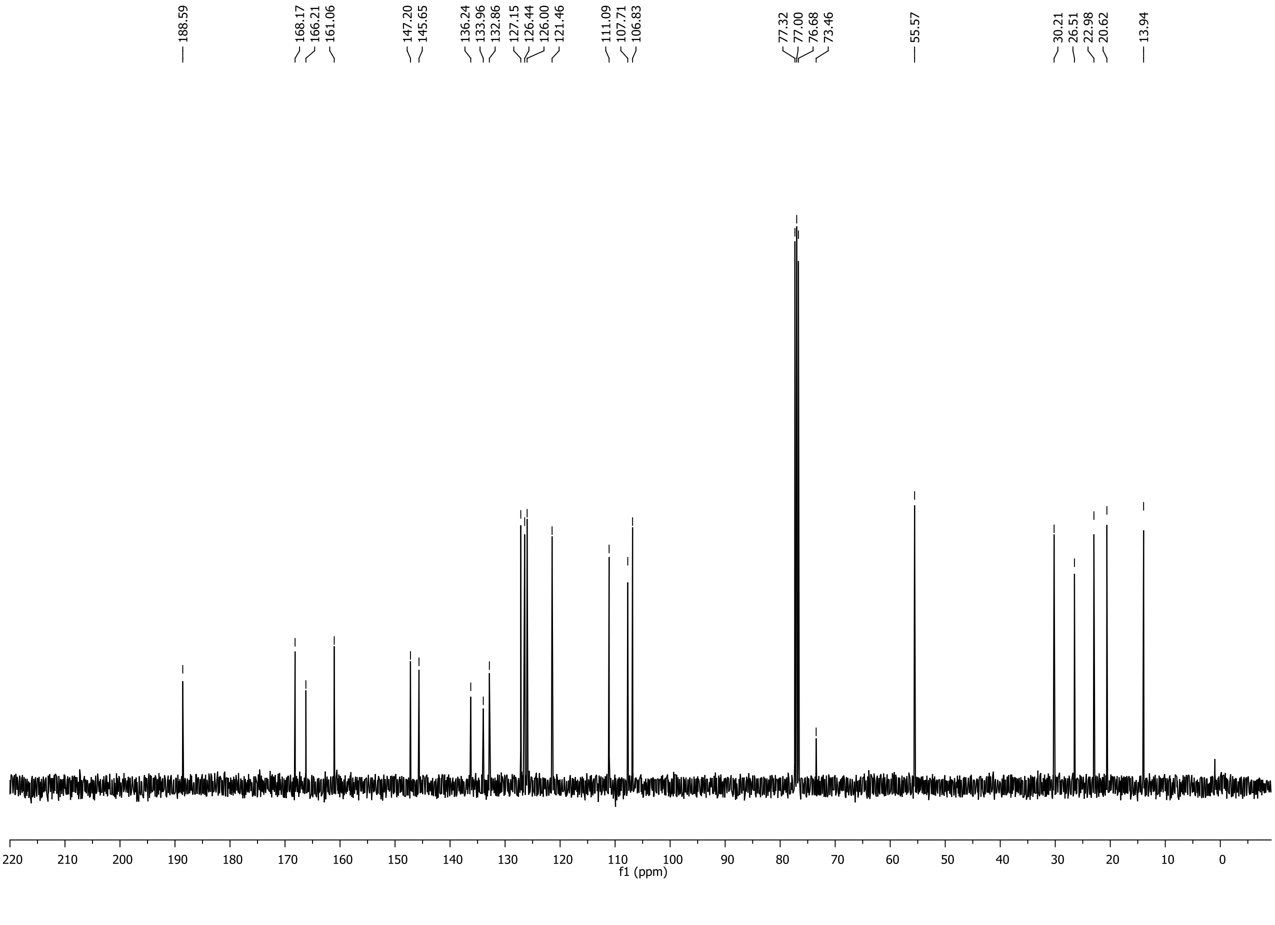

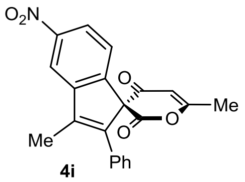
O2N
O
O
O
Me
Me
Ph

4i

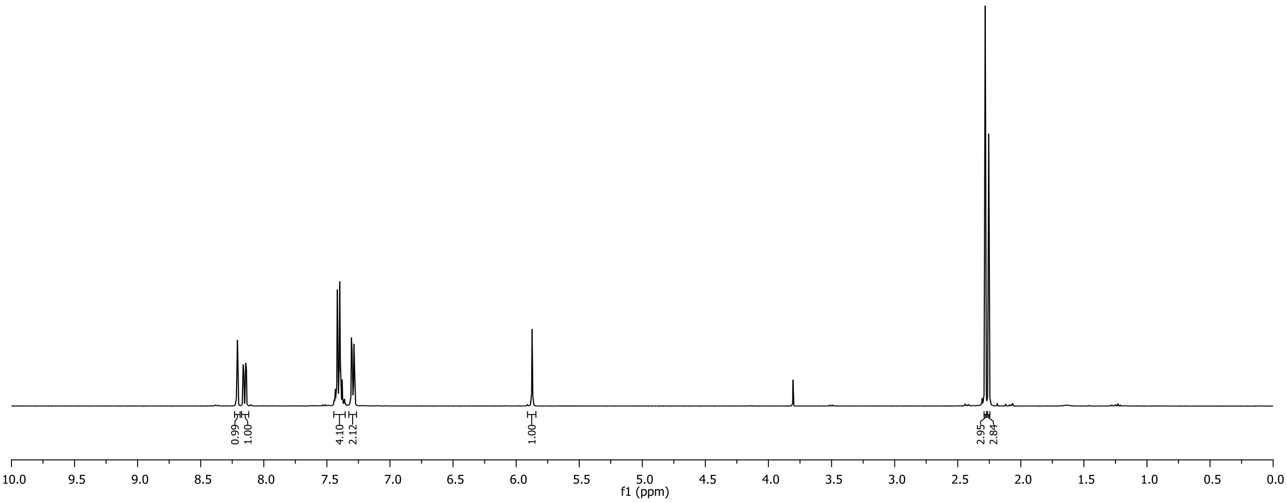
0.99
1.00
4.10
2.12
1.00
2.95
2.84
f1 (ppm)

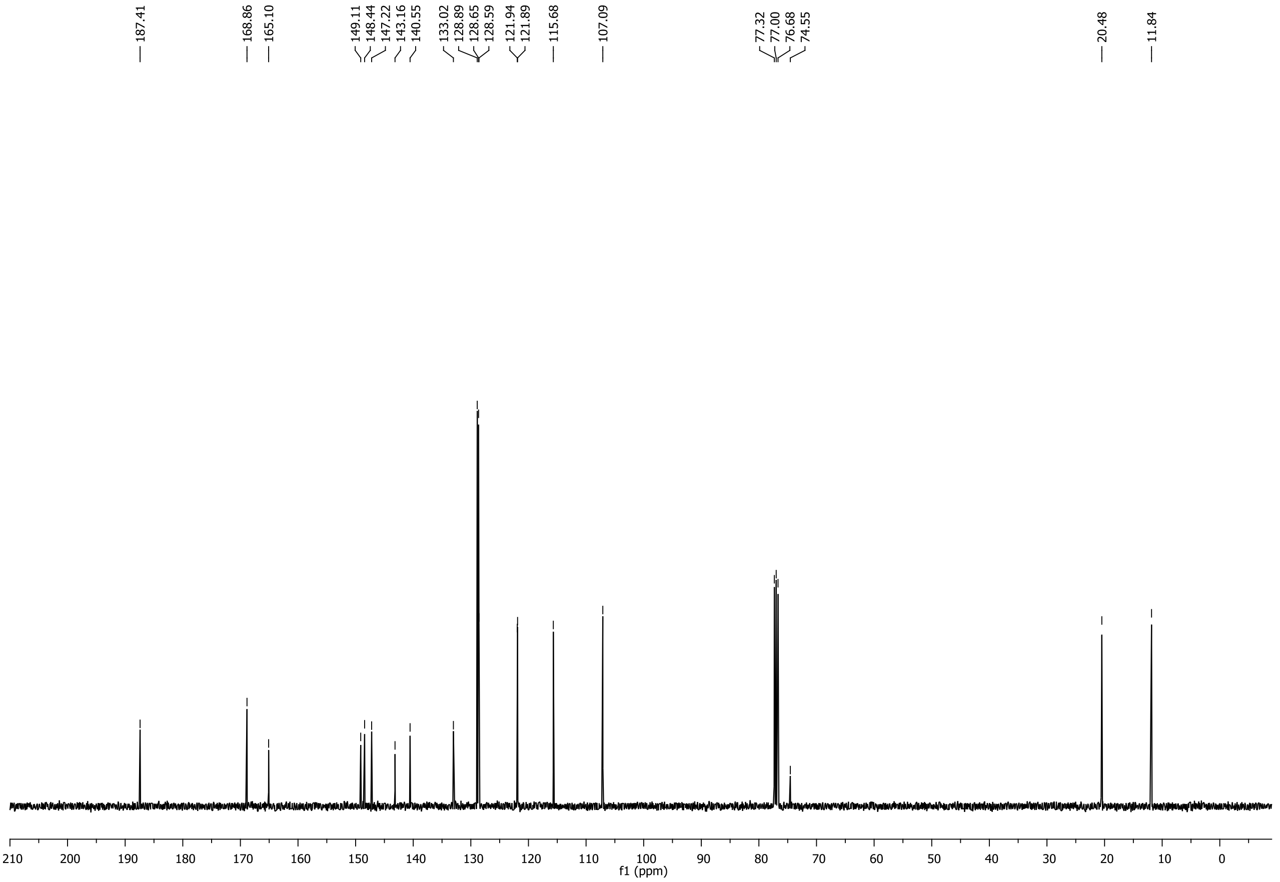
187.41
168.86
165.10
149.11
148.44
147.22
143.16
140.55
133.02
128.89
128.65
128.59
121.94
121.89
115.68
107.09
77.32
77.00
76.68
74.55
20.48
11.84
f1 (ppm)

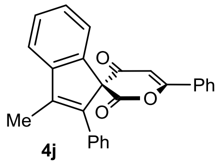
O
O
O
Ph
Me
Ph

4j

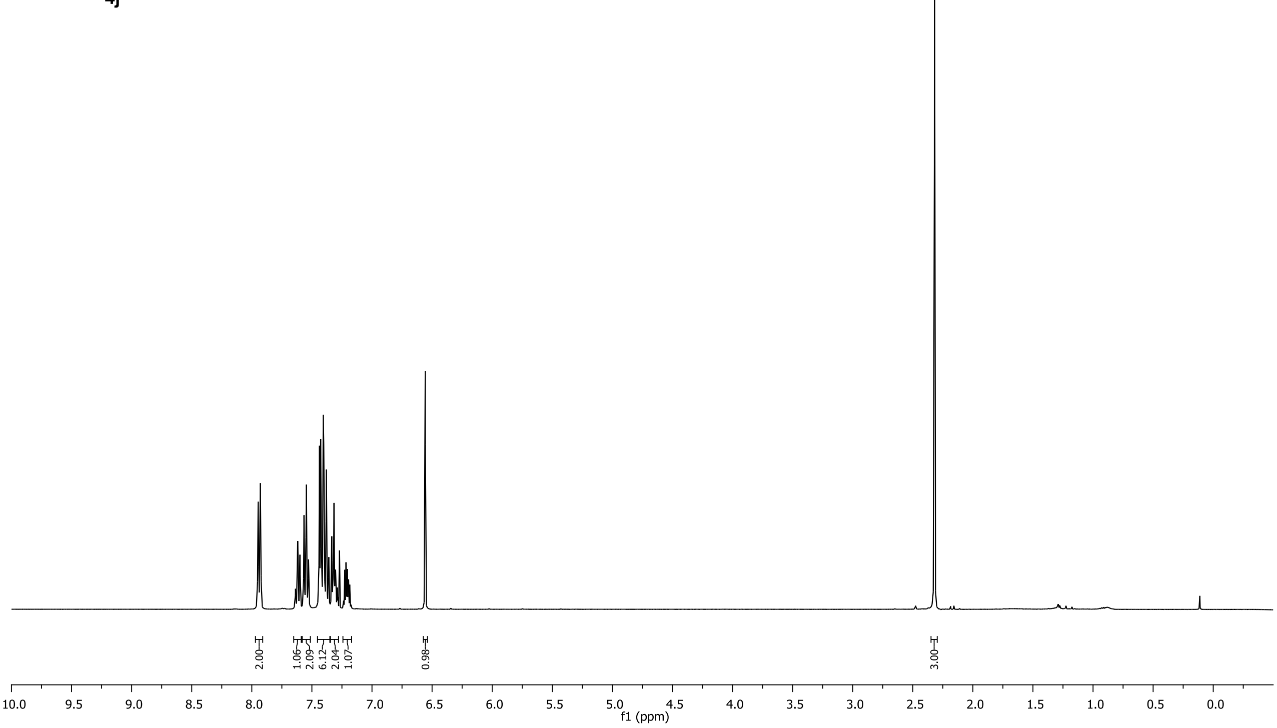
2.00
1.06
2.09
6.12
2.04
1.07
0.98
3.00
10.0 9.5 9.0 8.5 8.0 7.5 7.0 6.5 6.0 5.5 5.0 4.5 4.0 3.5 3.0 2.5 2.0 1.5 1.0 0.5 0.0
f1 (ppm)

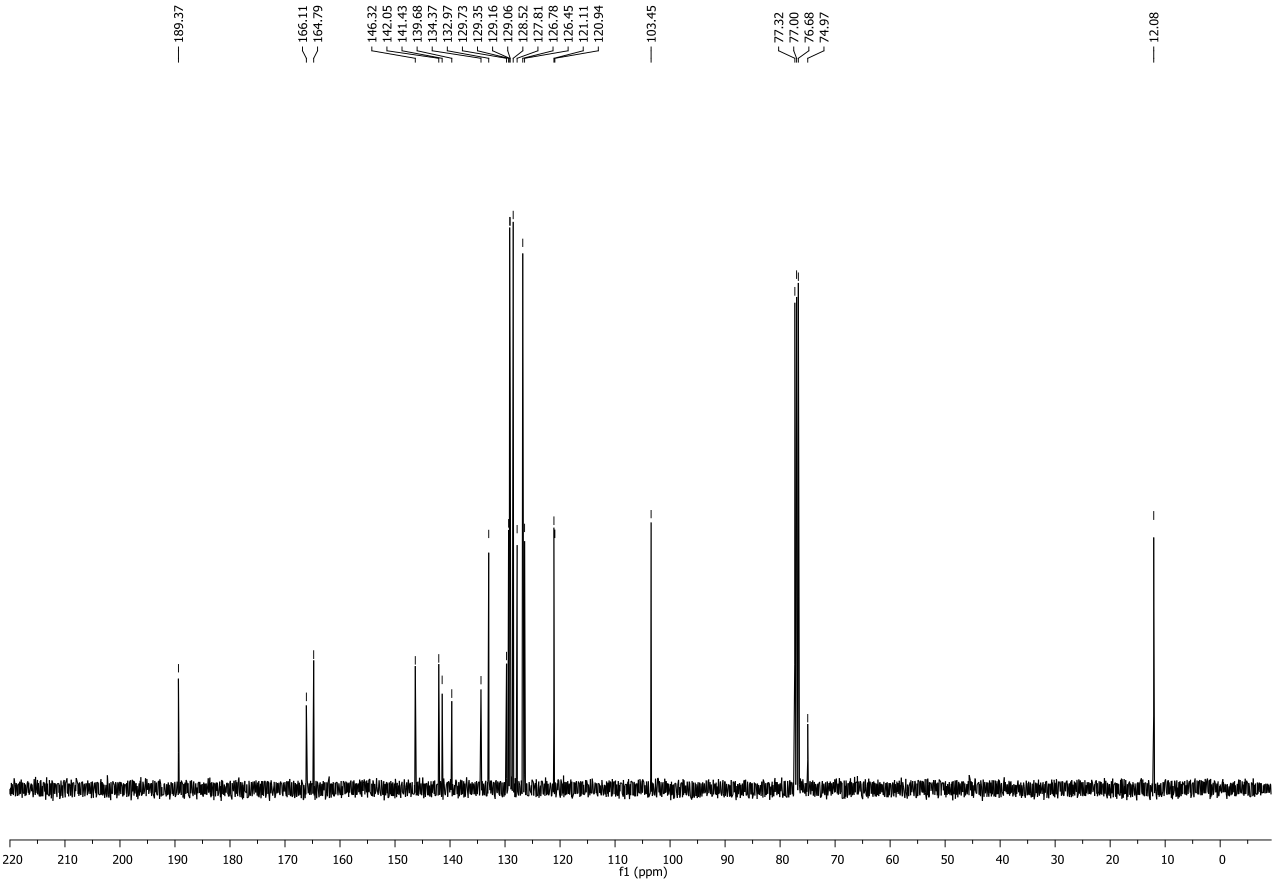
189.37
166.11
164.79
146.32
142.05
141.43
139.68
134.37
132.97
129.73
129.35
129.16
129.06
128.52
127.81
126.78
126.45
121.11
120.94
103.45
77.32
77.00
76.68
74.97
12.08
220 210 200 190 180 170 160 150 140 130 120 110 100 90 80 70 60 50 40 30 20 10 0
f1 (ppm)

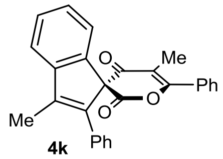
O
Me
Ph
O
O
Me
Ph

4k

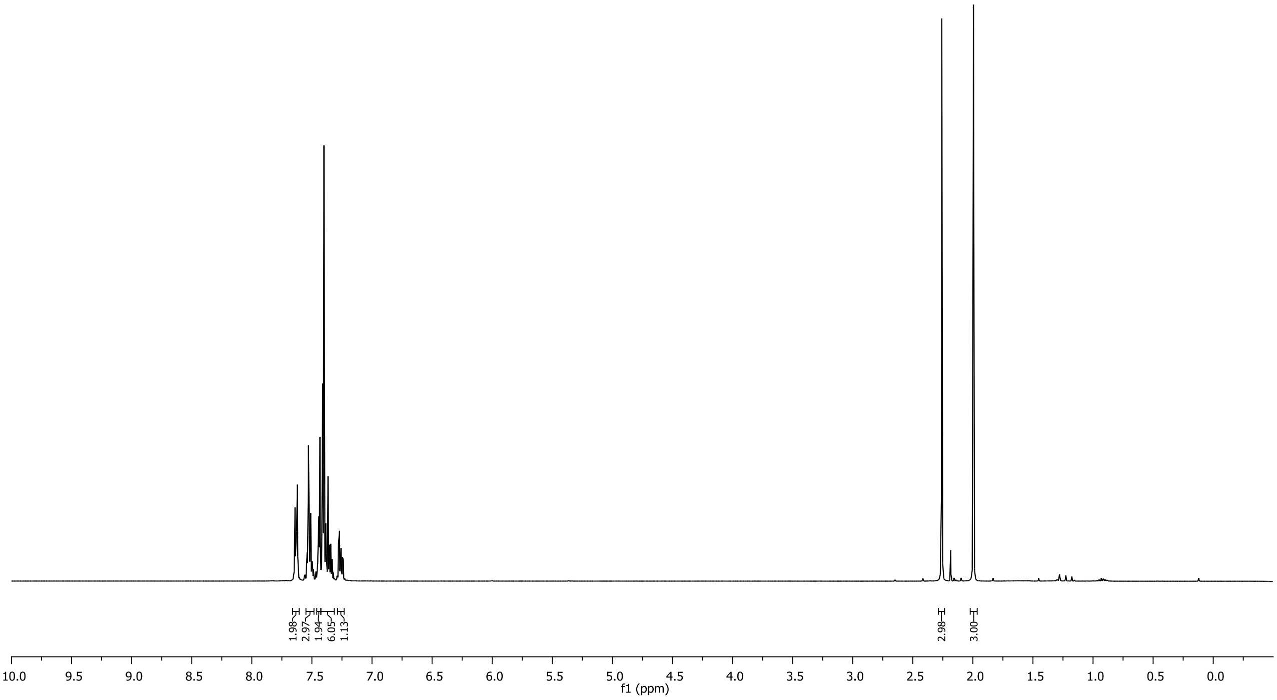
1.98
2.97
1.94
6.05
1.13
2.98
3.00
10.0 9.5 9.0 8.5 8.0 7.5 7.0 6.5 6.0 5.5 5.0 4.5 4.0 3.5 3.0 2.5 2.0 1.5 1.0 0.5 0.0
f1 (ppm)

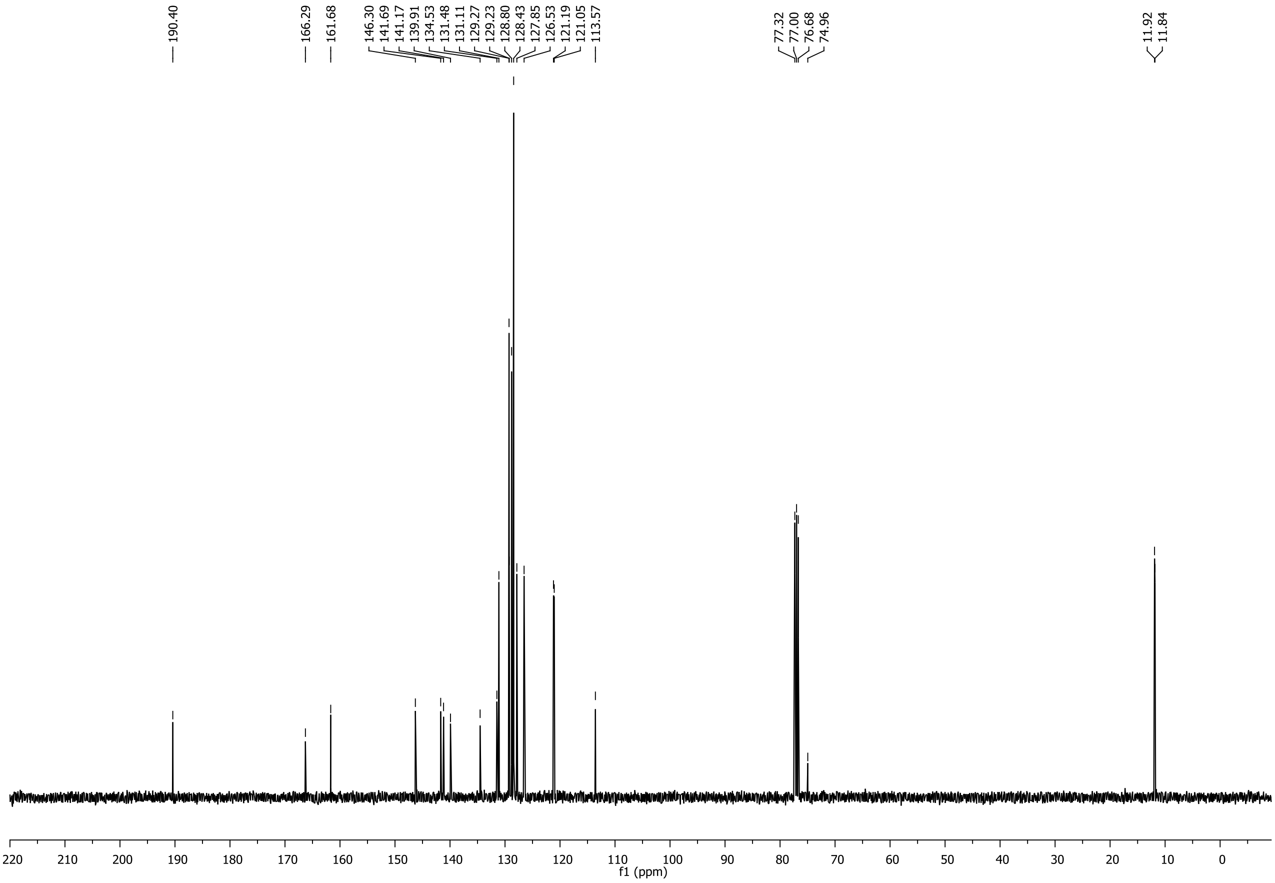
190.40
166.29
161.68
146.30
141.69
141.17
139.91
134.53
131.48
131.11
129.27
129.23
128.80
128.43
127.85
126.53
121.19
121.05
113.57
77.32
77.00
76.68
74.96
11.92
11.84
220 210 200 190 180 170 160 150 140 130 120 110 100 90 80 70 60 50 40 30 20 10 0
f1 (ppm)

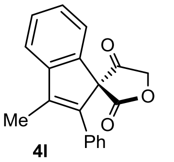
O
O
O
Me
Ph

4l

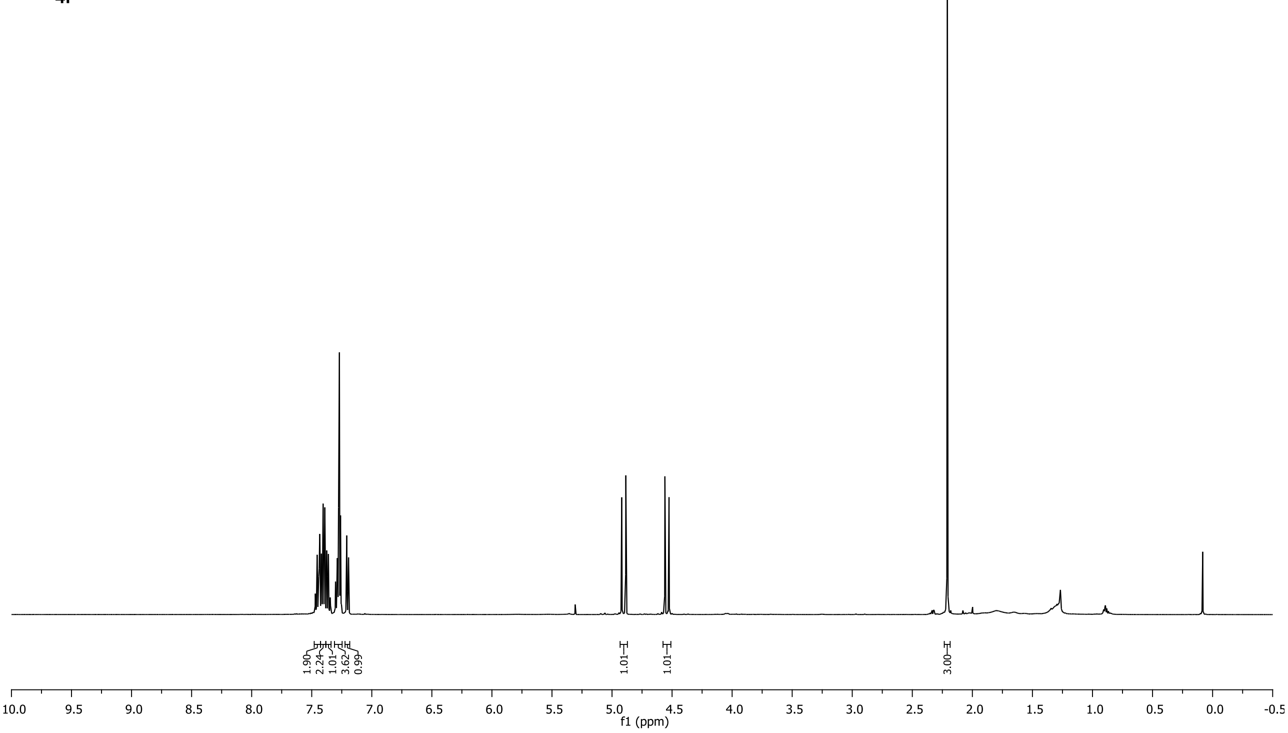
1.90
2.24
1.01
3.62
0.99
1.01
1.01
3.00
f1 (ppm)

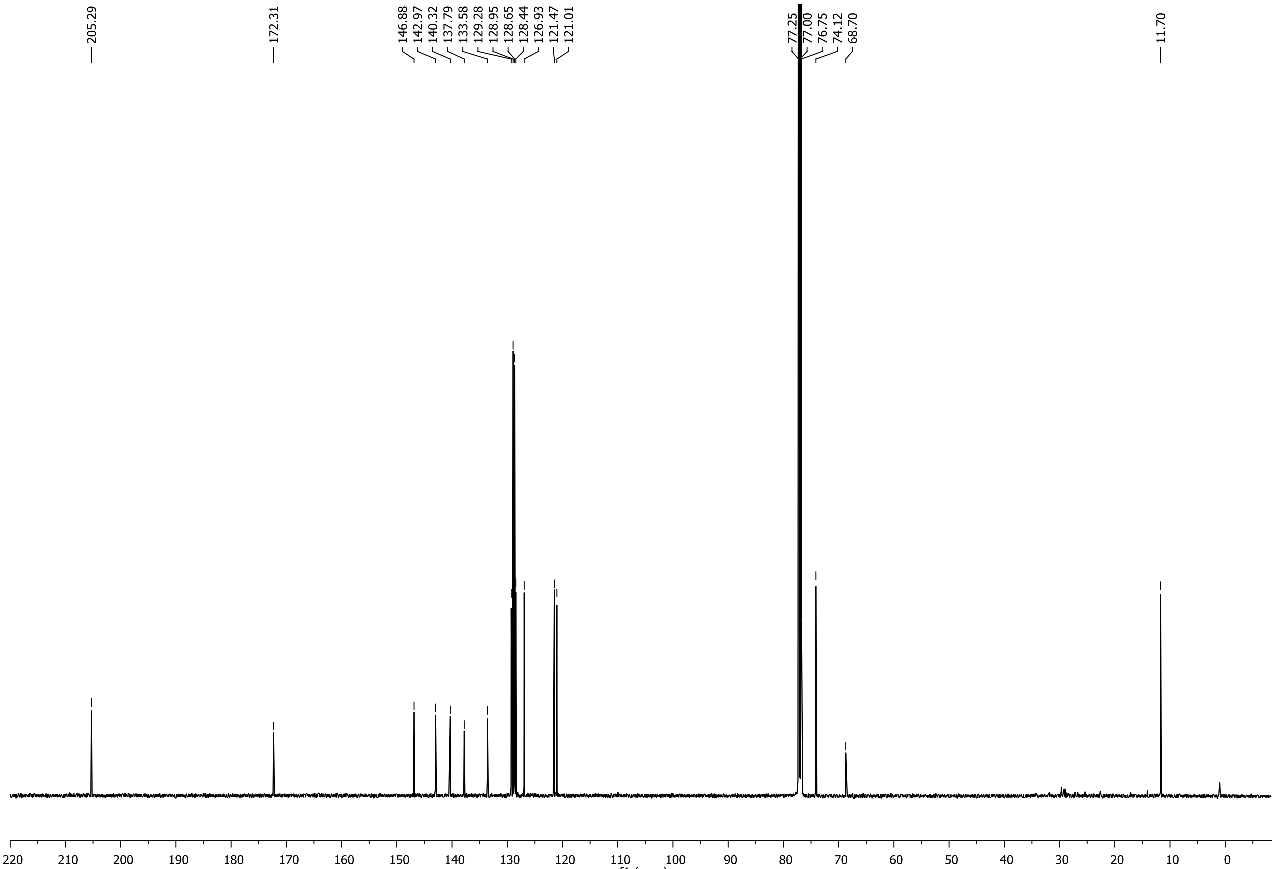
205.29
172.31
146.88
142.97
140.32
137.79
133.58
129.28
128.95
128.65
128.44
126.93
121.47
121.01
77.25
77.00
76.75
74.12
68.70
11.70
f1 (ppm)

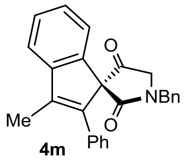
O
NBn
O
Me
Ph

4m

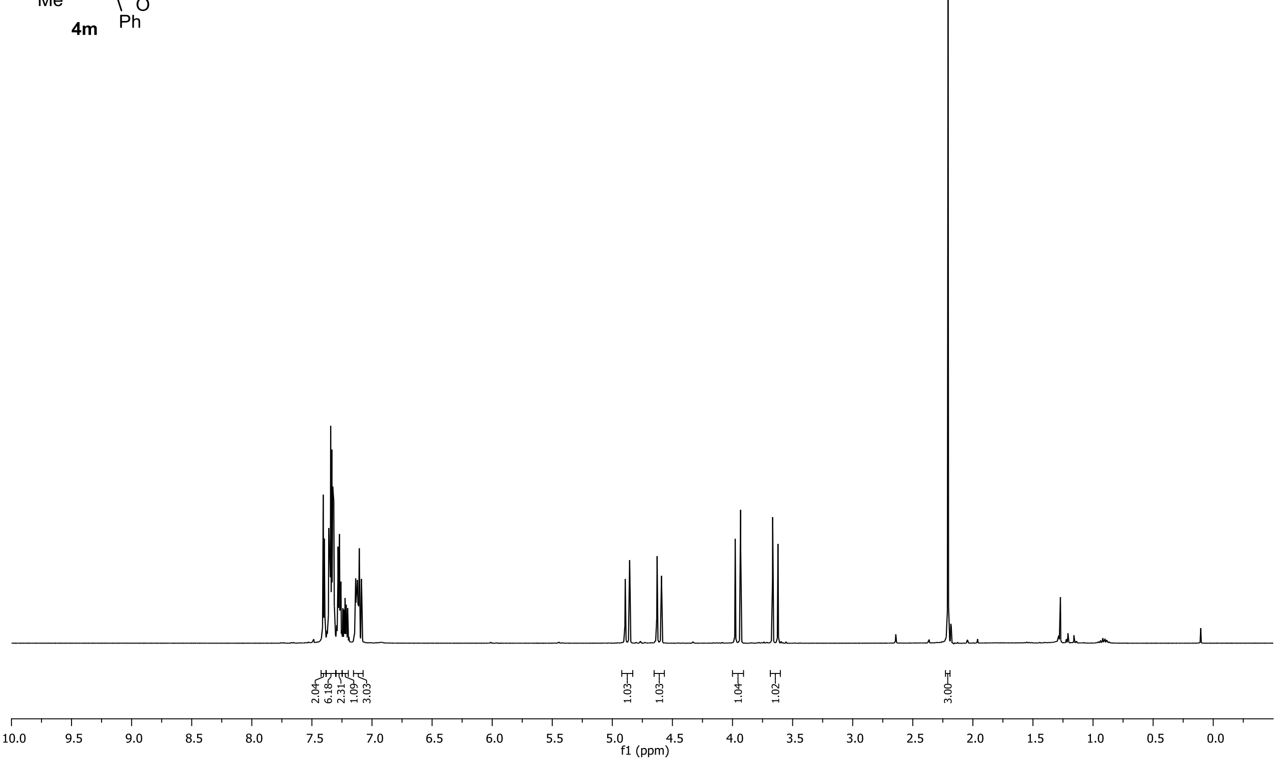
2.04
6.18
2.31
1.09
3.03
1.03
1.03
1.04
1.02
3.00
10.0 9.5 9.0 8.5 8.0 7.5 7.0 6.5 6.0 5.5 5.0 4.5 4.0 3.5 3.0 2.5 2.0 1.5 1.0 0.5 0.0
f1 (ppm)

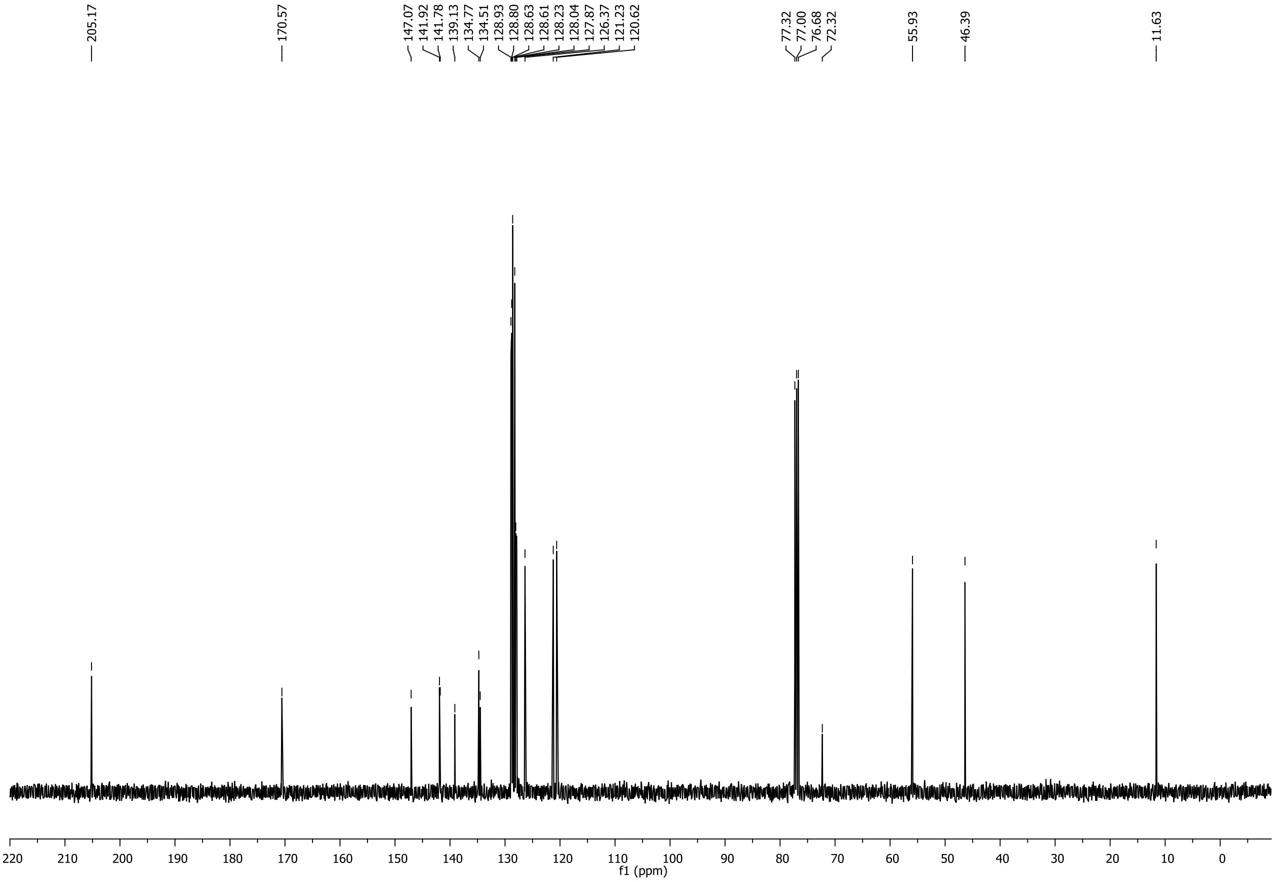
205.17
170.57
147.07
141.92
141.78
139.13
134.77
134.51
128.93
128.80
128.63
128.61
128.23
128.04
127.87
126.37
121.23
120.62
77.32
77.00
76.68
72.32
55.93
46.39
11.63
220 210 200 190 180 170 160 150 140 130 120 110 100 90 80 70 60 50 40 30 20 10 0
f1 (ppm)

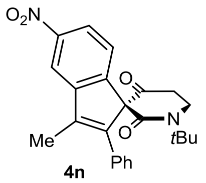
O2N
O
N
O
tBu
Me
Ph

4n

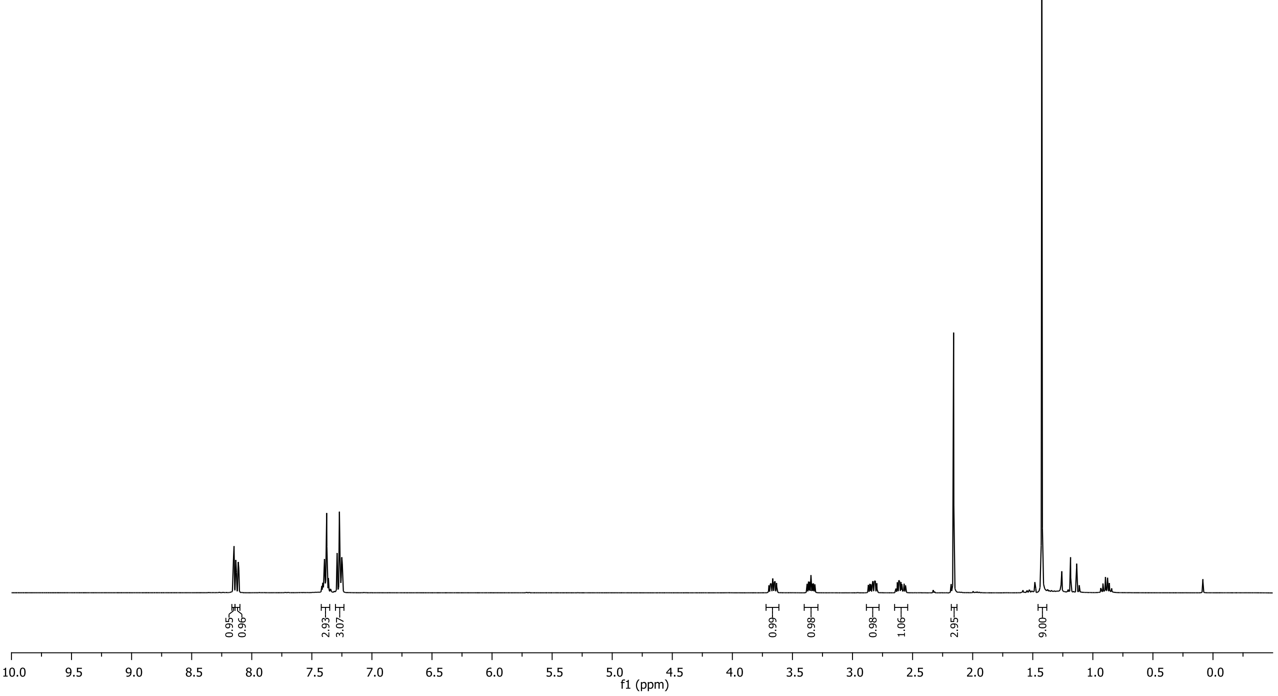
0.95
0.96
2.93
3.07
0.99
0.98
0.98
1.06
2.95
9.00
10.0 9.5 9.0 8.5 8.0 7.5 7.0 6.5 6.0 5.5 5.0 4.5 4.0 3.5 3.0 2.5 2.0 1.5 1.0 0.5 0.0
f1 (ppm)

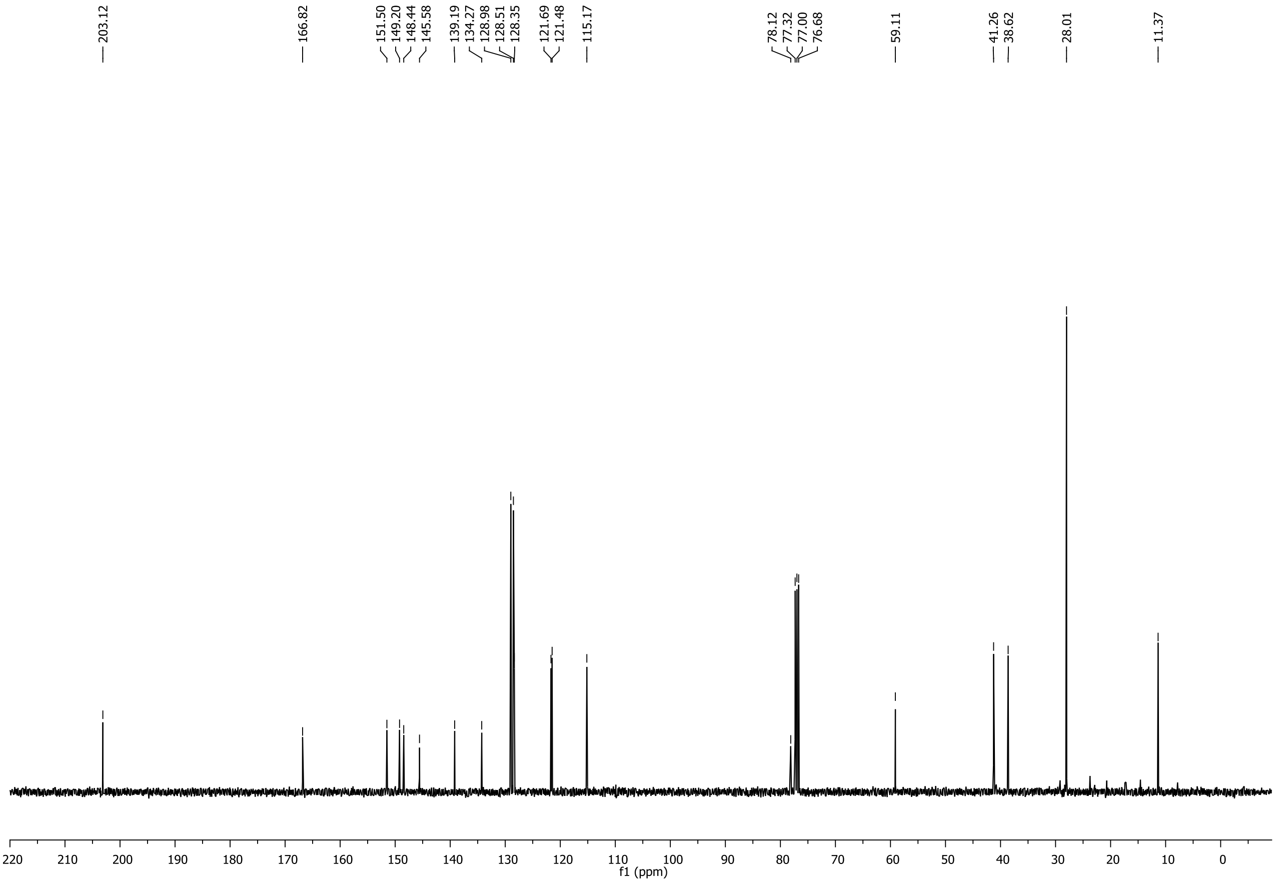
203.12
166.82
151.50
149.20
148.44
145.58
139.19
134.27
128.98
128.51
128.35
121.69
121.48
115.17
78.12
77.32
77.00
76.68
59.11
41.26
38.62
28.01
11.37
220 210 200 190 180 170 160 150 140 130 120 110 100 90 80 70 60 50 40 30 20 10 0
f1 (ppm)

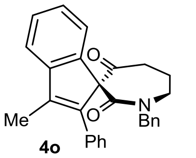

4o

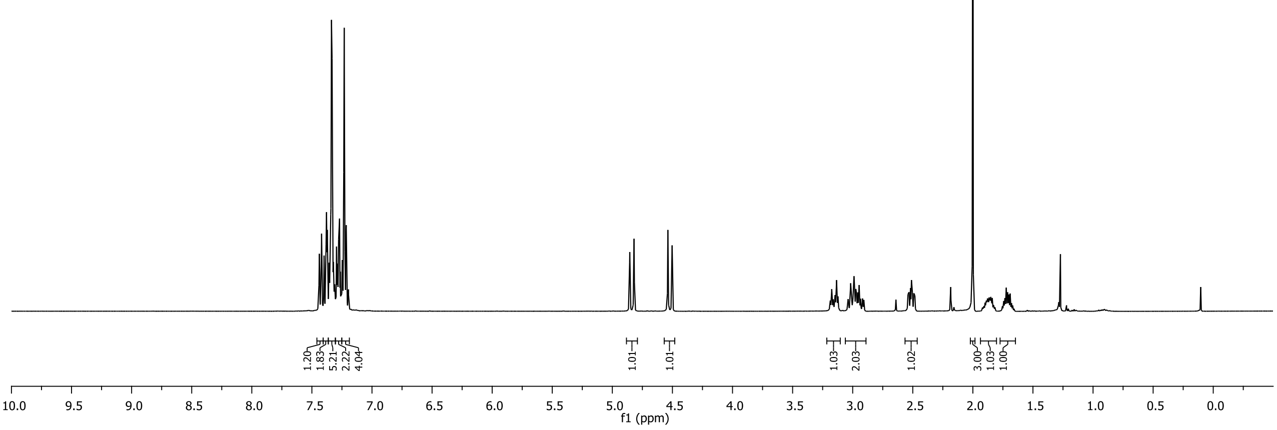

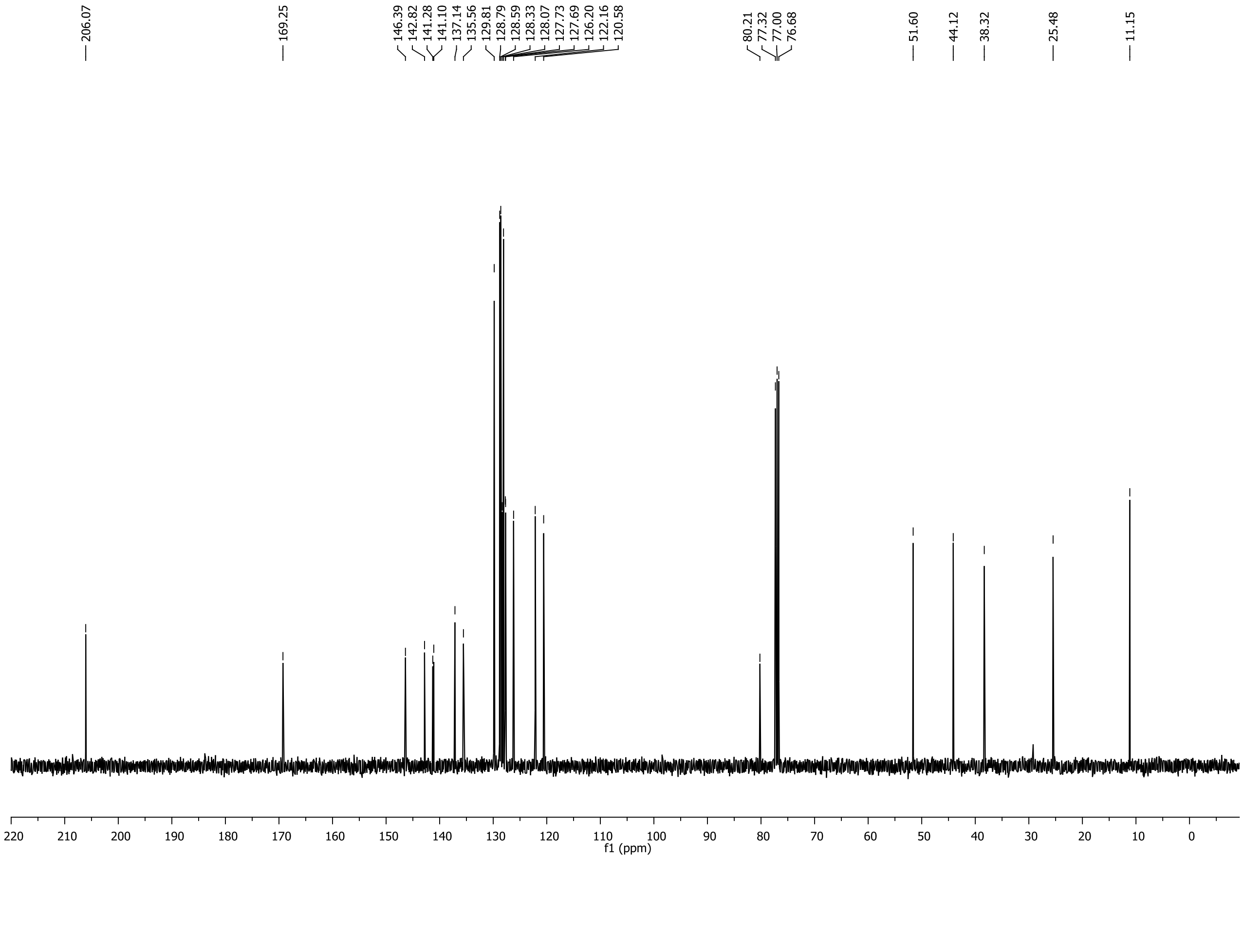

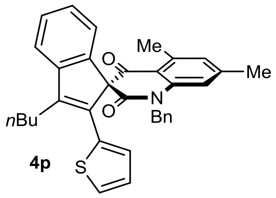
Me
Me
O
O
N
Bn
nBu
S
4p

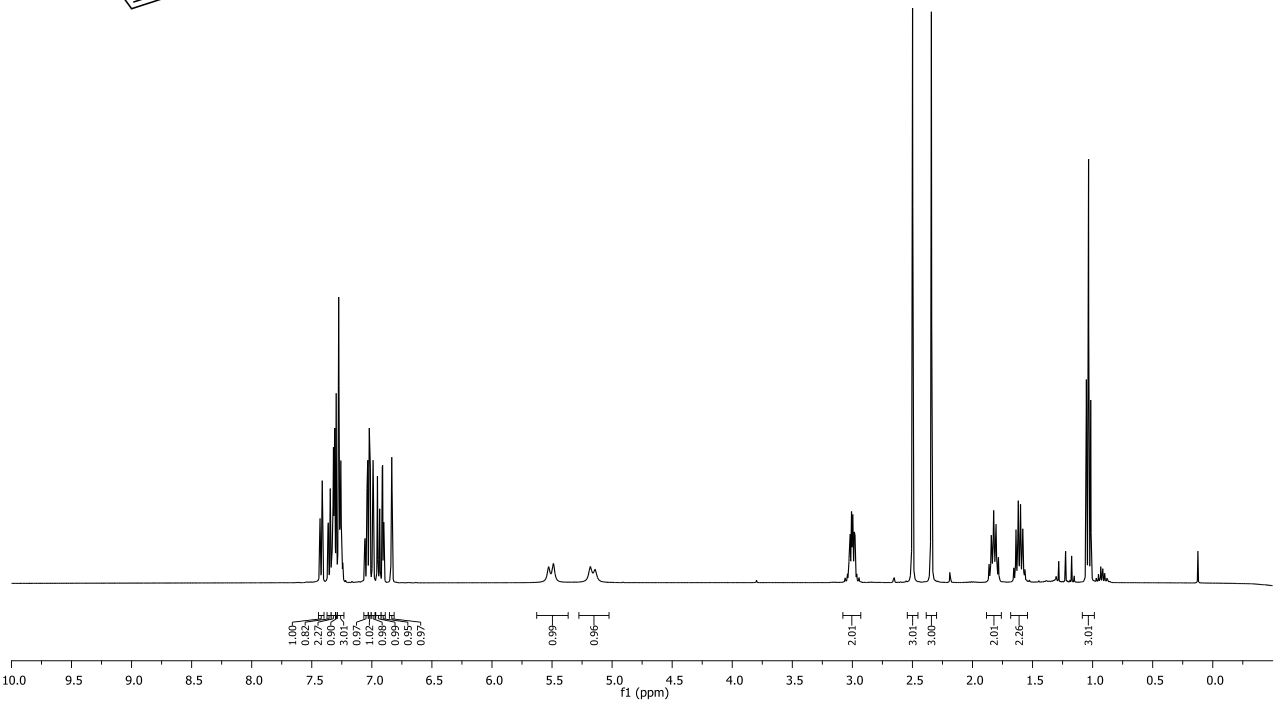
1.00
0.82
2.27
0.90
3.01
0.97
1.02
0.98
0.99
0.95
0.97
0.99
0.96
2.01
3.01
3.00
2.01
2.26
3.01
10.0 9.5 9.0 8.5 8.0 7.5 7.0 6.5 6.0 5.5 5.0 4.5 4.0 3.5 3.0 2.5 2.0 1.5 1.0 0.5 0.0
f1 (ppm)

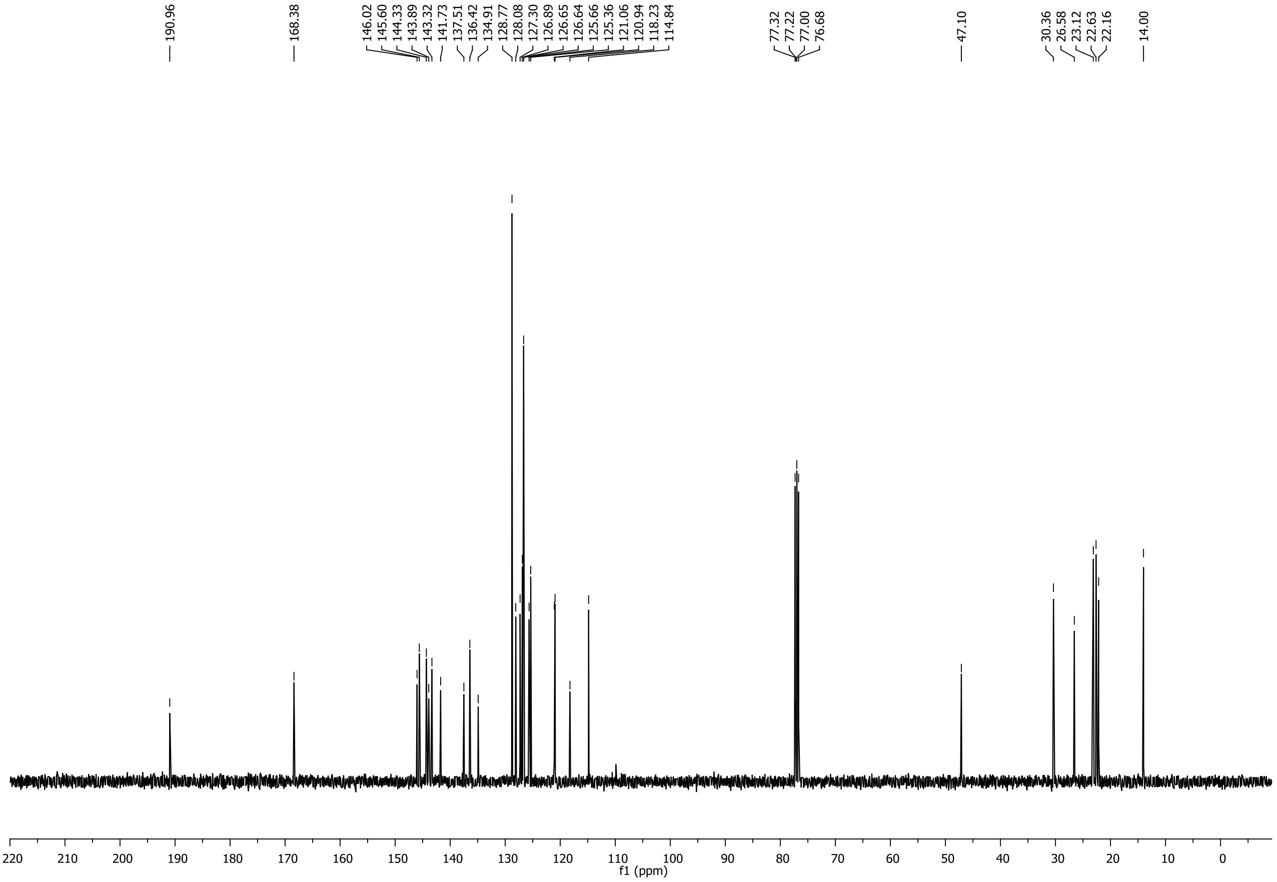
190.96
168.38
146.02
145.60
144.33
143.89
143.32
141.73
137.51
136.42
134.91
128.77
128.08
127.30
126.89
126.65
126.64
125.66
125.36
121.06
120.94
118.23
114.84
77.32
77.22
77.00
76.68
47.10
30.36
26.58
23.12
22.63
22.16
14.00
220 210 200 190 180 170 160 150 140 130 120 110 100 90 80 70 60 50 40 30 20 10 0
f1 (ppm)

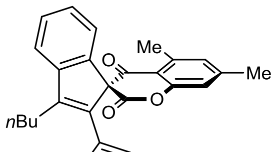
Me
Me
O
O
O
nBu
S

4q

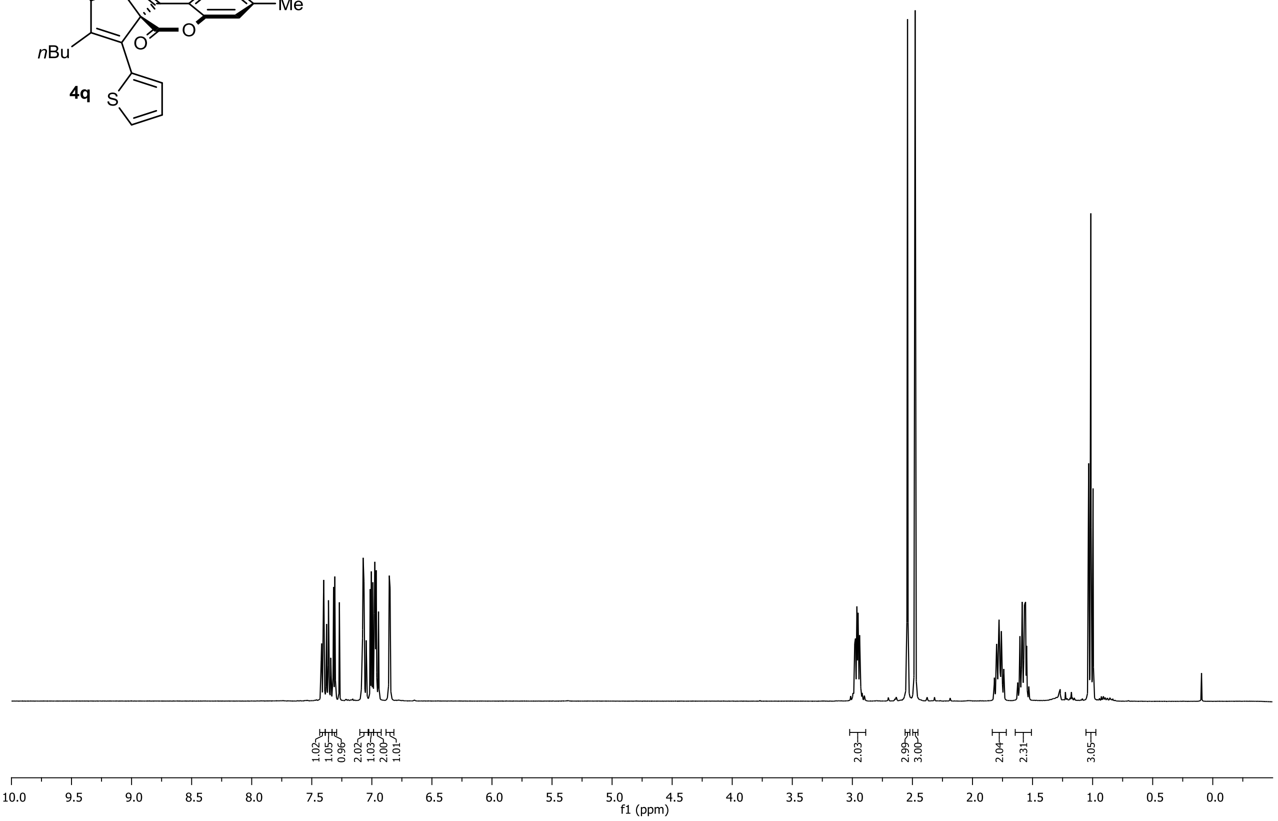
1.02
1.05
0.96
2.02
1.03
2.00
1.01
2.03
2.99
3.00
2.04
2.31
3.05
f1 (ppm)

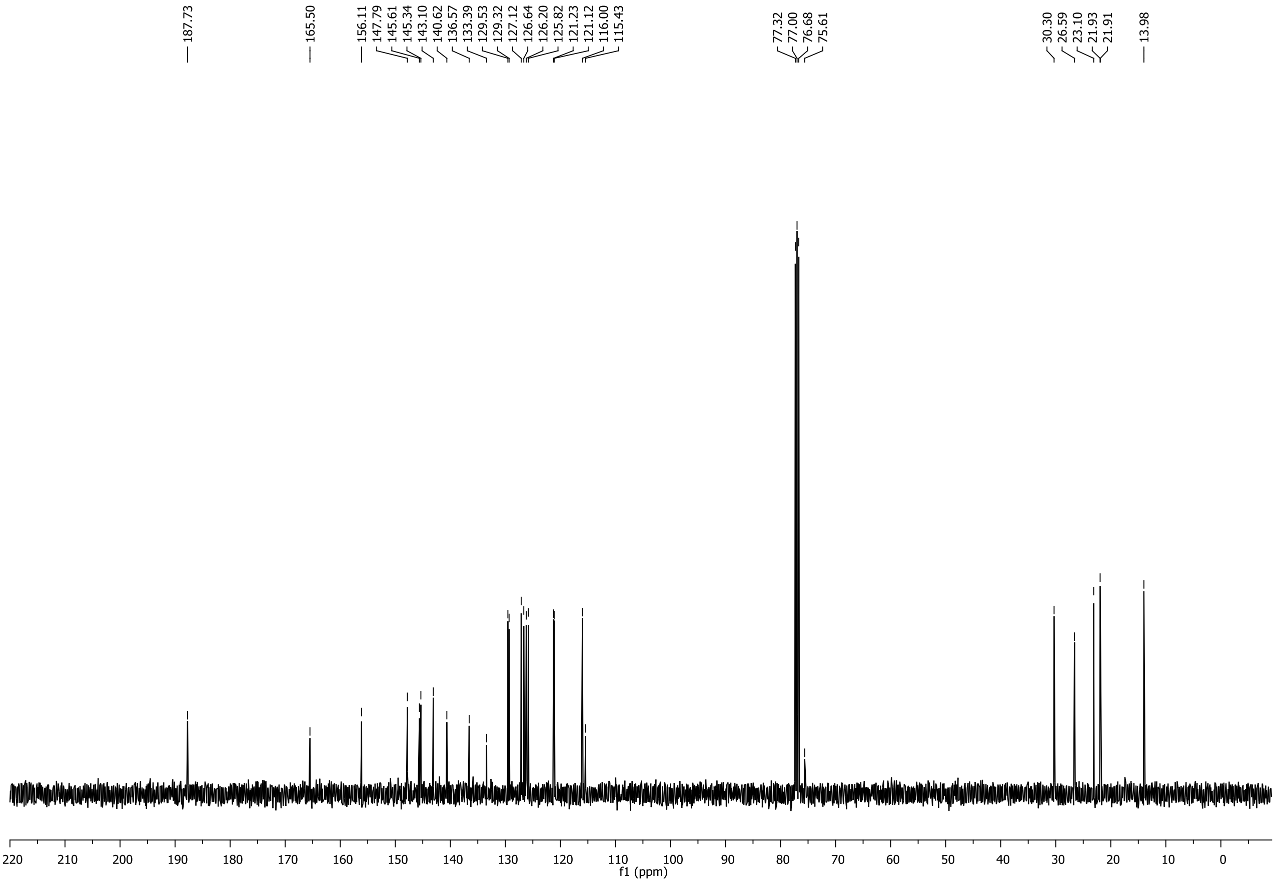
187.73
165.50
156.11
147.79
145.61
145.34
143.10
140.62
136.57
133.39
129.53
129.32
127.12
126.64
126.20
125.82
121.23
121.12
116.00
115.43
77.32
77.00
76.68
75.61
30.30
26.59
23.10
21.93
21.91
13.98
f1 (ppm)

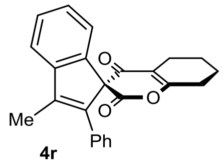
O
O
O
Me
Ph

4r

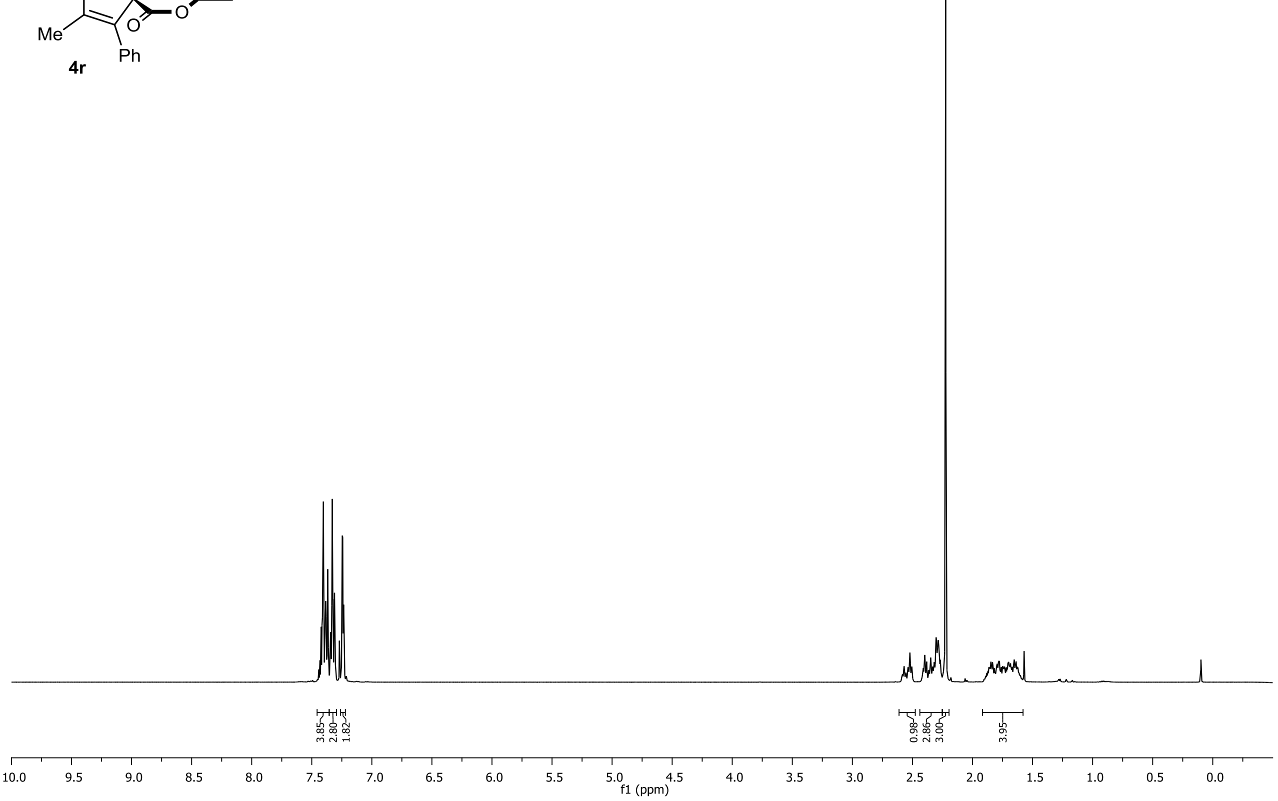
3.85
2.80
1.82
0.98
2.86
3.00
3.95
10.0 9.5 9.0 8.5 8.0 7.5 7.0 6.5 6.0 5.5 5.0 4.5 4.0 3.5 3.0 2.5 2.0 1.5 1.0 0.5 0.0
f1 (ppm)

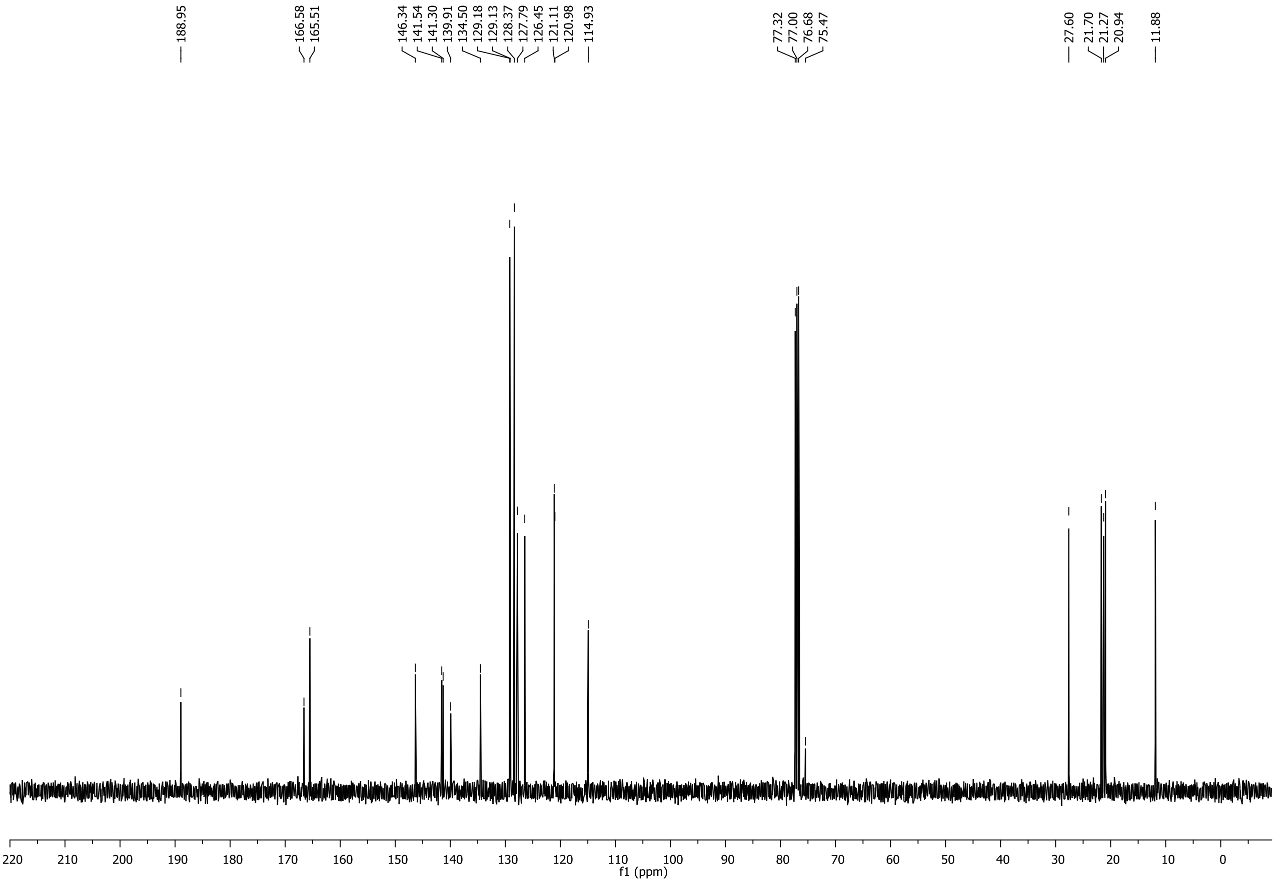
188.95
166.58
165.51
146.34
141.54
141.30
139.91
134.50
129.18
129.13
128.37
127.79
126.45
121.11
120.98
114.93
77.32
77.00
76.68
75.47
27.60
21.70
21.27
20.94
11.88
220 210 200 190 180 170 160 150 140 130 120 110 100 90 80 70 60 50 40 30 20 10 0
f1 (ppm)

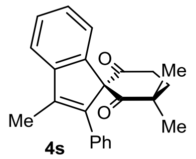
O
Me
O
Me
Me
Ph

4s

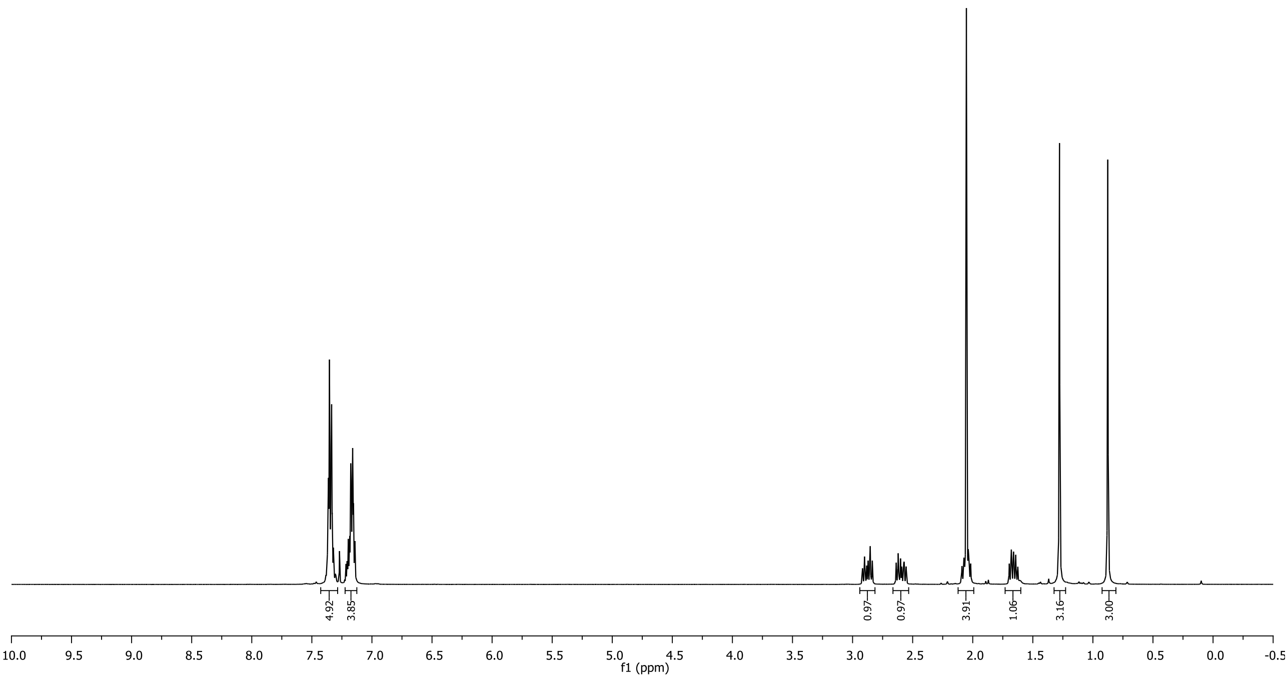
4.92
3.85
0.97
0.97
3.91
1.06
3.16
3.00
10.0 9.5 9.0 8.5 8.0 7.5 7.0 6.5 6.0 5.5 5.0 4.5 4.0 3.5 3.0 2.5 2.0 1.5 1.0 0.5 0.0 -0.5
f1 (ppm)

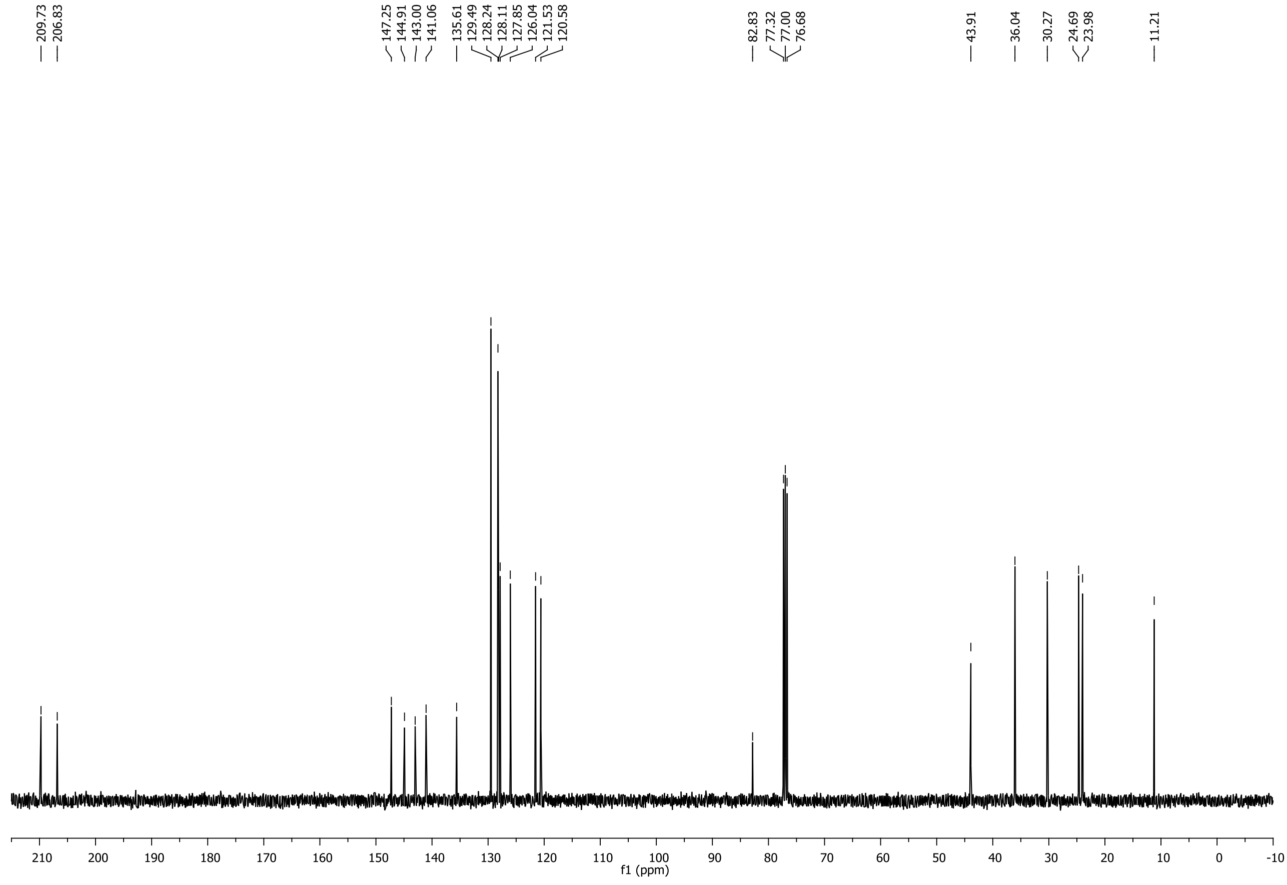
209.73
206.83
147.25
144.91
143.00
141.06
135.61
129.49
128.24
128.11
127.85
126.04
121.53
120.58
82.83
77.32
77.00
76.68
43.91
36.04
30.27
24.69
23.98
11.21
210 200 190 180 170 160 150 140 130 120 110 100 90 80 70 60 50 40 30 20 10 0 -10
f1 (ppm)

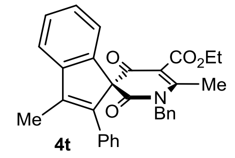
CO2Et
Me
O
N
O
Bn
Me
4t
Ph

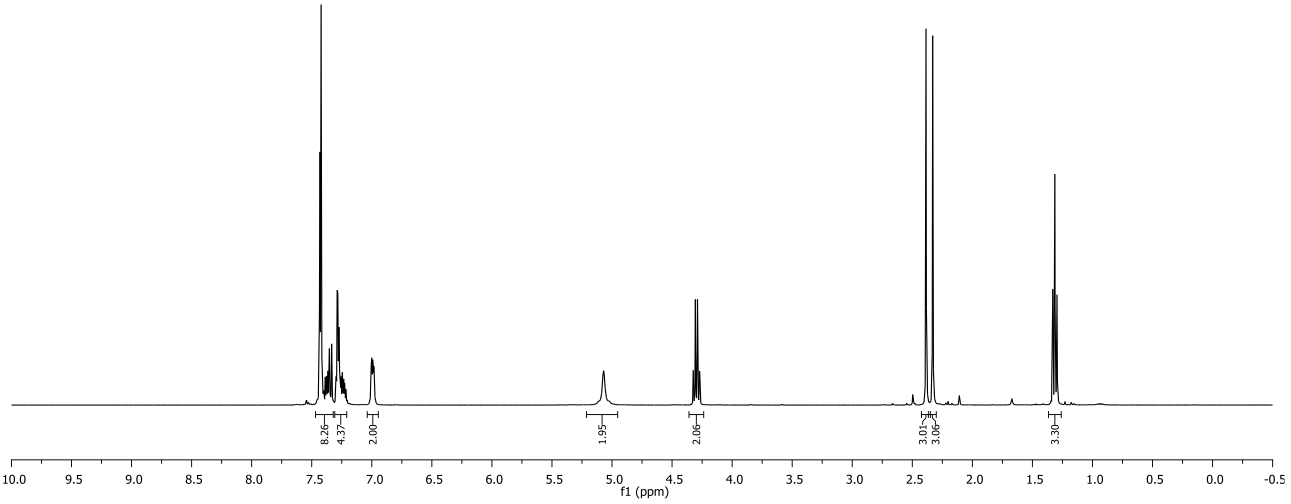
8.26
4.37
2.00
1.95
2.06
3.01
3.06
3.30
10.0 9.5 9.0 8.5 8.0 7.5 7.0 6.5 6.0 5.5 5.0 4.5 4.0 3.5 3.0 2.5 2.0 1.5 1.0 0.5 0.0 -0.5
f1 (ppm)

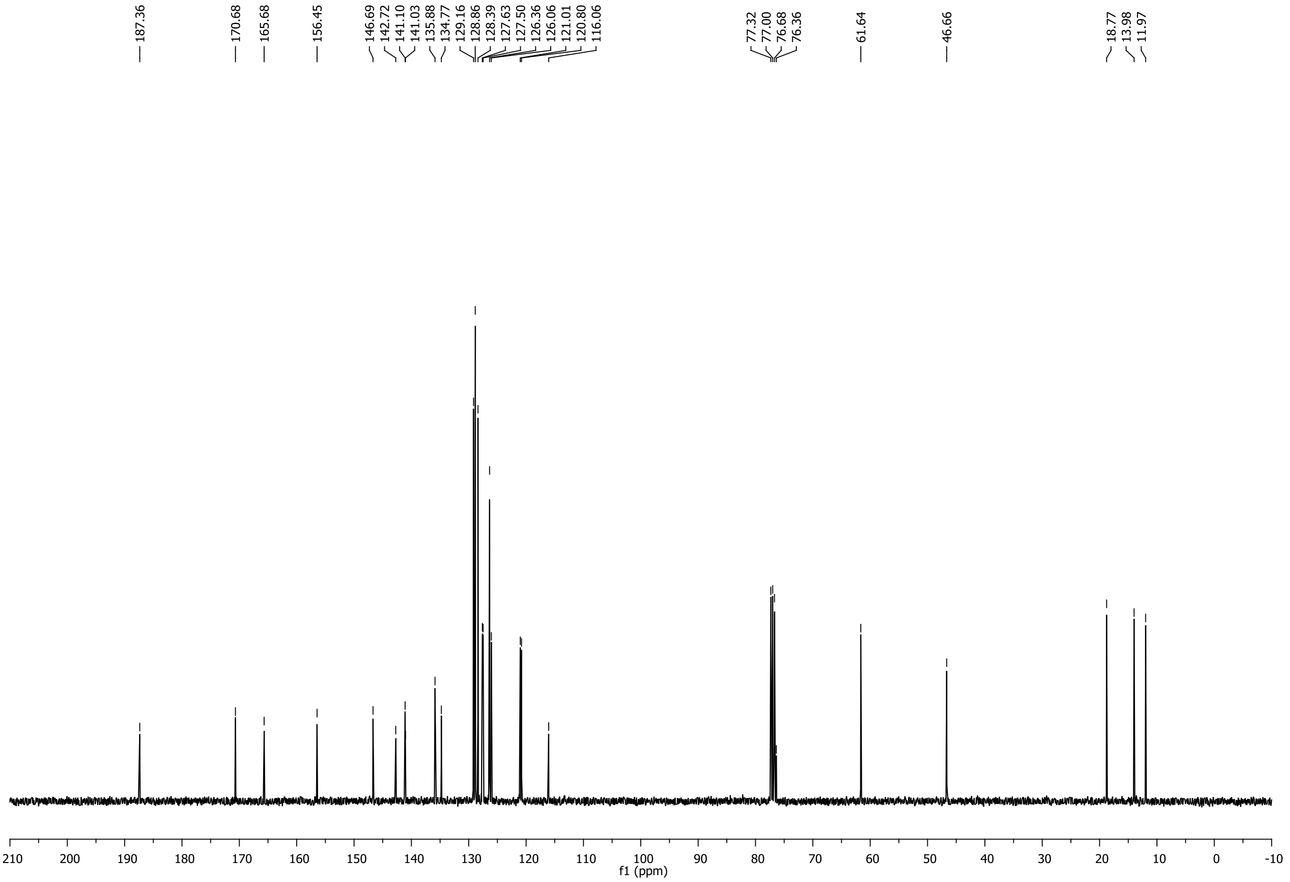
187.36
170.68
165.68
156.45
146.69
142.72
141.10
141.03
135.88
134.77
129.16
128.86
128.39
127.63
127.50
126.36
126.06
121.01
120.80
116.06
77.32
77.00
76.68
76.36
61.64
46.66
18.77
13.98
11.97
210 200 190 180 170 160 150 140 130 120 110 100 90 80 70 60 50 40 30 20 10 0 -10
f1 (ppm)

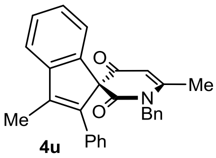
O
Me
N
O
Bn
Me
Ph

4u

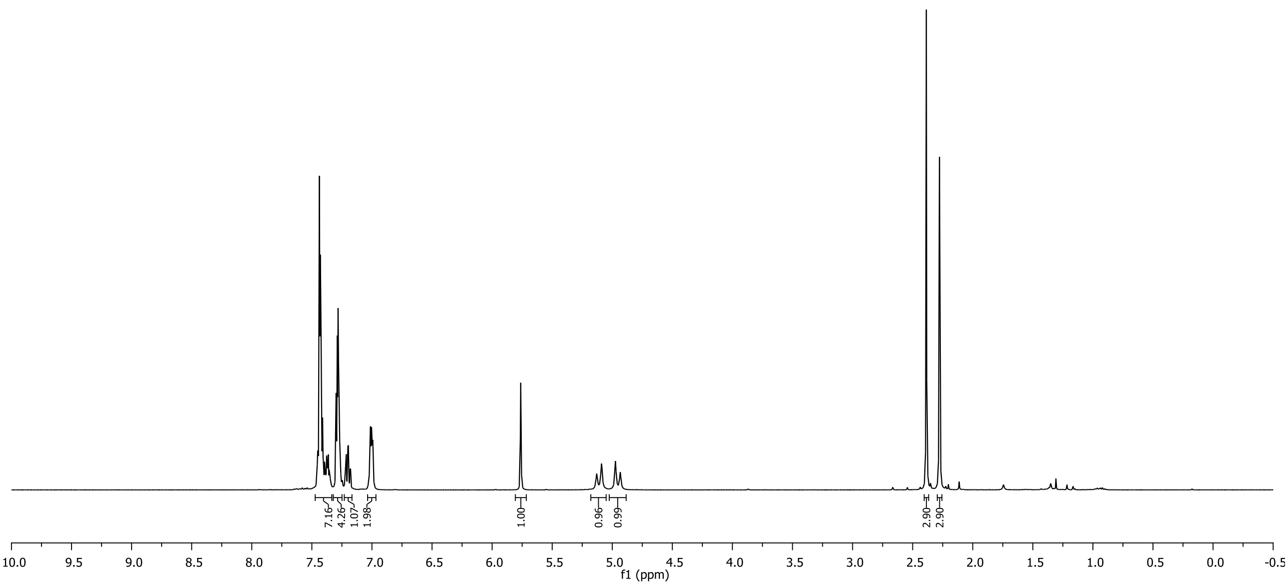
7.16
4.26
1.07
1.98
1.00
0.96
0.99
2.90
2.90
10.0 9.5 9.0 8.5 8.0 7.5 7.0 6.5 6.0 5.5 5.0 4.5 4.0 3.5 3.0 2.5 2.0 1.5 1.0 0.5 0.0 -0.5
f1 (ppm)

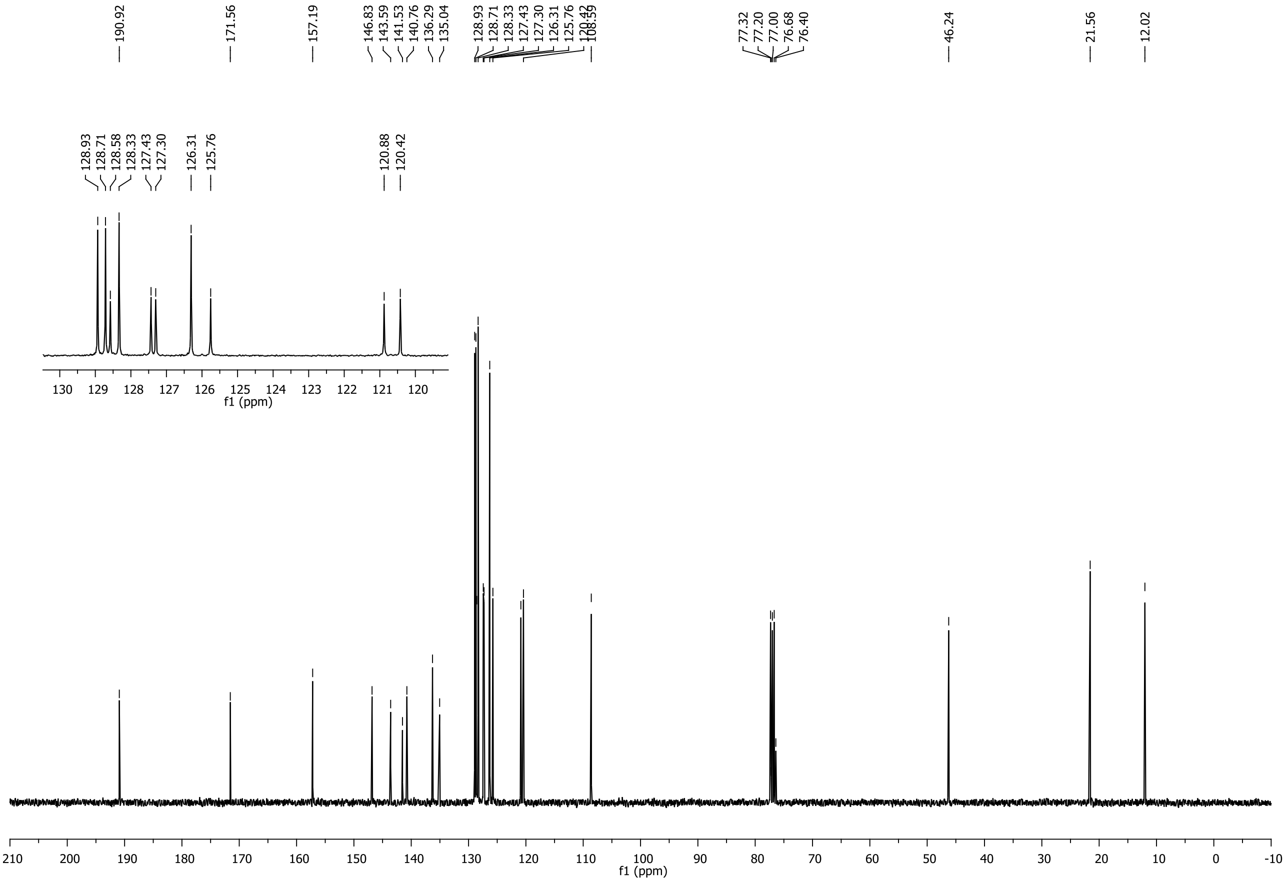
190.92
171.56
157.19
146.83
143.59
141.53
140.76
136.29
135.04
128.93
128.71
128.33
127.43
127.30
126.31
125.76
120.42
108.59
77.32
77.20
77.00
76.68
76.40
46.24
21.56
12.02
128.93
128.71
128.58
128.33
127.43
127.30
126.31
125.76
120.88
120.42
130 129 128 127 126 125 124 123 122 121 120
f1 (ppm)
210 200 190 180 170 160 150 140 130 120 110 100 90 80 70 60 50 40 30 20 10 0 -10
f1 (ppm)

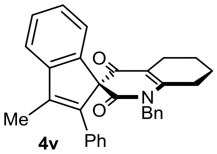
O
O
N
Bn
Me
Ph

4v

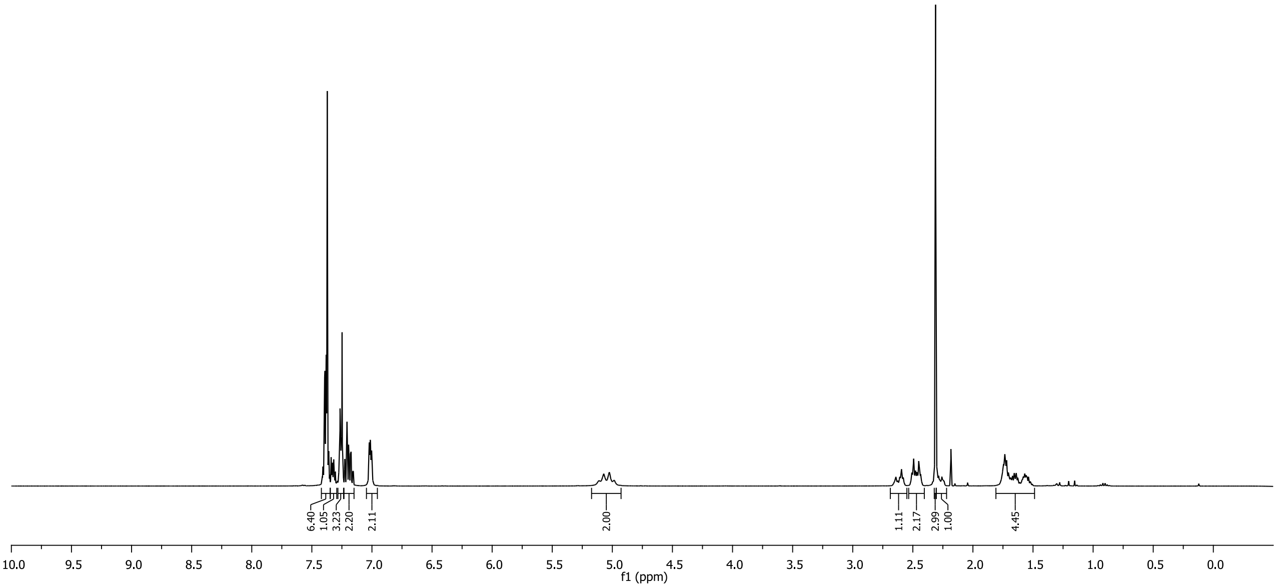
6.40
1.05
3.23
2.20
2.11
2.00
1.11
2.17
2.99
1.00
4.45
10.0 9.5 9.0 8.5 8.0 7.5 7.0 6.5 6.0 5.5 5.0 4.5 4.0 3.5 3.0 2.5 2.0 1.5 1.0 0.5 0.0
f1 (ppm)

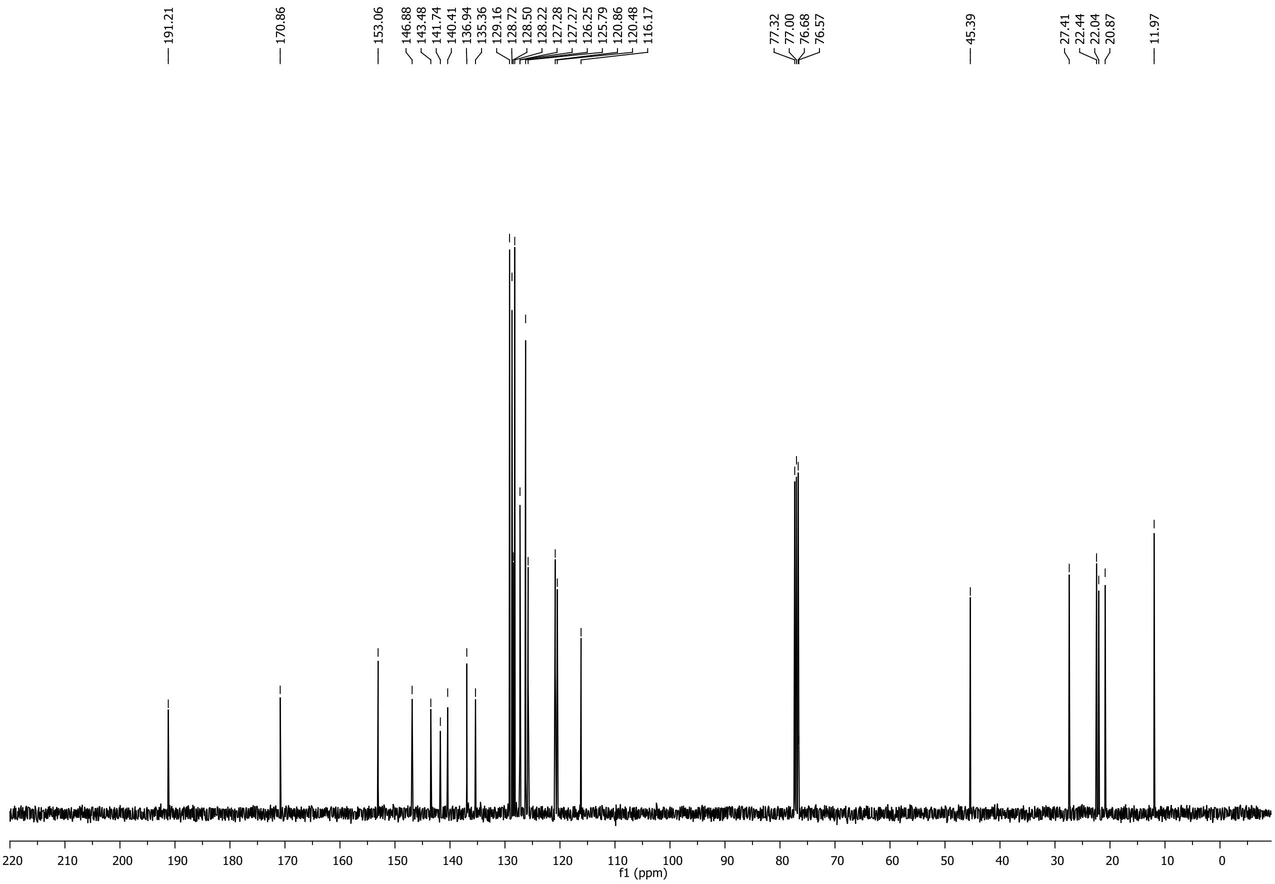
191.21
170.86
153.06
146.88
143.48
141.74
140.41
136.94
135.36
129.16
128.72
128.50
128.22
127.28
127.27
126.25
125.79
120.86
120.48
116.17
77.32
77.00
76.68
76.57
45.39
27.41
22.44
22.04
20.87
11.97
220 210 200 190 180 170 160 150 140 130 120 110 100 90 80 70 60 50 40 30 20 10 0
f1 (ppm)

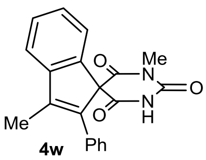
Me
N
O
O
N
H
O
Me
Ph

4w

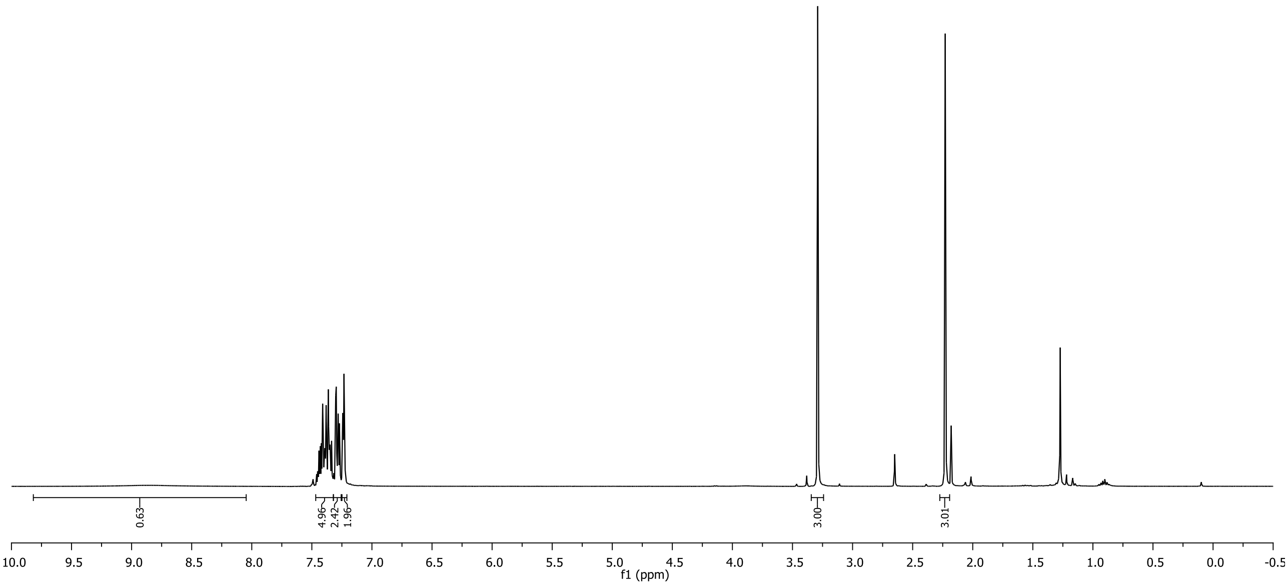
0.63
4.96
2.42
1.96
3.00
3.01
10.0 9.5 9.0 8.5 8.0 7.5 7.0 6.5 6.0 5.5 5.0 4.5 4.0 3.5 3.0 2.5 2.0 1.5 1.0 0.5 0.0 -0.5
f1 (ppm)

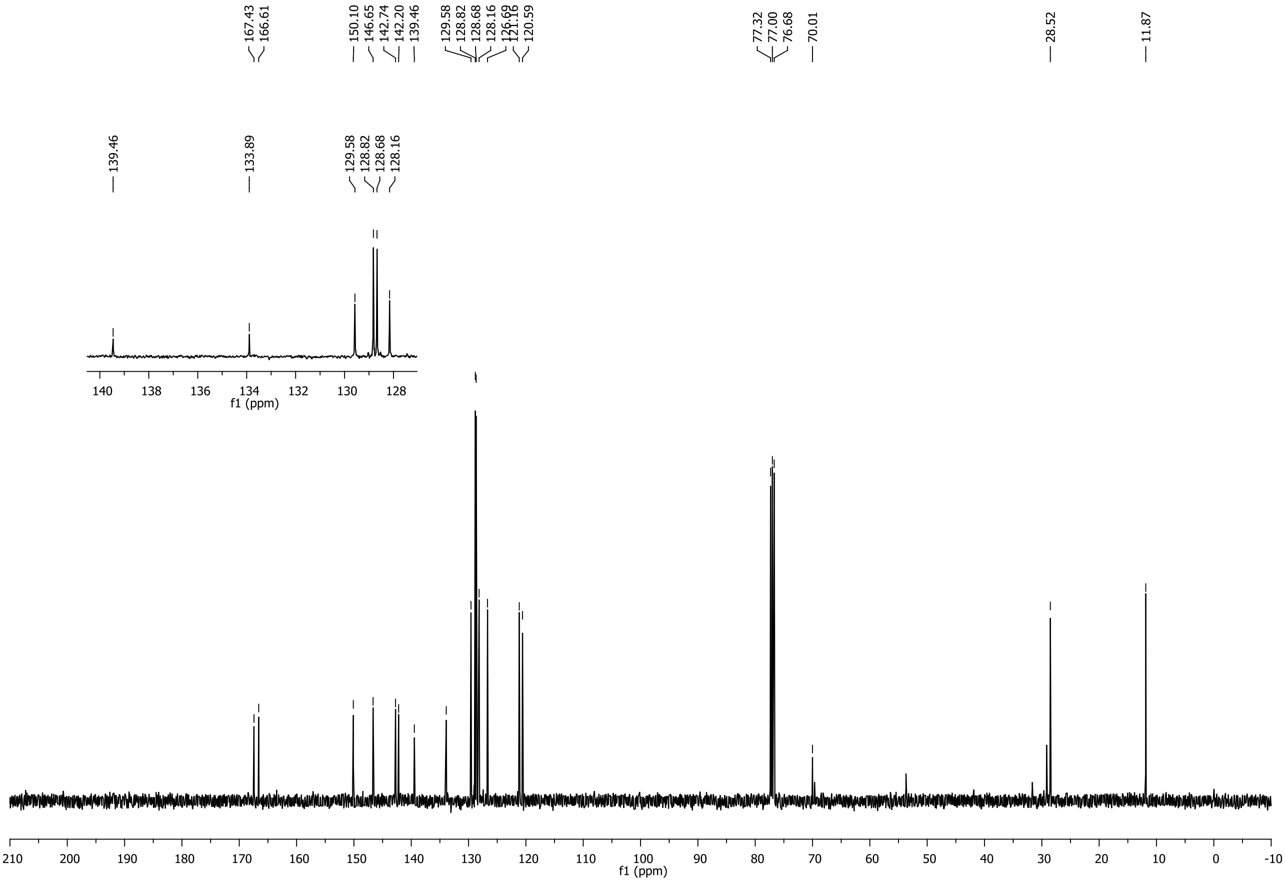
167.43
166.61
150.10
146.65
142.74
142.20
139.46
129.58
128.82
128.68
128.16
126.69
121.16
120.59
77.32
77.00
76.68
70.01
28.52
11.87
139.46
133.89
129.58
128.82
128.68
128.16
140 138 136 134 132 130 128
f1 (ppm)
210 200 190 180 170 160 150 140 130 120 110 100 90 80 70 60 50 40 30 20 10 0 -10
f1 (ppm)
